# Supplementary material for: Identification and expression profiles of candidate chemosensory receptors in Histia rhodope (Lepidoptera: Zygaenidae)
Source: PeerJ. 2020 Sep 24;8:e10035. doi: 10.7717/peerj.10035 (PMC7520089; doi:10.7717/peerj.10035)
Supplement: File S1 [file peerj-08-10035-s001.docx]

**The amino acid sequences of ORs used for building phylogenetic trees are listed below:**

>BmorOR1

MLLSFKDDSRSPDIQKPQNFQYMKILRFNLKIICAWPEKQLNEIRSLGHSIHRVILPIQSVVCLACGILYIHFHFNEIPFFILASTFITVMMNLVTCSRTALVMLFERYLVLTGRFITVMHLFNFQKNSDYAYKLCTFVNRMSHFYTLYVLFSMFMGLGLFNLLPLYNNYVSGAFSDPYGPNVTFFHSVYFAFPFDYSHNFRGYIIMALFNSYVSVTCSIGLVMFDLLMCLMVMHVWGHLKILSHNLINFPRPKASHVITTPNGPTNVETYTEEESKEVFARLRECIKHYGTVDDFANDMSETFGVILLVYYGFHQVSLCMLLLECSDLSTKAMLRYGPLTLIMIQQLIQISIIFELLGSVADRIPDAVYQLPWECMDVKNRRVVYGFLRRTQNPVRFKAMGMLDVGVQTMASILKTSISYFVMLRTVAT

>BmorORco

MMTKVKTQGLVTDLMPCIRLLQAAGHFLFNYHADTSGMNMLLRKIYSSAHAVLIVVHYICMGINMAQYKDEVNELTANTITVLFFAHSIIKLAFFAFNSKSFYRTLAVWNQSNSHPLFTESDARYHQISLSKMRRLLYFICGMTVFSVISWVTLTFFGESVRMIASKETNETLTEPAPRLPLKAWYPFKTMSGGGYVFAFIYQIYFLLFSMALANLLDVIFCSWLIFACEQLQHLKAIMKPLMELSAALDTYRPNTAELFRVSSTDKTEKVPDAVDMDIRGIYSTQQDFGMTLRGAGGKLQNFNAENNPNGLTAKQEMLARSAIKYWVERHKHVVRLVASIGDTYGTALLFHMLVSTITLTLLAYQATKINGINVYAFSTIGYLVYTLGQVFHFCIFGNRLIEESSSVMEAAYSCQWYDGSEEAKTFVQIVCQQCQKAMTISGAKFFNVSLDLFASVLGAVVTYFMVLIQLK

>BmorOR3

MIFVDDAVIGIKDPREYRHLRVLRTSLRLLGAWPGHYLGEETGSKYECAPMFLLMFIKIACLYLTIVYLRNNADVLGFFELGHVYLTIFMTFVTLSRGFSLTWNPNYHKVVKKFITEMHLLYFKDNSEYAMKTHRRVHKISHFYTVFLKVQMIAGLTLFNVIPMYNNYRQGNYASDRPANITYDLSIYYETFDILNTPNGYIFICVFNWFASYICCSFFCSFDLILSLMISTVSGHFRILIHNLLTFPLPEAITASKKFVDKHRCNGNRSEFVLEEAKLYSPAEMWQVTDRLRQCIDYHRKLVEFTGDISEAFGPMLFVYYLFHQVSGCLLLLECSQLNTAALVRYGVLTVVLYQQLIQLSVIVESVGTVTGRLKDAVYEVPWEYMDTSNRKTVAIFLMNVQEPLHVNALGLAKVGVQSMAAILKTSFSYFTFLRTVSE

>BmorOR4

MFKIIKNIIVENDALKQVEKPQEFQYMKWVQYHLKYIDGWPNMDMNKKNVSKIRFHKRHLLVVEQTITFLSQMFYIVKNYGKLSFFEIGHSYITALMTIVIFSRSVVTALGRYRKIARYFVSSLHLYHYKDISEYALQTHLLVHRLSHYYTVYLISLVVTGMLLFNITPLYNNISSGVFNSPRPENMTFQHAVYLGLPFDYTTDIKGYFVVFILNWHLSHIAASYFCTFDLFLSLLILHLWGHLRIILNNLKTFPKPYTNNSMYTEEENQVVLLKLQECIRYHNFIISFTVMMSNVYDVVIIVYYLFHQVTGCLLLLQCSTLDWESLSRYGPLTLIIFQQLIQVSMIFEILGFLSDKLPNAVYSIPWEAMNVTNRKLVQVLLQKSQKPIQFKAMNMMSVGVQTMASIIKTSISYFIMLRTIARD

>BmorOR5

MLLYYPNTQVKEKVNNVEEFTYIKFLKSFCKIMDFWPEREEKNSKTRIFRLRYILVLQFCFTLVAGVLYLTNSVGKQTFYDLGHTIITVLMNVVSLSRLILRCFKKYDVVGQQFINKIHLYHYRNDSEYAMKIHTVVHKISHNMTYIFSFCIIFGTVTFNLTPIFNNIGSDAYKNPRPDNVTLQQCVYYALPFDYTGNFKWYLLVAIFNVQKTFFCTSLFILFELSLSLMIICLWGHLRIFIHNLNHIPAPRNSFEYTKEERQEVDDTLKKCIQHHTLIIGFVRIMSETYGLAVLIYYAFQQVVGCLLLLQCSQMELKTVTRFGFLTLVLNQQLIQISVIFELLGYMSDKLQDAVYCVPWEYMDTSHRKMVYMMFRQSQIPLQLKAMNMLSIGVKTMVSILKTSVTYYLILKTVTTD

>BmorOR6

MKEEYYLQHPRTQLFYKVLAHVSTIESTIDLTWWGYTFPKYVGWFYHLQCNVVRLFGKCVVVSQILFIILNYQTIDKSVFIIAITITPLGALVGIKAESAKAECYVNLMKNFMDKVHIHSIYRKNENNEFVKKKVIQIERVSRFTAYFLVILIAINCLSWMLKPTLHNIKHFEEIMNKSMEFQYYIYFWTPLDYKYNLRDYIIIHTLCIYLGATAVTVIVTFDIFNFIAVFHVVAHIQILKNNVKSNWSDDFNESEKKGYLVSILEYHAYIIRIFGEVQSAFGLNVASNYLQNLIEDGLFLYQIMNGEKENVLMYGLMIILYLGGLIFLSIVLEEIRRQNYDLCEYVYALPWEGMSLENQKIFVVFLQRTQPDLEFETVCGMKAGVKPAFSIVKSMFSYYVMINSRF

>BmorOR10

MRTNAKSFLFVPSKVLTLCGVWPVEKTSIFSLIYRSIMLSSQFCFLVFNGIYIGLMWGDLKAVSDALYMFFTQTTCCSKAIGFYFNFMKIKRIVASMDDVLFTAMSIEDQATIFSHSRTVNKLYKGVLGFTGFTLVQWTVLSLIGSGRTLPFNEMWVPTDISKSPNYEITFVVELWMMVISAALFMSVDTITVATMMFSCAQLDIIMKKTQQIQEIPLSPDLSSRNRSELHEKNNGILIDCIKQHQAIVRFSELCEGTFQVHSFFHLGGIVFMICVIGFRMAGESPVSAQFWAALSYLVIILGQLYLYCWCANELTTKSEQLRDKLYLTPWYDQDVKFKRNLCIAMECMAKALTFRAGSYIPLSRAMFVSILRSSYSYFAFLNQANEQ

>BmorOR15

MMTLVYQTDIFKPNVFFWKMFGIWADRKSSKTYKYYSFVFLFITLIMYNSLLAINLLYTPLKIELLIREVIFCFTEITVTTKVLMILFKRNKILDAFDLLNKNEFRGNSEESSAIIQKNNSAYKTYWKLYAILSNFAYSSQVLGPLIVKLIWKTKLELPICNYYFLNEELRHDFFSGWYIYQSFGMYGHMMYNVNIDTFISGLLMMAVTQLKIIQTKLLSLKLNPRERKMDRGLMNITEVLKLNEILKHYELVLKYCSTVQSILDVAMFVQFGVASAIICVAMCGLIMVRSSTETLLFMVTYLFAMTLQIFVPAWMGTQLHFQSQELVFAAYNSEWIPRCQSFKRSIIIFVERAKIPITITGLKMFPLSLATFTSIMKTAYSFFTLIRNMQTLQEE

>BmorOR19

MHEFVINVQNETTKLYDQLNIILYILGLQGIWVDEIKLSRRFHVFFKVVTFILHIMCGMFAGLQFFAIFTQNSLNSQQKSDVIVIGISNPMAYIFCINFIRNRNEIKDLFYHLAVVLKIYYNDVEIEKSMVNKIKSYLSTYVFASITILVSNGIIAFFQTINSDEPFLGIITAWPDKTDTSKTASYARIGFYLFWCIHFFRISTVFAVIVCILISIKYQYKFLCSYFESLNKIFDDETSSHEVKEAEFENAFCNGIKIHTQIIWCVRRCQIMCRTVFSANIMLDTFVLVILMLAMVNSENDFYGLCSQMSSVLVTVVLMAFFMWTAGDINVQASQLPDAIYGSGWYNCRGKSSARIRSLVTISMNKAQQPILMWALGFVELSHKNFVAIIKSAYSVFSVFY

>BmorOR41

MMGNSTDLFLDRTKSILNFFAMWRSFEKPIPLKVYMAFIMTTQYLFLIFEIIYIVNVWGDMAEVSEASILLFTQASVCYKITSFISKTNNFVILLGLIESEIFSAQTELHEKILILKARKIKRLCMFFLVNAVTTCSLWAVIPLLDISSKMLPFKIWMPASTGESPHYELGYLYQMITIYISAFLFIGVDSVPLSMIMFGCAQLEIIMDKIGKVKSRPLDQQPMQRQAVLNSNYELLVECVRRYQSVVRFIELTEKTYHANIFFQLSGSVLIICNIGFRIAIVDSNSLQFYSMLTYLVTMLSQLFQYCWCGHELTIRGEELRETLYQSPWHEQDIRFRKVLIITMERMKRPIIFKAGHYIPLSRPTFVAILRCSYSYFAVLNRVRNE

>BmorOR7

MLLYHPNTQVEEKVNNVEEFTYMKFLKSFCKIMDFWPEREEKNSKTRIFRLRYILVLQFCFTLVAGVLYLKNNFGKKTFYDLGHTIITVVMNVVSVSRLILRCFKKYDVVGQQFINKIHLYHFRNDSEYSMKTYKAVHKISNNMTYIFSFSIFVCVVTFNLNPVFNNIGSGAYKNPRPDNVTLQQCVYYALPFDYTGDFKWYMLVAIFNVQKTFFCTSLFILFDLLLSMMIIHLWGHIRIFIHNLNHIPAPRNSLEYTREERQEVDNTLKKCIQHHTLIIGFVRIMSETYGLAVLIYYAFQQVVGCLLLLQCSRLDLKTITRFGFLTTMVNQQLIQISVIFELLGYMNDKLQEAVYCVPWEYMDTSHRKMVYMMFRQSQIPLQLKAMNMLSIGVKTMASILKTSVTYYLMLKTITANEA

>BmorOR8

MSLSTRCLLKDFCKYVYYAGAGNFWYEDIYKETVPYKMYVVISFFTYTVMIFLENLAALFGKLPEVEKNSAVMFAAIHNIVLTKMFLLLYHKRSISKLNCEMAAVGENLEEASIMRRQFRKMRLGTALYFISVYLSLVAYGVESARRTIVEGAPFYTVVTYLPDYDNTTVLASFLRIFFYITWLYMMLPMMSADCMPIAHLITMTYKFVTLCRHFDQIREKFQINVKIMAKTEATEILKLGFIEGIKMHQKLMYLADEIHRVFGIIMALQVCESSAVAVLLLLRLALSPHLDLTNAFMTYTFVCSLFLLLALNLWNAGELTYQASLLSNAMFYSGWYFCDFEKDWCRDIRRLVLIGCAQAQKPLILKAFGVLDLSYETFVSVARMTYSVFAVFYKRGD

>BmorOR9

MVARRPLQFHQGRNVDNVEDFKYVKWLRNHLKTVDAWPVHSKSKRKIQKRYVLPIFSAACFISQTVYLKNGIGTLSFVVLVHSYICFLINGSCLCRGILIATERYKRLATCYLKTVHLFHHKNRSEHAMKIHVIVHRLSHYYTIYLISLVFVGMVLFNFMPIYNNINSGAFKSPRPENVTFQHAMYLALPFDYTTNIKGYFVVFILNWYISLVTTSHFCTFDLFISLMIIHLWGHIKILMCSLEDIEGFVPGSSFKFTIEQNRKIYLILQECIRHHQFTIDFTNEMSSTFGLVILFYYFFYQVSGCLLLLACSQMDIESLSRFGPMTFILFQQLIQLSIVFELISSLSENLPNAVYNVPWEFMDKNNRKMIQVLLLQSQKLIQFKATSMMNVGVQAMATILKTSVSYFIMLRTMYQEH

>BmorOR11

MDEHSHFETSLNKIKVLFKYSGMNLENTVTNTYEFLNHRWVYILNHAWTLAAVTFICIGISNGQNFIEMTCIAPCVAMTVLAVSKSFFHYINENAVKSLLENLIELERTDFERTKSVQRTEIVATEKQLLNMVINVLYVLNCSMILVFDMTPLIIIAIKYWTTNKFVRLLPYLDIFVFVPYKFEYWVMAYILQIWAECIVLLFIGAADCLFFTCCTYIRIHFRLLQYDFERLTSSRRESDGLRDDEDFRETYTNLVKRHQGLIESSSILEMIYSKSTLSNFVLSSLVICLSAFNVTVVNDVTIVMTYLIFLAMSLMQVYFLCFFDMLMSASEEVGNAVYNCSWYTEKASTGKDLLFTITRAQKPCELTAAHFAYVNLKAFMRVSFTSASITTLPTI

>BmorOR12

MTRITDVFSLNFIFWKFLGLWGKSAPSKYNMAYTVFYLFASLFVYDIFLTLNLIHTPRKLETLVRETMFYFNHLVAVTKILMMFIMRKKILVIFDLLDCEEFKPNDENSQEIMKRKTDFYYIYWRIVAVTSNLSCFMLVIGPLIKMLIWKIELGLPVCKFYFMSDELRNKYFVIWYIYQSFGIYNQMVNNLNLDTFNCGMLWMAVGQLQILKTKFVNLKLNDFENGLDLKSRDDMQIERLRKYLTHYEIILKYCAIVQDILNITIFVQLGMSSIVICVGLCGFVAMPSNTETAIFMFSYLTTMTMQIFVPSWMGTQISFECGELMSAAYSCEWIPRSKLFKRSLILFVERAKTPVRITGLKIFTLSLDTFTSIMKTTYSFFTLIRQLQVDEVN

>BmorOR13

MAPKQIDCFEINWKFWKFLGIWSENKPHRYYKYYSKIFITFFVILYDVLYTINFYFVPRQLDLIIGEMLFYLTELSVLSKVFTFIIMRHKLKIIFEILESDAFQTDTEEELKILHRAKVFIKRYWKIVALVSITANLTHISSPLLKNLIFKVELVLPVCSYSFLSESFLKTFEYPLYFYQIVGIHFHMLYNLNIDTYFLGLMILIIAQLDILNVKFRNLKSGKDHTQLNESIMGLNKNLDHYNEIERFCSLVQNIFSFTLFVQFSMASCIICVCLFSFTLSVPVEYYIFLATYMFIMIIQIMVPCWFGSRIMDKSILLSSAIYNCDWTSNSKDFKINMRLFVERANKPLSITGGKMFSLSLATFTSIMNSAYSFFTLLRYIQTRE

>BmorOR14

MSNYIFKPFHETYRIITFTMIAAMIYPNPATEKRRLIYIGLMLLSVIPLAFMIVTEMYEFFMASDLNNTIRHSTVIGPFIGGFVKVALMYYKRRQANELVSEINRDHLAYNGLKGEDREIAASSIRNCQIYCELGWTLIVMSCGLSFPVIAILLKIHSFTFKLDSTKHMIHDINNPFTDDPEDRFESPFFEIMFVYTFFSSFIYIINYVGYDGFFGLCINHACLKMKLYCRALEDAMRSDSRRHEKIVAVIEEQRRTYEYIALIQDTFNIWLGLIYVATMIQMCTCMYHIVQSFNIDVRYIIFVISIIHIYLPCRYAANLKCMAAETPTLIYCCGWESVSDLRIKRMMPFMVARSQVIVEITAFNMFAFDMELFVWIMKTSYSMFTLMRS

>BmorOR16

MSFNSEDLYLNRAKFVMKYLGVWVPPENENFARKFYKIFMMSLQHLFLFFQIIYIVEVWGDLEAVSQASYLLFTQACLCFKITVFQINMNKLKELLKQMNGYVFQPKNINQQNIIKVQATRIKRLLFAFMISSQLTCGMWALKPLFDDVGSRKFPFDMWMPVSPERSPHYHLGYSFQLVTICMSAYMYFGVDSVAFSSVIFGCAQIGVIKDKIMSIKPLGIYRNHKTYTKISRYNRKTLIECVKHHQAVISFTELVEDTYNSYLLFQLVGSVGIICMSALRILVVDWRSVQFFSILCYLSVMISQLFVCCWCGHELSATSEELHTILYNCAWYDQDVKFKRDLNFMMARARRPILLRAGYYISLSRQSFVSILRMSYSYFAVLDQTNK

>BmorOR17

MREDKMEINNSQKFYTKMIFRYLYSVGLGDWWYQHEDRSDSHRKLYCLWAVISNAYIFLNICNELLANFRKDLTDVEKNDAIQFSFAHPLIFAKIASFFFNRKKIREVFGRLLEENRSVYSCGELEKESMKQIKRYSLAFIGVSYMTLVMSTIDGLRAHFKEGIPIRTEVTYYPSPSNSGVIVNILRFLVEFHWWYIVSVMVAIDSLAVASFVFVTFKFKLLQRYFKDMGLTVRRDQSNMTDEALADKFRRDFIVGVKLHENALWCAENVQKAFGWVYSVQVFETVALLVMCLVKLVTTNHNMIFLLANFAFMLCVIILNGSYMMPAGDVTYEASEVPTSIFLCGWELVRQTDLRFLVVVAIQRSQVPVIMKAFGIMTLSYSNFIAVSLFKFYVQFQINLF

>BmorOR18

MGDRMVTRGHFFDFNIKYLFYVGLWPSNEAKRIEKIAYKIYEYQLHVLSLIFLVTTGIGTYKNHKDIIALLTNLDKTLVAYNFVFKVIVFVYKREELRKLIEQIVQSGDQITEDRKALMAKLVIVLTGISTVIITAFSCLALFEGEMTIDAWMPFDPMKSKMNLFAASQILAATFVVPCGYRAFAMLGIVCSLILYLRDQLVDLQNKIRDLRFATGNVEKLRDDFKLIVKKHVRLLGYSKVIEMIFKEYFFIQNMAVTAELCLNAMMVSVVGLEQKTLAASFLAFLSVALLNAYIYCYLGNELIVQSEGIAMAAYESSWILWPVDMQKDLLIVITAAQKPMKLSAGGMAVLSVQTYSQTLYNGYSIFAVLNDIVN

>BmorOR20

MIQASKYPNSKTKELFRKIAHIAYICGLPNFWIEELNLPKSFIRVYDKIVRIFNVATYFFLGIEIAAHFTQHHLTNKQKFDLLLYSISHPILNGYGVIVSRQVGNVKKVLLDLIVNLKVKYNDPVIEEAMIKISMTYSVSFITNCVLSMLTYTFDALLMVYKKGVTFNVIITAWPDVEDTTTEASIGRIGFHIFWWLFVTRPFAVYVLVINLTTCLSHQYMNLQSYFFHLEDIFKENLSQNEKEAKYEAEYKIGVMLHANTLRCTRRCHMVWNGVMSGQIIFNISLIVIIMAQMMNSDRTLVNTFGTVLTASAILISTGFFMWNAGDVTVQASRLATAMYCSGWQNCRGKSSVSIRNMVMNTIAVAQRPLVLRGLGVIDLSYQSYLSIVKASYTVFSVIY

>BmorOR21

MNKNHYILKTYCDKIFLVGSGNFWYQKTESRNDKTLLYKIYSCVLFFTYGFMTVLEIMAAMMGDFPEDEKRDSVTFATSHTVVMIKFISIIKNKELLKTLNRKMMMICEAHEEQTLMDEMYRTVKINVVAYCVAVYGSATFYVFEGLRKFYNGSHFVTIVTYYPSNDDDTLAATIVRIATTLVLLMMLLTMIISVDTYTMAYLIMYKYKFITLRHYFKRLRENVDELVAAGKARLAAEKLAQGLVEGIKMHNELLSLSKDIDKAFGTVMALQLCQSSGSAVSLLLQIALSDQLTFTMGMKIFFFLAAMYLLLALFLCNAGEITYQVCTSIV

>BmorOR22

MNKNHYILKTYCDKIFLVGSGNFWHQKTESRNDKTLLYKIYSCVLFFTYGFMTVLEIMAATMGDFPEDEKRDSVTFATSHTVVMIKFISIIKNKELLKTLNRKMMMICEAHEEQTLMDEMYRTVKINVVAYCVAVYGSATFYVFEGLRKFYNGSHFVTIVTYYPSNDDDTMLASIVRIATTLVLLMMLLSMIISVDTYTMAYLIMYKYKFITLRHYFKRLRENVDELVAAGKARLAAEKLAQGLVEGIKMHNELLSLSKDIHKAFGTVMALQLCQSSGSAVSLLLQIALSDQLTFTMGMKIFFFLAAMYLLLALFLCNAGEITYQASLLSDEIFYCGWHKCNSPVLSTQRNIRDIVLIAILRAQSPLVMKAFKMVELTYATFILVVRSTYSVFALFYAQNK

>BmorOR23

MRAKTEFEKTIKLTKTALFLSGINIFLGEWNHWTRTFVDSIAYYLNIVGLYFVLIGEMYWLIDGTITGKSFVELSLIVPCLTISVLATAKVHYLYHNKESLLDVVDKLREIYPDEIEETANDNDQCLNDKKETVYDNDVTEVGIVNEANELLKFVNFLLSTVSFVVTMTFCTMPLFGMAGEFMETGKFVVLYPFAVKYPFDVYNTSFWVIVYVNQFWATIIVCTNIFGVDTLFYALCSYIGMNFRLLSYKFEHLEIKRNDRIINEIIVLIKRHQELIELVNKTQSLYSLSTLFNIVTSSLLICLSGFNITILSRSWSYFALLKTIYS

>BmorOR24

MPEELFLDRSIKKIESYFRWMGINIRSGDNNNKKDVFKIRCIYFINFVLLNTDVLGAIFWFRSGLEQGKTFTEVTYNAPCLTFSFLANFKMLSLIFYEKTVHELIAALQKLEIKHFLRQNCAEELKMLKDEKNFLHAVFKGSKIVNYASILTFGCSPLVLIASNYYKTGRMDYLLPLIVLYPFDVDNITVWPIIYVRQIWSVITAVIGVCATDYLFYTFCVYISTQFRLLGHSIERVVPNNGLSVRTRLNGNLRMKFVENLKWHQELIRAASLLEQIYTKSTLYNFVTSSVIICLTGFNVAVVEDFAVILSFLFFLFMSLLQIILLCFFGDKLMKSSTNISDAVYNSKWYLTEKNVGKVLLMVQIRSQRACRLTAYGFAEVNLRAFMKILSTAWSYFALLQSLYSSHE

>BmorOR25

MFEKALRSANFYMRVIGIPTDIRDGNRTLMERLRNRWFYCINFLWLNTDVAGEITWFVKGLLSGSSTLIENTYLIPCLTLCILGNVKTFFTIKYANHIIDLVAILKDLEIKNNAARKNETEIVKERLKFLTTSNKFLLFVIGTGIIAFGIGPLMLTASIYFSSGDMKLKLPFLIWYPFDSSDIRYWPFVYVHQVWSACIACCAVYGPDCFYFTSCTFIHIHFIHLQNDITNVIVESSRARRNGLYRGCHQAFLELTNRHKDLIRCVNLLEIIYSKSTLVNVVSSSLLICVTGFNVMAIDFLPLIAPFTSFLALGLVQTYLLCYYGDTIMCSSTEVSDAVYNSTWYGTNISQMRDYLFVMKRAQKPCKLTAYGFSDVNLRTFSRILSTAWSYFALLITIYRGNGQQ

>BmorOR26

SLSGSSVFTHLFLLRCCGFCRLSRSSTARRGLSVAHEVYRALTLTLTVVYLLQECVYAYQERTDMDKLSRVMFLLLCHITSVAKQLVFYLDADRIDYLIATLDDPSYNEMSHQRLLVDASRWASRFVWAYSGCAVVTCTLWIVFPIIYHVQGQTVEFPFWIQIDYTKSSMFVVVLLYSYYVTTLVGIANTTMDAFMATILGQCKTQFTILRIKFETLPTRAKQALRCDSEQNYDEVLMRLFHDCLKHYQKIVSAILIQFGIGAWILCMAAYKIVNLSVLSIEFASMILFISCILTELFLYCYYGNEVSTESERLVTSIYSMEWVGARLGFQRGLLVLLERARRPVRPAAGLVIPLSLQTFLKIIKSSYTFYAVLRQTK

>BmorOR27

MPSSFFLPNLENPDYPSLGPTLKGLKYWGMWQSGGIKRILYNSIHAFATFFVITQYVELWIIRNNVELALRNLSVTMLSTVCVVKAGTFVCWQKYWSGIIGFVSNLEKEQLSKNDAATQAAIVKYIKYSRRVTYFYWSLVTATVFTVILAPLVGFLSSPERELIANGTLPYPEIMSSWVPFDRSRGFGYWVTALVHTLICFYGGGVVANYDSNAVVLMSFFAGQMKLLSINCSRLFDDGNEVISNNEAMKRIKECHYHHVFSTIFNSLMSPVLFLYVIICSLMLCASAVQLTTDGTSNMQRIWISEYLMALIAQLFLYCWHSNQVLYMALEDRLGGLFEACLESGRFPSKWKTGRLVLLRKDGRPADSPAGYRPIVLLDEAGKMLERIVAARIVRHLTETAPDLSAE

>BmorOR29

MFDFLQNLEDSERPLLGPNFWLINKTGLLLPKTNFGKLAYILVHEIVTFFVVTQYVELYVIRSDLDLVLTNLKISMLSIVCIVKVNTFVFWQTSWREVLEYVNEADKFERNQTDETRGKMIETYTKYCRRLTYFYWSLVFTTFLTTTNTPLMRYWSSPIFRENLRNGTEDFPHIFSSWMPFDKNHSPGSYCTIVWHVLLCAYGAAIMAAYDTCIVVIMVFFGEKLNLLRERCKKMLANDLYNHAFVIGQLHDIHVQLIKQSRLFNSLLSPVMFLYILMCSLMLCASAYQLTSATSTAQKLLMAEYLIFGIAQLFVFCWHGNDVLFKNANVSLGPYESNWWSSSPRVRADVLLLCGQLRVRHVFTAGPFADLTLSTFIKILKGAYSYYTLLRK

>BmorOR30

MSVSNLKFEVLFKPTTMSLHMNRSHPSIKRNKIWLLQFISLMTLTAFCATGLITSLLFHDLKFGKYMEASKNGTIAMLSFTTTFKYSLLLYLQKSLNRLIAKIDMDYEIAKGLTPQEKVTVLNYAKKGVIVSKFWLFTAFAITFCFPLKAFIIMGYRFIIKDEFRLEPMFDMTYPEPIESYKTSFPVYFILFVVCFLFGCYASSLYVAFDPLVPIFVLHACGQLDLLSVRITKLFSDTKNPRIIAKELKVIIIKLQELYSFVNFIKVNFSILYEYNMKITTISMPLSAFQVVESLRRGEFNIEFTYFFFGCILHFFMPCYYSNLLMERSENFRFAIYSCGWENHNDKNIRQMLLFMLTRAAEPLGIATVFTNISLDTFAEVNTFDTVLLA

>BmorOR32

MTTRHAEPCAEAPRLSPAAGGMVGLAPVPQPSSNEMLVQERGRPPGLEGEYVANRPFRKSLRGSQEEPRANGKSENVRFLINSHILHCGLRFNETNCHTHYIAKVAIFCFIVTYMLQVMELYWSKGDQEKLFECFSILSFCGMGVMKLVILRVYHQRWRFLLNQVSILENRHLDPGPLSYDSDNDNDDNEIVTFITKYTDKFKRTSSILIKMYASTLVIYVLSPFVEYIFRQFRGDLNIAYPHILPAWTPLDEFSVTGYLIMVSFETVACIYCVFVHVAFDLTCVGLMIFACGQFYLLRYRSERIGGKGRICRLLKSTEVRAHYRIVFCHGIHVLLVQLIEELDRLIKHILGVYFFLATLTLCSVAVRLKTEDMSITQLVNLLQYMCGTLTQLFLYCKYGDSVYNEADEPYGLPDACLESGRFPKQWKTGRLVLLRKERRPADSPAGYRPIVLLDEAGKLLERVVAARIVQHLTGVGPDLSAEQFGFREGRSTIDAVMRVRALSDEAVGRGGVALAVSLDIANAFNTLSWSVIAGALQYHGVPAYLRRLIGSYLEDRSVVCTGHGGTVLRFPVQRGVPQGSVL

>BmorOR33

MELNFDKIFKIAIISQKFSGTYPYTKRDKKWATHFILMHGELTIICMLFIYNIIEFDLKAADYSQMCRNMCLSFVYLVITLLYINMLYYQSKLKMLIETMKAEYELAKTMSEEEQNVILEYAKKGRWLCRAWAILTTCGMAQFFLKSIVCTIYSAIQGNFRIVQYYEVICPEVIERHRNNPVIFITLYFCTFFYSLYTSALYTSVLPLGPIFLLHGCAKLEIVRLNIKNLFDNDDYVVQERLKKTVLQMQDIYCYSHEINECFQILYEFLLKATSLVLPITIFAVIQALGRGQFIPEFFAFIFGAFMVGTTPCYYSNMLMEK

>BmorOR34

MELNFDKIFRIAIISQKFSGTYPYTKRDKKWATHFILMHGELTIICMLFIYNIIEFDLKAANYSQMCRNMCLSFLYMVITLLYINMLYYQSKLKMLIETMKAEYEIAKTMSEEEQNVILEYAKKGRWLCRAWAILTTCGMAQFFLKSIVCTIYSAIQGNFRIVQYYEVICPEVIERHRNNPVIFITMYFCTFFYSLYTSALYTSVLPLGPIFLLHGCAKLEIVRLNIKNLFDNDDYVVQERLKKTVLQMQDIYW

>BmorOR35

MVVLSLGISSEIGTLKFFYTFIYIKKVQRIVREYLECDHMVVPGSRFADNVLKTMRNVKKRAILYWVVVIGNGVVYVTKPLFMSGRHHMEDRYIVYGLEPMFESPNYEVAYFLMMFGLCFICYPPANVTVFLIVVVGYTEAQMIALGEEMLRIWEDAVAHYNNKYHTVGALTNSSEKNKIINQYVKFRLTEIIKMHTTNIQLLRQVEFVFRSAIAMGYVFLVLGLIAELLGGLENTYLQIPFALIQVLVDCYTGQKVMDASSLFEQAVYDCKWENFDKSNMKTVLLILQNSQKSMRLSVGGITVLGFSCMMSVMKSIYSAYATLRTTMS

>BmorOR37

MELGCSRHLKLPCSLHPIGISKHGNTLSELLIYFPAIPKITYAILAVLLTVYYYIYLCSITWFVFVRCPQTGDLAAASIVFSLGVSSEIGAIKLFIMYVYRAKLRDITGEYLQCEADMAPGRLRARVGRSLRTVRRRAFVYWLVLVVNAFAYDLMPAFLPGRHLSEDVFVIYGFEPMFESPNFEIASTLMGVSVVFICYTAGSISAFLIVIVGYSEATMLALSDEISCVWDDACASECQQPNDFIRARLGKIVAIHTKQIRLIREVEVVFRGALAGGFACVAFGLIAALLGGLENTFLQLPFCVIQISVDCFVGQRLRDANVAFETAVYNCKWEYFDKSNMKTVLLILQNSQKTMGLTAGGVAALDFTSLMTIFKSVY

>BmorOR38

MVVFSLGISSEIGSTKFFNTIIYIKELRKLFKDYLLYDATCPAQGRLRLHLLTTLRYVKRRAIIYWLVIIGNGFIFAIKPLLVEGRHLAQDDLVLIGLEPMRQSPNYEIAYAIMTMGVCFICYPPAHVTMFLIIIVGYTEAQMLALSEELKHLWNDAIEHYEKHSRTEREADAAMKSKILNSFVNFRLVQIIKSHSTNVNLIGRVENVFRGSLAVGYVFLIVGLIAELLGGLENTYLQVPFALIQVAIDCFIGQRVNDANIDFEKAVYDCKWENFDKRNMKIVLLLLQNAQKTVSLSAGGIAKLNFSCFMSVIKSIYSAYTTLRTTMK

>BmorOR39

MLWSVFSYFTRADDVLAGIVIFSLGVSSEIGLVKLCFMYANIDKIQKITEGYLKSDAASARNSRFSKNILHTMQSVKKRGVIFWLVIISNGVVYLVKPIVTPGRHFMEDQFIILGLEPKYETPNYEIGFFMMAVGVCVTCYLPANITAYLITVAGYSEAQFLALGHELANLWPDAQLHCRAMNLSQSVNEQANEYVKMRLRELVKIHSTNVNLLRDIEGAFRGAIAVEFLLLIVGLIAELLGGLENTYMQVPFALIQVSVDCLTGQRVMDANLALERAVYDCRWEEFDASNRRVVLLLLQNAQKVATLSAGGIATLNFSCLMAVIKSIYSAYTTLRTTMK

>BmorOR40

MTGAGAGTFRTGAGPGRGDGVARRGESGETTTLGRDAFAALGCFGAADGSTARARFFPRVTVLNPSEVPGSGLAADSNSISDSESEPELDAAQDAIDAGAGVGGDIGESRARTVFGIQGHDASDSALRMHNNVAIYAKTTMSGNSQLTFATAATIFLKNASGPNGVAIGTDYAICVVSLSLFFCYRFTELVEDTYNSYLLFQLVGSVGIICMSALRILVVDWRSVQFFSILCYLSVMISQLFVCCWCGHELSATSEELHTILYNCAWYDKDVKFKRDLIFMMARARRPILLRAGYYIGLSRQSFVSVSIPRIRFNAILVI

>BmorOR42

MDIPKFEELLKQIKMNFWLMGIPFDNPKIQIRYYVLLLPLSLMLIEEIAFFGSRMSSENFLELTQLAPCICIGVLSVLKILALTAKRQKIYELTQNLECLHKIILNDTRKTELVRKNLVLIKFITKYFFVLNAVLIFVYNFSSPVIIAYNYIVSNEVQFVLPYAVLLPFKTDSWIPWLIVYVYSIFCGFTCVLYYATVDVLYCVMTSLVCNNFSLISFKLQKVNRNTAHLLKEVVKEQQYVLKLAEDLENIFTAPNLFNVLIGSVEICALGFNLMIGDLTQIPGCILFLSSVLLQILIMSVFGENLISESSRIAEAAFLCKWYEMDQKSKKTILTIMIRSHKPKKLTAYKFSVISYGSFSKIISTSWSYFTILRTMYTPPGTKFQDDL

>BmorOR44

MYTYFKVLVFWLNKDKVISLQKILHCKEFKPKEPEHKEIIRKSIRKARFVMTSYATMCVGAVSVGIILPLTENFDILPTNVEYPFFDVYKNPTYAYLYLHHIYYKPATCIIDGVMDTILAAFVASAIGQIEILAFNLRNFDVLAERRRKRAISGNKYIGKYTNLYFTKRILKECILLHNSIIRYVSVIESAFSLASALQFMLSVMVLCLIGIQFLSIENPTSHPMQMVWMAIYLTCMLIEVFILCWFGNELIWKSNDLRQAAFDGPWRNLNRKTCMFIIIFMERCKRPMRLSAGKIFTLSLDTYTVLINWAYKAFAVMRNMKK

>BmorOR45

MKVLDNVNHAVKVTMNCCRLYGLFVSDDLTKRQLIIMRAFSLMLYLFFVGFFITTQSALIITMWGDLNLMTNVGLVLGTHLTLSAKVFTLHYKEKEITNVIYKNEVRLRAETREQGKYIISEYPCNTTKSPAHEIILAHQGIAVILTATLEIAIVLLMTSIVAVCRCRLKLVGLSFETICDDLPSNIMNKLTADEQVIVAKRVRENVIEHQAVLECINDIQDCFSSAMLVHIAISTMIICATAYQLAVEKSLDLTQRMTMASFLGGMSTEIFLFCYQGGHLSIDSMEVATAVYSCPWYTFPTSLKRSLLVIMIRAQQPALLTAGGFAPLLLDTFVSIMKASYSFFTVLQNASE

>BmorOR46

MAFFIRNKMLGLTITLNTLSWAGLIMRDQYTKTQRIIVRVYGWLVFLYLFVAATYVQIADLIDIWGDLDLMAETSLLLFMELAVISKILTLIFKYDKIMEIINGTEDILCSENRLEGQKIIASIDKETTRFFQYYTSSVIFTTFFWFLGEHSSTFFIRAKYPFNELKSPGYEFALIHQCMMMVFTGYFEFNINIFFASVVAGCRCRLKLVALSLRNICINIPVNKKNLITPEEEKLITERLHCAISQHKYALDAAEDVKHCLSKVLLVQLTVSIVIICTTAYQMAVNKSTDTIQKLSMAGYLLGASFEVFLFCFQGQSLSNASEDIADAVYECPWYTLTQPLKRTLLIIMMRAQSPAILTAGGFVTLDITEYMAVLTGHGGFGDFLHRTGAEPMAECHHCGCDLDTVQHTLLVCPAWKGWRRDLVVKIGNDLSSVLWHRCSAATSRGRRCLTSASAPSRRRRRGA

>BmorOR47

MKLVFDNFISALKVTLNWSRYIGIFIPDELTGRRQKLLVQAYSVFMYYLFIGFFITTQIISFILVWGDLNLMTDVGLVLGTNLALSAKIAVFFFKREDLANILKKNDDTLRSETRAEGKKIISETSCESARVGTTTLPISAVVTILETLELISQASLLITVDIIMLSMIAVCRCRVKLVGLSLQTICDDLPCNVKNKLTSDEEVIVAKRIREYVIEHQAILDCISELQNHFSPALLVQLLTSVVIICVTAYQLAVEKSSDLLRKFTMASFLFAMSTEMFTFGYQGGHLSHDSMEVATAAYSCPWYTFPTSLKRSLLVIMIRAQQPALLTAGGFTTLSLETFVTIMKASYSFFTVLQEATD

>BmorOR53

MALKKMLALTKGLEDPTHPLLGPTLKALSVFGLWQTGSQKSTVIYNTFHFLTFLFVITEYIDLYTVRKELSKMLNNLSVTVLSTICMIKTLSYVCRQSHLKVLVREISELELELMKTTDKNIVKRLRQYTVYTRAVTYVYWFLVVGINVVLLTSPLLKYASSEIYRSEIKNGTEPPPLILCSWFPFDSARMPGYFWATMVHIIMSIQGCGVVATYDMNAVAVMSYLKGQTSILKDKCKAIFDETASSRDVLNRIRDCHRHHNILLRHYYMFNSLLSPIMFVYMLICSFTICCSIIQLDSSETTISQRIWIIQYSIGQISQLFLYCWHSNEFAAKVKKKHFPLFPINLF

>BmorOR54

MGLNTIKEFFVNVKRRFQDVSIDSLLWIVNIVPSLAGFSIRSDRVSAPFWIVHWSLLVYVYAVGNAVYQWKFANEAIDYITSFINVSLLILIGNNSWWFLANRRLLKSVLHKIEVNDELSRRSEQSRLKHKKLLKIIKRIVLVFYMSNYVNASFIYLPNRVDVLNNYAMTPCVGMEPLTVSPNRELCLTILCMQEFSIMTVVLNFQALLLCFIAHTAVMFQILADEIMALNNYENLEEHQAYVKEMLPIFVKRHSLTLSAVDNYKSLYSVPLGVNFGSNALTILLILYLPVLEWFKFIPIFVFCFMLFFLYCFLCQKLVNASEAFETAIYCCGWENFALREMKMIYVMLHQAQKPVELLAADIVPVNMNTFATTLQAMYKFVTVVKF

>BmorOR55

MCFLKIKQQIIDIQKHFKDYSLNGSLWIVNLLPRLMGFNLRADKVGVFFWTIYILLLVYVFGIGIFVYLWKHVDTMSGLMKSYLNLSLILVIVNNSCWFLSKRSLLNKVLKKIHLIEDLSCESEHALAKYRRVFKIVTHLLLASYVLFYFTEIYFMFLFRNYDLLEDYSLAPCVGLEPLSSSPNSEICLIIVLIHEFISTTVMMSFAALFLVLIAHTAVMFLVLAEDMTKLTDLINLADHRKMIRESLRSLIHRHSLLLQIVYELRLLYSVPLGINFISNAMSILVLLCLPIHEWPSFLHIIGYCFFAFFLYCFLGQNVINASEKFIDAIYCCGWEHFGVAEKKLVHVMLRQAQKPVEIIALGMISVNMNTYVEALQLIYKFVTVLKI

>BmorOR56

MKLLEKLEDPDRPLLGPNVKALKFWGLLLPESRSKKYFYLFMHFAVTVFTATEYIDVWFVKSDLALLLNNLKITMLATVSVLKVTTFLLWQNAWRDLIGYVSRADLEQRATSDSRKLALINGFTGYCRKITYYYWFLMYTTVAIVTVQPIFKFFSSAAYRLDVQSGNGTYLQVVSSWIPWDKNTLPGYLLASIYQTYAAIYGGGWITSFDTNAIVIMVFFRAELELLRIDCAALFDDEKSFGDMAFMRRLKECHRRHTELVKHSRLFDSCLSPIMLLYMFVCSVMLCVTAYQITIETNPMERFLMTEYLVFGVAQLFMYCWHSNDVLYASQDLSRGPYESAWWSRDVKYRKNLYILVAQFNKVIVFSAGPFTKLTVATFIRILKGAYSYYTLLSQSQMNKT

>BmorOR57

MPSLIKNRIFGLTLTLNTLSWAGLILRDDYTKTQRIIMKVYGGLVFLYLFVFTAYVQIADLVVIWGNIDFMTETSLILFMQLAVSAKVLTLMLKSKKIMEVTNEADAILISEKKVEGQRIIASIDKNTTLFLKYYGFFVAFTIICWFMGENTSTFFIRSKYPFNELKSPGREFAFVHQCIVVIFTGSFDFNVDIIIISLVAVCRCRLKLVALSLRNLCLDIPMNKRNLITSDEEKVITERLRNIISQHKRALDAAEAIKHYLSGALLVQLMVSIVVICTTAYQLAVKKSTTMQSLTMAGYLFGTSLEVFLFCYQGEFLRESSEEIADAAYECPWYTLTRPLKKTLLIIMTRAQRPATLTAGGFVTLDITEYMAVSLISNT

>BmorOR58

MKLVFDNFIFALKVTLNWCRYFGIFIPDELTGRRQKLLVQAYSVFMFMLFIGFFIITQIILFILVWGDLSLMTDVGLVLGTNLALSAKIAVFFFKREELASILKKNDDTLRFETREEGKKIISEYPCDTKRSPAYEIIMIHQTIAVAVIASLAITADLLMLSMIAVCRCRVKLVGLYLQTICDDLPCNVKNKLTSDEEVIVAKRIREYVIEHQAVLDCISELQNHFSPALLVQLLTSVVIICVTAYQLAVEKSSDMLRKFTMASFLFGMSTEMFMFGYQGGHLSHDSMEVATAAYSCPWYTFPTSLKRSLLVIMIRAQQPALLTAGGFTTLSLETFVTVS

>BmorOR59

MDTNPSAAGDSVAPHLRRLRQVGFCQLDPTSQSRRPILALMHRVYHRLVLAATVLYIFEQLTYAYQARNDMERLSRVLFLMLCHLTCIAKQFVFHSDADKINQLVVGLDDALCNQPVETHRLLLLETSRRAARLLMLYSGCAVSTCILWAVFPLLDQLRGRTVEFAFWIPIDYRHNAFQFAVVLAYAFYSTSLVAVANTTMDAFIATVLYQCTTQLRILRMNFESLPERAYALSRKTRQDYHTVTHELLVDCLLHYKKITETCNLLEQIFGKAILVQFGVGGWILCMAAYQIVDMEILSIEFASTALFMGCILTELFLYCYYGNEVTVQSGLVSESVYAMSWLSLCPRERRALVVVLERARRPLRPAAGRVVPLTLNTYLKILKSSYSFYAVLRQTK

>BmorOR60

MVRPCRYFAIHFILLRFLGLGWWHHPHENETRNYPGLYLYYSILTQLVWVVGLVGLETIDPFVGEKDMDRFMFSLSFVITHDLTLIKLYIFYFRNVEIQDIVRTIEIDLYRYYQNDDKIRATIRISRIFTAAFLFFGWVTIGNANIYGIVQDLRWKDIVKNLNETTSKPLRTLPQPIFIPWPYQEDKHYILTFILETMGLLWTGHIVMTIDTFIASVILHMSTQFAILREAIVTAYDRTMIALSEGALQSGVLCENSNGNEENNQIFLESFYSKEHIESVLESTLLSCIRQHQLLIGCVEKFSKTYSYGFMTQLLSSMAGICVVMVQVSQGASSFKSVRLVTSLAFFFAMVIQLAIQCFTGNELTIQAERIADAVMESKWEKMPVRLRRLLLVTMMRAQRPLHLTAAGFAYIDNTCFLSILKAAYSYYAVLSQKQG

>BmorOR61

MARITDVFRLNFIFWKFLGIWGKSAPSKYNMAYTALYLSASLFVYDIFLTLNLIHTPRKLETLLRETMFYFNHLVAMTKILKMFIRRKKILVIFDLLDCEEFKPSDEDSQEIMKRKNEFYYIYWRIVAVTSNLSCFMQVVGPLIKMLIWKSELGLPVCKYYFMSDEFRNKYFVIWYIYQSFGIYNQMVNNLNLDTFNCGMLWMAVGQLQILKTKFVNFKLNDIENSLDLKTRDDMQTERLRKYLTHYEIILKYCATVQDILNITIFVQLGMSSIVICVGLCGFVAMPSNTETAIFMSSYLITMTMQIFVPSWMGTQISFECGELMSAAYCCEWIPRSKLFKRSLILFVERAKTPVRITGLKIFTLSLDTFTSIMKTTYSFFTLIRQLQVDEVN

>BmorOR63

MKLWIRNANFTISLSLTLLRCLGFWSPDGLAGNKRLLYNCYSFVFFMFLLGIYILIQVVDMIKIWGDLPLMTGTAFLLFTNFAHATKVINIVIRKNRIQRVIQQANAVLMGVQSEEARRIVKSCDFETSIQLCLYFLLTFVTTVGWATSAEKHQLPLRAWYPYDTSKSPAYELTYIHQVAALLIAAYINVAKDSLVSSLIAQCRCRLRLVGLALASLGQDLKIDYQSQLSPAQENILNLRLKTCVLEHQTVLAAVTELQACFSKPTFAQFTVSLIIICVTAFQLVSQTGNLVRLLSMGTYLMNMIFQVFIYCYQGNKLSVESSEIAGSVYFSPWYLGSVKLRRALLIVMVRSRRVAKLTAGGFTTLSLASFMAIIKASYSLFTLLQQVKQKK

>BmorOR64

MGVSNGRGTVKPFLYPLVDELDYNLIVGVHLPFEYKTPSRYPLAYITVVIAFIYVSYFVMVTDLIMQAHLLHLLCQFNVLADCFENMLNDCVKGFEGPLVSLHEYIHPLIDEFEYNLMVGLRLPFSFDTPLRYLFTYVIVLIAFNYTAHYVMVTDLIMQSYLIPLICQYAVLADCFENILIDCSNDYGDHARRNDIVYSRSMELRAILSRPMLGQLASSGLLICFVGYQATTSISVNIVKCLMSLFYLGYNMFTLFVVCRWCEEITNKSLNIGNAVYCSGWESGMTVVPTVRSTILLVILRANKPIVFTAGGMYNLSLTSYTSLVKGSYSALTFLLRIQHE

>HvirOR1

WPLFSTCSYIVMGPTSLKRNMFFWIPVKKNKVDVAKPKVKNITTFQDALRATLIIGQVFSLLPFVGVFTNVASNVKFIKTSWKCGYSLLSLIGQMFMAVLCVNKLAKSNVSLNGTSPVIFYVTTCVTMMLFFQVARRWPALVQHISKAEDMDPNFDCSLTRKCNITCAVVLILALLEHILSLLSAFAGASACYTGMDTYQGFVTHFYPWVFNYLPYSIVLGVITQFLHFQSTFIWNFSDLFVICMSYYLTSRLEQVNRKLLAAQGKYLPEIFWRATREDYCRVTQIVRKVDEVISGVVFISFANNLFFICLQLFNTLEDGLKGTGECTQLNSQSKLKKIVVSKSGPLGGHEAAAYFLFSLVYLLSRSVAVSLIASQVNSASSVPAPVLYDVPSPVYCVEVQRFLDQVNGDKVALSGLQFFSVTRGLLLTVAGTIVTYELVMFQFNSSTPSLNITSPTSATHIITTLAT

>HvirORco

MMTKVKAQGLVSDLMPNIKLMQMAGHFLFNYHSENAGMSNLLRKIYASTHAILIFIHYACMGINMAKYSDEVNELTANTITVLFFAHTIIKLAFFALNSKSFYRTLAVWNQSNSHPLFTESDARYHQIALTKMRRLLYFICGMTVLSVISWVTLTFFGESVRMITNKETNETLTEVVPRLPLKAWYPFNAMSGTMYIVAFAFQVYWLLFSMAIANLMDVMFCSWLIFACEQLQHLKAIMKPLMELSASLDTYRPNTAELFRASSTEKEKIPDTVDMDIRGIYSTQQDFGMTLRGAGGRLQNFGQQNPNPNGLTPKQEMLARSAIKYWVERHKHVVRLVASIGDTYGTALLFHMLVSTITLTLLAYQATKINGINVYAFSTIGYLSYTLGQVFHFCIFGNRLIEESSSVMEAAYSCQWYDGSEEAKTFVQIVCQQCQKAMSISGAKFFTVSLDLFASVLGAVVTYFMVLVQLK

>HvirOR3

LIDDGFFSFNLKYLYFVGLWPEKTLTANQKILYKLYEYFITFLTTTFIVLASIGTYQHKHDLVVVFCNVDKCLVVYNFFLKTIIFFIKRDKLRDLIDEIEMSGDKVTEERKKLMANYVMFITGMTAAVIGAFSLLALLEGTMSVEAWLPFDPMESLMNQILALEVLAFCVFPGLCRAFAMQGLVCSMIMYLCDQLTHLQTELRDLTYVKESEMAMRLKFKNAIRKHIRLMGYSGRMESIFKEYFLVQNLAVTVELCLNAVMVTVVGVHQITLLVSFVAYLLLALVNAYVYCYLGNELIIQSQGIALAAYESTWTSWPVDLQKDLLIVILAAQRPLKLSAGGMALLCIQTFSQALYNGYSIFAVLNDAVN

>HvirOR4

EVTAAPVPSESGSRPSRPTHCVVGGAHAFILRISSFFGLAPLRFESRSNGFTVSISGAMCVYSYILVTVLVICTLFGLVAEINVGVELSVRMSSRMSQVVSTCDVLVVVATAGAGVYGAPRRMRNMLKFMENIASVDTSIGGQYSRVTERKLCGIILAILIFSVLIADDFTFYALQAKKLDREWDVVTNYLGFYLLWFVVLILELQFAFTALSVRARFSAVNDALALTARQVSIPVEKPKTSSPLNIYAIRVAPVDSQRSANVSLLVDTMTGREHVVIIKRTASGEPRLIVSPCDAVRRLAALHGTLCDVVNSIDDSYGLPLVVILISTLLHLIVTPYFLIMEIIVSTNRIHFLVLQFLWCVTHMLRMIVVVEPGHYTIAEGKRTEGLVCRLMTSAPSTGVLPSRLEIFSRQLMLQSVSYAPMGMCTLHRPLIASVIGAVTTYLVILIQFQRYDN

>HvirOR5

AYLDKVFLWSCLYGVFGSKRFISLIWSTLILGSLVIIEVLAIWKVIRALAGVARDMSGHRSVTARLAGTIFYSISILSLVLVSKLYYNWRTNIAGVWGKVERSVGVKIPVDRTLKCRMTFVAGLMTFFSIFEHAMSILSSVGLDCPPSLILKRYVLVSHGFIFMGQDYSEWFAMPLVIISTIATLLWNFQDQVIVLISMGLTSRYRRLNECLAKVCELEKQHKDSDKKIEAVKVYTWRKIREAYVKQAMLVRKIDVALGGIVILSCSCNFYFICLQMFLGITQGLSSDLLSLIYYVISLAWLCTRVISVVLAASSVNTHSKLALNHLYNYETHCYNVEVERLQDQLTKDYIALSGMGFFYLNKTILLQMAGAIVTYELVLIQFDDQGNDALNATKI

>HvirOR6

MNLRKFLFENEAVEGINSPADYLYTRILRFNLDFIRTWPRKELGEPENLAFTVFMQYFYLILNIVTVMGSTSYIVVRGSELSFIEAGLMYLIFLIGIVDTLTVVCLTFSEKFRVLAKDFLTKTHLFYYKDRSKHAMEIHKKIHLISHLFSLWILFQMLSGLSLFNIIPMYSNLAAGKYRKGGLQNSTFEHSLYYLYPFNTSTDITGYIIACILHWIISYLCSCWFCIINLFLSLLVFNLWGHFKILISTLNEFPRPSSKSVDTQESPYKYTEEELIEVAEKLKDCINYHREIKIFTNRMSDVFGPMLFIYYAFHQASGCLLLLECSQMTARALMRYLPLTIIMLQQLIQLSVIFELVGTESEKLKDAVYGVPWDCMDTKNRKVVMFFLMNVQEPVHVKAMGLANVGVTTMASILKTSLSYFTFLLSQTKEE

>HvirOR7

IEKLGVLFRFSGMNIKNKIVTPLDTIKYRWLYTLNFLVVFSAIIGSVYYVILGIKQGKNFIEVTSVAPCLTFSILSMIKSLYHLMYEEHIQELIELLRELELRENNREKCIEKEEIIASETGFLNKVINVLYVLNCSMIVVFDMTPMIMIAVKYYKTNEFEMLLPYLDVFSFIPYELKYWPFAYIHQIWSECVVLLDMAAADYLFFTCCTYIRVQFKLLQYDFERMIPDRGISKGKFYEENELRNKFTELLKWHQDIIYSSTILEIIYSKSTLFNFLSSSLVICLTGFNVTIVDDIVIIITFLTFLSMALMQVFFLCFFADLMMTASLEISNAVYNCRWYSANIKVGKQILFVQTRAQEPCKLTAAGFADVNLNAFMRVLSSAWSYFALLQTVYGGK

>HvirOR8

CTRVIIFYKFKYVDFSNLLDSYCKIHNSFAIKIFFIMVFRQIDCFKINMKFLKFLAIWPGKDFTRRYKYYTVAFLTAYFIIFMILFTINLFFLPKQLDIFIENMVFYFTDSATLSKVMTIGFMRKKILQLFEMLESDIFQPDNAEGLAVIEKAKKFNKLYWNIILAVSFASCASNLFPPIIAHFILGTELVLPICNYGFLSEDFRQMFGVPLYLYQGSAMMFDMLYSVNIDTLFAGLMVLAIAQLDILGIKLRRVTDKEVLEETDSETSRQHRDNHKEAIKKINHCIIHYEKIHKYCSLVEDVFSITLFVQFGMASCIICICLMRFTMPAPLVYYLFLATYMFVMILQILVPCWFGQRIIDKSNLLAFSAYDCEWTSETRQFKSSMRIFIERAHKPLSITGGKMFCLSLVTFTSIMNTAYSFFTLLQNVKSRK

>HvirOR9

MVDQFQKCLKSVNLYLKFIGLHLESKDTTKTFIERSRSHRLYFAHFFSLNLEVVAQILWVLEAVITRKSFVEITRLIPCLILTLISDFKTLSLLYYARHNNEFIVTMKSLLLNQKQLEEKETRFREDLIDKHVLMLTSITKKISYLIGMGLLMFALAPAFIIIPHYFKTNEVKLEMPFIAYYPFNEFDSRIYPWVYLHQVWTACVAMIMVYGPDCFFFTCCTFIHIQFSLLNNDMERIVNEDTPRYDKTKFKELAVRHIELMRCVNLLEKIFSKSILFNALTSSVIICVTGFNVLVVDNIVMMASFTAFLLFGLMQIFLYCYYGDTIMRSSMQVSTSIYNSPWYNIRAADRKGFFIVIIRAQKPCELTANGFFKINLSAFTSILSTSWSYFALLKTMYHPE

>HvirOR10

IDAVSEASYLLFTQASLCYKSTAFMVNKQSLLELLEIMDCEIFEPKSAEHEKILAAQARKIKRLCLFFLTSATTTCTLWAMIPLFDAASKRSFPFRIWMPVTPLKSPDYELGYLYQMVSIYISAFLFISVDSVAVSMIMFGCAQLEIIMDKIQKIKYVFESADSEEKRREIIKINNEFLVECIKQHQTVERFIQLCEDTYHANIFFQLTGTVAIICNIGLRISIVEPNSVQFFSMLNYMVTMLSQLFLYCWCGHELTIRSENLREWLYQCPWYEQDTKFKRALFIAMERMKKPIIFKAGHYISLSRPTFVAILRCSYSYFAVLNRVNTE

>HvirOR11

MHLAGNAVTGITGPMDYKYMKVLRFVLRIISGWPGKALGEKTLRIEGMGHAYYNTILSLVYLALGIAYLKKNFHRFDFLELGQLYIVLLMNMLSTSRAFTLCLSQKYREVAKIFIQKIHLFYFKEKSDYAMKIHVIVHKISFISAVYLSVLLFIAAVMFNLIPMYNNYSAGRYSSFDNLENTTYEQAISCLYPWNFETNFNGYLVATLSGWYGTMLCGSSVSMFDLFLCLMIFNLWGHFKILIHNLEHFPRPASEIVDAEGAERSGRIIGSEMYSQAELEQVAVLLRECIQYHMLIFDFTNNMSDAFGMALFIYYSFHQITGCLLLLECSQMTAAALTRYLPLTIIMFGELVLLSIIFETIGTMSEKLKDAVYKVPWEYMDTKNRRTLLIFLIKVQEPIHVKAGGLVDVGVTTMASILKTSFSYFAFLRTF

>HvirOR12

MMEEEPLLIDKTVKKVEFLFRWTGINIKSGTKTRMDTIKSRAVYIINFIWLNSDLAGAVVWFFAGIANSIGFTELTYVAPCITLSFLGDLKSLYLIIREKNVDNLIQMLRDLEINERARPKSEEKDAIIKYEHNFVTTVISVLNVFYFVLLVAFALSPVTLVALKYYTTNELELLLPFLIVYPFNPYDIRYWPWVYLRQIWSEVVVVIDICTADYLFYTFCTYIRMQFRLLKHCIERVIPEDDGSGRLLNIEQVRAEFVQLIKWHQDLISSANMLETVYTRSTLFNFVSSSVLICLTGFNVVAISDVAFVVTFLSFLFMSLLQIFFLCFFGDLLMTASTEVSDAVYNCRWYLADTSFGKDLLLVQTRAQTPCKLTASDFSDVNLKAFMKILSTAWSYFALLQTLYGAPT

>HvirOR13

MKILSDGSDLEGVEKVEDIFYINLARKSMWILDSWPKAFNASSKYRYFVLALNVATLIGGAIYLRNNTGVLSSFELGHTYITVFMNCITCSRCLMILSKDYNHVMTLFVQKIHLFHHKHKSDYAYLTHIFIHKISHFYTVYLLGLALNGLFLFNMIPFYNCYSRGMFRDVIPANATYDHAVFYSVPFDYTTKFKGYLAMTSFNVFISYTCTSYFCVVDLTISLVIFHLWGHMRLLTYHLANFKKPASVLESNDNNKDEIKDHSYTEEELKEVFSKLREYIQHHNLILEFSSEMSNAFGPALLAYMVFHQVSGCILLLECSQLDTKTLVRYGPLTIVIFQQLIQISVIFELLGSSNDKLIDGVYLVPWEYMDTKNRKLVFTMLRQSHRSINLTMMSMVTVGVQTMTAILKTSFSYFVMLKTVAEEE

>HvirOR14

MTGIRDFFFNYEAKDGVTNPTEYPYMIMSRHLLTVITCWPKKPKEGLNARAKLRAKIWVIVQKIFHMSLCLLTTLGMAMYIGLHKKSMSFLELGHLYISLLMTVVIFSRITTLCLNPKYRAVSTEFLTKIHLFYYKDDSEFSMQIHKQVHKISHLFTLYLTGQMIAGLSLFNLTPMYNNFSAGKYKKGGLKNSTFEHSLYYSYPFNASSDVGGYIVSNICDWIISYLCSTWFCTLDLFLSIMVFHVWGHFKILLHDLDHFPRPANLTTFKLDNSNITLTSEKFSSIELGQVSEKLKKCIEYHRKIVSFTDEMSEVFGPMLFVYYGFHQTSGCLLLLECSQMTVAALVCYLPLTIMLFQQLIQLSIIFELVGSVSDKLKDAVYSLPWEAMDIKNKKTVAIFLMNVQEPVHVKALGLAEVGVTSMTAILKTSMSYFTFLRSK

>HvirOR15

MTGFRDFVFNYQPKDGITNPVDYPYLIIARYLLTFISMWPKKSVVYHSARAELKARIWLWVQKFYHLLLCAVAFFGGVLYITLHKKSMTFYELGHLYISLLMMACTFSRITTLCFNDEYRVVAKDFVTKIHLFFYKNRSDYSMQIHKKVHMISHVFTLYLSGQMMLGLFLFNVTPMYNNYSAGKYKSGGLKNSTYEHSLYFSWPFNASTDMRGYIVSNILNWMLSYTCSSWFCVIDFFLSLMVFHIWGHFKILLHDLDHFPRPLNKVNSVIEDSITITNEMYSQTELDQVFDRLGKCIDYHREIVSFTDKMSEVFGPMLFAYYGFHQASGCLLLLECSQMTVAALVRYLPLTIILFQQLIQMSIIFELVGSVTDKLRDAVYGLPWEAMDTKNRKTVAFFLMNVQEPVHVKALGLAEVGVTSMTAILKTSMSYFAFLRSM

>HvirOR16

MGLRQFLFENEAVEGINTASDYLYIKILRFTLVIVNSWPRKEIGEPESPRLSAFAKYFYLFLTVLAAIGSIAYVAVHNRELTFLETGHMYIVVLMSLVDVSRVATLTMSTTYREVARDFLTKIHLFYYKDRSKHAMETHRAVHKISHLFTLWLVGQMLSGLSLFNLIPMYSNYAAGRYSGDVSKNSTFEHSLYYSYPFDTSTDIRGYSIACVIHWVLSYLCSTWFCMFDLFLSLMVFHLWGHFKILINTLNDFPRPSSKVEGAQFSDEELVDVAARLKDCIVYHREITLFTDRMSNVFGPMLFVYYSFHQASGCLLLLECSQMTAQALMRYVPLTIILTQQLIQLSVIFELVGSESDKLKHAVYGLPWECMDVKNRRVVLIFLANTQEPVHVKAMGVANVGVTSMAAILKTSMSYFTFLRSM

>HvirOR17

MSLRSECARSVAPHVRVLRRVGFLRGAALPARPRAVRLALRGYHALALAATSTYVLQQAVYAYQERGDMEKLSQVMFLMLCHVTCVVKQIAFHVDADRIDRLIAGLDEPLLNQCEGERGALLRGTARGAARLLRTYVGCAVATCVLWIVFPILNRIQGISFEFPFWTGFSYDHNVVFSLVLLQSFYCTNLVAIGNTSMDAFMATILDQCKTQLRILRINFESLPERASALHMETGENYDTILDELFVDCLVHYNKITEMCAELHDVFTAPLLVQFAVGGWILCMAAYKIVSLEVLSIEFASITLFITCILIELFIFCYYGNEVTVESERVSQSLYSMEWRRARLAFRRSLVLVMERAKRPLRPAAGRVIPLSLDTFVKILKSSYSFYAVLRQTK

>HvirOR18

MEMKVDVLPEKKYKGFNETFKLCAFSLAFAFLYPNRTTALRRCITITLIVTFCGGQLFWFITYTFKCLYTLDIYNFARNMTLAVVLVLFFIKTYYVIYATSKFAPLLDKISEDLLEANNLEEEFQVLYDDHIKIAKVGEISWLLIPTIMSALFPIYAGALMTIESIQTDDYERRMVHDMELLFVEDIQSETPFFQCMFAYNCVQCVVLVPNYCGFDGSFCIATTHLRLKLKLMTLKVYKAFKYSKSRQELRVRLYESIKDHQDALDFYVQLQNVYGPWLFAVFLLTSFMISFNLYQIYLLQRIDPKYTSFGVVGVLHIYLPCRYASDLTRVSEEIPDDLYLAEWEAWADPSITKMLMFMITRAQKEMIVTGMGLVVFNMEMFKSILQTSYSFFTLITA

>HvirOR19

MKDRDILFKYCKVMFYIGSGNCWYKEDEIGNDRSILYRVCSASLMLLYSYMAIFELIAFAFGNFPEEEKRQALIGGAGHTVMLLKALFLTTKKLPIRSLNRKIVSICEDYEDSALMARKYKIMKINAISYLGLVNGGVLLYIIEGLRNMLNGSHFVTVVTYYPSFEDDSMLATIVRVFNTIIFIMIMLTIVISVDTYIVTYFIMYRYKFITLRKYFENLRNDFFTLIERKEVELATEKLANGLVEGIKMHSSLIRLKPEIDKAFATVSAIRVFESSSLAVCLLFEISPSDQRIPIEETVKTMIFIFALFFAMGLFLCNAGDITYQASQLTDAIFYCGWQSCPPRPRSAPNHNIRKMVLLAIMQAQRPPVMKAFKVLELNYATYIQLVRSTYSVFTLLCAQKT

>HvirOR20

EFKPFHETYKVITFTLCIAMIFPNPRTEKWRLISIPLLIATVAPVASMIFLDMYKCWTNNDIVNIIRHSTVVGPFLGGFFKMILMYHKRKEAKQILDEFDRDHFMFNDFSETYKDIARASIRNCQIYSERLWALLVTTCVMTFPVMAIVLNIYNFLFKSEPTKYMIHDLEKPFSESPEERFESPYFELLFAYMFYAAILYVVNFTGYDGFFGLCINHACLKMELYCKALEEAMVADREEVYGRVVAVIREQCRMFRYVDLVQETFNIWLGIIFIATMIQICTCLYHITEGYGFDIRYMIFVYGAVVHIYLPCRYAAKLKAMSMETSNRFYCCGWEKVDDERVRKMVLFMIARAQVPNEITAFNMLAFDMELFLSILQTSYSMFTLLRS

>HvirOR21

MDHFSGYYKSSKTTEFLINLNQFVFIFGLPNFWVQELDISDSFRKIVGYLNKYGNWSIFGLILAEYGAFFTQKNLNQRQSSDLVLFMISHSIITGFRVRICHQEVEIRNVMYKLGIALKEVHNDSEAEEQMIKRSKFFSWALILNCVISFLMYTIEAVLRVIRAGVTFTTVITVYPDVEDRSGLSNGVRVMFYIIWCIYLTRVFAVYTLVICLTIAMSHQFKNLTSYFYSLSSIFDDDQMTQAEKEQEYERAFRVGIKIHSDTLNCTGDIQKICRDVFSGQIIFNITLLIVLMYQMVNSARSLTNALTLVMVALSILLSTGFFMWNAGDITVEAKSLPTAMFSSGWEHCGRDSSVRVRKLIVIAMMQAQEPVVLTGLGIIALSYQSYVSIVKSSYSVFSVLY

>HarmOR9

MVDQFQKCLKSVNLYLKFLEAVITGKSFVEITRLIPCLILCLISNFKTLSLLYYGRHNNEFIVTMRSLLLNQMQVEEKEHRFRKELIDKHVLILTSISKKISYVIVLGLLMFALAPAFIIIPHYFKTDEVKLEMPFIAYYPFNEFDLRIYPWVYFHQVYSAVIAMIMVYGPDCFFFTCCTFIHIQFSLLNNDMERIVTEETPRYDKTKFKKLAVRHIELMRCVNLLEKIFSKSILFNALTSSVIICVTGFNVLVVDNIVMMASFTAFLIFGLMQIFLYCYYGDTIMRSSMEVSTSIYNSLWYNIPAADRKGFLIVIIRAQKPCALTANGFFKMNLSAFASILSKSWSYFALLKTMYHPE

>HarmOR19

IDAVSEASYLLFTQASLCYKSTAFMVNKQSLLELLEIMDCEIFEPKSAEHEKILAAQARKIKRLCLFFLTSATTTCTLWAMIPLFDAASKRSFPFRIWMPVTPLKSPDYELGYLYQMVSIYISAFLFISVDSVAVSMIMFGCAQLEIIMDKIQKIKYVFESADSEEGRRNIIKTNNEFLVECIKQHQTVERFIQLCEDTYHANIFFQLTGTVAIICNIGLRISIVEPNSVQFFSMLNYMVTMLSQLFLYCWCGHELTIRSENLREWLYQCPWYEQDTKFKRALFIAMERMKKPIIFKAGHYISLSRPTFVAILRCSYSYFAVLNRVNTE

>HarmOR18

MEMKVDVLPEKKYKGFNETFKLCAFSLAFAFLYPNRTTALRRCITITLIVTFCGGQLFWFITYTFKCLYTLDIYNFARNMTLAVVLVLFFIKTYYVIYATSKFAPLLDKISDDLLEANNLEEEFQVLYDDHIKIAKVGEISWLLIPTIMSALFPIYAGALMTIESIQTDDYERRMVHDMELLFVEDIRSETPFFQCMFAYNCVQCVVLVPNYCGFDGSFCIATTHLRLKLKLMTLKVNKAFKYSKSRQELRMRLYDSIKDHQDALDFYVQLQNVYGPWLFAVFLLTSFMISFNLYQIYLLQRIDPKYTSFGVVGVLHIYLPCRYASDLTRVSEEIPDDLYLAQWEAWADPSITKLLMFMITRAQQEMIVTGMGLVVFNMEMFKSILQTSYSFFTLITA

>HarmOR20

EFKPFHETYRLITFSLCIAMIYPNPRTEKWRLFSIPILIATVAPVAIMIFLDMYKCWKNGDIVNIIRHSTVVGPFLGGFFKMILMYHKRVQAKQILDEFDRDHLMFNTVAETYKDIARASIRNCQIYSERLWACLVTTCVMTFPVMAIVLNIYNFMFKSEPTKYMIHDLEKPFSKEPEERFESPYFELLFVYMFYAAILYVVNFTGYDGFFGLCVNHACLKMELYCKALEEAMMADREEVYGRVIAVIREQCRMFRYVDLIQDTFNIWLGIIFIATMIQICTCLYHITEGYGFDIRYMIFVYGAVVHIYLPCRYAAKLKAMSMETSNRFYCSGWERVDDERVRKMIVFMIARAQVPNEITAFNMMAFDMELFLSILQTSYSMFTLLRS

>HarmOR3

MIDDGFFSFNLKYLFFVGLWPEKTLTRNQKILYKMYEHFISFLTTTFIVLAGIGTYQHKDDLVVVFCNIDKCLVVYNFFFKTIIFFIKRNKLRDLIDEIEMSGDEVTEERKKLMANYVMFITGVTAAVIGAFSLLALFEGTMSIEAWLPFDPMGSLMNQILSLEILAFCVFPGLCRAFAMQGLVCSMIMYLCDQLIHLQKELRDLTYVKETEMVMRTKFKNAIRKHIRLMGYSGRMENIFKEYFLVQNLAVTVELCLNAVMVTVMRVLHIALHITSLAYLTLALQKTHLFLYLAAELIIQSQGIALAAYESTWTSWPVDLQKDLLIVILAAQRPLKLSAGGMALLCIQTFSQALYNGYSIFAVLNDAVN

>HarmOR4

GAHAFILRISSFFGLAPLRFESRSNGFTVSISGAMCVYSYILVTVLVICTIFGLVAEINVGVELSVRMSSRMSQVVSTCDVLVVVATAGAGVYGAPRRMRNMLKFMENIASVDTSIGGQYSLVTERKLCGIILAILIFFSILIADDFTFYALQAKKLDREWDVVTNYLGFYLLWFVVLILELQFAFTALSVRARFSAVNDALALTARQVSIPAEKPKSSSPLNIYAIRVAPVDSQRSANVSLLVDTMTGREHVVIIKRTASGEPRLVVSPCDAVRRLAALHGTLCDVVNSIDDSYGLPLVVILISTLLHLIVTPYFLIMEIIVSTNRIHFLVLQFLWCVTHMLRMIVVVEPGHYTIAEGKRTEGLVCRLMTSAPSTGVLPSRLEIFSRQLMLQSVSYAPMGMCTLHRPLIASVIGAVTTYLVILIQFQRYDN

>HarmOR12

MMEEEPLLIDKTVKKIEFLFRCTGINIKSGTKTRKDVIKSRTVYIINFLWLNIDLAGAVMWFFTGIANSKSYTELTYVAPCITLSFLGNLKSLFLILREKHADKLIQVLRDLEINEKARPKSEETDAIIKYEHNFVTTVISVLNVLYFVLLVAFALSPVSLVALKYFTTNELELLLPFLIVYPFDPYDIRYWPWVYLRQIWSEVVVIIDICTADCLFYTFCTYIRMQFRLLKHYIERVIPEEDDCGRLTNIEQVRAEFVLLIKWHQDLISSANMLETVYTRSTLFNFVSCSVLICLTGFNVMAISDVAFVATFLSFLFMSLLQIFFLCFFGDLLMTSSTEISEAVYNCRWYLADTSLGKDLLLVQTRAQTPCKLTASDFSEVNLKAFMKILSTAWSYFALLQTLYGAPT

>HarmOR14

NPTEYPYMILCRHLLTVITCWPKEPKEGLDTRAKLKARIWVTFQKIFHLNGCFITTIGMAMYIALHKNSMSFFELGHLYISLLMTVVIFSRVTTLCWNPEYQAVATDFLTKIHLFYYKDDSDFSMQTHKQVHKISHLFTLLLTGQMVAGMSLFNLTPMHNNFSTGKYKKGGLKNSTFEHSLYFSYPFNASSDVRGYILSNIFHWIISYLCSTWFCTLDLFLSIVVFHVWGHFKILIHDLNHFPRSLNTISFRLDQSNITLTTEMYSSRELVQVSERLNKCVEYHRRIVSFTDKMSEVFGPMLFVYYGFHQTSGCLLLLECSQMTVEALVRYLPLTIILFQQLIQLSIIFELVGSVSDKLKDAVYGLPWEDMDTKNRKTVAFFLMNVQEPVHVKALGLADVGVTSMTAILKTSMSYFTFLRSK

>HarmOR11

MHLAGNAVTGITGPMDYKYMKVLRFVLRIISGWPGKALGEKTLRIEGMGHAYYNTILSLVYLALGIAYLKKNFHRFDFLELGQLYIVLLMNMLSTSRAFTLCLSQKYREVAKIFIQKIHLFYFKEKSDFAMKIHITVHKISFISAVYLSVLLFIAACMFNLIPMYNNYSAGRFASFDNLENTTYEQAISCLYPWNFETNFNGYLAATLSGWYGTILCGSSVSMFDLFLCLMIFNLWGHFKILIYNLEHFPRPASEVVDAEGEERSGRTVGSEMYSQSELEEVAVLLRDCIQYHMLIYNFTNNMSDAFGMALFIYYSFHQITGCLLLLECSQMTAAALTRYLPLTIIMFGELVLLSIIFETIGTMSEKLKDAVYKVPWEYMDTKNRRTVLIFLIKVQEPIHVKAGGLVDVGVTTMASILKTSFSYFAFLRTF

>HarmOR13

MKILSDGSDLEGVEKVEDIFYINLARKSMWILDSWPRTPNESVTYRYFVLALNVATLVGGAVYLRNNTGVLSSFELGHTYITVFMNCITCSRCIMILSREYNEVMLSFVNKIHLFHHRHKSEYAYKTHIFIHKISHFYTVYLLGLALNGLLLFNMIPFYNCYSRGMFRDVIPANATYDHSVFYSVPFDYTTKFKGYIAMTSFNCFISYTCTSYFCVVDLTVSLVIFHLWGHMRPLTYHLANFKKPASVLESNENTDAIKDHSYTQEELKEVFGKLREYIRHHNLILKFSSEMSNAFGPALLAYMVFHQVSGCILLLECSQLDMKTLVRYGPLTVVILQQLIQISVIFELLGSSNDKLIDAVYLVPWEYMDTKNRKLVFVMLRQSQRSIDLKMMSMLTVGVQTMTAILKTSFSYFVMLKTVAEEE

>HarmORco

MMTKVKAQGLVSDLMPNIKLMQMAGHFLFNYHSENAGMSNLLRKIYASTHAILIFIHYACMGINMAKYSDEVNELTANTITVLFFAHTIIKLAFFALNSKSFYRTLAVWNQSNSHPLFTESDARYHQIALTKMRRLLYFICGMTVLSVISWVTLTFFGESVRMVTNKETNETLTEVVPRLPLKAWYPFNAMSGTMYIVAFAFQVYWLLFSMAIANLMDVMFCSWLIFACEQLQHLKAIMKPLMELSASLDTYRPNTAELFRASSTEKSEKIPDTVDMDIRGIYSTQQDFGMTLRGAGGRLQNFGQQNPNPNGLTPKQEMLARSAIKYWVERHKHVVRLVASIGDTYGTALLFHMLVSTITLTLLAYQATKINGINVYAFSTIGYLSYTLGQVFHFCIFGNRLIEESSSVMEAAYSCQWYDGSEEAKTFVQIVCQQCQKAMSISGAKFFTVSLDLFASVLGAVVTYFMVLIQLK

>OfurORco

MMTKVKAQGLVSDLMPNIKLMQAAGHFLFNYHSDNSGMTTLLRKVYSSVHAFLIVINYLCMAANMAQYSE

EVNELTANTITVLFFAHSVIKMLFFAVNSKSFYRTLAVWNQSNSHPLFTESDARYHQLALTKMRRLLYFI

CGVTVLAVMSWITITFFGESVRMIANKETNETLTEPAPRLPLKTWYPFDAMSGTMYVVAFVYQVYWLFFS

MAIANLMDVMFCSWLIFACEQLQHLKAIMKPLMELSASLDTYRPNTAELFRASSTEKSEKMPDTVDMDIR

GIYSTQQDFGMTLRGAGGRLQNFGQPNPNNPNGLTQKQEMLARSAIKYWVERHKHVVRLVASIGDTYGTA

LLFHMLVSTITLTLLAYQATKINGINVYAFSTIGYLSYTLGQVFHFCIFGNRLIEESSSVMEAAYSCQWY

DGSEEAKTFVQIVCQQCQKAMSISGAKFFTVSLDLFASVLGAVVTYFMVLVQLK

>OfurOR1

MFKIENQDDINARQPMDLRYMKMLRNLLHLISSWPYKLLGEDVKPLPLRGTFYLFVEWAIVLVTGLIYVKTHINKLSFFEMGNTYVTVSLNVVGLQRITIFWFKSYRQAIKEFVLGVHLFHHRHKTEYSEHIYQYIYKICAVFVVAIHAETFFGVLLFNVMPFVNNERHGMFNEEMPPDRQFEHSINYSLPFNYHTDLVGYIVIAIVDLILSYDCLLAFCGFDLALSVIVFHMWGHLKILDHDLRTFPTPAEMRATRGRPEDEMSYTKEENQRVRAMLKDIIDHHRHIMHFMTQASDAFGPMLCVYYMFHQVSGCILLLECSKMDPESIGRYAALTVTLNQLLVQLSVIVELLGTQSETLKDAVYSMPWECMDTSNRRTVLFLLYNVQEPIRLKPMGIVTVGVTTMASILKTSFSYFMFLRTFS

>OfurOR5a

LVLKYFKSIRTVMVAPGVWPAEIFGEKISVVAIVHRVTLPYHMSLIVFGELYYIWMHMHELSFLDLGHVIITTLLGILTAVRSILPQLQNYHKLLLKFINVMHLMHSTNKGPYYNQMNDTVDKVCSYYTKFSLGLMFLSCSMFNFAPFCNNVANVFIFKTENYTLEFSFYYQYPGIDPTDYFTTTSIYNFYLSYNCAMMVSGLDLILFLIIFQITGHVYILRYNLENFQWPKNKVVFKLGDILKYKINESITSEMFDAEEGKEVRFKLAECIEHHKEIIGFTDEVSVLFGPILACNYLFHLVCCSLLLLECSEGGYSAMLRYGPMTVLIYGQLIQMSVIFELLGSETEKLPDSAYFLPWECMDTSNRRTACIMLHKMQYKISLKALGLAAVGVSTMTGILKTTFSYYA

>OfurOR5b

LALKYFKSIRTFMVAPGAWPAEIFGEKISVLVIFHRATLPYHTSLIVVGKFYYIWMHMDELSFLDLGHMIITALLGTLTAVRSILPSLQNYHMLLLKFINVMHLMHSTNKGPYYNQMNDTVDKVCSYYTKFSLVITFISCLMFNLIPFYNNVTNVFIFKTENYTLEFALYYQYPGIDPSDYFTITSIYNVYLSYNCAIMVFGLDLILFLIIFQIIGHVYILRYNLENFRWPKNKVVFKLGDILKYKMNESITSEMFDAEENKEVRFKLAECIEHHKEIIGFPDELSALFGPILACTYLFHLVGCSLLLLECSEGGYGAMLRYGPLTLLIYGQLIQMSVIFEMLGSETEKLPDSAYFLPWECMDNSNRRTACIMLHKMQYKISLKALGLAAVGVSTMTGILKTTFSYYA

>OfurOR3

MFKIGNENDINARHPMDLRYMKFLRMLLRMIDSWPHQQLRDSKPVRFRDSRYLFIEGAGVGIGGLFYVRSHYKVVPFLEIGQTYLTIFLSVVATQRVTIAWFKSFREVITEFVLKIHLFYFRHKSNYTENVYQRINRLCSVFVAFVAVEVTIGIFLFNLMPFLNNYKKGMFNQELPANKVFEHSINYSLPYVDCYTNLIGYIVMTLINIICSYDCGMFFSSVDVCIAVIVFHIWGHLKILDHRLRTFPTPVQMRGHQPGEPGNDLMYTKEENMKAAAMLRDIIEYHGMIMRFMTKTSEAFGPTLCLYYVFHQVSGCILLLECSSLDPESLGRYAGLTVTLFQLLIQVSVIVELLGTQSETLKDAVYSMPWECMDTSNRRTVLFLLYNVQEPIRLKPMGIVSVGVQTMATIIKTSFSYFMLLRTFT

>OfurOR4

MPAVHQNPSTLSYIITVKNALGPSGIWPSNIFEDKLQPLFFRIHRETLPYHTMLIVFGGLYYLSDNFRIMSFLDMGHIILSTFLAMVTAMRSVVPNLKIYVALLTKLGREIHLMHFAHKGPYYEEINKTVDKASHIYTKFIVVFMYMTMMMFNITPIYNISKNILSSKTENSTQEYALYYSFPGINPMNYYPTTTVYNFYLSYNCGIMMCGLDLVLFLMIFQLIGHVYILRHNLENFPSPKNKVVLNIGDLPRYKNKENCIVEMFDAKENEEVRVRLAECIEHHKIIIRFTDEISVVFGPILAFNYMFHMVGCCLLLLECSAGNQIIRYGPLTTVVFGQLIQISVMFEMLGAETEKLKDSAYFVPWECMNISNRRTAQIMLHKMQDKISIKALGLAAVGVNTMMGILKTTFSYYAFLQTMND

>OfurOR6

MQQESPLQLGYIKTIRFFLRPSGSWPSDVFEGYLPLPIRIHRATLPFHTTIIVMGGLYYITDNFHRLSFLDMGHMIITTFLAMVTALRSILPNLQTYNSLLCKFIQEFHLMHHAYKGDYFEEVNKTVDKISSYCTKFSTIIMYLAILFFNITPTYNNIRHTLISKTENYSMEYSVYFSFPGFNPLDHFASTTVYNIYLSYNCSTLFCGFDLLLFLMIFQIIGHVYILRHNLENFQSPKNKITLNLRGDALITNNTCTYEVFDAQENEEVRLQLAECIEHHKIIIGFTDDVSGLYGPLLAFNYFFHMIACCLLLLECTEGSYDAVLRYGPLTILVFGQLIQMSVMFELLGSETEKLKDSAYCLPWEAMNTSNQRTAFIMLHKMQYKISLKALGLAAVGVNTMVGILKTTFSYYAFLQTMGDR

>OfurOR7

MVIIRSAFRVVGAWPSKFIGDVQTTSDVVVKYIQLVLNVVCQVAGILYLRENMDKLSFFELGHSYITVLMSVVSMSRIITYCTEAYQEIFSLYVRKIHLFNVRNDSEYAMEMHTKINKLCYFLTFFIHAFMTLGILMFNLIPMYSNYISGKFNRETGAFSGVSNATMEHAVYFLWPFNDTTDPIGYAIIVVFNWYISLVCSINYCTFDLFVYHLVFHIWGHLKILIHNLETFPRPIGAINEEQNDYTEEESKQIYERLKKLVQHHNLIIDFIARISDTFGLSLFVYLCYHQVCGCILLLECSTLELSALIRYGPLTAITFQLLIQVSLVFELLGSITESLMNAVYELPWEYMEVRHRRTVHIMLRQSQVSLNTRALNMVDIGSRTMIAIIKTSLSYFVMLRTFATDD

>OfurOR8

MSNILKYFNTRNSYELSFFREGDPLALNYFKIIRIFMVAPGAWPADVFGEKLSLLVRVHRALMPYHTSVIVIGELYYLYIHKEELDFLNMGHMIIFSFLGVLIAIRSILPQLRKYHLLLTKFVKVMHLMHFKNKGPYYKQINETVDKISYYYTIFVALLVTTAMINFNIVPLFNNVTNVLIYKTENFTLEFALYYKYPGFDPLDYFTSTTIYNVYLSYNCSIMVSGIDLILFLIIFQIIGHVYILRYNLENFPSPKIKVVFKLKEILKHKGNEDISSEMFDAEENREVRLKLQECIEHHKLIIGFTDELSELFGPILAINYFFHLVCCSLLLLECSEGGAWIRYGPLTVVIYGQLIQMSVIFEMLGSETEKLPDSAYFLPWECMDTSNRRTACIMLHKMQYKISLKALGLAAVGVSTMTGILKTTFSYYAFLQTMGE

>OfurOR9

MLYYSQLTVRRKVIKNIVDGYLACDAQTLKSDRFRQNLLKGLRIVKKRGLIFWMVIIGNGTIYIMKPIVTPGRHIMEDLFIIYGLEPMFESPNYEIGFLLTAGGVICTCYLPANITALLTVLIGYTEATMLALSEELVHLWSDAQEHYNKYLLETQVDNAGALVTPNDDIKNQIINKYIKQKLEEIVKIHTTNINLIQQIEHVFRGAIAVEFLLLITGLISELLGGLENTYIEMPFALMQVAMDCLTGQRMMDACDKFENSVYDCKWENFNVANMRTVLLMLQNAQKTMVLSAGGMTQLSFTCLMTVIRSIYSAYTTLRSMMA

>CmedORco

MMTKVKAQGLVSDLMPNIKLMQVAGHFLFNYHSDNAGMSTLLRKIYASAHAVLIVIHYLCMAVNMAQYSEEVNELTANTITVLFFAHSVIKLLFFAINSKSFYRTLAVWNQSNSHPLFTESDARYQQLALTKMRRLLYFICGVTVLAVVSWITLTFFGESVRLIANKETNETLTEPAPRLPLKAWYPFDAMSGTMYVVAFVYQIYWLLFSMAMANLLDVMFCSWLIFACEQLQHLKAIMKPLMELSASLDTYRPNTAELFRASSTDKSEKVPDPVDMDIRGIYSTQQDFGMTLRGAGGRLQNFGTNGSNPNGLTQKQEMLARSAIKYWVERHKHVVRLVASIGDTYGTALLFHMLVSTITLTLLAYQATKINGINVYAFSTIGYLSYTLGQVFHFCIFGNRLIEESSSVMEAAYSCQWYDGSEEAKTFVQIVCQQCQKALSISGAKFFTVSLDLFASVLGAVVTYFMVLVQLK

>OfurOR46

MLLKKWKEFYNKEDFDYSTGYVDPYQFHRTFYFVQSAFQVTDEPFKPWTYVSKTITLICGIGVLTDACFSFYHAIDIFDMGLITEAGTYVLMLLYKMMTLTITKVNLPSYIHFMKCMKDDFGYICTKSEKYRKVFFETQLATWKLCVSTCLFMFCLANSLVLFAIGSLFFYLATHEPGDGTNRPLVFPFWAPGIDYTSSPAYDIAFNFANIGVLACTYNYTFVLQTNIVWVRQIASKAEMIEMCITDLLEGIQPANNEEEKRHYANLINYRMREIISQHQKMLLDSYASVFKKCLMFEQLVSSPVICMLAYCSAEKLDAGEIHTVMILLCVGAILILFLPCYLCTYLRMKVTRICDACWEIRFWDAGPNIRPYLILIMQRCLRPLPLQAPGFQEVSIKTFSSKMTSAYSLFNMLRQADLDL

>OfurOR45

MDIPTFEELFKQIKLNLWFFGIPFNGRKIELRFYFMVVVVIIMLIGEISFFVSRYAPENFMELTQLASCICVGALSLLKILPIAHKKQKIFELTESLDGLYNTILENPKKKAIIRRQMILVKILMKYLFIVNIALFVIYNISPLIFMTYNYIATNEVEFILPFALGVPFSIESMATWFPVYAYSVFSSFVSVSYFVTVDALYCILTTHICSNLSMVSEELQNVDTSNEDELKELVKNHQYILKLSENLEEIFSLPNLFNVMMSSLEICAVGFNLTMGPVSEIPRSVVFLSSVLLQILMLSVFGEKLIEESTKVGDAAYNSKWYEVDQKTKKTILIIMTRSSKPQQLTAYKFSVISYGSFTKIISTSWSYFTILKTVYKPPE

>OfurOR49

MFVYHGIEVEDYSFATECFCYFVMLSVIPILYGSVLMNNRNVVLLLDKMDKDFKFICKLSFKYRDHFLKRQLLIWQLCFTWLGFLCCVAVLYVLMTLAPLTYQSLFATQDEHMIRPLIFPMWLPKDDPYRTPNYEVFLFLQMNFLLIFIQSFGVYVYIQFHVLLHNFILLELVILDFDVIFEGLDESVVGLSRYDPRRASIQHVFNKRIERIVTWHDSVFKSIATLSTVQGPVIVYQVMFSSLGICLMMYQVADKLDSGTFDILFMLLTVATTLQLWIPCYLGTLLRNKAFDVGDACWNCGWHETSLGRMIRNEILIIIMRAQHPISIKFTGLPNLSLETFSSIMSSAYSYFNMLRQYNK

>OfurOR42

NRLSNRLYRIYQYSLFIGSFIFLISTGIGTYMSKDDVIRLLSNVDKVTITYNYFFKIMIFLIKHEQIKSIISSILHSGDQIDVNRKYLMKIHVVMVTLLVTSITGAFQFLAQIKGELIMDAWFPFEPKKNKLTFLAATLIISILFVLPFMFRAIAIQGIVCSVVMYLCDQLVELQRRLKALKYSVESETYLREEFKDILKKHIRLMEYSKSIKSAFNEFFLVQNLAITAELCLNALMMSLIGLEQKNHLVSFMAFLMMALFNAFIFCHLGNNLIDESAGISLAAYESTWTSWPVDLQRDLLIVITVAQKSLSLTAGGIADMSMQTYAQALYNGYSIFAVLRDVVN

>OfurOR52

MPEKVYCALKKLTQIELDFLSILGSKLFLYPFIGRTKRVYACYMLVSLLLIVTAAQLLTALLVADVKEWIEIINVAPNLAVVLMALLKYTKVHVNRDLYKKIFEHFRDELWDVISDSVEHQNIVTQYAAKTKFISRFLIYYSIPLVIFVDSFPRIIMYLERELLGSDDTKYLYPFDGWYPFDKVDWYYTVYLWESFMTMNVVVVYAFTNMIHSSYTLFICLELKILGSSIKNLVTSEDVANIVNRTKLQETHGDIKRKLKTIIIRHQLLAQIVSEFDIVQGDVTLANYLFGSVFLTLTIFSSTVVDNMYKSLRYFFMFCSLLIEVFFYCMIGQILTDDSDCLTDAIYSADWPYANTETKMALLIIMTRTQRPFKYTAKGYIAMNLNSFSGVCSMSYQFFNLLRTAYSQ

>OfurOR50

VFVTLTTALHFVFYTLNLIYTPRKIEIFATQAVYYFSSVSGLFKIGTVLTKQQEILSTFEIIDCKEFLGNCSETKKYLQQFRRTYFRYFRLYLTFCMCCAVVFLCGLPLVNYFFRHEDLKLPVCEYYFLTDEVRKNYIFYWFDYQILGLIVTIVYNSTTHTFLCGLILMGITQFKILNFNIANIRLDEDIEINNQEEREQALINKLNQCLKHYDIILKYCENVQNIADVFFFVQFSLAAITICFCMYMLILDLSDKDKVFTTCFISAMLLENYTPSFLGSHLTAESDNLRIAAYSCNWTPRSHSFKKSLILLMERAHRPVVIVALKMVPINLETFASMVKTAYSFFTLLSGAQE

>OfurOR41

MNLLNFFKKYTEDDLINIQEHHFESFNKTYQWIAFTLTLGIMFPNPATDRFRIISINVLLVCVFPLAMMVLIDMYKCWMVKDIFNIIRHSTIVGPFLGAFFKMFLMYYKRAQAKEILDEINRDHASFNFLPRKQQDIAFLNVKKGVFNVERLWAPIVSIAIMTFPGMAVVMTLYSYAFSDNPKRYMIHEVKPPNSRDPEDMLKSPYFEILFVYETGSAIICVLNYTAYDGLFGIATNHACLKMSLCCMKLKEAFRCDSTEDMYKGILTFIEEQKKMFRFVDLIQDTFNIWLGTILTSTMIQIGSLLFHISAGYGFDLRYTLFSFTSVVHIFLPCKNAATLKDMSTEMSTMIYSSGWERSRERRILRMIPFMVARAQVPNYITAFNLFIFDMELFVFILRTSYSMYTLIRS

>OfurOR37

MIVKNVTTSVSMSLTALRLVGFWMPEHFGGNKRILYDCYGFFSFMFLLGTYLIIQAVDMCMIWGDLPLMTGVAFILFTNLAQATKIFFMVWRRKQVLTIIRGADEVLRAVESDEAKAIVKSCSRETTFLHIVYNCLTLVTMVGWGTSAEKNQLPLRAWYPYNTSKSPAYELTYMHQIGALCVAAFLNVCKDSLVTSLIAQCRCRLRLLGLSLRSLCKDLHATGKQYTAEQEAIVRARLCACVREHQAALVAAQQIQDVFSEQTFAQFNVSLVIICVTAFQLVSQTGNLVRLMSMGTYLVNMMYQVFLYCYQGNQLSEESAMIAGSAYECPWYLMSISLRRSLLIVMIRTRRVSKITAGGFTTLSLASFMAIIKASYSLFTLLQQVEGKK

>OfurOR36

MKDYEILKKHCKRIYLIGSGDFWYEDGTIGDDKSWYYKVYSWSLLSVYGFMTILEIMAAMIGDYPEDEKRDSVTFAVSHTIVMLKIFSVHSNKQMIKAMNKNMVYICEAHEEPTLMAEKYKIVKINVLAYFSIVYGSGLFYVFEGIRKIFAGSHFVTIVTYPPSYEDDSLYSVAFRVSTTVILFMLLLTMIVSVDSLTMTYLIMFKYKFITLRNYFERLTEDFYKMNDVNPREAADKLTNGLVEGIIMHKELLRMAKDIDQAFGTVIALQLCQSSGSAVSLLLQIALSDQLTFVASMKIIFFVAALFFLLGLFLCNAGEITYQASLLPDAVFYCGWHACARQPPRRSARRIVLLACAQAQRPIVMKAFKMIQLSYSTFLQVLRGTYSVFALFYAQNK

>OfurOR35

MGIVMENVKKRLTILQPILPYGVIEPWDDLNPRLYHAIHIYWLKFYGMWYNNASPKTIVFWLQLIYTATVLWLVCFLPGIGEVVYLLKRRGNIGDVAEGLYLFLSEMYTYFKVAVFWLNKDKVINLLRYLSCEEFKPVEMEHREIIRKSIKAARFVMTYYSTMCVGAVSVGIIMPLTENFDILPTNVEYPYFDVYKSPVYQTLYIHHVYYKPATCIIDGVMDTILAAFVASAIGQIEILAFNLRNFDVVAERRRKRAVAENKPSAAWTQERHIRAVLKDCILHHNSIIKYVSMIEGTFSLASALQFMLSVMVLCLVGIQFLSIENPSSHPMQIMWMAIYLTCMLIEVFILCWFGDELIWKSTALRQAAFDGPWLETNHKTMVFIVIFLERCKRPLRVTAGKIFTLSLDTYTILINWSYKAFAVVSNMKK

>OfurOR34

MTSTQANGNRVYSRNDYDETYKLIITNILAKVGIRMTRKDSKYARLGWNVFFCFGFGNMVVTLFLDLVTFQDVVRSGVGEDGYIVFMMLPCMGYMALAMLKTYKMVYKRDVFENLISELREMWPEGLVTEEEHTIISRALNELNIIVKGYYWCNLGLGVSFMAPSFVVAIRRIFGADIPPSLPYFYWLPYDQSQPVAYEFTLVMNTYHTLLTLWYMLAGDLLFCVFLSHITTQFDLMSVRITRLFQVPVDQQLIPEYPLGQQIKDFPENGHLPRLSNNEINSKQENELQKIIVRHNALIRLSGDVEDLFSFAIFINFFNSSIIICFCGFCCVMIEKWNSLMYKTFLATSLSQTWLLCWHGQKLLESSERVADALYNSGWYTAANGIKKSILIMIHRSQKNVYVTTYGFSIICLASYTAIIKTAWSYFTLLLNTYNP

>OfurOR33

MEDTIKVFHRVLSFAGILIYAEGNWDSKLWLAFQIFNFIIGSLSFIFTTGFVVVNCSDLLIFIQGACIWTTGVIMTISLGVCLIFRKKFRIFLGEMVFKDEILEMPLIQFVLKLESGKKLIELKQMVNDSQEKLFRLTKVLLKCYVTSVWLVATMYLCSPIYEMLSRGDKSLRLLAFDMWFPWSLENFKVYVISFVFHAYAGYLCCVAYPGLQLTIILLIGQVIRQLRILTFVMENLDELVMEIIKEKGDRWQMCCTAVLSQCVDHYIKLKRFSNRLNVICQPFYLTLILVAIMLVCMCSVKIAISEKLSPDTIKYYVHEFCFIMVVLMFCLLGQQVDNECANLERAVTENWYIFDKKHKIHVRIFKMALSQRMRVFIFGSIPLSLPTFTWFIKTGMSFFTLVMSVLED

>OfurOR30

MATFNSEDLFLSRAKFVMKFLGVWMPPVDETLPRKLFKIFMLTLQYLFLIFQTIYITQIWGDLEAVSQPSYLLFTQACLCLKITIFHVNIDNLRELLKQMGSEVFLPQSRVHEEILKTQAARIKRFLLAFMISSQIVCTIWVVHPMLQKTGPRKFPFDMWMPVSPDDSPQYEIGYAFQLLTICMSAYMYFGVDSVALSLVIFGCAQVEIIKDKILSISPVQHRLKESERKIIFEKNHKILVECVIQHQAVVTFTQLVEDTYHWYLLFQLTGDVGVTCMSALNILAQEVRSLQFVTILIYVIVMLSQLFICCWSGHELTATSEGLHTVLYQCIWYEQDLKFKRDLRFVMMRMSRPMVLRAGHYIGMSRQTFVAVLRMSYSYFAVLNQANRVEQQ

>OfurOR29

MKWKKNKYGSRIVSTKKDNEFLQKNNLVTMITHRIKNIGLTFCVSDGIKIHWLAIFAIISFVLTQGLQIIGLFNAKDDIDKVFEYFSVMSFCGMGILKLLSLCRNHKQWKILLDNIKQLEKTQCQNETSNVEYESDGENDTFTFPSYIESYTKKFKIVSTVLSRMYGFTAIVYILSPFAEFTLLIMTGNEDYEKPHVLPGWAPFDSRSFVGYLTNVAVEIISVTYCVLVHITFDLTSIGVMIFICGQFSLIRDYSSNIGGSGASCTLSKRREDRAHHRIITCHKIHCLLMNTCDELGKQLQNILGVYFSVATLTLCSVAVRLNSELSRMELASLLQFMCATLTQLYLFCHFGHNVLHQSSIGMGDGPFGAAYWCLSPRIRQELVILGMGMMMPRYFKAGPFISVDLPSFVQVLRTAYSYYAVIRK

> OfurOR26

MAKEAFENSLRLTKLFLLLSGIRITRRKWRKSVENFFDYYLYYISLSWLYTDVCGELNWLIEGILTGKSFIDLSLTAPCITISMLATSKSIFLYWNRDVVAKIVDKLRDIHPEDKEFDEYKQLGLYQVESNEPDVEKEIVEESRKFLSFVVHLLFYICAVVICAFPLMPVTSMAYDYYTTGSTECKYPYLVKYFFDPYTMKMWIAVYFHHVVSTAIVGANVFGSDSLFYVVCIYIQMHFQTLCHRCECAVVSSREGTRRNVANAVKRHQELIDLVNQVELLYSKSTLFNIVTSSVLICLCSFIITVLDEIIVVVTFATFLVMNLSQISLLCYFGDILMRSSTEVSSAVYNSLWYETDQSVKKSMLVILMRAQKPCKLTAWNFADLNLTAFTTILSRSWSYFALLKTMYK

>OfurOR25

MEEESLFDKSLKKITFAFRLTGLNIENDKRNLKQNCVYLFNFLWLNTDIVGALQWVLYGIASGKNFTELTYVAPCLALSILGDIKGVFMILNEKKVHILMDNLRSLELKAKEFENSEREDMIEPEIKFLNIITSVLNVLNCLMIVVFDASPLILIAVKYFTTGQLELMLPFLDVYPFDSFDLRYWPFAYIHQIWSECIVLLEICATDYFFFTCCTHIKIQFKLLQHQFQEIIPSRSVSAVDSIDQAAIRTKFQELIKWHQEIIRSANMLEGVYSKSTLLNFCTSSLVICLTGFNVTTIDDKAFVMTFIIFLFMSLLQVFFLCFFGDILMSSSMDVSNAVYNSRWYLTDVMMGRNVLLVQTRAQDPCKLTAAGFADVNLRAYMKILSTAWSYFALLQTIYC

>OfurOR21

PRLARAHALYCRFALAATSVYLAQECVYAYQVRNDMDKLARVMFLLLCHVTSITKQLVFYMSADKIDEMINALDDPLYNQPAAWQRALLAATARSAGRLLRAYSGTAVVTCTLWIIFPILYYSQGLPVEFPFWTNLDHSKPTFFVILLMYSYYVTTLVGIANTTMDAFMGTVLYQCKTQLRILRMNLENLIERATTVVKENSDEIFDKVLDRLFLECLEHYRQISETNRRLQDIFGTSILVQFGIGGWILCMAAYKMIGLNILSIEFASMTLFITCILTELFLYCYYGNEVTVESDRMVEAVYAMEWLHAPLRFKRSLVLVMERAKRPLRPAAGHLIPLSLDTFVTILKSSYSFYAVLRQTK

>OfurOR20

MWNIRFLKEKRFTILNVFNFLEDPRYPLVGPHLRLLGLTGLWHPNLNSKTRFKQYLFFITIAFFFSQYVKCAVKLEPSSLMLILQYAPFHLGIIKSCFFQKDHKKWESLIDYISGVERKEIANGNKDSNDIISEYISRSRKVTYFFWALAFFSNFTIFTEPYQKNQINVNGTSVYLKIFDGYTPFSEVPPGYYASMLTQTVLGHIVSAYVVGWDTLVCTIMIFFAGQLKISRLNCANVIDINNAERSHENIVNCHSFHTILVKNQKLFNSLISPAMFVYLIVISVNLGVCIIGIVQLQDDLTTLISSCVFVMACLIQLLLFYWHSNEVTEESTLVSYGSFECDWVELDQRFKKEVALLGMATRTRLVFKAGPFNEMSLTTFIAILRLSCSFYTLLSKTM

>OfurOR19

MEGLEAYPEEFVNSLKLSLEYYKRVNITFFGSKTSFWDKYRHFFVFGIPFAFFYYTVTMYMVKVVAEGLDPFAKPDMIALWLISTQVIFKYILFTKNKEGVRLVIEHLGAVWRMTDLTKEQILIKNSSLKFLKYGLYIYNKSCMTTAWQYFLYPFISMLFKHIFWGNEIEMVLPFPCEYPFAVDNWPVYLAVYALQIIGALQMVHLYLAPNFLLTNLSIHISTQFRLLQDDLINIKPTNNKKTKYQYDMEITKYYEGKEYTIEDFVRRHQDIILLTRQLNDAFNKMVFVNLVISTVVVCFFAVAVKTTIDPAYKLTNGAALVAYMANLLIVCYCSEMLSISSTGIALSAAKNMWYDGDLRYQKIICIIIMRSQKPCTLLALNYYSISMKTFNKALKTTYSYFSLASHIYDGRKERKYTTEY

>OfurOR18

MKFPWRRGGMRPKEPLSTLDSLPINNYTHFLEIPLKIVGCWDWYDHPKSEKEIIINNVYFCMVLFVLINVPATLYIHLHTEWVDVMTSLDKLADCLPFVVSIIIVVYFGLYRKELYELTKFMQRKFHYRSANGLTNMTMLNSYKTARNFGYFYTACTMFSVSMYMIPEIVNRLKRQPLQSYMYMDVTRTPFFEFTLLRQCVAQAFVGLAMGQFGVFFASNAILLCGQLDLLCCSLRNARYTALLRCGVSHRSVAAAHSDIQGDELYNYIYNIAEMRQSIYHYDQRMSYEIMNKRSSFDIYSSEFDAATCEALRDCARACDVINTFKAKFESFVSPLLALRVVQVTMYLCMLLYAATLKLDMVTVEYLVAVALDIFVYCFYGNQIIIQADRVSTAAYQSAWPTMGVRPRRLLLNILLANKRPVVVRAGNFLSMDLHTFVVIIKTSFSYYTLLVNVNEK

>OfurOR17

MEKSPNFEFGYALVAASVWFLCYVPANVTSVLIVFAGYIEAQMLALTQELLHIWADAEQHYANINLNTLKRGTFVDAKYKKRVINEFITMRLHDIIRKHATNVHILHLLEEVFKGAIAFEFLFLIMGLIAELLGGLQNTILEMPYAFVQVAMDCWTGQRVMDASAEFAAAVYACNWEMFDVPNMKIVLLMLASAQKTMKLSAGGVTMLSFECLMSVVKNIYSAYTTLRSAFTINTHAH

>OfurOR16

MSLWSTIRKFGLGYCDLPTMLWNVSFMLRALTLNIDSRYKKRIPLIFYIIFALVAASYFYIYLISMAWFVFWHSRETGDLVAAMVVASLGISSEIGTAKLIYMFLYRNKVRELVDMYLDCDALVKPGSRFANNLTKTLRNVKKRAMIFWIVIMGNGVVYVLKPLLISGRHIMEDLFTPYGFDPVYESPNYEIVFLLMTAGVLFTCYLPANITAFLIIITGYTEGQMLALSKEMLNLWSDAQQFYLDHRTFDLDTTRPVVTLDSEQITKKKIVNEYVKKRLHEMIKIHTTNINLLNHVERVYRGAIAIEFGILVLGLIFELLGGLENTYLEVPFALMQVAMDCLTGQRVMDASKAFEDAVYDCKWENFDVANMKTILLMLQNSQKTMRLSAGGVTTLSFSSLMMVFRSVYSAYTTLRTTMNK

>OfurOR14

MTILNSIWRKLTNTKALEKSSGCLETQFFETVYRVSYLTGISMADEDIPYLIYSSVVKLLIFLLIVGEFWHLATEVTSFDEMADMVNITVIQYIAIFRYRSMLYHKDVYKKLAISMESQYFDISTKERRDVVDYWVKRNANNVKLLLVLGNCTLIAWFLYPLVDDLEYNVFIGIRLPFPYYSPVCYAFVYLLLLIVFSYISHFVMANDLIMQAHLLHMVCQFDVLCNCFENLMEDCAKGFKGIDRESLLANANYREVFKARLGDMITQHRYILDHAMELRHTLSGPMLGQLAASGTLICFIGYQATTSGAYNITKCLMSLFYLCYNLLVFYIICRWCEEISVQSQRVGEAVYCSNWECGASNIPGVKVSLLMVITRANKPLTLTAGGVYDLSLMTFSSILKTSYSALTLLLRLKSTE

>CmedPR4

GVSFCMQDLFLCLMIFQLIGHIKVLVKTLRSFPKPQRGAPLEYRRRNGSIYMVDIVKNFNAQENETIRKLIKECVDHHVMIVSFSDNISSFFGPMLGVNYLYQVICLCIMLMQCMMGPAAMLRYLTLTLGTLGQLLQFSIIFEIVGVEVTIFLLLYY

>CmedPR3

RYGDVMTNFLTNFHLIHFKHKSEYSKKIYEEVNKISLYFTRIMFGMTWTGVMSFNLTPLFLNYRSGLYHELIRGQATNLTMQFAVRYSFPGFEQEDHFLLSSLLNLLFSYMCGFTVCTVDLLLFIIVFQIIGHIRTLRHNLEVFPKPREMRDSLLKGIASDRVKFVRNFDDRENARIKTLLDDCVRHHLMIVSFTDEISSFFGPILGFNYLYHLVTCSLLLVECMEGKGAYMRYGPLTLSTLAQLTQMSVIFEIVGSESDKLKDAVYFVPWESMSVRNQKQVCFFLSRVQ

>CmedPR2

MTAINKSGQRTEDPMTLKYMKLIRSMLITVGLWPGEAVAGKPHRIPFANMFITWQSCFCIYGELLFIYRRFRILSFFVLGDVCIAFSLTMLNLVRAVFPYSDTYGAIFHDFVSVFHLKHFKHKSEYARKTCETVDRISYYFSLYMTVIMVIGVSSFNLTPQYHNYQNGIFKDDAPENITIEFAVYYSFPYFEQEDHIAVSNLYNVFLSYICAVEVCILDLFLCVAVFQTIGHIHTLVNTLRSFPGHRKLQHPVQFRKSVGADSGSISVEIIRDFDDEENKIIKKLIKECVEHHFFIVSFTERLSKFFGPLLGFNYMYQTFCLCILLLQCMGGGGALMRYAPLTLITFGQLLQFSVTFEIVGAESEKLKDEVYYLPWESMSVSNQKAILLFLRRVQTPIHVMAMGMTPVGVKTMGNIIKTTFSYYAFLRTSQGLK

>CmedPR1

MLLHLFGHFMFIYCRFHELDFTMLGDVYLTMIFTCLIIFRFSLLITEGYGKLFWSYLREFHLSHFKHRGEYVQQLCEKIDRLSYLFTLYQIALAMAGTVTFNITAFVINLSRGAFRTPRPENITLEFSVHFMWPGFTIEDHFYFTSIDNLFNSMLFGVSLCMQDLFLCLMIFQLIGHIKVLVKTLRSFPKPQRGAPLEYRRRNGSIYMVDIVKNFNAQENETIRKLIKECVDHHVMIVSFSDNISSFFGPMLGVNYLYQVICLCIMLMQCMMGPAAMLRYLTLTLGTLGQLLQFSIIFEIVGVESEKLKDEVYFLPWESMSVSNQKAILLFLRRVQTPIHVMAMGMTPVGVKTMGNIIKTTFSYYAFLRTSQGLK

>CmedOR46

PKFWLIVGLRVLMGIFIFTIPLCAQLLYAIKIVMSDEPQIQEIASSINLALTEFLTSFNLLDLFLRRHRLQQLIDLLNCDEFTCFNNTQRNILERGVKFSRRAFLILAVGTTADVLMHMLILPALNHFETLPVKMELIFFDANDPAYFNYVCAYQILYKPTLITTFVALYSMNWSFMSCTSSQLDVLINKLENMNELVKITTVENSCDENEACEVIYKNCLLHHSAIIKFSRTFESTFNGQLSMTLIMSSCVIGTTALQIISIESPRENVTEVIWVLGFLIFVIGSLFIDCYLSQDITDKSVHIPTLIYSSPWLNLPTKMKRNLIIFIAKTQQPIVLKGSQLIPISIDTFTTIMNWSYKGFAVMNQMKK

>CmedOR45

MTRIEDLHQSHGKGYELVVKVIGTLTGFKIFEDSSNTHWNFFKKTVTVVCYVTFAAWSLISVFDGDDADTILPALAIVSMGIQYLPKLYLLLFKPKVFAELIASIDEVWPSTSRLSPEYLEEFNNSLRQSSTFFTGVLLFSGMQSILIQSYGLYKTLWAKFLNPNVKYTFPFEFCYPRETDSLLVFVIIFAIQWSGCVMNGMGIWDGVGCLFGTIVSSVSRLFIVVQSDLKNIVKARGDDAPRSVEETEEDYNKLKAVVVYHQRVLKIADALNSMCTVLVFLDVTFTALAIGIYGFRTTTMKDSATKLINFFSGLYVLQITLIWSWTGQLLADASAEVAIAAHSCLWYTGDARFRRLIGIIMIRSQSPVYLTSLGFSRVTLQTFVKIMSTAYSYISLFSQVYE

>CmedOR43

STKVKLHNGYRAFIWLFVLSYNLQHGIKVIQTRHSTDAMVDILFILLTTLNTLGKQVAFNARVDRMDRIIRVINGFDFAPKNSAHVEILKRNATSMARILYIFLSMVLVACITFWVYPIVNKAMGQEVHIAYFPFDTNNSPVFELAVCYLSFNLSFQAFGNGTMDCTIAAFYAMAKTQLSLLRYNLEHLVDKEDDEGISEMSLRYKDSKTIQKRLENCVKHHRQILWFVKEVESIFCEAMAVQFLIMAWVICMTMYKIVGLNIASLEFMTMIIYLNCMLAQLFIYCYFGSQVKVESEFVAQSVYNGGWTQLSPSFRRQLLVLMQCSSRPIIPCAAKIVPISLDTYISVLRASYTLFTILNEK

>CmedOR42

FVILLVYSYYVTTLVGIANTTMDAFMATVLHQCQTQLRILRLNFENLTQTATEIVRKNPEEVYDQVLNKLFIECLMHYKHIIETNKRLQDIFGTAILVQFGIGGWILCMAAYKLISLNVLSIEFASMTLFITCILTELLLYCYYGNEVFEESDRVVQSVYGMEWLHAP

>CmedOR41

MDKTFKIYHRINSIAGIAIFAKNNWNSRAWLAYQVFNFIIGIFCFIFTSMFVIVNSSDLIIFTQGACIWSTGVVMTISLGVCLNFRNRFRVFLTEMAFKDEMLEMPLIQFVLKVERGKKLLELKNMVLDSQEKLLKYLRLWTTGYIISVVITTTMYLINTIYEMTTKGDSSLGLLAFDMWFPWSLEKMSVYVPSFIFNAYAGYLCCFAYPGLQMTIILLLCQVIRQLRILTFIMTNLNDLAIDIVEDKQDMWQVYCTSILAQCVDHYIKLKGFSNRLNVICQPFYLTVILASIILVCMCSVQIAILDKLSPGTIKFYAHALCFIIIVLMFSLLGERVDIECGHLELA

>CmedOR40

MLKKKIKAFFNKEGFDFTEDHIDPTKYHVTFFFMMRAYQVADEPYKTWTYIFKTFITICGIGVLTDACLSFYHAVDIFDMGMITEAGTYVLMLLYKMMTLTITKVNVSEYVHFVRTMKEDFEYISTKSNKYKKAYFETQLETFKASVVVCTFMFCLANSLVLFAIGCLLFYLATHAPEDRTQRPLVFPFWAPGVDYTTSPAFECAFYFANVGVCACTYNYTFVLQTNIVWVRQIASKAEMIVMCIKDLLEGIQPAKNEQEKKYYASLINFRMREIVTQHQSLYRLIDSYACVFKKCLMFEQMVSSPVICMLAYCSAEKLDQGEIHVVMNVLCVGAIVILFLPCYLCTFMREKLTLISDACWEIRFWDAGANIRTYLILVMQRCLRPLPLQAPGFEEVSIKTFSSKMTSAYSLFNMLRQADLDL

>CmedOR39

MLALNSFWRKLTHTKALETSSGRLEIEFFETIYRVTYLTGFSSADDAFPYLVYSSAVKLLIILLFCAEFWYALTATSSIDEIAASINTITIQLISVYRYGKMIIHKDVYRKLARSMQSPHFDISTVQRRNLVNYWAQKNENYLKLLLFLGNCSLVPWFLYPLIDELDYNMFIAIRLPFNYDSPMAYALTYLGLVMVFMYMSHFVMGNDLLMQAHLLHLVCQFTVLSDCFENILKDCALDFKDQDWNVLVTNDRFREVYKKRLGDMVYQHQNILDHVMDLRRTLSGPMLGQLAGSGLLICFLGFQATTTGADDVTKCLMSLLFLSYNLFEFYIICRWCEEITVQSQRVGEAAYCSNWEFGLTDLKGVKSSLVLVIARANKPLILTAGGMYNLSLLSYTTLVKTSYSALTVLLRFRQN

>CmedOR33

MSDKSYDWMKNKLLHELNFLRSLGIMIFIYPFIGQSKTANIGYGFVFFLIILTATQLIITLCLKDFKDWVQIVNVVPNLAVVLMSALKYITVYMNQPIYHKIFEHFRNDLWDVVHDCKQHKKIVVKYSVLAKFVTRFLFYYSVVLIIFVFSFPRLMMYLETFLTGEECEALYPFDGWYPFDKAHWYYVAYIWEGFMTSVVVCIYGIPNMFNSSFTIFMCMELKVLGRHIENLITRDDVVNLSKYESVRKTHLDIKRRLRYIIIRHQFLAQISAEFDAVLGDAMLVNYVFSSVFITLTIFTATAVENLYMRLRYFFMFCSLMVEMFHQCMIGQILSDHSEELAEAIYSADWTYADNSTKKMLLILMVRTQRPFEYTANGYLAMNLQSFSGICSMSYQFFNLLYTAYN

>CmedOR32

MEVFERVKQSILNTKFRLQQNTYASLLWLVNIVPSIAGFSIQRDTISAPFWIAHLSLLVYIYGVGNVVYQIEYAQNAGDFIKSYVNISLFVLIANNSYWFIKKRPLLKSTLDQISESDQLSIVNETFREQHARSVSKIKRILFIFYGFNYGNAAFVYLPHRVDVRNNYAMTPCYGMEPLTSSPNREICLALLCAQEFSIMTVVLNYQALLLLLIAHTALLYDLLSAEIMTLNNFDRKLHYNNPAAKDLLPVIIKRHALILSVIDKLKALYSVPIGVNFGSNAVCISLFFYLPLQEWLQFMPILVYCFLVFFLYCFLCQRLSNAAEEFEIAVYACGWENFDRNNRKAVYVMLRQAQRPVELLAADIIPVNISTFATTLQAMFKFVTVVKF

>CmedOR31

MFAQKIISWLEQLEDPRNPLLGPNIKILFLFGIWQTGDTKLRNKLFNIIHVSTAFFVLSQFIDLYKQRNDFNKALNNLSLTSIGLICCTKCFSYVLRQPQWQKLAANISEEELIQIKFGNEKVIKKMEQYKLYARVVSYLYWGLVLMTNVVLIGTPMLKLLISETYRQNIKNGKEEYPQIMSCWFPFDYNKMPGFVYSSVVQIFMALQGSGVLAGHDANAISIMTFMKGQMQILKEKCANIFEVKENEDPKEVLNRIKECCRHHSFLLQQSELFESLLSPVMFLYLMICSMAICCSVVQFSSDEATATQKLWAVQYTIAQIAQLFLFCWHGNEVFTESIVVDQGVYASDWWRGDLRQRRLVLLLAGKLNRPILYTAGPFSNLTVPTFISIMKGSYSFFTLFSQMQEAN

>CmedOR30

DWTWLQRSIYKAYENSLLLTALVYLALTGIGMYTKKDDMIVFLENMDKAIVAYNYLLKIIIFFIKRDQIKMLITEILHSGDTIKEDRKKLMKYHIVVITGFIFVVMSTFQMVAQVKGEVVVDAWLPFDPLKNKWTVFLAGQIMGVLFLVPYIGRAIAIQGIVCSIIMYMCDQLVELQSRFKALNYTVTNDIPTRKELKEIIKKHIRLMGYSRSLKSVFKEYFLIQNLAITAELCLNALMVSIVGMEQKKHLVTFMAFLIVALIVAYIFCYLGQELMDQSAGIALAAYESSWTSWPLDMQKDLLLVITAAQKNFSLSAGGMAKMSMETFALALYNGYSIFAVLRDMVD

>CmedOR29

MEKSPNYEIGYTLVGASVWFLCYVPANVTVFLIVFAGYIQAQMLALAEELLHLWPDAELHYKNLDFSEFRRYNFNKERVLNDFIRRSLEDIIRKHAMNVNLLKQLEELFAGAIALEFMLLMLGLIAELLGGLQDTYLEVIYAFVQVAMDCWIGQQVMDASVAFERAVYGCRWENFDVSNMKVVLQILGNAQRTMKLSAGGVTMLSFASLMSVVKSIYSAYTTLRTAIK

>CmedOR28

MIKQFLLSLEDPKKPLFGPNYWILKKMGLILPDSSLGKFLYVLLHEIVSFFVFTQYMELYIIRSNLDLVLTNLKISMLSVVCIVKSNTFVFWQAAWKQIIEYITEADSSERNTDDPSRKSILEKYTGYCRRVTYNYWVLVFTTFLTTIGTPLMHYLSSSSYRESLHNGTEPFPHIFSSWMPIDKNHFPGSWITVAWHTLLCAYGAAIMAAYDTSIMVIMVFFGGKLDLLRERCKTMLGTDGVPVSDDEASAKVQELHEIHVQVMKYSRLFNSVLSPVMFFYIVLCSLMLCASAYQLTSSNNAAQKLLLAEYLIFGIAQLSIFCWHSND

>CmedOR27

MPKKFFLISDGSDLAGITIANNIRYIRILRNSMLWLYAWPRKAVGEKERKTMFYERIFIISLIIACIISSSIYVKNNTAKLSFFELGHTYITILMTVVCLVRSFLIISPSYHKLMADFLAKIHLLNWKEKSDYAMKQHLLIHKMCHFFTMYIFGTMYSGMILFNMTPIYNNITTGAYNPAKLGKENVTLQLAVYYALPFDYMSTYKGYAIVFSFNCYVSYMCSCWFCIIDLMMTVFVIHIWGHLNIITYYLENFTNIQGLDGERNVPWYTEEENHKAFLGLRDIIIYHSQIVKFITRMSQIFGPSLLVYYAFHQTSLCLLLLECSQMSTEVLLRYGPLTVILFQQLIQLSLIFELLGSMTDKLVNGAYSVPWELMDTRNRKLVIVMLRQ

>CmedOR26

LGPNIWISKKCGFVLPKSSVQRFFWMLYHSAGAFFVISQFVELYVIRSDLDLVLTNLNISMLSTICVMKGWSFFIYQRNWMELIEYITEADIFERKTENPVRLNILKKYTKYSRKVTSFYWILVLITSFVSFSAPAVQYISHRIAISSSNEGSSNNETETFTEPFLFPHMFSSWMPFDKYNWPGNWFTVIWHIACCYYGSGSMSSFDSMVMVFMVFFSGKLELFRERCKELFGKNGDQISDEEATAIIGELHQIHVNVLKYSRLFNALLSPIMFLYMVLCSFLLCANGYRITSTTSIVQKLLLAEYLMFFISQLFVFCWHSNDVFVKYQSVSIGPYESEWWAVRTVKQRKNIHLLAGQLHVMTVFTAGPFTNLTLATFITILKGAYSYYTLL

>CmedOR25

SDFDESLKKINFAFRLTGLNIQNKKRTRKQNCIYLFNFLWLNTDIIGALSWVFEGILNGKNFTELTYVAPCVTLSVLGDVKAIYLLLNEKKVLNLIEHMRNLEKKANGFVNSEQENLTRPDIKFLNVVIKVLNVLNCLMIVVFDLSPLILIAVKYFTRGELELMLPFMDVYPFDGYDLRYWPWAYAHQIWSECIVLLDICATDYLFFTCCVHLRIQCRLLQHQFQEVIAPRSISAIDSIDMEEVKNKFKELLKWHQDIISTASMLEGVYSQSTLFNFLTSSLVICLTGFNVTTINDKTFVITFITFLFMSLLQVFFLCLFGDILMNSSMEVASSVYNSRWYLSDVDMGKNVLLVQTRAQKPCKVTAAGFADVN

>HrhoOrco

MMTKVKTQGLVTDLMPNIRLMQIAGHFMFNYHDENAGMSFLLRKIYAGVHAFLFVIQYVCMGVNMAMYSEEVNELSANSITMLFFAHSLIKLLLFAINSKSFYRTLAIWNQSNSHPLFTESDSRYHQIALKKMRRLLYIICGVTLFSVTSWVTITFFGESVRFIVDKETNETLTEPAPRLPLKAFYPFNAMGGPMYVFAFIYQIYWLLYAMSIANLMDVLFCSWLIFACEQLMHLKAIMKPLMELSATLDTYRPNTAELFRASAEKSEKLPDPVDLDIRGIYSTQQDFGMTLRGGGGRLQTFGEPTPNNPNGLTQKQEMLVRSAIKYWVERHKHVVRLVTAIGDAYGTTLLFHMLVSTITLTLLAYQATKINGINVYMFSTVGYLLYTLGQVFHFCIFGNRVIEESSSVMEAAYSCQWYDGSEEAKTFVQIVCQQCQKAMSISGAKFFTVSLDLFASVLGAVVTYFMVLVQLK*

>HrhoOR1

MKRPTKHIISLINSIIFLFPLNGIKLEEINSMNTSMYRFFFVLNFLCMNFDAAGEIIWFLDGLWSGKEFVMLAHIVPCLIMTFLGNIKMIFHFLNEKEVVKLIINFKELEDDKNIELANSNEQKSTENIYDREIKHLNTIIKLVKVLNSSTIIAFGLAPFILMGAKYLKTKEFVPILPLYVKYHFFDPYNMIYYGLTYIHQFWSMCICMSSVTGVDMLFCTMCVFIKIH

>HrhoOR2

MHSCRIKPIDAFETSFQWLKLTGYFIAVPNIENPTKALLHNIYRAFTVFILIVYELQHLIFIIQVFGNVEYMIDGLVVLITSLYETIKLFLVNINSRRFEYLNDILNDDMFCAYLPIDEEIMRENKEQLERLSKIIYRTIAVTGAFWLIAPFLNKMSDGHPLLAAYFPFDTNDWFGFTCANIWLTTIIVWVGYGHMSLNILLIGYYSQVKVQLRIIRHHLEHIADDHVNSTEANKYTNYLDTVCCDVEKEFIVLLQRYEKVVRFYQEVELLLDRAMLVQFCGSTGIICAVVYKMTDIPINTTFLYLALYLAGLLLELFIYCYYGTL

>HrhoOR3

MVSKKVSSLVKELEDPNHPLLGPNIKAFYFFGFLQSYHTIQNICYKAWYTIGFFYVVTQWIELWLLKGDLNKALENLSISTLAIMSTTKGVTTVIWQKYWKELLENISTEEKKQINKKDNTTMKLMKNYKNYSRIITYFYWFLVSATVSMVITSPFLRYMYLKSRINDQNNSTISYPEIASSWYPFDKTNILIHSMKCFLDAIMLGQGATVIATYDSTVIVIMIFLKGQMRILQENCKKMFQEKQISNHVVINRIKNCYEHHQFLVRQHNLLNSLLSPIMFMYVLLCSIMICCSVYQLTLEEATTYQRLWFVEYTIAIVFQLFLYCWHSNEVAVESDLLDRGLYESDWWKSDINIRPIFILLAGKLNRIFVLEAGPFTTLSVATFIKIMKGAYSFYTLFTQMQK*

>HrhoOR4

MGLTKIQQENKYHSIIMFVYICGLPHFWYKDVDWSPRKKNLLKFASKFINHIGNLFFITELLAYFTQKELDEQQFSFWFGCAFTHTMCISAIFSLVYHKKNIESLITRMIVTIPNIHHDEEVSEKMIKKCFLYVLTSISTLNLTVLFHGVKASKEYMNGGIFLPVITFWPKTSDLSTAATIGRFVAYIMWWIWVARVSGILTTAIILTICSSHLFNHLQTYFKKMSTIFEENLTIDQKQQKYEASMKVAFKMHHDILNHIEVLIDVCNVTYGGQILMNVSILTIMMFQLASLEHFSIVEILPHIMIMITVLTVTACYMWSLGDVTIESAELSNAIYMSGWENCQNDYSIKMRRLVMIAMTQTQKPLETKTLGLIPVSHESYVSIVKASYSIFSLIFYNNT*

>HrhoOR5a

MCTKASKGVRAAVVRLRVCGFYRLGAGAVAAEAHMAYRALMLILTAIYLLQEVVYAVCERYDMDKLARVMFLLLCHFTSIVKQIVFFVDADRIDNLITLLDEPIFSRGPATLEAAALGAERLGRAYSGTAAVTCMLWTVFPVLNFLQGHHVEFPIWTGSINYNSNSLALLCFTFLYQLRYSLLTFSLLLIEIGGANTNINRMITKSKITYLPISRSYFCYVI*

>HrhoOR5b

MDAFLGTLLHQAKTQLSILRGNYENITERAKIVAQLTKEDYDKVLKRLFVDCLTHFKKVSELLELLQSIFSSAIVVQFTIGGWILCMAAYKIIELNVLSIEFTSMVLFILCILTELFIYCYYGNEVTLESERVAGSVYAAQWVPAPAWFRRALLAALVRARRPLRPLAGRVLPLSLNTFLKILKSSYSFYAVLRQTKNQV*

>HrhoOR6

MRNKIVSYLENEEHPLLGPTLWGLRAWGLYPPINSNWYTIISCSVHLAAIAFVVTQYIELWLIRFNLNLAMRNLSITMLSSICVIKAGTFVLWREDWMKIINFVSSLEKTQLSETYAIKNNIISRYTKYSRCVTYIYWGLVTATVFTVVMAPLAIFLSSPSTNELMRNGTIPYPEIMSSWTPFDRTRGYGYWACVVEHMLICFYGGGIVANYDSNAVVLMSFFAGQLKLLSANCTMLFGDENEVVSYNEALRRIRDCHYHHVQLVKYAKILNSLLSPVLFLYVIICSLMICASAIQLTTEGTTSMQRIWIAEYLMALIAQLFLYCWHGNEVLFMSEKVDDGIYTSTWYLQNNVLRRNVLLLRGQLRKRIIFTAGPFTTLTIAAFVGILKGSYSYYTILNKRDD*

>HrhoOR7

KMLTKTSVKKEFLCDMAFLTTIGSKIFLYPFEGRTGYKIIGYTTVCCLIYITIAQLFLTLFVTGFHDLIDISNIAPNIGVCILTVIKYTKIYTHRKLYHAIITHYQKYMWSIIQMNDIKNLKTIVKYKSVSYFINKILYYYSLPLIIIIVSLPLIVMFYNKKFNDNEFEYLYPFDGWYPFDKVIWFYFIYAWESFMTALVIYTFAFSDMINMSCVAYMCMELSVLSNLLTNLITNEDIEDLKKFKNIFKIHQRIRRSLKIIIKRHQFLNKLAQGLDLALEDVSLINYIFGSVFICLTAFTFTFIDDLYKKIRYFFFFISLNFVILNQSIIGQILSDHSVKLTNAIYSSNWIYADRTTKTTLLYLMMKTQIPFTLTAKGYISMNLNTMTQVTSTSYQYYNLLRCIYHS*

>HrhoOR8

YWPSSNDNSISGDAFRVTTTIVLCILMVTMLSIDCSTMIYLIMYKYKFITLRNFFENLREEFDKNVKENEIVATNLLTDGVIKGIVMHEELLSLSQDIDKAFGTVMACQVCQSSGSAVSLLLQIALADHLTLAGGMKIIFFVAALFFLLALFLCNAGEITYQASLMSDAIFYCGWHASLPQPPQHRDLRRLVSFACARAQRPLVMKAFKMLELTYATFILVLRSTYSVFALFYAQNK*

>HrhoOR9

MVVFAGFTVILFVSITYSIFYTCSIFTRMHFTILKSDFEGIINIKKPFDVQKFNKNFVSLFKRHRLLIQIVDLLELVYNKAILANFISGSILICLCGFNAMEMNNKVAVVPYAFFLMMCLMQMFFLCLCGDMISRAVT*

>HrhoOR10

MLHDRRPLNYFGLHFWLLRFLGIGWWHDPFSEDKRNFPSWYLYYSILMQIVWVAGFVGLETIDPFVGDRDLTQFMFSLAFVVTHDLTIFKLFIFYIKNKDIKDVVRTLEVDLYDYYQQDEKIFATIKKTKILTGAFLFFGWIIIGNTNVHGAIVDIQWKAEVAMLNNSSTKPPRTLPLPIFIPWSYQNDTSYIFTFIFETIGLFWTGHIVMTIDSFIGTLILHMSNQFIMLQDAYRSAYDRTVNRMLQKHNFNDNIDAEIEINKHFLEADKKELIVRKIYTAEHFNSELEKTLKSCYKQHQILIECVQKFATTYSYGFMIQLLSSVTAICALMVQISHDASSLTSSRLVTSLAFFVVMIIQLAIQCFTGNELTYQAGLVSEAVMECKWEHMPVRLGRMLVLCCARAQRPLRLTAAGFTHINIDCFLSIMKAAYSYYAVLSQKQYSN*

>HrhoOR11

MLACAMDVLEKFTNDYKNTFIRYIKMINFIGLDFYLETGESLFKSRKRYYLFLICFIIFFLCQITFIFRSHKTDAKFLDIANAVPCLVLVIQDFVKLLAITTKRKEIKNVIFKINDEWPKEKNYGERSNIIENWTKRNKSFQNVYYGISLFCLCIYELIPLVATLYNRVMGLDTEYFFPFELYYPYKVDSFFVYLVTYFCQAFASSSLHACIYIASDLLITSLLSDVTALFALLQYDLENVTSQLKQTSINQDEEQYNCAVKNIVNRHQRLLGIIKELNGIYGVVIFIFITSSSIICCFFCFLTVVQNGLQSVKNLLAGGAMLGAILVVAFPGQQHYDMSFGVAYAAYCSLWYERNEKFKNLILILIVRSQRASCLSALGFSDVTLATFSKVSF*

>HrhoOR12

MFFLTSAITTCTLWAMIPLFDDAGSRSFPFKIWMPVDPQKSPHYEVGYVYQMITIYISACLFIGVDSTTLSMIMFGCAQIEIIMEKVRQLQPLSNIKLKPYKRKQQIEEKNALFIECIKHHQEVVKYIEKVEDTYHANIFFQLSGTVAIVCIVGLRISIVDKSSVQFYSMVNYMVTMLSQLFLYCWCGNELTIRSQDLREVIYQTPWYDFDRKFCRLLWVAMERMKRPIIFKAGHYIALSRPTFVSILRSSYSYFAVLNQANK*

>HrhoOR13

MASVKSHWLFAPSKDFFEFNLKYLTYLGLWPKEDWSKPQRFLFEIYSMTLSVFEGVFLILTSIGTYNCKNDITALLTNLDKILVVYNFVMKALIFFIKRKQIKILIDEIKHSKDEITMSRNRMMSVHVVVISVLVISIVSAFSLLATYKQEMTIEAWMAFDPLIDKKHLILASLILAVLFVPCACRAMAIQGIVCSILMYLCDQLIELQIRIRALDYRPETHKQMRVDFNEVIKKHVRLIGRFFRYSNTLRAIFKEYFLFQNLAVTVELCFNAMMVTMVGFKEKTLLLTFFAYLSVALLNSYIYCFLADELIVQSQGIALAAYESQWTTWPVELQKDILIIILVAQRPLTLSAGGMATMSIQTFGQTLYNGYSIFAVLSDVVD*

>HrhoOR14

HKVLLKRQHLTMPKNDLTFANNFKLTTFALKVTRCHPDIIRNKFWIFQMLLIIIMNTTVAWILLNSVVFHDIKSGDFAEVSKNVAMFIICVTISFKLYIIVRECKFLIETMNNMNQDYRDSPENERNIIIKYTKRGAAVSKFWLAAASATSCIYPAKAAVAMVHAYLNGEFHLVPMFDVAFPENLNEKKHNLEISLPFFTLCLTFALYAMTVYVCFDPLAPIFMLHVCGQIEIINTKIAQVFCNKDLTRENFKTIVIKLQELYKFIRCIEKKFTIVYEFVMKTTTILLPLSAFQIVQSVQRREVNLEFISFFSAAMLHFWLPCYYSNQLMEKGEELRSAIYSCKWECSNDLLARKMVLLMLVRATNPLVISSVFYTIKLETFTQMCRDAYAIFSIMNAAWS*

>HrhoOR15

MDKPMLVQFCGSTGIVCAVVYKMTGIPFNTTFIYLALYLGCLLLELYIYCYYGTLLMNESLLVNDSIYLSNWTSLSPRFRRIILIAMTRWSRPMTPTAAGLVSISLKTFVSVLRLSYSIYTIIKS*

>HrhoOR16

MNDPIKSKYILRKITTFAYISGIPYLWYEELGWPKQLTNFHDKITNILTVLLCIFMSLEILALFTQKNMNNQQSSDALKYAISQPIFFMNFFSFVYYKEEVRILFYNLTNSMSTYHRDEEVERKLVKKIKFYVSAFISVAIAASMSAGLDGLWRVLEKDDTFTTVITAWPDVDDRSLAAGIGRIIAFIMWCIHTVRFVGGVTIVVALTVCVCHQYKYLQSYFYSLDKIFEINCSQSVKEEEYEKGLLIGVKLHNNIIRYTQDLCNVCNIAYGGQIVVNVTVLVILMVQMQNGNRQFMLVLTCAIMVSALLVLNGFYMWNLGDITVEASEVCTAMYMSGWENCTRRSSVRVRKILMVAMTQAQKEVTIKTLMILQVSYASYVSIVKFSYSVFSLIY*

>HrhoOR17

SLFDHKGSTRFTYTTLSVFVPRTNVRMESLGRIDCFKFNINFWKFLGVWPETKSCYYAFYSKIFTFAFTFLYVILPTVNLAYIPPVMDIFVEEMMFYFTEVIGLFKVLTMLLTHEKIVNILKVLQSDLFHPESSESVEIINKAKIFIVKYWKFIATVSVTSNLTHVLSPLIVHLLLSVELQLPLCNYFFLPKNIKDTFIYPLYLYQCFGIHMQMWYNVNFDSFILGLMILVIAQLEILDLKLRTITDRCAKLYIKRKQNDTEVLTKLNQALQHYNELGRFCNLIQDVFSIALFMQFGIASCIICVCLFRFTLPAPWQYYIFLGTYILVMIFQILIPCWFGTRIIEKSQLLKFSVYSCDWTCQSRIFRSSLKIFSERANRPITLIAGKMFALSLGTFTSIMNSAYSFFTLLRHMQTRDDMD*

>HrhoOR18

MAHVNNEDMYLNRAKFVMKILGVWMPLENETRFKKIYRTAMMFLQYIFLIFQTIFIFQVWGDLEAVSEASYLLFTQACLCFKITVFQIKIPMLRDLLKQMNADIFRPQTLEHINILILQATRIKRFLLAFMVSSQITCGMWALKPLFDNAGKREFPFDMWMPVSSEYSPQYELGYGFQLITICMSAYMYFGVDSVALSMVIFGCAQIDIVKAKILSIKSISNSVGTTKEHTRRIRKENYNKIIECITQHQAVLKFIQMTENTYHAYLFFQLTGSVGLICMSALRLVVVKFPSIHFFSIVFYVSVMISQLFICCWCGHELTATSEELFATLYQCLWYEQDFKFKRDLRFMMMCVRRPVVLKVGNYITLSRQTFVSILRMSYSYFAVLNSTEK*

>HrhoOR19

LDRRHDAMPLLKKIKTYFDKEGFDYSKNYINPYEFHSTFYFFMKYFKVIDDEPAPKWANVVQVLIGAVGFTNLCLSILMCAINSVKPFTLPKFIEGGTYIIVVFYGISIHLCSIFNKSGYHCLLRMLRQDFDFICTRGQTYRKKFFENHLIIWKLSIVSIIFTQSIAIGMIAFSIILLSYYMATHEPGDGTSRPLLIPFWIFNLDLNKSPIYEILFNYSHLAQLCYGFTYVFLVQTQIVWIKHIETKADIVIWLLNDLFDNLTYPNTEEEKKVCDTEIKNRMCFIIRQHQSVYTLLESYAAVYRKLLMFEQKLCGPVVCFASYCIVLQLEAGEFNGVLLLLCIGALTLTYIPCYLCTTLSEKIMSVSDECMNIPFWNANPKIIRPYLVLMIRRSLRPLPLKVPGFQPHTLQTFSKSMVSAYSLFNMLRQANVQ*

>HrhoOR20

LLDNFNKDFIFICSLGTSYRTSFLNSQLIVWKLFIIWITFCITISCAFVLNTVVYLMYQTLFMTITEDTVRPLIFPLWLPTGDDPYRSPNYEMFFVFEILGTALIPTAFAVYTYNLFHLLLHVYNLMDVMIKAISELFLGLNPDVADLPARNPERQITQAILKTKIKQIVKWHKSVYKYMSTISSIYGPVLVYQVMFSAIAICVMAIQIANALDKGKIDFLFCMLESAALLQLWITCYIGTMIRNKAFAVGDACYNSGWENSKFGCWLRSDIVLIIMRSQIPVSIKFPLLPQIELETFSSITSTSYSYFNMLRRAT*

>HrhoOR21

MKRLKHKHIITSINSIRFLFELNGLKLEDTNSSDNLKYRIIYAFNFTWLNFDGIGELIWFCDGIWSSEHFVKLAAMFPCVIMCFLSNIKMILHNLNEKKVADLINSFKELEDDEFINDTDADDRSKEKIYEEEMKYLEKIVKIVKVLNVVTVIAFGIAPFLLMGAHYLETKEFMPVLPFYVKYYLFDPYNMKYYGLLYLHQFWSMCICLIGILGVDMLFCTMCVFIKIHFKLLEYDFERFIPIHTTPHGCLRENETITRRFKWLVKKHQKVISCSNLLNRIHSNEFMMNFFTSSFLICLSAFIITVVEEMRFRISFLSFLVTSLQQLFLLCFFGDMVMTCSINLSGSIYSSLWHSVKCNIGKQLSYALQRSQKPCKITAGGFIDVNLIVFTQIIGKTWTLFALLRTIFNP*

>HrhoOR22

MLNDEGSFDVNEKFKPFHETYKIFTYIMTMGLIYPNPKTEKIRLKLILFAILFVSPLLFLIGYDVYKCCLRHDIVNIIRHSTVAGPIVFILLKIMSFYYNRDLVKELIDEINRDHVRYNKLPTKYQDIVEKSLRYHKTTEKRWVACVSISSFLFVIMATVFTIYSQIFDAEPMKYMIHEIDAPTIESIIGWPYYEIMFVYESYVSIYFVLNFSGFDGFFGVVINHACLKIKIFCNAFSDALKESNEDEIMRLIHEIIRDQCKMFSFVNTILAVFSSWFVCILIVALALICNCMYLVIQGHGFDIRYIVFTIATIIHIFMPCWYASKLKSMSQESSTMAYFSGWEDVPIPRVRRTLMFFMARGQVPLQIEALNIIKFDMELFVSIMRTSYTMLTLLQSSS*

>HrhoOR23

MKNSECLSASIAILKVTGVWWSDSMVYKIIGSLIQLFLYVFTVLAEIAYVLMVLGDTERTVDAAVLLLSHLVQGVKVATVWFRQRRIKGLIKLIDGPNFEKTDLMKAKMIESFGALLKLSGHLFLSTAAVTALFWVIVPMLKSEITLPLKTAYPFDINDPGSFTLMYGYTTISVVLVGVGDAAENYLLAALLILPTIHLEILCQELQELDHGDDIYERTVSCIKYHQHIIEYANEVASVFGIIMFCQFVTSSVIICMTLFKITITTEPIEMITMVFYLVCVCLELFLYCYAGDLLMNKSLLVSEASFPGKWLKDTRSCRALLMTTVRAQRPLIVKAGGVFTVSLPTAAAIMQTAYSYYAVLQQKTKEHN*

>HrhoOR24

MSSSRRQDDPQNTSSIIISIIMQSIQFIGVWSSVGYKRSIANFATICFILTIGAQVINLVLERNDSEKMMEAFSVFSVCLMGLLKYISLRRNSTAWQYLLSRVSQIENEKMNENNDNLLDYETDDDNKETVPSVKHVYTYNDKAKFISTVLTRFYTLTVFMFVSTPIFEYFWKTYKNHEPMKLPHILPGWTPQDDFHFCAYFITVACEAIAAVYCVRIHVTFDVTFVTLMIFSCGQFNYLWVKSERIGGSGNNCQLSIKRDKRANFRIMQCHKSHIMLVDLVTRLNKLLKIILGVYFTVITLTLCTVAVRLRASDKLGLVKLILLLQYMATNLMQLYLYCRYGDALLNQSSINMGEGPFGAAWWALSPPTRRHLSLLAAGMSRQQYMSSGIYIDNLPAFLQVT*

>HrhoOR25

LTTLNKMTIFIRNVNLSISVSLTVLKLVGFWAPNDIKNNFNIIYYLYAIVTFMILLGIYLIIQVVDMFLIWGDLPLMTGTAFLLFTNLAQAIKILNLVWKRQRVEEIIFNANQLLRDQRTEEGKNIVKKCDRETTLQQLLYFCLTTITVAGWAGSAEKNKLPLRAWYPYDTSKSPAYEITYVHQVGALFVAAYLNVGKDTLVTALIAQCRCRLKLVGLALRNLNEELKADDKHIFNAEQQFVVRRRLNRCVVQHQSALEATVQLQDCFSVPTFAQFTVSMVIICVTAFQLASQTGNLVRVFSMGTYLLNMTFQVFLYCYQGNQLSEESMEIAGAAYESPWYTFSSSTRRSILVLMSRSRRPARLKAGGFTTLSLASYMAIIKASYSFFTVLQQVDENK*

>HrhoOR26

MDDINSDKIPNYTHYIILPLKLVGCWDWYKNPEKEYQIIINNGYYALVLFVLMNLQWSLTVNLYTEWTTIMDNLEKLADSLPLLVSLAIIIHLASNKKKMYELVDFMNNNFKYHSARGLTNMTMRESYTTAKKFGYVYTACTLFSITVYVCMPLLSYAWTKQPLQYWVYVDVSSVSDFVIVFIRQCVGQIFVGLAVGQLGVFFASNAILICGQLDLLCCSLRNARYTALLQNDVHHRDIVADHGDIVNDEKHSYVYNKAVLQDSDYHYDEKMKPTLSRTDFDIYDAEFDVATITAYRECAKMSQVILQYKRDFELLVSPLLVLRVVQVTLYLCTLLYAASLKFDMVTVEYLAAVALDIYIYCYYGNQIIIQADRVTCAAYQSAWPAAGARARGLLLRVLAAHCPRRPVAVRAGGFLTMDLHTFVVIIKTSFSYYTLLVNVNEK*

>HrhoOR27

MYKWLALIPLFNGAICICIVLVVISKEIDWHFVTHILPLFGEIFVYSYFGEQIKTKAKNIELALLSFDWCNMNKEDKINYIIVFTYMQKQFGIEVASNKDLCMVTLTAVLKLTYQAYTVVQSIDF*

>HrhoOR28

CVFIKIHFKLLQNEFERFIPTQTKSRCYLTENETIKKRFKRLVKKHKKVISCAKLLDKIHSNELMLNFVTSSFLLCFSAFTMSIAEDVRFRISFLTFLLAGLEQLYLLCYFGDMVMT

>HrhoOR29

ETLQTFHSILSFAGIPIYAKTNWDSKIRLTHQIFNVFIGFLTFIFTTVFVIINYSDLLLCIQGACIWTTGIIMFISLGVCLIFRRKFRMFLTEMGFKDTMLEMPLVAHVMSLELEDGQKLKELKLKVTESQERLLKLTRDLLKLYVASVWLCATLYICSPIYFMITGEEKSPRLLAFDMWFPWSFDNLNVYVASFVFHAYAGYLCCIAYPGLQLTISLLVGQIVRQLKITSFIMFHLEEIATELSKGRLGNKQMYCTNILTQCVDHYIKMKRFSNNLNVICQPFYLALILVATMLVCVCSVKIAISDKLSLDTMKYYVHESCFILVVYMFCLLGQQVDNECENLERAVTEKWYKFNKTHKINVKIFKMAVNQRMPIYIFGSMKLSLPTFTWFIRNGMSFFTLVMSVLED*

>HrhoOR30

MELDFERIYKLSVNLLKFNRFYPFYKIDMKWIVQVLFLYSISLLIFLALMQSSVYYIKINELSDVCDNGVFSLAFLGLTFMYGTVIWHKNDLIYLIESVQKDYDESKDLSQTEINFILDYIEKGKRVVHLWAFVSIFNVFFIPSRILVVMVSEGKYNLVDVLDSFHPNILEKSTSDIWIFFLELLIRLYYSIYANIMYVGFSPLGPIFMAHACGQLEIVMTRIKSIFTERNYDEREAKIKLIDVVQRMQRIYRFVDSINNTCELYYQITLNSTSLMLPLIVYMVIKDFQMDKVFQYITFIFGSFLMTFIPCSYSTLLLAKGDEMRESIYMSGWERHLDRDARATIIIVLTRASRPISIHTLFKTINLDAFTDVNIDFFINQSATIFIPDHRPLATPS*

>HrhoOR31

MKIMNNNMNGKLAFLLPFLPLSDSETWDKLDPKLYHGVHIYWLKIYGLWYYSFSPKTFKFWLQLAYTAIVLWLVCFLPGIGEIVYLLKRRDNIGDVAGGLYLFLSEMYTYFKLAVFWLNKQKITRLLQYLYCDEFKPKELEHKDIILKSIKRARFVMSAYSTMCVCAVSVGIVMPLTENFDVLPTNVEYDQFDVYKSPAYGILYSHHIYYKPATCIIDGVMDTILAAFVASAIGQIEILSFNLRNFDKLAYRLRTRAIIAKENLYPDQYYIQVTMKECIKHHNSIIRYVSMIEEAFSLASALQFMLSVMVLCLVGIQFLSIEDPRSHLMQIVWMAIYLWCMLVEVFILCWFGDELIWKSQSVRQAAFEGPWLNVDVKTAKYIIIFLERSKRPLRVTAGKIFTLSLDTYTILINWAYKAFAVMSNMKK*

>HrhoOR32

MENKIKPLDAFKMLFRTLTFTAYFMPVPDIENPVKKKWHRYYRIATVIILLIYDLQHITFVILVFGDVDRMIEGLSVLLTILNVTYKLITVNLNEKRFNKLHNVLEDDIFSAKCPKHEELMTKNKEELDGISKTINRTVTVIAVCWLLTPFLKKLSDEEVILPAYFPFPTDDWISFSCASIWITFVIIWVGYGHMTLNILIVGYYSQVKVQLSIVRYSLEHLADDDEGIPHEVFTCRNHGYKDNQSKLYQEKLVALIKRYDKAVWFSKEMESIMNKALLVQFSGSTGIVCTVVYKMTGVSNQYIIKNL*

>HrhoOR33

MVVALFCFMPITLMAVDYYKNGKYKVNFPFLVKYFFDPFTEIWPYVYFHQVVSTFIVWVNVYGPDTFFYAFCVYVQMHFRILSQRLRKLFYKPNLLVEDKEKLIKLLKRHQELIQLVKDFETLYTSSNLWNMVISSILICLSAFNATTNPDAKAVLTFICFLFMSLSQISILCFFGDMIVNSSALVAEAAYSCGWYNVDADVKKSLLIVIMRAHTPCKLTAANFAVLNLRAFAMIISKSWSYFALLKTLYK*

>HrhoOR34

KEVAEMISRFLETFEDPKRPLLAPNYWILNKVGLLLPDSKLGKFFFIIIHEIATLFVLTQYIELYVIRSDFDLVLTNLRISMLSTVCIVKSNTLLLNQSKWKQIIDYVTTADIFERENRVPDKKNMLDSCTKYCRSVTYFYWVLVFSTVMTTTSTPLVRVMSSLSYRDELRNGTELFPHIFSSWMPFDKYHSPGLWITVGWHIIICVYGATILGAYDTTVIVMMEFFGIKLELLRARCQMMFGKDESGISDDKATKIIQQLHTIHVKLLEHSRLLNSILSPVMFVYVIICAFMLCTSAYQLTTATSTTQKVFMAEYLIFGIAQLFMFCWHSNKFLVKSQEGMFGPYESNWWAAGIKQKKLILMLTEQLKLVHIFSAGPFTNLTVSTFLGILKGAYSYYTLLRK*

>HrhoOR35

MMCNVSTDSFMAGIVTILIAQTKVLNYKLQNLKVRNENFVEETHVQHKIFTVILKKYLKHYGLIMECYIKIQEILSLAMFVQFTMASAIICVTLCGLYLGPTMETLIFLVTYLIIIILQIFIPSWLGTQFSHECNKLAVAAYNCDWISQSEDFKKSLNLFILKANATVILKGLKIFPLSLETFVSIMKTAYSFFALVQNVQAR*

>HrhoOR36

MTLQYDMAKIKKKETETKFKSFHETYSMCAFALAIGLMYPNSENRRKRLIYMIIFTTANVPQLYWLTINTLQTLKDSDFYNFSRHITISVVVLLFLFKTVYAIIMCDMYKKLLNQITDDMNKGNELDDSYKAIYKQYIKEAKFGQICWVFIPIAMSFQFPAYAAICTIYESIISDVGPKCMIHNLDLSFMGDQYNISPYFEIMFVYNAIQTIALVPNFTGFDGSFCIVTSHLRLNLKLLSHKLKRIFEDSKSNLELRKNIKTCVIEHQEILRFYDAIQVFYAPWLMTVFLLTSVLISFNLYRMHLDQKIDLKYSFFALSGVIHMLAPCYFSSKLIEAGEEVAIEMYSVKWQRWNDNKVTKVLIFMIARAQKEFVLVGAGIILFNMNLFLSLMRTSYSVFTLLCTR*

>HrhoOR37

MFDSIRNYIAYFKNRIKDNNFDSLLWIVNGAPSIVGFNLRKDKIWAPFFVIHMSLLTYVYGVGNVMYQVKYAKNTGDFIESYVNITIMILAAVSGYWFIVYRPPLRVILNLAEENDRLSKTSPIVKKKREKLLATIKIIVFIFYGCNLTNATFVYLPHRVDILSHYAMQPCVGLEPLTSSPNREICLTILCAQELSIMTVVLNYQALWLVLVAHTAVMYQVLSEEMLIVNTDEEILEDKLLSFIHRHNVILDITHRLKEIYSMPIGMNLGVNAICMCLFFFMPLSDWFKFMPILVYCFVVFFLNCFLCQRLINASEEFERAVYGCGWENFDVIKKKWVYIMLMNSQKPAQLLAADIIPVNIATFATTMQSMYKFITVFKL*

>HrhoOR38

MENTNSPERSVIQYVRSSRAFRQFKNPPQPHMCIQDTLKDTTEKMFINVLGWQKIANPKQYSDPIPLYGGMQVPQGCGPNSNKPPLLVFAVMVNPDILKANGKNATNPTDRDALVNLLCDFVEAMNPGLALARKPVILRDRDLAGELKDVWLAVQKKRDKEKEGNQEVMYKVYDIDGVGNDEVNDDERQANLRYNQGDGGSPTKVQNNRKNAVKSSKQILMNAGQKSEFDLGMNNCQLNQNHSNRDNKCATDTTYCTPVYEQIVSYSENHNQINDIQEKFTKHDANASTSFTAEWDALHGKPSDGWEDFSKRNINSIKSNEADTRQRCSKTENGKLVNKTQYNFFPVFNKTEADSSIDNVSNEQNLRIEEKAKIILDPMQKLVLHSTDNKICDNNSSALSSLSS*

>HrhoOR39

MSSLSSHRAIKLFRRMCCYAYVTGLPNFWYEKPNWSIHVGKFHDFISHVTDVINCVFYLGQFFSFFTQKNLNERQETDQIIFTTINPCIYWAPIAMNYYKEQVRDLIRNLVLVLPSVYNDREVERKMVKKSCLYVSLLLSTANATLLLYGIDSFIKVLKGAVFTTVITAWPSVEDRSRIAGIGRAAVFIIWTQFMFRSCGAISLIISLTINTSHQYIQLQSYFRNLSNIFQENLIQEEMETKYEEHLKIGIQQHIKILSYTKKLKQACILVYGGQIFTNMVILVMTMMVMMGDDLSLTKLMTFMTLAISSTVTNGFYMCTIGDITVEVQRYGSQ*

>HrhoOR40

MTVWPDLKDESFKAGAFRIATYIAWWILMFRLSGAIVLVMSLMTYTSYQFKQLQSYFITLANIFQQDLSQLEKERKYEEALKIGIKLHVDVISVTRKLVTTCNVSYGGEIIVNVIVIATIMIRLANEDRNLTNILASVQIALTVLGITGFYMWTLGDITLEAD

>HrhoOR41

MTGAKNVKNRVEAWNVNKRQNRINCANANPVHSEATFQEALRATLIIGQIFSLIPVDRVSSKNTANVKFSWTSWKCFYLVLSLGGQVFMTAMCLNKLFDRDTSLKATTSVIFYTMTTVTMLMFFQIARNWPCLVQQIANTEQMDPNFDRNLTFKCNITCAIVLTLALTEHILSLLSAFAGALTCYPNMSLYEGFVKYFYPWVFNFLPYTVPLGILTQFFHFQSTFIWNFSDLFVINMSYYLTSRLQQINNKLLSVQGKYLPESFWRVTREDYSRATQLVRRVDDVISGIVFISFANNLFFICLQLFNTLEDGIQRTEACRRRLGRTGPLGGHEAETYFLFSLGYLIARSVAVSLIASQINMAASVPAPVLYDVPSPVYCIEVQRFVDQVNGGSVALSGLQFFNVTRGLLLSVAGTIVTYELVMFQFNSSGNSNSTEVAANATIS*

>HrhoOR42

MFIDGELKDGSEQLEQLNKKKKTKSNAKGTNQQEAPKSMLVSLFVPFENSMPESVEYMECDGSIHFSGVVSSSVFMYPKATVNEAIASVKQDIVRSLASRFTMHCDALIDDNLLPEEKVCFNEPPRRVLVPVGALHLCDYLFPGEAPAEALLSVRELLDLHITEADVVCDIETPADTSEFDALDRDTSSEELLASPQEASQFMYITGICFAMLVLFISIIIHYYDGITKFISGMFSKAT*

>HrhoOR43

MPRSQSIRNTEWFNVTFILMAAVGIWEPPCSENKIIVKYLYLIYRLIFLSLFAFAIISMQLFLFFLVLGDMDALIEASVLFFCNIIHGIKMITIIIQRKRIKSLLTIVDDDVDNHKVYENLGKRAGFMSNMFYLNVAATGILWSIYPMTKSELKLPYSCPLISKDSYWFTYFYVY

>SlitOR5

MTNQKDLDFENIFKITTTALHISGSHPSVPKDLKWALKFTILHGTFALGFVTVIYSIIYHDLKEQNFVQICKDCVVFVLFCVTSLQYCVLLIHQDNLVLLIKNINSDYEQMQNLSDKEKQLMDKYMNLGAKVCRHWFVLVLVTCLIFLIKSVGFMCYYYLINDIKYIPYYDIKYPEFIEERKNENFSVFLITYCLMFYFALYSVLIYVAYVPLGPVFMLHASGLLEVVKNRIDDLFSDSNPEKIREKLKDVVMKLQYIYSIVDDMKTIFRFGYEVSLKGTAVLLPVTFYAVLEAAKNGEISMEFISFIAGGIVISAVPCYYSDLLMEKGEAVRLSLYMCGWEQHYDRRTRTTLQLMLLRALRPIAIQTLFRTLCLDALTDLFQQSYGIFNLMNAMWS

>MsexOR83

MYLNFDEFYRWPILAMKLNRSHPHIKRDKKWFMQFFILHGMFTIIFLLIIYCVIFHDLKNNDFSATCRNGCLSVMYFVVSFNYAVMLANQDLLTSMFEIMKADYKETQSFITEEQKVVLKYAEQSKWVCTQWLAISIAGVVLFPLRNFILYAYYYYKDDFKLVPVYDMTFPRIIEENKESNFFVYLLTYFLIMYFGLYGSFMYAAFVPIGPIFMLHSCARLEVIIKRVKDLFILNNEEEANEKLKDIIQEIQYVYSFVEKTKICFRVVYELTMKATTIILPIAIYEVIKAFHKGEIRVELGAFIFGGVLISSSPCFYSDLLMEKGEELRLAVYSSRWEEASSSRARSTLLVILLRALQPIAIRTMFRTVCLDALTDLFQQSYAIFNLMCAMWG

>DpunOR20

MEEIDFDNIFKIQMTGMKIIQCHPDTKHDIYWLLKTSIIFGLYTLEFYFVGNSMLVDIRNKDLLNAFQSAVPVSFYIFVMINYSFLINNSKSIGQMFKIMKSDYEKMPNLDQRCRSVIIDTTTKCSWILKYWSRLTTSCVVVFCLKSICLSIYYILKDQYEFVPLHEMHYPEFIEKHKKNNIYVFAFTYVAEFYFISLTILVYVCSTPLGPLFMLHACSQLELVKIKFEDIFKKDDVEKRLSDIVEDLQLAYGFIAEINKSFTIIYEIMMKEFSVRCPVTCYAFVKAFSHREFAVEHVIVIVATMIFTAMPCYYSDLLMSKGEEVRQAAYDCGWERVFNPGARKTLLIIVNRASNPTAVKSIFCTINLDTLTNVFRQAYTIFNLMCAMWD

>DpunOR4

MEGIDFEKFFKIQTIGMKMALCHPDTKHDIYWLLKLILLYGPYTLNFFFLCNSVLIDIRKQDYFNAFRNCVPVTIYICIVFEYIMLIKHRKTIGKLIALMRDDFDKIPNLETRSKSAVFKHINNSTWIMQNFLVLMFCSVWIFIIKSIILSIYYAVTSEFRLVPFHDMYYTDYIDRNRDSNLFIFAFTYVMGINYTCCCTMIYLCALPLGPIFMLHVCGLLELIIIKFENVFEKDNVNERLIEIVKELQYAYGFVADINTCFTFMYEVMLKQTMILLPVISYAIVKSIGHGEFTIEYITVFMAIISMSTPCYYSDKLMSKGEEVRQAAYACGWERVFIPEARKTLLIILTRALKPITIKSIFHNINLDTLADVYRQAYTIFNLMSTMWN

>DpunOR45

MVELNFEKIFKLVMICMKISQCHPETKINKYWVLRSIMIFGVYTLSFLGLSNCIRYYLIGNDYFNAFKNGVPIIYYVCMMLNFGIFIKKRFQMRNLIESMKSDYSKACAMDKRSKKIVQGFAAKGRLITIFWGYLMVGTLCTFIVKSIFLTIYHSKRSGELRLTSFYEVYYPFNISELRVYNTWVYVPVYLFEVYFTGLTELLILWSTTLGPIFMLHACGQLELVKIQFDNIFENDNVDENLNRIVKRLQYIYSFVREINECFTLMYEVMLKQSVLLLPFCSFALIQSIKRRQVTVEFSGVLLQSVITSSIPCYYGDLLLQKGEDLRFAAYNCGWERVHKPKACKTLLIIMTRATRPVAIESIFSSICLDTLTNVFSQAYTIFNLCLQFGE

>DpunOR46

MEELDFEKLFKLQMVCMKINLCHPDTKINKYWVLKNMIAFTPYTISISLLINCITYCMRRNDYFNAFKSGVPVIFWVSMKLNFFFLIKKRSKLRYLIDCMKDDYSKALAMDNKRREVIEEYSDRGRKVLLFWGYLMVCTVGMFLLKSMFLTVYHSKRNGELRLYSFFDMHYPFNIRELRVYNKLYFFVSYFIEMYFTSKSALIFFCTIPLGPIFMLHICGQLELVKMKFEKVFENDNTDDILKDIVQKLQYIYSFVGEINDCLMFIYEVMLKQNILLLPFTSHAIIQSIKRGEITAEFLGILLQSIITSSIPCYYGDLLMQKGEELRDAAYNCGWECVHKPKARKTLLIIMTRALRPVAIQSIFSTICLDTLTEVYSQGYTIFNLMSAVID

>DpunOR47

MAIEFETMFNIPTLALELNLMHPQIERNYKWILKFLITYGFYTLIFLNIIYYNIYPNLKNGEFIKACQTALLAVVYLVTLFRYCVIFWKGETILKLINLMKEDFQTGDTSIEEEIIVENFAKKGRSISRHFLVYTAYTVLVFPVKHFCLEIYYFVIGDFQILPPYDITYPSFIEDVKFEFIPYVLLYLVFLYYLIYSGVVFVAFEALGLICIVHACGQLELAKRDISSLFIDSDEEAIARRMKIFTLRMQRVYMFVDKVNGTFRLAYELNLKAIVIVLPLTSYAVLESFRNGAINVEFLSILFGGIVSSGVPCYFSDLLKQKSEEVRQAIYACGWESQHSLSQRSALLIALTRTLRPVAINTIFSTLCLETLADVLKQAYTIFNLMNATM

>DpunOR63

MVELNFEKIFKLVMICMKISQSHPETKINKYWALTSILILSPYTLSFLSLINCTQYYLTNDDFFNAFRNGVPIVYYTCMMLNFVILIKKRFELRNLIECTKSDYSKACGMDERRKHIIQGYAVTGRRVSLFLGYLLIISVSLFMAKTIFLTIYHSKRSGELRLTSFYEVYYPFNISELRVYNTWVYVPVYLFEVYFTGLTELLILWSTTLGPIFMLHACGQLELVKIQFDNIFENDNVDEN

>DpunOR68

MVELDFEKLFKLLMICMKFSLCHPETKINKYWVLTSIMAFGPYTVSFLVVINCISYYLTRDDYFNAFRNGVPIVYYISMMLYFGIFIKKRFHLRNLIECIKRDYSKACAMDKRSKKIVQEYAAKGRIITIFWGYLMVSTVGIFVVKSIFLTIYHSKRNGELRLTSFYEVYYPFNISELRVSNTWVYVPIYFLEVYFTWMTELLFLCSTTLGPMFMLHACGQLELVKIKFKHIFENDYVDENLNKIVQHLQYIYSFVREINECFTFMYELIIKQSVLLLPFTSYAIIQSIKREEITVEFGGVLIQSALTISIPCYYGDWLLQKVTFLNSYYTAISLSYHSQLRNGCTSA

>DpunOR7

MKFMQCHPNTKLDKYWVLKILIGVGTYTLYFMLLINCTKWYINKNDYYNAFRNVVPVMFYVCMMLYYCLLIKHQTTLDNLINSMKNDYAKACDMDIRRKTIIHNNAAKGKEIMKIWCVLMIATVLVFIVKSMCLTIYQSKRHGELKLIPFYDMHYPLNLTERREHDNMIFAVTFLAEMNFTTICVSIFFCSVPLGPIFMLHACGQLELIIVKFENVFETDNVDGNLNDIVQNLQYIYSFVSEINKCFTLTYEVMLKQNMLLLPCTAYAIIQSISLEEVRLAAYNCGWERVHKPNAR

**The amino acid sequences of IRs used for building phylogenetic trees are listed below:**

>HrhoIR8a

MDFFVLFFILMIVNVIFVTSEISLRFVFILEHHDYELAEQIGNALKTVEESTPGVHLSDAVIFLNREEDGESYRKLCSSVSTGVSMIINLSWAPWPAAEDMASSSGVPIIHTALGSQQLIKALDDYLESRNASDAAYILESEKDVDKTLYELLGRSNVRVWVHAGLTRDSANVLKSMRPEPSFHVVVGNKGFIMDTYRRAVKEKLVRRKYRWNLVFTDYSGADMDWSQVTLPAMVLYINPDECCKLMKQEKCTCPPDFQRTQSMLSYLIEYIVTSYGKLEDQQFTTKLDCNAIEMGDMNVTKEKLLDFFNQDSTNNDSLFYWNIERSGLFLRSRFVLSYSDGSERLELVAKWSADEEYKLLPGVTLEPLRMFFRIGTSAAIPWTLHKMDPNGQPMVTDEGDPVYEGYCIDLIQKLSEVMEFDYEIVTPKSGSFGRKLPNGTWDGLIGDLARGETEIAVAALTMTAEREEVIDFVAPYFDQTGIIIVIRKPIRKTSLFKFMTVLRTEVWLSIIAALILTGFMLWLLDKYSPYSARNNPDAYPYPCREFTLKESFWFALTSFTPQGGGEAPKALSGRTLVAAYWLFVVLMLATFTANLAAFLTVERMQTPVSSLEQLARQSRINYTVVEGSSIHQYFINMKFAEDTLYRVWKEITLNATSDQAQYRVWDYPIREQYGHILLAINASGPVPDARTGFRQVEEHLDADFAFIHDSAEIKYEVTKNCNLTEVGEVFAEQPYAIAVQQGSRLQEDLTRALLDLQKERFFEQLNSKYWNESLRQSCPDADESEGITLESLGGVFIATLFGLGLSMITLAWEVFYYKRKQKTNVHDTTTSEKPRPAFVKKGKLRRRKKTVTIGDSFKPAVDVSHITVYPKGYVP*

>HrhoIR21a

MFWYVFGTFTNCFTFVGKNSWGKTTKNTTRLLIGWYWVFTIIITSCYTGSIIAFVTLPIFPATVDSIKQLLSGFYRIGTLDRGGWERWFINSSDPDTNKLFKKIELVSTIQAGIKNTTKAFFFPYAFLGSQAELEYIVQANYTKTRSKRAVLHISNECFVPFGVALSFPNNSIYTSRFSNDIRRVLQSGILKKIVSDVKWEMQRSSSGKLLSVGSAILKSTSIEEKGLTLEDTQGMFLLLGAGFLIAAGALLSEWMGGISRRCCVIKKKATSANSSRNLMTPFNTENEVKDDTDGFDLERKSDLDSNNSSADSRKTLDGHIIKLTENSITVHENFNSNDWNCRRSSSVDIDQEVKEIFEKDISRRRKAFDDRSSLQDGRLTTASKGKFGEYIP*

>HrhoIR76b

MVANGRAVFRSFTSDRDFLPTVKAGAVLVKEQTAVDHLMYFDYLTKVREGVVEEERCTYVVAPNAFMKRTRAFAFPMNTNLTTLFDPILTYLLQSGIVDFLEHRDLPTTKICPLDLQSKDRRLRNSDLMMTYMIMGVGLASAIAVFIIEMILKRYAVKHKLKPLKKFNSKTFTFKDDSMPPPYDSLFGKNSKYRGSKRTVVNGREYWETKMKDGTTRLIPLRTPSALLYQ*

>HrhoIR41a

MLTSKMLSIPIEILLKIILQKYFINSYCITVVSEDSIQLKTSIPFIYAIPNDNFVDLLLNSSDIGCSDYIVNMKNPQEFMKAFEKVTHLGLLRKSDRKILILTHSKSYNAQDKDAILKVLSMNETRFVANILLVIQADVNEKCYIYDLITHQYVGKDDVRKPIYLNQWNSCTGFTNNVSLFPHYNMSDLYGKTLKLACFNYEPYSLLDLDTSVDPLGRDGMEVRVMDEFCRWVNCTIELVRDDNQWGEIYSYENLTGVGVIGNVVKDEADVGISALYSWYEEYIALDFSTPLVRTAVTCIAPAARVLASWELPLLPFSLHMWLGLGFTFFYASMALMIAKGFNTDKMFLTTFGMMITQVRFLNSCKKSGGYHSPH*

>HrhoIR60a

MTMVVFLFISLLINEVSLINPNGPTAVEDFTNCITSIVKVSFKNPGLLVFVDTFFIAEAVGRIKGNVLKQIHLNKKFSVRVVRPKNEYPVCVNLNEFNTGVVHQNQVDVIPLADYFVIIVDSYSEFTHAASRLIRLRNWNPHGKFLILLYSFDNIYYLKQIEYIFTCLFRYNVLNVVVLVPHIRNIRATIIYTWEPFEPPKYCGYYNETAENRIKVADFCEKGHLKNNTTLFENVVPIDMMSCVVNILAIEKQPFIGKDDNVQEANIERFLINEVLSTINMKTNYIITNKSRGERFYNEWNGALKKIVSKKFNVLLGGIFPDFDVHEDFQCSNTYLEDSYTWVVPRAHPRPPWVALTIIFHKTVWLSVLIGFTISALSWKFLSTVSGDSTYYTSIDHCLLSTWLCILGLTTHIRPKKESLRIFFVFFNIYCIIFITAYQTKLFDVLTNPSFEYQIANVEELIDSGLKFGGFEELHDLFYNSSDPFDNLIGSQWVIVENMSNAMVDVVVHRNFSVLCSRLELTYLSATMPQLSDSIGHHKYYAFKTNVFTVPIELIAMRGYALVEKFSEILEAFKQSGIVSGIRRHYVTFAERKRASIILKLQSQQNDVRALTIQHLQGGFLALVLGYVGGIIVFIVELIIKCNLVQKVLYQK*

>HrhoIR75q.2

MKAVNLLALFIITVISCLAEADLVSIIGDLIRVMNKPSSVIATLCWPQYKQLKLYYFLHRENISHLTTIQFLKLGHEPKNYWPSQNILFLLDLNCTNVTNHLKLSNDKNLFRSPYRWFLIGIDETLNNSNSNINIKRQFKPFDIFPDSEVMIILFHYNSRNDSRYEVIDIYKTCKNSEDMKTKLYGNWDATNRFQKSLNFYKPTALQRLDLGGCEIAISYVLTNNNSIHHLYDQMDDHVDTITKVNFPTTNHLLEFLNATRKYSFTDTWGYRLNGTWNGMSGYLFRGEVEIGGSPMFVTSERISFVEYISNPTPTSSKFVFQQPKLSYGNNIFLLSFRETVWYCATALVILIFLTLFAVTFWEWKKINDGNMLDNRDPGILRPNVTDILILIVGALCQQGSPVQLKGSLGRIVLLVLFLALMFLYTSYSANIVALLQSSSSQIKTLEDLLHSRMKFGVDDTVYSRYYFSIATEPIRKAIYETKVAPRGEKPRFMSMEEGIKNMQKGLFAFHMEVGVGYKFVGKYFLEGEKCGLKEIPYLQVQDPWLAVRKNTPYKEMFKIG*

>HrhoIR68a

MNFKSKLYKPVNADINKWGQKQSNGSFSGLIGEMVRGKADVGLGNLQYTPYHLDLMDLSIPYTSQCWTFLTPEALSDNSWKTLILPFKLYMWITVLLVLLVTGSIFYGLAKFYLNLLEFKDHSSIYDKHLDKQIVYDGAKPVGLYLFGEIINSILYTYGMLLVVSLPKLPTGWSIRLLTGWYWLYCILLVVSYRASMTAILANPAPRVTLDTLKELVESKIACGGWGTQTKIFFEESLDEIGEKIGEKFQIVNDPDEAAAKVAQGVFAYYENKHFLKYLSVKRKNSLIDTTPQDNSTANSTAVTAKKKDERNLHIMSDCVINIPISLGFQKNSPLKPLADLYLSRIVEVGLVEKWLNDAMHPIKSLDSQEEEIKALMNLKKLYGAFIALAIGYFLSTISLIGEFIHWYLIVKKDPNFDKYALDVYYANKNKRQ*

>HrhoIR75p

MKLYNLLYILLCIPTDVACSRNVDDMLLSYITMENQPTSLLAPELCWPLHHKTSFTRLLNGVGVNVAYTMRPSRKEQYLHHITILADFSCSSASDLVLQSDEHGFFMSPYRWIFINLHQQSPNATILDKLNILIDSNVVVVQKVDDTKYVFHEVYKIAKDYQVIKNLRAVWRAVNDTEKRNEQTTSSIHVNLTPGNNSVSVNSKTNGEIEDLFYSTPLSCRRRNLRGHSLTMVNVITDSNETKNHMHDRLFLHHDSISKMSYMVVRICFEMMNASENLLFTNTWGYRDKHGNWQGLIDHLLKKKADLGTLTIFTKERTEHIDYIAMVGSTAVRFVFREPPLAYVSNIFTLPFSGAVWFAILICVLGCALFLYITSKWEATMGSHPLQLDGSWADVLILIIGAVLQQGCTLEPRFTAGRCVTLLLFISLTILYAAYSANIVVLLRAPSPSVRSLQDLLSSPLKLGASDFEYNRYFFRQLNDPTRKSIYDKKIAPKGKKPNFYNMTEGVEKIRQGLFAFHMELNPGYRLIQETYQEDEKCDLVEIDYINEIDPWLPGQKRSPYKDLFKVK*

>HrhoIR40a

MRVAVVTNPRESVFRIYYNQGTPNLLHHLTLVNWWSGRLYRSPVLPPAEKVYKDFRGREFEIPVLHAPPWHFVKYNNDSTVNVTGGRDDKLLSLLAKKLNFRYKYYDPPERSQGSSISGNGTFKGTLGLIWKRKAPFFIGDMTMTWERLQAVEFSFLTLADSGAFLTHAPAKLSETLAIIRPFRWEVWPLVLATLLVTGPALWMVIAAPSLWRRQRRDQLQLFNNCCWFTTSLFLRQSSSKEPSSTHKARLVSVVISLGATYVIGDMYSANLTSLLARPAREQPIGTLQALEEAMRDNGYELVVERHSSSLTILQNGTGVYGRLAKLMKRQQVQRVRNVEVGVRLVLTRKRVAILGGRETLYYDTEKFGSHNFHLSEKLYTRYSAIAMQIGCPYLETFNNVVMTLFEAGILTKMTTDEYRDLPKLSRRSDPVTESDTEGSDAIGESTAASQTQVESTKGLEPVTLRMLRGAFCLLGIGYLLAGVSFCIEIQIHRRRTRTKAPVPETKIKEKQKKFQRILTNIKIRFRRIAIKIYSKIDTALGP*

>CpomIR7d

SVDMPIDGELNDDLMLFNKNITAAQNLAINAAKIALNNFEWRYVTMVFHNSSILLGLTAFMQIYRKSVIVGKGTFLHGKESSADRISQFVIFGSDLVDIMCTLDWMRKREFDNTGKFIVICNNCDERKAMDIFWNHKILNVVFINDSSGTSSLIGFTYSIYDNQKCVISPPEPLLDSCIHNSCMGVYPLKLRNLHKCQIIVSTFEQVPFMSLKTGTPIGADGDLLLLIAEALNATLKVMTPHRGAGWGQLDKDGNWLGSLADVYHDLANFSMTSAAITLTRFKAFHLSTDYHSINMAWVTHPAVPLPGWQKLLRPFKMKARISLAVTFVLIILVAVFVKSNLWAKLSKRINTSARPQTCVLFYSWTICMGMPATSLPSKPTFLTMFLLWMFYCFMIRTFYQTSLIHAMKDNLNYPEFENLQDILNSGYPFGGVPALKDFYIDDPEVYNNWKSINSTEINDMMVSLSRGMKYVLAMNKVTAQSFILKHYGDIHIVPQMIVTSPTVLYFKKFSPMVQSLNLILDRLVEGGFTEKLYKNHASTHARKKTDSTAPMNFEQYMGCYVVLAAGWIVSILVFICEVYCYKFSV

>CpomIR8a

MDFCCLFLAIFIFNLGCVASELSLRFVFIIESHEQDLPQLIGRALKFAEEAQPDLRVSEAIVSLDRENEDESYRQLCSALSNSVSIIVDLSWSPWDSLEELSSTAGVPLVRARLGSQHLVRAVDEYLESRNATDAALLMESEADVDRTLYELLGESNIRVWVHAGLTRDSARALKTMRPEPSFFVVVGSGAFATDTYKRAVKEKLVRRDYRWNLVLTDYSNLELQPVKPAMVLQVDAAECCKVMGQKDGCSCSQDFERKQPILSALLQLLAETYSKLDDDDFTTRVDCDNLVPENGTRSKVYRQLAEELGASNESLFYWDGERSGIFLRSRFILSTLKPDIGPQHAAIWSADDEYKLLPGVTLEPLRQFFRIGTAPAVPWTMPKLDSNTGEPMFNEDGEPMYEGYCIDLIQKLSESMDFDYEIITPKTGTFGRRLANGTWDGVVGDLMRAETDIAVSALTMTAEREEVIDFVAPYFEQSGILIVIRKPTRKTSLFKFMTVLRTEVWLSIVAALVLTGFMIWLLDKYSPYSARNNPDAYPYPCREFTLKESFWFALTSFTPQGGGEAPKALSGRTLVAAYWLFVVLMLATFTANLAAFLTVERMQTPVSSLEQLARQSRINYTVVEGSTIHQYFINMKFAEDTLYRVWKEITLNATSDQSQYRVWDYPIREQYGHILLAINASMPVPDAKTGFRQVDEHTDADFAFIHDSAEIKYEVTLNCNLTEVGEVFAEQPYAIAVQQGSRLQEELSRALLDLQKERLLEQLAAKYWNETARQQCPDADESEGITLESLGGVFIATLFGLGLAMITLAWEVFYYKRKEKNKVRQEDEETKPKKAFEKDLEKKIAGGVARLRKRDKKEKKGQVTIGDTFKPVSEKDGVSYISVYPKTEYKP

>CpomIR21a

MRFLRTALFNYILLHYVISQEIEYYPSQASSFARKLVSEFNSEPYQHKHDLFKREAQWRKFNNNDDTEFTKNKTQKRAVDPVFHGHPKTREELWNERIINESLAFDQTPSLISLIHNITLTYLNDCIPIILYDSEVKSKESYLFLNLLKDFPIAYVHGYINENNELAEPKLVRATRECIHFIAFLSDVTKSAKILGKQAESKVVIIARSSQWAVQEFLAGPQSRMFINLIVIGQSFKDGDDDTLEAPYILYTHKLYTDGLGASQPVVLTSWSHGKFSRQVNLFPRKMTEGYAGHRFVVAAANQPPYIFRTIKTDADGGNPRVVWDGIEVRLLTLLSQMNNFSIEIKEPREPHLGSGESVLKEITGGRADIGVAGIYLTSDRIRDTDMSFSHSTDCAVFVTLMSTALPRYRAILGPFHWTVWLALTLTYLFGIFPLAFSDKHTLKHLLHNSGEIENMFWYVFGTFTNCFTFVGKNSWSKTTKITTRLLIGWYWLFTIIITSCYTGSIIAFVTLPVFPETIDSIQQLLDGFYRVGTLDRGGWEKWFLNSSDPKTNKLLKKLQLVGDVPSGIRNTTKTFFLLPFAFLGSRAELEYIIQSNFTKTKKSKKAQLHISNECFVPFGVSLTFPNNSLYSSKLSGDIARILQSGLMDKIENEVKWEMQRTPSGKFLSAGSGTLKLGAITEKGLTLADTQGMFLLLAAGFVLAAAALISEWMGGCSRKCRPQKKEDEPSSAHSREHLIPTPKSDVDSEIKVISDSAESRFRLNPRPDSEDSRDSLEGTIINVTKESIIIHNNYHTSNWDSRRSSSVDIDKEVQEIFEKDEKRRRINSGTVPLKDNQREATASKGAFGDHLSDH

>CpomIR25a

MASLIILLLFLFVPDSFSQTTQNINVLLINEENNALAEKSFEVAKEYVRRNPTLGLAVDPVIVVGNRTDAKAFLENVCRKYNDMLSAKKTPHVVLDFTMTGVGSETIKSFTAALGLPTISGSFGQAGDLRQWRNLDANQTKFLLQVMPPADILPESIRAIVTKQDITNAAIIFDEFFVMDHKYKSLLQNIPTRHVITPVKSFNRDEIKTQLRSLRELDIVNFFVVGSLRTIKNVLDAADENQYFGRKTAWFALTLDKGDISCGCKDATIVYMKPTPDAKSRDRLGKIKTTYSMNGEPEITSAFYFDLSLRTFLTVKSLLDSGKWPNDMRYISCDDYDGKNTPNRTLDLKTAFHEIKETPTYAPFFIPEDDPMNGRSYMEFNTDLSAVTVKDGASIGSRNLGSWKAGLSNPLSLTDPQNMSDYSAQLVYRVVTVEQKPFIIRDDEAPKGFKGYCIDLIEEIRQIVKFDYEITLVPDGNFGTMDENGNWNGIIKELVEKRADIGLTSLSVMAERENVVDFTVPYYDLVGITILMKLPRTPTSLFKFLTVLEDDVWLSILAAYFFTSFLMWVFDKWSPYSYQNNREKYKDDEEKREFNLKECLWFCMTSLTPQGGGEAPKNLSGRLLAATWWLFGFIIIASYTANLAAFLTVSRLDTPIESLDDLSKQYKIQYAPLNGSAAMTYFERMAHIEVRFYEIWKEMSLNDSLSDVERAKLAVWDYPVSDKYSKMWQAMKEAGLPNSIEEAIQRVRDSESSSEGFAWLGDATDVRYYVLTSCDLQMVGDEFSRKPYAIAVQQGSPLKDQFNNAILQLLNKRKLEKLKENWWNNNPEAMKCEKQEDQSDGISIQNIGGVFIVIFMGIGLACITLGVEYWWYKWRKRPIIGDVTQVEPSKTTRNNADNSTTKIGEGFTFRSRNMGLSNFRSKF

>CpomIR41a.1

MIMPSKLFPVEILLNILINEHLQEYFCLTFVTETKLTVNIPINMSLMIIQPNNSVLAEQILDASEKGCSDYIIQMHEPENFMIAFEKVNHLGDIRRSVKKLIFLPVQDDMNNRSVLTNILALRETGFVANILLVVPSLQSSGDCKVYDMITHTFVGSDEDVQKPLYLDRWDSCTGHFERGVNLFPHNMSNLYGKTVKVAAFTYKPYVLLDLDPSLNSLGRDGMEMRIIDEFCRWVNCTVEIVRDDEHEWGEIYENNTGVGVLGNVVEDRADIGITALYSWYDEFRVLDFSAPIIRTAITCVAPAPRILTSWDLPLVPFTWTMWMCLVFTFFYASFALSIAQRSTDNVFLDTFGMMITQTREDATSWRIRSITGWMLVTGLVIDNAYSGGLASSFTVPKYEASIDTVEDLVDRKMEWGATHDAWIFSIMLSEEPLIKSLLSQFKTYPADILRQKSFSRSMAFSIEHLPAGYFAIGEYITKEAAMDLEIMLDKIYYEQCVVMLRKSSPYTAKLSELVGRLHQSGLMLSWETQVALKYLDFKVQLEVRLSRARKDLEEIEPLSIKQLLGIYIFYFGGVVIALLVFFGELLSKCSKPSIVL

>CpomIR41a.2

MVKMLIPSTIYFPIEILLNTIINNYLQTSFCLTFVTETELMINLPLNMSSMRIIPNNSELVQQILETSEKACTDYIIQMDEPRNFMIAFDKVNHVGDVRKSDKKLIFLPLEDEFYNPSVLTDLLSLKETGYVPNILLITPTGQKSSDCKVYDMITHTFVGAEEQIQNPLYLDRWDCCTEVFEKEVNLFPHDMSNLYGKKVKVGAFTYKPYVLLDLEPSLAPLGRDGIDIRFIEEFCRWINCTVEIVRPDDGQEWGEIYENNTGIGLVGNLVEDRTEIGITSLYSWYEEYRALDFSAPIIRTAVTCIAPAPRILSSWDLPLVPFSWLMWMCLIATFFFASFALFVAQRSTDDIFFVTFGNMIGQSPGDSSSWRIRSISGWMLVTGLVIDNAYSGGLASSFTVPKYEASVDTIQDLVDRKMEWGAPVDAWLYSMILSEEPLIKSAISQFKVYPPETLTKKSFTRSMAFSIERLPAGSFAIGEYITKEGAKNLELMVEDMYYEQCVVMTRKSSPYTAKLTELVGRLQQSGLLLCWETQIALKYLDFKVQLEVRLSRTKKDIDGVEPLNVKQLLGIYLLYFGGLSISIVVFIAELLIKRGKAVIVI

>CpomIR60a

GFYVQATMLKIICLLSIGVNAKVNPHGPTVVSDFSSCVSEIIDKNFAQSGLLFFVDTFNVSTPVAGIRNGIIKSVHTKLKYSVKIAIPTKKDKGICVNNDKTAIEISVKSRMDHFEATSLADYFILIIEDYKDFSYIASRLIRAISWNPRALFILVYFSISNSDDQNIRHAEDMLFCLFKVNVINAVVIIPEVNNVRRANIYSWRPYAPPKYCGHYNESIRNRLIVENVCERGKIKYAKKIFESKIPSDMMGCSLKVLALERQPFISHNPLDPNIESLLINQVAKRYNLSLRYEILNSFRGEKLFDGDWTGALKELTYKKGHLLLGGIFPDDEVHEDFECSSNYLADSYTWVVPRALPKPAWLALFVIFQKTVWLTVITCFVFIALSWMVLAKLSKDPTYRTNLDHYFINTWLSNLGFCAFSRPITNSLRLFFVFINIYCILLLTAYQTKLIDVLTNPSFEYQISTVEELVESGLKFGGSEELHDIFENSTDSIDNYFLDGWIDIADIRDALRDVAIHRNFSLMCSRLELAYVSAIIPELSDQFGKYMYYAFPTNVFTVPLEAVSMKGFPFMKGFSRTLTHFEQHGVNNGVIVYFGGYLLRQRALLLNKFKIEHNSRDPLSIQTLQGGYLALMFGSVCGTFVFIVEIILNTKFVKKLKIL

>CpomIR64a

MNLTTYSALFTIFSTAEINLITDVFKHKHLHFGTIFHCSKPENAIFLQKHLKKMDLRFSTIMMHSNASHFKQTNDSRVGIVLKTSCENWTQVFEHFNCNLFEKSLYSWLIFTDDLSSASEALSRYPIEVDSDVAIIYRQEKSYYIYEVYNTGYFTNGRYHVEPVGYWYYKLRIKGHRRTNLDGIVLRSAVVVTHSIGHQTFEEYISRLKPEVDSLHKLKYFTLLNYLRDMYNFSLIVQRTNSWGYVTNGSFDGMVGTLQRGETDIGGTPVFIRADRAKFIYYVTATWPSKPCFIFRHPKHPGGFLTIYTRPLSYNVWLCIIALLVFAGSLLCVLIKLRVTRTAGDDGDLSASLALLSIWSAVCQQGTTVNLSANSVRLVLFFSFLFSLFVYQYYNALVVSTLLRAPPVTIRSLEDLLRSKLKAGVEDVLYNKDYFRRTTDPIALELYSRKIASSPRPNFLPPDRGMALVKQGGYAYHADTAYSYPIIRRTFTEREICELQEVELFPPQTMFAVMKKGSPYIKHLSYGIRKMAESGLMQRLKTIWDEPKPLCVRTPDSSIFSVTLREFITPLLLLCLGMLAATVVFMAEHVFYRLQWKRIQFRH

>CpomIR75d

LQVQTRIFAGMELVSFALAYFAAKRLSLLTAFLCWRPEELSALCRDAQRQGMRISIADWTHLPPLEPYATHREGMLLDVTCPDAPLVLEKASSTRAFNLRHTWLLLHNAPFNASLMEVTLDSTLVLPDADVAWVANDQFLDVYRIKHDQALITMPLGDDAARVALPAAPTRRRDLNNVYLRSSTIISQPQFFKGWNDLTVRQIDTFPKLTWPLMHLLADDLHFRYNLIQVDLYGENRNGSFDGLAGQLQRQEIEVGITSMFLRADRMQVLHFCSETVELRGAFMFRQPSKSAVSNVFLLPFSRGVWIATALTLVLAAVTLALLARRPRLXXAVDASLEQLSIGEAVIFTVGTACQQGFHIVPELASARVVMFCALMTALFAFTAYSAKIVAILQTPSDAIRTIDDLTNSPMTMGVQETTYKRVYFAESTQPATQRLYRHKLLPLGDRAYLSVVDGVAAMRTGLFAFQVEEPSGYDIISKTFTEREKCGLMQIQAFKLPMVAVPIRKHSGYKELFATRLRWQRETGLMDRTRRIWLASKPRCDANSGGFVSVGIIDILPALHVLAAGMVASVLFLVLERSMARLKCCGVRGS*

>CpomIR75p.1

MDIWKLGAIIWLFKSHVEGRAGIGKFLTSFVDNERKPTTVVFHGICWNNSVKLHVMKELSKAGIRSSQSMSKRTSLIDHTVLLLADLNCTGTDDLIINATQRELHRLPYRWLVLSDAPRFGRSSLWDLFLVDSELVLATVDGAGYSMTEVYKPSPTSPAILTPRGTFHHVLTDTRPHRELFRRRRDLMGVPLTITNTIQESNSSIYHLLQEDSLELEHDLISKNSYTLAKVAFLTLNSTPVATFTNSFGYLQNGQWTGVIKELLEYNADIGTNVGMSQSRLKQVMFLDPLDNGRARFIFRQPALSLTANIFSLPFSPDVWIATGLSSFVAGVAYYLSTRLIKTRAEKGTVRDAYLLTMSALSQQGCEVQPRHVSARIVLWVVFTSMMALYAAYGANIVVLLQAPSTSVNSLATLAKSKLALGAADVNYNHFLFRASSDPVRNDIAKRINSDKGPKAFYGLTEGVEKIRKGLFAFHSVVEPVYRQIDRTFQEKEKCDLMELDYIGYAAFHVPGSKKSPYLELLRVTFKRLREVGIKSAVNFRYEARRPSCKESIAMFSSVGITEMRPVLIFMAYGVALSVAVTAAELLVFHANRYRLRQQRLAVQLGRI

>CpomIR75p.2

MKILFSFVIVLFLSLGKAFDDNDINMIVSFVTLDERATAVLTPYVCWSTYELTSLAKSLHDTGISMAASLQPKRPELFLQNLVIVADLRCRRTDDFLIKASDEGFFKSPYRWLLISQDQTELNVLDQLAMLVDSDVVIAQRRGADYQYVEVYKIVENSQLIYNTRALWRPIDKNNNTAIITYYNKSKVVANKYGAVEDYRKSKILSTRRMDIRKHTLTMVNVITDSNDTRKHMDDRLNLHQDSITKMSYMVVKICFEMMNSTEKLIFTNTWGYVDKNGSWNGIIERLIKKEGDIGTLTIFTQERLKIIDYIAMVGTTAVRFVFREPPLAYVSNIFALPFTGAVWLAVFICVLACALFLYITSKWEATMGIHPMQLDGSWADVLILIIGAVLQQGCTLEPRRAAGRIVTLLLFIALTILYAAYSANIVVLLRAPSSSIRSLQDILNSPIKLGASDFSYNRYFFKKLNEPLRKEIYNKKIAPKGKKANFYTMKEGIEKIRKGLFAFHMELNPGYRLIQETYQEDEKCDLVEIDYINEIDPWVPGQKRSPYKDLFKINFIKIRESGIQNCIHQRLHVGKPRCLGAVNTFSSVGIMDMYSAMLATLYGMFMAPAVLLLEIAYKRLMVAREKRMQHNNSHSHT

>CpomIR75p.3

VRRRDLRGADVVLPTVLLHNQSLEDLPDYLHRERDTLTKVVYYMCTHLVEWVNGTKIWNRTTSWGYLQPDGQWDGIVREMQDGRADIAGSCMMAQKERVKYVNYVLAPSKIEAMFVFKKPALASVTNIYVLPFDIGVWVSIIVLIIISSSTLFLSSFGEDRMKNYYSKSWLQKMSDGFFDTLCLMFQQGTAADPLSIASRQILLLGLMAFMFLYTAYSANVVALLQSPTNDINSIETLLTSPVACGSQDVVYAWQMFGHESRPIHRLLADRKINSQGKKGFLSVEDGIRKVREGMFAFHVEQTAGFDQIQKTFLEDEKCNLGFIKYMSTTSPFVAFSQITPIKEMLRIGANRIMEVGVQSRSARRLVPERPRCGASAAMFNAVRLSDVAPAFRVLLAFYALTLPILGLEILVKRREMKRGIFESKSVDTATAEITNNIII

>CpomIR75q.1

MYTSLTIVCAIFICSSFALSISKENDIKVIVDVIQSFNKPTDVISNVCWTNIYKKKLTANLAAADNPRSIKFVNDIHAKDLVHPEKVTFLIDVRCKDSGGFLNKAASRKYFGRPHRWFIINTPANEVSVPLVIDKMHLLPDSEVYVMQLINNSYSINLIYKIKPNREWIIENYGNWSTENGLTISRRAKKVALVMRRRNLARASIVTSMVITDNGSFADLETLRYKQIDSVTKGGFHQLTALYEFMNASREFVFTDQWGHHVNGTWHGMVGHLADGTAELAGAILFITKERMPLIEYMSHPMESSIKFLFREPPLSYQNNLYLLPFQASVWYCVGSFVLVLIFAMYFSAYWEAKKVADEKQKADDTTVLVPTISDVTIFVMCAISQQGSTVELKGMLGRFMILILFLVFLFLYTAYSASIVVLLQSSSNQIRTLTDLLNSKLELGVEDTPYNRYWFMNEKEPIRRAIYEKKIAPSGSKPKFFDLTEGILQLQKKPFALNCNLGVAYKVMERYFYEHEKCGLQEISYLQNNNPWQAVRKGSPYREIFKIGLLRNAEFGLNDRTNRIMFSKKPVCSVRGGSFVSVSLVDCYPILLLLLYGMILGVMLLLVEILYYRKMNTRP

>CpomIR75q.2

MVADVIRAMQRPSAVIAMLCWSSNLKLQLYSALEGENVTQITMMQFLKAGTVPERHAQDQHIVFLADLDCPDIISYFQTSSLNKHFRSPFRWILIDSGNNDTSQSYIPNAVGNFDILVDSEVILAHHLGDGSYRLHLIYRIGNNTDWKKEFYGTWDERRRLQKQVMEGEIILRRIDLESYELPICYVLTDNDSINHLYDNVNDHIDTITKVNFPTTNHLLDFLNASRKYVFANTWGYRVNGTWNGMTGYLVREEVEIGGSPMFFTSERISIVDYISSPTPTRSKFVFRQPKLSYENNLFLLSFRASVWYSSIALLLLLVIVLFIVTIWEWKKTRGHEDKKLEADSGILRASVVDVVLLIFGAACQQGSTVELKGSLGRIVMLILFLALMFLYTSYSANIVALLQSSSSQIKTLEDLLHSRIKFGVHDTVFNKYYFSTATEPVRKAIYETKVAPSGSKPRFMPMDEGVKKMQKGLFAFHMETGVGYKFVGKYFQESEKCGLKEIQYLQVIDPWLAVRKNTPYKEMFKLGTKRIQEHGLQSRENRLLYEKRPKCSGQGGSFVSVSMVDCYPALLVLFYGAVFSVGLLFIEILTKRRNDILRKISRAKTLGVDVEDY

>CpomIR76b

MTGLELIVSSICNATFCEVVYDNPITDTLLPAQKKELLKIAEDLNGKHLKIGTYDNYPLSWVHTEDNGKLTGRGVAFVVLDILRERFNFTFDVVTPLKNFEIGIEGRMEDSLIGLVNSSQVDMAAAFLPIVYKYQQFVDFSSILDKGVWMMMLQRPKESAAGSGLLAPFEIQVWYLILAAVLSYGPCITLLTYLRSKLVRDGEKNISLTPSFWFVYGALLKQGTTLAPEANTTRILFTTWWLFIILLSAFYTANLTAFLTLSKFTLDVEYPEDLYKKNYRWVAPEGSTVQYVVNDADENLHFLSKMVANGRAEFRSVNADRQYLPYVMGGAVLVKEQTAIHHLMFEDYLKKTKAKVPETKRCTYVVAPNPFMEKLRSFAFPKNSKLKLLFDPVLTYLLQSGIVTFLEFRDLPSTKICPLDLQSKDRKLRNSDLSMTYMLMGVGLATAIAVFGGEMIIRYYVRIKIRKNRGERTRTKTVKTSKHRRFRIQDDSHPPPYDSLFGQNSRYKMNGDSTTKIINGREYWVVGTVSGDIRFIPVRTPSAFLYQRDK

>CpomIR87a

MLLLRTIHSGIKYSVMVKDSFYKHANASHFPEKAKNYMLILEEKSELVRNILQLNKLPTWNPLAKAIIYYQLLPDEDGETIAKKFINELREYKLLKSIVFIYSPDDAGLISYTWAPYSDTNCGGECDSVYILDTCKNSIVKQKNAQREMFPLNMKKCPLVTQAIISEPYVMPPVRQLTNTSYPDAYEFQKGGEINLVKLISEFTNMSLIVRISDVPENWGLIYPNGTATGAYGILRNDSVDLVIGDIEVTRTIRKWFHPTVSYTQDEMTWCVPKSAQASTWNNLVIIFQWTTWVATLLSIVTMGLIFHYIYYRENDRKVTKLPTNSLLNTFSMILGWGASFKPKTATFRILIFAWLFFGMIMSISYESFLRTFLMHPRYEKQISSETDLIQSGIPLGGRAIYRSYFETNNASSFYLYRKYISTSFSEGIKRAALERNFAVVASRRQAEYQDQKLGKGEQLLYCFKEGNNLYKYGVVLLARRWFPILERFNNIIRSVSENGLIEKWNQELFIHTVGVDGTSKVVPLGIRHLLGAFIFIGIMYAASVIVFVVELLLNVSKKRKGNKPICRACLSSKYSSRR

>CpomIR93a

MRIWVVLICVVGVRGEEFPSLITANASIAVVLDRQYLGEQYQPLLDTLKDYIKELARVELKHGGVVVHYYSWSTISLKKGFIAVFSIASCEDTWSLFSRAEEEELLLFALTEVDCPRLPPDSAITITYTDPGQELPQLLLDLRTTRAFNWKSAVILHDDTLNRDMVSRVVQSLTSQIDDEDVPTISVTVFKMRHEINEYLRRKEMHRVLSKLPVKHIGENFIAIVTSDVMSTMAETARDLFMSNTQAQWLYVISDTSIRNSNLSSFVNALYEGENIAYIYNITDDREDCKNGLMCYSEEMMNAFISALDSAVQEEFDVAAQVSDEEWEAIRPTKIQRRDTLLKHMQQHIAVNSVCGNCSTWQAMAANTWGSTYGGNVQADNVAAPDNETNEAIQKIELLQVAYWRPSDGLRFTDFLFPHIVHGFRGKVLPIITYNNPPWTILKANESGSISSYSGLIFDIVDQLAKNKNFTLKLIFPGDMKDVLSNKTVTNDMYSQSAKLTMMAVARKQAAFAAAAFTVLSDRNPGINYTIPVSTQSYAFIIARPRELSRAMLFLLPFTTDTWLCLGFAVVLMGPTLYVVHRLSPYYEAMGVTRQGGLATIHNCLWYIYGALLQQGGMYLPRADSGRLVVGTWWLVVLVVVTTYSGNLVAFLTFPKQEVPVTTVSELLENRAVYTWSISRGSYLEFELKNSDEPKYVSLLKGAELTSDSSGLEGNLASGSPLLSRVRQDRHVIIDWKLRLSYLMRAEHLATDKCDFALSAEEFLDEQVAMIVPAGSPYLPVFNKEINRMQKAGLITKWLSAYLPKRDRCWKTSSVTQEVDNHTVNLSDMQGSFFVLFLGFFSASSVLLMEWFYHRRKSQKEDVAIKPYVE

>CpomIR1

RMTMWRLLFLVASAASLPTDWPHMAVDYFQHKHVKYVAHLSCKDAAEIKGVLRLLMNEGIRAAVGLIDQGPMNIMPLLYQYEASVGVLVDGDCINTRDILNNASESMMFDDTHFWLVMNDNCSMGFVEDTFLDLKLSVDADVVVASYCGDIYQLTDVFNFGRVQGNVLETRELGAWTSERGLEIVLQGFKYYNRWDFHNLTLRAVSVIRNSSKEFHEGMLYEPGFTVGVAAMTKISSQLLNLLKEMHNFRFNYTIVGRWIGTPERNSTKAMSNMLLWRDQDISSTCTRLFSNWLDWMDPFFPSVTELETKFYYTISEKGIGDYENQFLTPMSPEVWWCAAATGVVCALXXXXXXXXXXXXXXXXXGVFSVLAAGFQQDYEDGHQTKKDSSSRKLALLVVGLTSMLMYNYYTSSVVSWLLNAAAPSLDSLDGLIKSDFELVFEDIGYTRQWLDNPGFFYYMGYKNEKEDELRAKKVTNVKRTLPLFESIEDGIELMRTGKCAFHTEPYTASQVISRTFADKDLCSLAGLQIMPPSYVYVMGQKNSPYRQFFVWSMMRLLERGHTRATRARVGGQIPPCSGLTPRSFKIFSI

>CpomIR2

MEEETFTVASTPDCMTVLQDERNDPLWRETQVGCKVLLLLQEIHKFNITYSKGPYHYNERGFYEYVDEVDIYAKPRSLWSPVMVNYTPIAPIMDWKFGYILRHPFNIQHFYSLAFSKPAWHFIVAMILLVSVLFYILNRAEQKLTGENLKCYFWSELLIAFGIICQHYISINPMELTSRRIAFISFFMFSYILYSYYTSTLLSDLVYDRDNEMDLETLAESDYEHAVLDSVTSVFKVLVEQLRNNNKMFPQRQSFTENKLINHRVVNISTGLAEVKMSKTALLSDYVSIHSGVIQWFSESEVCDLIKVDIFSNVLKYLVTSKKFKYIDEFKISTLRAYEAGVLQRLLSPHPIQHFSPTCVSSHFQADIGLVRKPFILLALGYVLCGFILLVERVYYNRYKVWPYVN

>CpomIR3

LSAKMVGKSILFFLLSSVAGLEDRDIDFSVDFLKARDVKYICMLTCGDRTWNKKFAKNASKSSIAVSYVRIDDSLSDLDSVRVCLSPEFTDVGVLIDTKCPLYEEVLMYASENLLFDANHKWLIIDIDTWISNISTVFNVETNENFSWLMNTLEKLNMSIDANVMLSLQKGSENNIYEVYNFGKLRGGNVVVKKLGNWRNRADLIQHLNAYKYYRRWDFENFTINYVAVMSTPPKVFDVNMLVGDTPAPGVAVMTTTVTRVLLEIAELHNIRYNYTIVDRWIGKFERNTTPVVATLLYFKEQDITPVLRVTSEVFQRVDMVSPPITSIETRYYYRIPTTGPGKFENQFLRPLTKGAWGCVIAVISLCALVLFLTVRAETRPAALQYAVFSVAATFCQQFFEDGGYDDPRRESSARQLTILVTGASCVLIYNYYTSSVVSWLLNGPPPSINSLQELLESPLSLIYQDIGYTRSWLQNPKYYYNKKNSEVEDQLRKLKVFKKKKGEPLLVPLEEGIEMVKAGGYAYHTEVYNANMLISRSFNQEELCELGSLQSMEETPVYIAIPKDSPYKEFFNWKLVAKNELKFIGKMKHRILQGHE

>CpomIR4

MIIPAVAAFFKYKIVSSIIIFTCGNEFEQIRLVRQLSLQGMRATVSCDPGILNEEHKTLQGVLYFNRPNDTLLDETSWEHFSMWYKWLIIGNEVPSRLNHTTRYDADITLLGLRQLGAIDAIDNSSVAYHESILFEDLYVHLRDGVSRHPWAVWTPAGFQPLYELERIRRRHDLKRYTMRIPTPVGHYDDSYEGTFADYVMDNSQPGRDSAIRCGYGTSSLILEWLQAKEVILQMEQWSTDAGNKSMFTRLAQGTSELSGGILRMQHKRLLKLDYVIPLWIFKVGFTYVAERESSSNMFVIPFTGTTWAACAVVTLVLAIAQRATAKQESEKEGAFVAVMATWLQQDASAVPEGASGRITFMALSICSMLVYAYYSSAIVSALMSAGSSGPTTLRALGDSRYRLASEDYEWIRAQMFDVYIPNWPEMEYLKRKKLQSMANFYLDWQAGMQLVKSGTTAYHAEYNHVYPLMSVLSDDQVCKLQYVDTVPPIMSWLVTTRRGQWTNLLRIGGDWLHETGLVKRMLSRWQLKPPPCRAALLAERVSYGDVAPLIILTVVGLLTSVAVLFLERAVAKWRAKKTDKSS

>EposIgluR

MQGLTPSPMSILDSLCKEFLAVNVSAILYLMNHEQYGRSTASAQYFLQLAGYLGIPVISWNADNSGLEKRASHAALRLQLAPSIEHQTAAMLSILERYKWHQFSVVTSAIAGHDDFIQAVRERVTALQDRFKFTILNAVVVKKPADLNELVTSEARVMLLYATREEAADILSTAGDLHLTSENFVWIVTQSVLGSMQQPNKFPVGMLGVHFDTSSSSLIAEIATAVKVFAYGVDSYVSEPENARHPLGTRLSCSGAGAGEARWSTGERFYRHLRNVSVDSEAGRPSIEFTPDGELRAAELKIMNLRPAIGEQLVWEEIGTWNSYPKERLDIKDIVWPGGLHTPPQGVPEKFHMRITFLEEPPYINLAPPDPISGRCSLDRGVICRVAPEVDVAGLEAGTAHRNSSLYQCCSGFCIDLLQQLAEQLGFTYELSRVEDGRWGTLHNSKWNGLIADLVNKRTDMVLTSLIINSDREAVVDFSVPFMETGLAIVVGKRTGIISPTAFLEPFDTASWMLVGAVAIQAATFSIFFFEWLSPSGFDCSTGQNSKRVPQNRFSLCRTYWIVWAVLFQASVHVDSPRGFTARFMTNMWAMFAVVFLAIYTANLAAFMITREEFHELSGLDDPRISRPLAIRPPLKFGTVPWSHTDATLAKYFREPHAYMGQYNRSTVSAGVSGVLTAELDAFIYDGTVLDYLVSQDEDCRLLTVGSWYAMSGYGLAFTRNSKYLSMFNKRLLDLRSNGDLERLRRYWMTGTCKPNKQEHKSSDPLALEQFLSAFLLLMAGILLAALLLLLEHVYFRYMRAHLAASTVGPCCALVSLSMGQSLSFHGAVVEAAARGFGAGGRGHCRSAVCAAQVWRARHERDAAMARARQLAATLAAHGLQPPPRRLASAAALLGEAHDATRPRTLHAPADLLPDLDRPLSCGDLRAKERTRVEMETVL

>EposIR1

MWLILLLLATAVDSRLPPGEMVTDYFLHKDAKYVAYLTCGASAEYKAVTSVLMSEGMRAAVGRIDQETIDLDRLLYQWDAAVGVVLDGACRNTQNVLINASESMLFDAAHAWLVLADEADASARFEDLKLSVDADVVVATYFDEDKYQFTDVFNFGRIQGNALETKQLGTWTVDTGLDLPLPRFKYYDRWDFHNLTLRAVSVMRTIPKVLDEKMLSEPVYTDGITKFTKISSMLLNQLKDMHNFRFNYTIVDRWTGTPQRNSTPSLSNTLLWREQDISAASARLFPIWMNWVDPIFPPVTQLESKFYYIIPDSGVGDYENRFLTPMSADVWWCSAAAGVVCALVLVAAAATEGRPEPGSYGIFSVLAAGFQQDYEDGAQSQEDDSTQSRKLALLVVGLTSMLLYNYYTSSVVSWLLSAKPPSLDSLDALIKSDFELIFEDIGYARGWLDNPGFYYFSGYVNKKENELRMKKVTSAKRSVPLMQTVEAGIELVRTGSYAYHTEPYTASQIVSRTFVDEELCALGGLQMIKPAHVYIMAQKRSPYKQFFVWSMMRLLERGHIKATRARIGGEVPPCSGQTPRALSLGQAAPAFLLLVEFMVLALLMLVVEVWWYRYKKRTNKRGQAPLKVAQKSR

>EposIR3

MLRNILLIFLVTKVSGLQQNVKEFALDFFKTRDVQFICLLACGEKTWEQKFVNNASKLSIAVSSVTIDDSSDYSDALRVCLTQKYTAVGVLIDTKCPVFEDVLLFASENSFFNGNHKWLIVDNDVWMSIVTSDSDGNETNGNLFWLNSMFQKLNLSVDADVSLSLQKGSENNIYEVYSYGTIRGGSTIVSKMGSWKNKSALLPQLNGYKYYRRWDFNQSSMNLVAVMSTPPEVFDLDMLIGDQPKVGVAIITTTSLKVLEELKQLHNIRYNYTIVDRWIGDFDRNSSRVAANSLYFKEQDITPVLRVTREIFQKVDMLLPPLTAIETRYYYRIPTTGPGKFENQFLRPLTPGAWGCVCAVILLCSFMLFLAAKAERRPSAVQYAVFSVMATFCQQFYEDNIGNEDPTRQSSARQLAVLVTGASCVLIFNYYTSSVVSWLLNGPPPSINSLQELLESPLSLIFQDIGYTRSWLQNPTYYFNKKNAEVEDKLRRYKVFNKKAGAPLLVPLNEGIEMVKAGGFAYHTEVYNANTRIAKTFTQSELCELGSLQSMEKSQLYASVPKNSPYKEFFNWNLFRLHEAGIVSRIQRRTSSPEISCGGSSPRALALGGAAPAFMLLAFGFFLSTIILLLERFIHRNNSRWENKFNALAP

>EposIR4

MTEIAGGSSELSGSILRMRLDRIDRLDYVVPVWPFLVGFTYLSERESSSNLFVMPFSRGVWGACAAIALVLTLAQRATAKAPVERDGAYVAVVATWLQQDASAVPEGASGRLTSMMMSLCSMLVYAYYSSAIVSALMAAGGGGPTTLRALADSRYALASEDYDWIRYMMFDEETGWDDLEYLKRKKKTSSFYQSHERGMQLILEGNTAYHAEYNHIYPLMNIFSDDQICKLQYVYTVPPVMTWLTTKKQSQWKEVMHSVGGWLHETGLAKRLVARWQLKQPPCRAALLAERVKYGDVAPLVYLSTAGVIAAVVLLFLEIAVARWNARRSVESNGNDDTIIMEDDVA

>EposIR7d

MKVFSALTHSRLRCLVLPISSLHMDHFEPIPLADYFVAIIDSYDDFTRLASRLTRARSWNPKALFIFVFFGITSTDDENIKHAETMIHCLFKLNAINAVVIIPQANNIRRANVYGWKPYDPPEYCGYSNESARRRLFVENVCDRGVVKYAKTVFEEKIPSDMKGCTFKMLALERQPFISTDPDDPNIEQLLINDVAKRYNIKLEYNILNVFRGEKQINGHWDGALNDLILKKGELLLGGIFPDNEVHEDFECSSTYLADSYTWVVPRAFQQPLWLAFFIIFKKIVWFSVVAVFIVAALTWKLLAKLSGDPTYRKNLDHYFINTWISNLGFAAFSRPVTHSLRLFFIFLNLYCVLLLTAYQTKLIDVLTNPNFEHQMSTVEELAASDLECGGSEELRDLFENSTDPMDKYFESKWQNILDIREAMIDVALHRNFSLLCSRLELAHIAAVVPELSDKYGNTKFYVFEHNVFTVPLEMVSLRGSPFLKKFSKTLESFRQYGVNDKVRKYFAGYTLRKKAVLQTDLETENSSRDALSVITLQGGFMALLFGYVFGSIFLVVELIMNTKLVKNIKIFKKSVYTLI

>EposIR8a

MDFCCIFWFIFVINLACVVSELSLRFVFIVEVHEQDLPPLVGRALKIAEDSQPETRLSDSIVLLDRENEDESYRQLCSAVSEGVSTIIDLSWSPWDSADQLASDAGVPLVRTLLGSQQLLRALDEHLESRNATDAALLLESEGDVDRTLYELLGESNIRVWVHAGLTRDSARALKAMRPEPSFYAIIGGSAFVADTYKRAVKEKLVRRDYRWNLVQTDYSPPAVTAAAPAMALHVDPGECCRILAMKDGCSCSQDFERKLPILSALVQLLAETYSKLEDESALTARVDCEGVAPELNDTRAKLYRQLAEDSGASNESVFYWDGNRYGLFLRSRFILSTLKPDAGLQTVATWTADEEYKLLPGVTLEPLRQFFRIGTAPAVPWTMPKLDPATGEPMFNEDGQPLYEGYCIDLIQKLSESMDFDYEIVTPKVGTFGRRLPNGTWDGVVGDLMRAETDMAVSALTMTAEREEVIDFVAPYFEQSGILIVIRKPTRKTSLFKFMTVLRTEVWLSIVAALVLTGFMIWLLDKYSPYSAANSPHAYPYPCREFTLKESFWFALTSFTPQGGGEAPKALSGRTLVAAYWLFVVLMLATFTANLAAFLTVERMQTPVSSLEQLARQSRINYTVVEGSTIHQYFINMKFAEDTLYRVWKEITLNATSDQSQYRVWDYPIREQYGHILLAINASIPVPDATTGFSQVDEHTDADFAFIHDSAEIKYEVTLNCNLTEVGDVFAEQPYAIAVQQGSRLQEELSRALLDLQKERLLEQLAAKYWNETARQQCPDADESEGITLESLGGVFIATLFGLGLAMITLAWEVFYYKRKEKNKVQVLEDTEKKPKKAFEKEPHKKISDSVARLRKGRKKPERVTIGDTFKPVSEKEGVSYISVYPKTEFKP

>EposIR21a

MLVTIKIVLLKIIILYAAGQDVEYYPSQSLLNTFDGKILPQQYKFKSKFGVKSKPYLDSHFEYKREAQWRKFYKEDNVEKNNIRKRAVDPAFHGHPKTREELWNERFINKSTAFDQTLSLIGLLRNITLTYLSDCIPVILYDSQVKSKESYLFQNLLKDFPIAFVHGYINEDDEVVEPKIIRATKECINFIAFLSDVTKSAKILGKQADSKVVIVARSSQWAVQEFLAGPQSRMFVNLIVIGQSFKDGEDDSLEAPYILYTHKLYTDGLGASIPVVLNSWSHGKFSRSVNLFPAKMSQGYAGHRFVVAAANQPPFIFRTIKTDLDGGNPRVVWEGVEMKILSLLAERNNFSIEVKEPQDLHLGSGDAVTREVKSGRADIGVAGMYVTGERTRDMDLSYPHSQDCAVFVTLMSTALPRYRAILGPFHWHVWLALTLTYLFGIFPLAFSDKHTLRHLLHNSGEVENMFWYVFGTFTNCFTFVGKNSWSKTTKITTRLLIGWYWLFTIIITSCYTGSIIAFVTLPVFPETIDTIQQLLDGFYRVGTLDRGGWEKWFLNSSDPQTNKLLKKLQLVPDVASGIRNTTKAFFLLPFAYLGSQAELEYIIQSNFTRNKKNKRAMLHISNECFVPFGVSLSFPNNSIYSAKLSGDIARMVQSGLINKIMDEVRWEMQRGAIGQRLAVSPGSINIVSVEEKGLTLADTQGMFLLLAAGFILGASALISEWMGGCTRKCRLTKKEETPSSANSREHLIPTPKSDMDAEIKIISNSGDSRFHLNPRGSSADSRDSLEGTIINLTTENISVHDQFSVNGWDSRRSSSVDIDREVKEIFEKDESRRRAKSTAGVELTDSQRQATASKGAFGAHLSEP

>EposIR25a

MSSLTILLLFLFVPVSLSQTTQNINVLLINEENNALAEKSFEVAKEYVRRNPTLGLAVDPVIVVGNRTDAKAFLENVCRKYNDMLSAKKTPHVVLDFTMTGVGSETIKSFTAALALPTISGSFGQAGDLRQWRNLNANQSKFLLQVMPPADILPESIRAIVTKQDITNAAIIFDEFFVMDHKYKSLLQNIPTRHVITPIKSFNKDEIKTQLRSLRELDIVNFFVIGSLRTIKNVLDAADENQYFGRKTAWFALSLDKGDISCGCKDATIVYMRPTPDAKSRDRLGKIKTTYSMNGEPEITSAFYFDLSLRTFLTVKSLLDSGKWPNDMRYITCDDYDGKNTPNRTLDLKSAFQEIKETPTYAPFFIPEDDPMNGRSYMEFSTDLTAVTVKDGASIGSRSLGSWKAGLSNPLSLTDPENMSDYSAQLVFRVVTIEQNPFIIRDDDAPKGFKGYCIDLIEEIRQIVKFDYEITLAPDGNFGVMDDNGNWNGIIKELIEKRADIGLTSLSVMAERENVVDFTVPYYDLVGITILMKLPRTPTSLFKFLTVLENDVWLSILAAYFFTSFLMWVFDKWSPYSYQNNREKYKDDEEKREFNLKECLWFCMTSLTPQGGGEAPKNLSGRLLAATWWLFGFIIIASYTANLAAFLTVSRLDTPIESLDDLSKQYKIQYAPLNGSAAMTYFERMAHIEVRFYEIWKEMSLNDSLSDVERAKLAVWDYPVSDKYSKMWQAMKEAGLPNSVEEAVQRVRDSKSSSEGFAWLGDATDVRYHVLTSCDLQMVGDEFSRKPYAIAVQQGSPLKDQFNNAILQLLNKRKLEKLKENWWNNNPEAMKCEKQDDQSDGISIQNIGGVFIVIFMGIGLACITLGVEYWWYKWRKRPLVGDVTQVEPSKSTRNNADHGTTKIGEGFTFRSRNLGLSNLRSKF

>EposIR41a

MLEPPIILHPVEVLLQILINKYLLSSFCITMVTETELIIRPPSNLSFMYIYPEYNLTDQILDASEKGCSDFIIQMNEPENFMDAFETVNHVGDIRRSDKKLIFLPRQDDNFNASVLMDILGLKASGFVANILLVLPSINCTDDCNYYDLVTHNFVGTDDEVDQPLYLDRWHFSQEQVDNGVNWFPHDMSNMNGKTLKVAAFTYKPYVLLDLDPSDNALGRDGIDVRIIDEFCRWVNCTVELVRDDENEWGDIYDNLTGVGILGNVVEDRADMGITALYSWYGEYRVMDFSAAFVRTAITCIAPAARVLSSWDLPFLPFARLMWVCLIFTFFYASFALFIAQRSTDKIFMSTFRMMITQARDDTADSWRIRSIAGWMLVTGLIIDNAYGGGLASSFTVPKYEASIDTVQDIVDRKMEWGATHDAWLFSISLSEEPLVKELISQFKTYPEDELKRKSFTRSMAFSVEHLPAGSFAIGEYITKEAAEDLELMLENFYYEQCVVMLRKSSPYTAKLSELVGRLHQSGLLLAWESQVALKYLDFKVQLEVKLSRSRRDVDEVKPLNLKQLVGIFIIYFGGLSICLICFLVELLTRCGKASIVI

>EposIR68

MWITVLLVLLITGAIFYCLARFYTNLLQYQSDHSHVVVTAKDQDEKPVGMYLFGDIINSILYTYGMLLVVSLPKLPTGWAIRLLTGWYWLYCILLVVSYRASMTAILANPAPRVTIDTLQELVDSKVTCGGWGTETKHFFEESIDDIGQKIGERFEMIDDPNEAATKVAQGVYAYYENEYFLKYLSVKRKNSDDKMSIETQNNSTNATVQVKLDSERNLHIMTDCVVNIPISLGFHKNSPLKPLADIYMRRTVEVGLVEKWMNDVMYPIRALDTTDNEIKALMNLKKLYGAFIALAIGYFLSLVCLLGEVIYWNCIVKRDPRFDKYAMDLYYEKKK

>EposIR75q.1

MKVVLIASVVLLCLSFVSMNDAKILKMVVNVIQSFNKPCHVVANLCWGSFEKKQLMDGLGSVDNPKTVKFVQNMDLDHFVLREKTVFLVDLSCPSVTSFFNKANSSRIFNKPYRWLIVGSSVDEGFPSELDNLQILPDSEVYLSQSNGNDSFIINLIYKIKLGRDWISEYYGTWTNNGVIKSRISETSVAMRRKNLAQETIVTSMVVTDDSTITDLFELRYPLIDSVSKSLTQQLMPLYYFMNATARVIHSDTWGYFVNGSWSGMLGDMVEGRAELAGTLLFITEQRIEVQEFLTYPSSFPVRFIFHEPPLSYQNNLYLLPFNTSVWYCCGSFVGVMIVVLLINAQWETKKLKKESANTVLFPSVSEVTVFVVSAITQQGSTVELKGSLGRVVIFILFLAFLFLYTAYSASIVVLLQSSSNQIRTLSDLLQSKLEIGAEDTPYNRFWFAAAKDPVRKAIYEKKLAPKGSIPKFYGMVEGIKMMQNKPFAFHANLGVGYQIIQQYFQEHEKCGFQEITFLQDSTPWSSCYKLSPYKEIFKIGQIRIQEHGIANRINHLIFAKRPVCSVRGGRFVSVSIVDCYPSMLVLLYGLVLAVMILFVEILYDKRCSARKRVSASIVSSSRGTVSSSSGLPSVSIDF

>EposIR75b

MKLLFLLNFALCLSSIFSLDSDDISMIVSFSQQDGRSTSVLAPYVCWSSYEVATLAKSLHEVGVNAARSLQPRRTENYLQNLLILADLGCAGTDEFLIKANDEGFFKSPYRWLLITKDPEDLGILDRIVMLIDSDVVLAQKTSDGYKLIEVYKVIANSEVIFTTRAKWYPNGGTLKNTTTDGNNTALTMDDKITSITVVEDKYGVLEDHRPSKILSTRRMDIRKHTLTMVNVITDSNETRKHMDDRLQLHQDSITKMSYMVVKICFEMMNSTEKLIFTNTWGYVDKNGSWNGIIERLIKKEGDIGTLTIFTQERMKNIDYIAMVGTTAVRFVFREPPLAYVSNIFALPFTGAVWLAILVCVLACALFLYITSKWEATMGMHPLQLDGSWADVLILIIGAVLQQGCTLEPRHAAGRVVTLLLFVALTILYAAYSANIVVLLRAPSSSVRSLQDILNSPLKLGASDFAYNRYFFKKLNEPLRKTIYNKKIAPKGKKANFYTMKEGVEKIRRGLFAFHMELNPGYRLIQETYQEDEKCDLVEIDYINEIDPWVPGQKRSPYKDLFKINFIKIRESGIQACIHQRLHVGKPRCLGAVNTFSSVGITDMYPAMLATLYGMLLAPAVLLLEIAYKRLMEMREKRKAILQSDHDAT

>EposIR75q.2

MKTYCLMVSLIFFSGGCYAENETKLFMVSDVIQSMQRPSAVIAMLCWSSRMKMQLYSALGENDTEIKMMQFFKAGKVPQLDAQAQHVVFLADLDCPGISSYLTMSYLEKHFRSPFRWLLIGTGSKNTGNEISIPEALASVDLLVDSEVILAQHLEHGSYELHLIYKVGPNTEWKKELYGTWDKNKRLQTSLMEGELILRRLNLENYEIPISYVLTDNDSINHLFDNVNDHIDTITKVNFPTTNHLLDFLNASRKYVFTNTWGYRVNGTWNGMTGYLVREEVEVGGSPMFFTSERVSIVDYISSPTPTRSKFVFRQPKLSYENNLFLLSFRAAVWYCTIALLNLLVLAVFIAAVWEWKKTHGHENEKSETDASILRPSLMDVTLLILGAACQQGSTVELKGSLGRVVMLVLFLALMFLYTSYSANIVALLQSSSSQIKTLDDLLHSRIKFGVHETVFNKYYFSTATEPVRKAIYETKVAPRGSKPRFMSMEEGVKKMQKGLFAFHMETGVGYKFVGKYFQESEKCGLKEIQYLQVIDPWLAVRKNTPFKEMFKIGTKRIQEHGLQSRETRLLYEKRPKCSGQGGSFVSVSMVDCYPALLVLSYGSIVAIVLLFMEIMSAKKSFIYGKICRTTETED

>EposIR75e

MFLFVLMCICLANTRASHLNGDFVKDFLRREERPATIVSHLQLTKREQVHLSKTFFANNKQFQIALETPITNNEHVVHLADCRNNYTWEILQNAINDKSIRSPARWLLLLEDDLGNDLDSVQINDYKSDIERRLLDSYVFMDTEVYVACQRENHVELFSVYKTKPHLPLIWEEHGVWSQAGFSKPRPAPLAMRRRSLGGAPVVAATVILDNRTLEHMPDYLHREVDTLTKLMYYMTMHLVEWVNGTRVINRTGSWGYRQPDGRYDGVVREMQDGRADLSGTVMIPTIERSKYMNFVLPPAPVEAKFIFKKPSLASVTNIYVLPFSIGVWLSILVLIFVSSLTLFLSYYREKVVNPRYNMSWPQKVSEGLFETLCLVFQQGTAGDPISIAGRQILLLGLMAFMFLYTAYSANVVALLQSPTNDISSAETLLTSPLACGAQDVLYNRNMFLHETRPIHRALSSRKILPQGEKAFLSVDEGIRKVREGMYAFHVELTAGYDQIQKTFLEDEKCNLGAIKFMSITYPYLALAEGSHIKEQLRIGANRIMEGGVQRRTARRMLPAAPRCGAGAATFAAVRLRDVAPALRALLALYALALPVCALEVLARRRELRNAEKKADEEDTPEDISENFIS

>EposIR75d

MELASFITVYFAAKQLSLLTAFICWKPEELLVLQRETARAGLRLRTPRGAXALAPDGDRREAMLLDLXCPGAQLILDQASASRAFNLRHSWLLLDXGPFNASXVQXTLAXTLVLPDADVALAADDALVDVYRXRADQPLALAPLGAGPAEWAAXPAAPTRRKDLNNVYLKAATIISQPQHFKGWADLTMRHIDTFPKLTYPLMLLLAEDLHFRYNLKQVDLYGEERXGAFDGLAGQLQRAEIEVGITSMFMRXDRTRVLHYCSETVELRGAFIFRQPSKSSVSNVFLLPFSRGVWAATAAVLAAATALLAALARQARLRAVDPMLEQLSLGETVIFTVGTVCQQGFNLVPAIASXRVVMFCALMTSLFAFTSYSAKIVAILQTPSDAIRSIDDLTRSPMTLGVQETTYKRVYFAESTLPATQQLYRXKLLPLGERAYLSVVDGVERLRTGLFAFQVEEPSGYDIISKTFTEREKCGLQQIQAFKLPMVAVPVRRHSGYKELFATRLRWQREVGLMERSRXMWLAARPRCDAGAGGFVSVGLIDIISALHVLAAGMALSLVMLAAERAAQRCGPPRLRARRXEELVT

>EposIR75p

MSGRIIQWVMFASLMALYAAYAANIVVLLQAPSNSINNLAQLAKSKMTLAGYDADYNNFLFKGTTDPVRRAIAKRVDPERGPKPFYSLEEGVERIRKGLFAFHVVSDPTYRQVEKTFFESEKCDLVEIDYAGFKKFYMPAYKHSPYLELLRVVFKHIREVGIKSAINFRLETGKPSCKNAAAMFVSVGVMEMRSVMLFMVYGVGISXAILLVEILVFHLDRYRQNRSAVRVGQVNQVCVGITWQESPKLGGKGAIFGRNRRSMDTEIQPCSTLVEG

>EposIR76b

MAGMELIISSFCNATFCDVIYDSPSPLTEHQAEVIATKNELNGKHLKVATYDNYPLSWVATTENSTLNGRGVAFVIFDILREQFNFTFDVITPSQNFEIGGSEPEQSLIGLVNSTKVDMAVAFVPILYKYQSMVDFSSILDEGVWNMMLRRPKESAAGSGLLAPFEVHVWYLILAAVLSYGPCITFLTYVRSKMVKDDESHIPLSPSFWFVYGAFIKQGTTLAPEANTTRVLFTTWWLFIILLSAFYTANLTAFLTLSKFTLDIENPQDILKKNYRWVAPQGSTVQYTVGDVDSDLYYLNKMVANGRAQFPSVNADKQFLPLVAGGAVLVKEQTAIDHLMFADYVRKTKDGVAEADRCTYVVAPHPFMEKLRGFAFPRGSKLKPLFDPVLTYLLQSGIVKFLEHRDLPSTKICPLDLQSKDRKLRNSDLTMTYMIMVTGLAAAIGVFVGEMMIRRYVRVKMKNMRGDTKTKLPKNAAMTRRFKLDDSRPPPYDSLFGNNSRYKMTGKSKRKIINGREYWVVGTVNGESRLIPVRTPSAFLYQRDK

>EposIR87a

MRSRLFLLYLCFIHFAAAKTNPLLMPSEDSGKLVKAAECVVKMSAKYFVEHKALSGSIIIISMNSMVSIAQRAMIDTIHRGIKSTVMVKDSLFPHANASHFREIAKNYMFILESQQELTRDVIQLNKLPTWNPLAKAVVFYQLQPGEDGEQISIDFINLMRGYKLFKSIIFMFSPENEEVISYSWAPYSDTNCGGKCESVYILDKCTNGKVRQVHSQIDMFPLNMKQCPLVTYAVVSEPYVMPPVRKLTDTAYDDAYEFEKGGEIKLVKLISEFTNMSLIVRMSDVTENWGLIDANGTATGAFGVLRNDSVDLVIGDIEVTRTIRKWFHPTISYTQDEMTWCVPKSAQASTWNNLVIIFQWSTWVATFLSIVIMGLIFHYFYYRENNRKVTKWPTNSWLYTLSMLLGWGASFNPKSATFRILIFAWLFFGLIMGISYESFLRTFLMHPRYEKQISTASDLIRSEIPLGGRGIYRSYFETNNESSFYLYRKYVETSFSDGIRRAALGRNFAAVSSRRQAEYQDQKLGKGRPLLYCFKEGNNLYKYGVVLVAKRWYPMLDRLNNMIRRVSENGLIEKWNQELFIHSVSADSSGVVESLKIQHLLGAFMFIGLMYAFSVLVLIGELAMGSLDKRRNNKTKEKAVYRVKLI

>EposIR93a

MRIWLLVFCIAGVRSEEFPSLITANASIAVVLDRQYLGEQYQALLEGLKDFIKELTRVDLKHGGVVVYYYSWSTISLKKGFIAVFSIASCEDTWSLFSRIEEEELLLFALTEVDCPRLPPDSAITVTFTDPGQELPQLLLDLRTNKAFNWKSAIILHDDTLNRDMVSRVVQSLTSQIDDENIPTISLTVFKMKHEINEYLRRKEMHRVLSKLPVKHIGENFIAIVTSDVMSTMAETARDLGMSHTQAQWLYVISDTDNRRANLSMLINALYEGENIAYIYNITEDREDCRNGLMCYAEEMMTAFVSALDAAVQEEVDVAAQVSDEEWEAIRPSKLQRRDMLLKHMMQHITVNSKCGNCSTWRAIAADTWGSTYSGYVDTSDTGDNETTGVIEQINLLQVGYWRPIDGPRFTDYLFPHIAHGFRGKVLPVITYHNPPWTILKANESGSISSYTGLIFDIVNQLAKNKNFTLKLLFPGDIKNALSNKTSAEGTYSQSAMLTMMAVAKRQAAFAAASFTVLPDKNPGINYTVPVSTQPYAFMIARPRELSRAMLFLLPFTTDTWLCLGFAVVLMGPTLYIVHRLSPYYEAMGVTRQGGLATIHNCLWYIYGALLQQGGMYLPRADSGRLVVGTWWLVVLVVVTTYSGNLVAFLTFPKQEMPVTTVAELLDNRALYTWSISKGSYLEMELQNSDEPKYVSLLKGAELTSESSGMEGHLQTRSPLLMRVRSQRHVIIDWKLRLSYLMRAEHLATDTCGFALSTEEFLNEQVAMIVPAGSPYLPVFNKEINRMQKAGLIAKWLSAYLPKRDRCWKTSSIAQEVDNHTVNLSDMQGSFFVLFLGFVTATSVLLIEWFYHRRKSQKEDVAIKPYVE

>DmelIR25a

MILMNPKTSKILWLLGFLSLLSSFSLEIAAQTTQNINVLFINEVDNEPAAKAVEVVLTYLKKNIRYGLSVQLDSIEANKSDAKVLLEAICNKYATSIEKKQTPHLILDTTKSGIASETVKSFTQALGLPTISASYGQQGDLRQWRDLDEAKQKYLLQVMPPADIIPEAIRSIVIHMNITNAAILYDDSFVMDHKYKSLLQNIQTRHVITAIAKDGKREREEQIEKLRNLDINNFFILGTLQSIRMVLESVKPAYFERNFAWHAITQNEGEISSQRDNATIMFMKPMAYTQYRDRLGLLRTTYNLNEEPQLSSAFYFDLALRSFLTIKEMLQSGAWPKDMEYLNCDDFQGGNTPQRNLDLRDYFTKITEPTSYGTFDLVTQSTQPFNGHSFMKFEMDINVLQIRGGSSVNSKSIGKWISGLNSELIVKDEEQMKNLTADTVYRIFTVVQAPFIMRDETAPKGYKGYCIDLINEIAAIVHFDYTIQEVEDGKFGNMDENGQWNGIVKKLMDKQADIGLGSMSVMAEREIVIDFTVPYYDLVGITIMMQRPSSPSSLFKFLTVLETNVWLCILAAYFFTSFLMWIFDRWSPYSYQNNREKYKDDEEKREFNLKECLWFCMTSLTPQGGGEAPKNLSGRLVAATWWLFGFIIIASYTANLAAFLTVSRLDTPVESLDDLAKQYKILYAPLNGSSAMTYFERMSNIEQMFYEIWKDLSLNDSLTAVERSKLAVWDYPVSDKYTKMWQAMQEAKLPATLDEAVARVRNSTAATGFAFLGDATDIRYLQLTNCDLQVVGEEFSRKPYAIAVQQGSHLKDQFNNAILTLLNKRQLEKLKEKWWKNDEALAKCDKPEDQSDGISIQNIGGVFIVIFVGIGMACITLVFEYWWYRYRKNPRIIDVAEANAERSNAADHPGKLVDGVILGHSGEKFEKSKAALRPRFNQYPATFKPRF

>DmelIR10a

MAVLGTVFLLFMLDLKTLNLTRLNGLLVEPTRDLPQLELWLRAGSDHQDAENPYVQWFLLRTEIPLSIVTYQENRYWMDDPFGRRNLVLVMSLDQLLTNRGAAAPIQKASTFFYILADQDKDLSADEQLRLEGSCRQLWTQHKVYNRFFLTRDGVWIYDPFKRRDSAFGRLVRYYGSETLDKLLFRDMAGYPLRIQMFRSVYTRPEFDKETGLLTRVTGVDFLVAQMLRERLNFTMLLQQPEKKYFGERSANGSYNGAIGSIIKDGLDICLTGFFVKDYLVQQYMDFTVAVYDDELCIYVPKASRIPQSILPIFAVGYDIWLGFVLTAFACALIWLTLRVINLKLRIVSLGNQHIVGQALGIMVDTWVVWVRLNLSHLPASYAERMFIGTLCLVSVIFGAIFESSLATVYIHPLYYKDINTMQELDESGLKVVYKYSSMADDLFFSETSPLFASLNKKLSWNRDLRADVIDEVARFRNKAGVSRYTSLILESSHFTLLRKIWVVPECPKYYTISYVMPRDSPWEDAVNALLLRFLNAGLIVKWIQDEKSWVDIKMRSNILEADAESELVRVLTIGDLQLAFYVVIGGNLLAFLGFLAEHFRWKLQKKGV

>DmelIR52c

MVWLIIILFCLGNSSSQILDVTNNSHLDFDYRLFGLLQRLQVEKSYDTLLVYGEDCAIPSLFERLQVPAVLVSSGSTNFDWNFSSLTLILSCNFQDEREENYRTLMKLQTSRRLILLKGHIKPESVCDFYSKKEQHNVAMVKENFYQLEVVYSCRLFQDQNYEKLNLFDGKSIYKDQFRNMHGAPIRTLSDKEPPRTIPYIDSKTGEEKFKGYVGMLISQFVKKVNATMQIREDLIKDDEEVSFVDITNFTSNDILDIGICEARTLEMSNYDAISYPYLMSSYCFMAPLPDSLPFSDVYMAIVAPSILIMFLIIFCICSVLIIYIQERSYRSLTIRSVLMNDICLRGFLAQPFPFPRQYNRKLKLIFMLVCFSSLISTTMYTAYLQAFLWGPPIEPRLTSFDDVKKSRYTMAINIYEREFLEALNVSLEDVEIYDYGKFSKLRSTFNTNYLFPVTALQWFTINEEQKLFKYKIFYYCDAFCLNQFDILSIPLRRHLPYRDIFEEHMLLQKEFGLTKYWIDQSYRDMIRANLTTFKDFSPLLENDYIEVHNLYWVFTMYFVGMGMGLCFFILEILRPLRYWRNCKIKCEYCYAFLKNFAK

>DmelIR56a

MGSRFFIRNLILFGLLASSNMQIPFGELEKKFELDVDFLLGVTELVGHIQGLYSITVYADCIDIHPSIQQRIMDKFMVPVNTIGSNLSRPNYHKLDNSRIRIVLFTGLNDTILVNLNKTDVPYSDNFYMLAYASAIKNKCIELDFIEEVFTLLWKMSIQNAILLIRGEFMMEMWSYLYMGKIHKIKLTKPNSYLESLRKYNYRFSLEVINDPPAIFWYNSSEQADVTGGGNLSVSGPLGLIIINFLRHLNVTIDIVPIPGKQTSQYELFQQPDNLRAENGVNMVGSALLKYSPMVTQSRMCLLVSNRRMIPFSRFLDRLVSPGVHKLTFVSSIGIFVIKYFSHRPRSFVDAIFCTIRFFFAIPLPSIILNRLPVVDRFIEVFIIIFVQILLSSNISITTSALTTGFWEPPIINVETMRASGLHILTEDPTILQAFKENILPSSLADLVILVDEDTYFHHVTTLNNSYVYVVQAHNWQIFRLYQQQMTNEPFEIASEELCSKWRILGIPLNPKSPLRFMFKDYFYRILESGLREQWVHSGFKKFCEFNNLKKLPVDSVDSWQPLSIEFYSNVIRAYIIGLVIATLAFVAELLHNGYRRKNVKKT

>DmelIR11a

MRFAILWLFSGCLLPGIQVGIWVVVRAQPTGRDVLLSRLGNQQNELNTRRLANASSYLTRNYIANRINTLVVREICVECPYELSERQRQLVDQILASLAPELSVLLHKGTAEETTWEYTLFVVNDHTAFTGQVFIFPDELLEREFFCIVVVSEIQSRQFVRQTVGSIVKSNLQMHFVNVVVVAQLEDGTVGTYSYKLFKANCTPGITVRQINHFDRITGKPQQSMPDLYPVRNGHLGDCPFNVGAAHMPPHLIYKRHKDPPPASNVSIPAEDLAGIDWDLLQLLAKALKFRIQLYMPQEPSQIFGEGNVSGCFRQLADGTVSIAIGGLSGSDKRRSLFSKSTVYHQSNFVMVVRRDRYLGRLGPLILPFRGKLWGVIIVILLLAVLSTCWLRSRLGLSHPIEDLLTVIVGNPIPDHRLPGKGFLRYLLASWMLLTLVLRCAYQARLFDVLRLSRHRPLPKDLSGLIKDNYTMVANGYHDFYPLELTCRQPLDFSARFERVQRAAPDERLTTIALISNLAYWNHKHPNISRLTFVRQPIYMYHLVIYFPRRFFLRPAIDRKIKQLLSAGVMAHIERRYMQYENKRKVASNDPVLLRRITKSIMNGAYRIHGLVIVLATGMFILELLAGRSNGRLRRWMEWVHQ

>DmelIR7b

MKYWLYILSCCSLVASTMESSSDWDLAEALAQVVANSEMGRFKTLYIYTHTNSQSTGGHLEELLDQVLMIVPNNLQARRLLLQQSMEYKPYVHAVLALVDGLPSLSAIYARIRATQDLSHTLIYMSMPTDAYGEEMQATLRFLWRLSVLNVGVVLRPPGDHILMVSYFPFSALHGCQVISANVVNRYQVGTKRWASQDYFPSKLGNFYGCLLTCATWEDMPYLVWRPDGSGSFVGIEGALLQFMAENLNFTVGLYWMNKEEVLATFDESGRIFDEIFGHHADFSLGGFHFKPSAGSEIPYSQSTYYFMSHIMLVTNLQSAYSAYEKLSFPFTPLLWRAIGLVLILACLLLMLLVRWRHHHELPRNPYYELLVLTMGGNLEDRWVPQRFPSRLVLLTWLFATLVLRSGYQSGMYQLLRQDTQRNPPQTISEVLAQHFTIQLAEVNEARILASLPELRPEQLVYLEGSELQSFPALAQQSGSSARVAILTPYEYFGYFRKVHPMSRRLHLVRERIYTQQLAFYVRRHSHLVGVLNKQIQHAHTHGFLEHWTRQYVSAVDEKDESVARIASTSYSTLDGIDGDPSLSESEEDQQVAPVRQNVLSMRELAALFWLILWANLGAVVVFVLELLLPRIKLRKILRKMKSDIKKQISKLVRK

>DmelIR21a

MSYYWVALVLFTAQAFSIEGDRSASYQEKCISRRLINHYQLNKEIFGVGMCDGNNENEFRQKRRIVPTFQGNPRPRGELLASKFHVNSYNFEQTNSLVGLVNKIAQEYLNKCPPVIYYDSFVEKSDGLILENLFKTIPITFYHGEINADYEAKNKRFTSHIDCNCKSYILFLSDPLMTRKILGPQTESRVVLVSRSTQWRLRDFLSSELSSNIVNLLVIGESLMADPMRERPYVLYTHKLYADGLGSNTPVVLTSWIKGALSRPHINLFPSKFQFGFAGHRFQISAANQPPFIFRIRTLDSSGMGQLRWDGVEFRLLTMISKRLNFSIDITETPTRSNTRGVVDTIQEQIIERTVDIGMSGIYITQERLMDSAMSVGHSPDCAAFITLASKALPKYRAIMGPFQWPVWVALICVYLGGIFPIVFTDRLTLSHLMGNWGEVENMFWYVFGMFTNAFSFTGKYSWSNTRKNSTRLLIGAYWLFTIIITSCYTGSIIAFVTLPAFPDTVDSVLDLLGLFFRVGTLNNGGWETWFQNSTHIPTSRLYKKMEFVGSVDEGIGNVTQSFFWNYAFLGSKAQLEYLVQSNFSDENISRRSALHLSEECFALFQIGFLFPRESVYKIKIDSMILLAQQSGLIAKINNEVSWVMQRSSSGRLLQASSSNSLREIIQEERQLTTADTEGMFLLMALGYFLGATALVSEIVGGITNKCRQIIKRSRKSAASSWSSASSGSMLRTNAEQLSHDKRKANRREAAEVAQKMSFGMRELNLTRATLREIYGSYGAPETDHGQLDIVHTEFPNSSAKLNNIEDEESREALESLQRLDEFMDQMDNDGNPSSHTFRIDN

>DmelIR60a

MWCNNPGLIIIIFLGQILNLCQGIVNLSNETANTVIFMLPEKDLGPDVWKAGVGCLDSFAQIFFFRNPKERFTRAYNLMLVHAFHLSSPADQIQEGFSKLINEAVTNPGPPDREELFQMRVASDYNITNGTEDKGELILADNYVIVVDSVDRLKELMKKKIVEMRSWNPGARFLVLFHNATCRNRPLGVASNIFKDLMEMFYVHRVALLYANSTMNYNLLVNDYYSNVNCRILNVQSVGQCHDGKLYPNNAVVKASMQDYVSGFSPRNCTFFACSSISAPFVEADCILGLEMRILGFMKNRLKFDVNQTCSLESRGEMDGPANWTGLLGKVQNNECDFVFGGYYPDNEVADHFWGSDTYLQDAHTWYIKMADRRPAWQALVGIFEAYTWIGFILILIISWLFWFTLVMILPEPKYYQQLSLTAINALAVTISIAVQERPICETTRLFFMALTLYGLNVVATYTSKMIATFQDPGYLHQLDELTEVVAAGIPFGGHEESRDWFENDDDMWIFNGYNISPEFIPQSKNLEAVKWGQRCILSNRMYTMQSPLADVIYAFPNNVFSSPVQMIMKAGFPFLFEMNSIIRLMRDVGIFQKIDADFRYNNTYLNRINKMRPQFPETAIVLTTEHLKGPFFILVVGSCWAALTFIGELIIHRWRTQLVSTSEQQDRRSDKRRRRRRRRKPEKDNRWQRQVQVAPVVRFTPVKRRKVFQGQTSQK

>DmelIR68b

MKFLVGLLLQWYLPGIYALAEIACRIAVEQNVQVTYLYRCASCPASFDADYSALELDLYRCVGSRLPVITRNMEAHELEPFRRTDSLSIFQIPAAEKGDSLVRRILDMLNPHQRRKHMHKYLFVWPNAGRHQLLRLFRGSWAKKLLYGLAITGRENGTFDFDPFAWGGLQVIQRLDGEVPYARKVKDLRGYPLRFSMFTDPLMAMPRSPVETAGYQAVDGVAARVVGEMLNASVTYVFPEDNESYGRCLPNGNYTGVVSDIVGGHTHFAPNSRFVLDCIWPAVEVLYPYTRRNLHLVVPASAIQPEYLIFVRVFRRTVWYLLLVTLLVVVLVFWVMQRLQRRIPRRGVIQFQATWYEILEMFGKTHVGEPAGRLSSFSSMRTFLMGWILFSYVLSTIYFAKLESGFVRPSYEEQVDRVDDLVHLDVHIYAVTTMYDAVRSALTEHQYGLLENRSRQLPLGIATSYYQPVVRRRDRRAAFIMRDFHARDFLAITYDSQAERPAYHIAREYLRSMICTYILPRGSPFLHRLESLYSGFLEHGFFEHWRQMDLITRVGASPDAEEFLEDLGDQTDTDSGSNELAIRNKKVVLTLDILQGAFYLWSVGIGISCLGFAVEHAHWFWRRQTLRNAVEARTS

>DmelIR56d

MDNRAAELILRERNIFPTNGSDNITLLNNMFVLEMFYRITQLYHFKNFIFYISERLDLNNKDSQEFFHNFWTYFPMAPNLIITREHHLGIPMMQFISTPSLVMVFTTGKDDPIMELASHNQQGIHWLKTIFVLFPSLQSRDFETNPESLAQFTAEIKDVYDWVWRKQFINTFLITIKDNVFILDPYPTPSIVNKTGVWQAEEFFHKYAKNMKGYLVRTPILYDMPRVFKSDRPTNRYEKNFIHGTSGNLFLGFLEFVNATLMDTSANVTADYLNMTNLLDLVSQGVYETLIHSFTEITTKFVVSYSYPIGINDCCIMVPYRNQSPADQYMHEALQENVWVLISLFTLYITVAIYLCSPLRPRDLSAAFLQSICTLTYSVPTFIIRTPTLRMRYLYILLAIWGIVTSNLYISRMTSYFTTAPPVRQINTVQDVVEANLRIKMLAIEYERMAKSPLQYPESYLNQVDLVDKHMLDLHRDPFNTSFGYTVSSDRWRFLNLQQLHLRKPIFRLTEICEGPFYHVFPLHKDSHMRSVMTEYIMIAQQAGLMNHWERETFWEAVHLHRIHVHLFDDEPMALSLDFFSSLLRTWTLGLILAGLAFAAEMKWHEHVTFKRRPVIRITRKPRSFLRRFMKL

>DmelIR51b

MCKVLTLLVVILLLALTNAAYNVTLLKSVLSLISTREPWINTPIFVGHNTQGGDLNDLIIWLHQTMGVTSLTMNLFLQPEHIRPLGHFKITRYNGIALFFCHDKHDIMWLTLDRNLRKLRRIRLIIILRNQRSGSQGAIKSIFNALWQYQFLNVLVLQRDQLYSYTPYPAMRFFKLDIHTEPLFPHAARNFHGYVVSTPAENDIPRVFHVHDPLTKSRKVLGYAYRTFVEYLDHYNASLRLTNPDENLDPTTSVNMNHIVQLIIDGQLEISLHPYVFTPPTATKSYPLLIYPNCLIVPMRNEIPRHMYLLRPFQLYSWYILLFAVFYITGILYCISPKLNKSSWPQRLGLNFLDAISKILFISPPITIYRPTWRHLIIFLQLSVLGFMSTSWYNIELDSFFTTIVVGEQVNSMDQLVHQQQRVLVKEYEINTFLRHVEPRLVEKVSRLLVPVNASEQVSALLSFNRSFAYPFTEERWQFFAMQQQYAFKPIFRFSSACLGSPHIGYPMRVDSHLETSLNHFILKIQDTGLLNHWVVSDFNDAMRAGYVRFVDNVLGYQSIDVDTLRLGWCVLGIGWILSALVFSCEYWHLYPWRFIA

>DmelIR48b

MILQQSSNLLKLLLLLAISSVRTQGLNDIIIELNQRLLISNNFLYCNQSDKLNEYEIKYLQHMPPISLMIFTSIESMNFTQVEYNLGADNKLFLIMGNEEPPYDFLHALNLHFQFAEYIIVIDEPVDLKKSTKWLDFVNHLWQQGYVQLLIYTSYDEKLYHKIIFPETVIEETLVEQYISIRGSFNNLYGYPVRVAAYNNAPRSMLYVNRWGKHIFAGFYMRFLRAFIDARNGSFVPVLTPSNSPGNCTLNLVNETVDVCADALAANPAAFSLTHGFRIASANVLVTHAKPLHSYRYLTAPFQWSVWACLVIYVLLVVNFLSFIGWLRSGKWEFSKYLLEVFSSLLFSGFYLKEIRGRERYILFGVLFIAGFVYSTEYLGLLKSMLISEVFEKQIDTFEALVESNITLMVDPYDKILFAKYNMPEILSPIMELVSFETLLKHRNRFDQDYAYILFSDRMALYDYAQQFLKHPKLLRIPIDFSFLYTGIPMRKRWFLKHHLGRAWYWAFESGLTRKLALDADFEAVRVGYLSFLITEHVEAQPLNVDYFVMPAIALAIGYILALLSFVIEMTAWRIREFLGCRKATMTSTGCSEGGHVDVD

>DmelIR7a

MFHHLWLLMGLRSLAMGALHPPQPEAMTPLVAAALEILAEQVSPSQSTLAVMDLTQDAEHRDERQEQLMTIILRSVGSEMALRTFQKPPAEVPASFVVFLVNSAQAFNTLGFHFTDIHSTREFNFLILLTHRMSSRAERLQVLRDISRTCVRFHTSNVILLTEKRDGVVLVYAYRLLNMDCDLSVNLELIDIYKNGLFRHGHEARSFNRVLSLSGCPLQVSWYPLPPFVSFIGNSSDPEERAQIWRLTGIDGELIKLLASIFDFRILLEEPCNKCLSPDIKDDCSGCFDQVIISNSSILIGAMSGSHQHRSHFSFTSSYHQSSLVFIMHMSSQFGAVAQLAVPFTVIVWLALVVSSLLLVLVLWMRNRLVCGRSDLASHALQVLTTLMGNPLEARSLPRSSRLRILYAGWLLLVLVLRVVYQGKLFDSFRLPYHKPLPTEISELIRSNYTLINQEYLDYYPRELTVLTRNGSKDRFDYIQGLGKEGKFTTTSLIATMEYYNMMHWSTSRLTHIKEHIFLYQMVIYLRRHSLLKFAFDRKIKQLLSAGIIGYFVREFDACQYRKPFEEDYEVTPIPLDSFCGLYYISLIWLSAAVVAFILELLSQRIVWLRRIFE

>DmelIR64a

MHWWLLVFLPLSCQGLPEHELLELELDYGLAEPQRTSLLQSSLILQFSQDYKHIPRITYFTCQKPHLQTPNQIPNAAEHRDAFAAKNFQLIKSLYESELFVRIVLLDVLAQSPSSGRPNRPGSGPTGGFSQTPSQAQSNSEWLEGVLRMEALRQIAVVDLACGAVSRRFLELASAKMLYSEKFHWLLIEDFAWHGRTQTAEGSGKRDDGEMEEEEPPGQQIQATDDEDLPSIESFLGGMNLYMNTELTLAKRMSEAAHYTLFDVWNPGLNYGGHVNLTEIGSFTPTEGIQLHTWFRTTSTVRRRMDMQHARVRCMVVVTNKNMTGTLMYYLTHTVSGHIDTMNRFNFNLLMAVRDMFNWTFVLSRTTSWGYVKNGRFDGMIGALIRNETDIGGAPIFYWLERHKWIDVAGRSWLSRPCFIFRHPRSTQKDRIVFLQPFTNDVWILIVGCGVLTVFILWFLTTIEWKLVPHDGSALIKPKGGAPPRHHYQQQQQQEQVEAPVRPITAVSVVVSKEKVEEKQEEYEDSTPIDAGTLWQRCYQKLNKYIKDRKAKQKKAPERVGLFLESVLFFVGIICQQGLGFSTSFVSGRCIVITSLLFSFCIYQFYSASIVGTLLMEKPKTIKTLSDLVHSSLKVGMEDILYNRDYFLHTKDPVSMELYAKKITSVPTTKENEADEDEPVDPNPASTDPAKSYRDIVHSHETGAHAKDNAASNWLDPETGLLRVKHERFAFHVDVAAAYKIIAETFSEQDICDLTEVSMFPPQKTVSIMQKNSPMRKVISYGLRRVTETGILTYHFNVWHSRKPPCVKKIETSDLHVDMDTVSSALLILLFSYAITLMILGTEILYSKWHNRIQLKWVGAT

>DmelIR93a

MNPGEMRPSACLLLLAGLQLSILVPTEANDFSSFLSANASLAVVVDHEYMTVHGENILAHFEKILSDVIRENLRNGGINVKYFSWNAVRLKKDFLAAITVTDCENTWNFYKNTQETSILLIAITDSDCPRLPLNRALMVPIVENGDEFPQLILDAKVQQILNWKTAVVFVDQTILEENALLVKSIVHESITNHITPISLILYEINDSLRGQQKRVALRQALSQFAPKKHEEMRQQFLVISAFHEDIIEIAETLNMFHVGNQWMIFVLDMVARDFDAGTVTINLDEGANIAFALNETDPNCQDSLNCTISEISLALVNAISKITVEEESIYGEISDEEWEAIRFTKQEKQAEILEYMKEFLKTNAKCSSCARWRVETAITWGKSQENRKFRSTPQRDAKNRNFEFINIGYWTPVLGFVCQELAFPHIEHHFRNITMDILTVHNPPWQILTKNSNGVIVEHKGIVMEIVKELSRALNFSYYLHEASAWKEEDSLSTSAGGNESDELVGSMTFRIPYRVVEMVQGNQFFIAAVAATVEDPDQKPFNYTQPISVQKYSFITRKPDEVSRIYLFTAPFTVETWFCLMGIILLTAPTLYAINRLAPLKEMRIVGLSTVKSCFWYIFGALLQQGGMYLPTADSGRLVVGFWWIVVIVLVTTYCGNLVAFLTFPKFQPGVDYLNQLEDHKDIVQYGLRNGTFFERYVQSTTREDFKHYLERAKIYGSAQEEDIEAVKRGERINIDWRINLQLIVQRHFEREKECHFALGRESFVDEQIAMIVPAQSAYLHLVNRHIKSMFRMGFIERWHQMNLPSAGKCNGKSAQRQVTNHKVNMDDMQGCFLVLLLGFTLALLIVCGEFWYRRFRASRKRRQFTN

>DmelIR87a

MSTPEQRFWLAALLFLLSQHSEVRGFGINLMKVQTEDKGQEACILALLRKYFDSGDGLSGSVLCINRNYQLPNIEEQLLRGVNNYENYPWSLLITNSREGPSPAKFLMNEKPQCYFLIVDNLEDEDLDEVFEHWKGMVNWNPLAQFVVYLASLEETDEEMNDLMVELLLTFINKKIFNVNVIGQSEENQFYYGKTVFPYHPDNNCGNRVISVELLDACDYPSEETDSEDENDEDEGDGAQEEDDGPQEEGDGEQEEEDGPQEQEDGDQAKGDEGQENDDGGLENKVENEFRIGASDDDELENDLSSNSSEPEAIIEEFFRAKFEDKFPRDLSGCPLTASFRPWEPYIFRNSEEQPVDDYYYGLQGDEDDYNDTSPNYGESDDESYADPGEDGDGAIPDTETQSGGKLKLSGIEYEMVQTIAERLHVSIEMQGENSNLYHLFQQLIDGEIEMIVGGIDEDPSISQFVSSSIPYHQDELTWCVARAKRRHGFFNFVATFNADAGFLIGIFVVTCSLVVWLAQRVSGFQLRNLNGYFPTCLRVLGILLNQAIPAQDFPITLRQLFALSFLMGFFFSNTYQSFLISTLTTPRSSYQIHTLQEIYSNKMTVMGTSEHVRHLNKDGEIFKYIREKFQMCYNLVDCLNDAAQNEHIAVAVSRQHSFYNPRIQRDRLYCFDRRESLYVYLVTMLLPKKYHLLHQINPVIQHIIESGHMQKWARDLDMRRMIHEEITRVREDPFKALTFDQFRGAIAFSGGLLLVASCVFAFELCYVKYVYRTEKRERKTKKITKKVHNIKIQHD

>DmelIR7e

MNISALLNSYYDLSGEQMNHINEFVARAVLHVVHHYILSVTPSLVLTLCCRSNHTCNFYNKMMSTLFREWGLAPLQIVNVLRGVPWHPVPGRRHFNVIFTDSFAAFEEIRMEYYSREYNYNEHYFIFLQARDRLLQGEMRLIFDYCWRYRLIHCSIQVQKSNGDILFYSYYPFGEHGCSDMEPQLINRYNGSMLVEPDLFPRKLRNFFGCPLRCALWDVPPFLTLDEDQEEVLRVNGGYEGRLLLALAEKMNFTIAVRKVHVNMRDEALEMLRRDEVDLTLGGIRQTVARGMVATSSHNYHQTREVFGVLASSYELSSFDILFYPYRLQIWMGILGVVALSALIQLIVGRMLRERMGSRFWLNLELVFVGMPLLECPRSHTARLYCVMLMMYTLIIRTIYQGLLYHLIRTHQLNRWPQTIESLVQKNFTVVLTPIVQEVLDEIPSVQHMRFRLLEANSELDPLYFLEANHQLRQHVTASALDIFIHFNRLSADKVHQRGEQGSGAHFEIVPEDIISMQLTMYLAKHSFLIDQLNEEIMWMRSVGLLSVWSRWELSESYLRNEQSFQVLGTMELYAIFLMVLVGLIVGLLVFILELVSMRSIYLRKLFT

>DmelIR7f

MNTTSDSNAGSSLSSGSGYSIYKSYLENSRIDMQGEDANLYVARALRLVIENVLAQLSTTLVVTISTRHLGTAHWFEYMMNILMDSWRMVAVQLLRIRPDLVVNPVPGRKRVSLLMVDSYQGLLDTNITASNANFDDPDYYFIFLQARDHLIPKELQLILDHCLAHFWLHCNVMIQTAQVEVLVYTYYPYTADACQKAYPIPVNTFDGRKWKASQMFPDKLSQMHGCPLTVLTWHQPPFVELVWDPKHNRSRGSGFEIQLVEHLARRMNFSLELVNIALLRPNAYRLAEGSSEGPIEKLLQRNVNISMGYFRKTARRNQLLTTPMSYYSANLVAVLQLERYRIGSLALLVFPFELSVWMLLLLALLIHLGIHLPSARRGNEEDGGGGLQVVALLLGAALARLPRSWRHRFIAAHWLWASIPLRISYQSLLFHLIRLQLYNTPSFSLDQLLAEGFQGICTANTQRLLLEMPQLARDPDSIQSVDTPFDWDVLNVLTRNRNRKIFAVANQDVTLSFLHSSAHPNAFHVVKQPVNVEYAGMYMPKHSFLYEKMDDDIRRLDASGFIHAWRRASFASVHRKEQVHMTSRRYINHAKLSGIYMVMAGLYLLAGLLFAGEVLLRQRN

>DmelIR7c

MLHSAVHNVSLVYALVWAIDNYYGMATSTPLAVVQFPTSRESRRLHNDLIDAALGRSSGTGRIQFLLEDDRVEMTETDTDPPPPSGLTGRPIAIWFLDSLRSYFRLEMYLNQLGSPYKRNGFFLVIYTGLEDQPMESLKIMFRRLLNMYVLNVNVFLQRDGTVHLYTYYPYGPHHCQSSLPVYYTAFQDLAAPANGFGLTKPLFPRKLTNMHGCEMVVATFEHRPYVIIEDDPKTPGGRSIHGIEGLIFRSLAERMNFTIKLVEQKDKNRGEILPDGNFTGILKMMVDGEVNLTFVCFMYSKARSDLMLPSTSYTSFPIVLVVPSGGSISPMGRLTRPFRYIIWSCILVSLIFGFVLICLLKITALPGLRNLVLGRRNRLPFMGMWASLLGGLALYNPQRNFARYILVMWLLQTLILRAAYTGQLYLLLQDVEMRSPIKSLSEVLAKDYEFRILPALRTIFKDSMPTTNFHAVLSLEESLYRLRDEDDPGITVALLQPTVNQFDFRSGPNKRHLTVLPDPLMTAPLTFYMRPHSYFKRRIDRLIMAMMSSGIVARYRKMYMDRIKRVSKRRNLEPKPLSIWRLSGIFVCCAGLYLVALIVFILEILTTNHRRLRRAFNVINRYAA

>DmelIR8a

MELPLLVLLLALRFAGSEVLKITFWIEPVQRAEFDTDIAMVLKELDALRLDVKVDDTTLTLTRSEDGLDMQRFCEILSTVGASAVIDLTYSHWEEGYNLVRSLGIGYVRLERIMRPFLDMFGDFMRQKRANNVAMVFMNARDAVEAMQQMLVGYPFRTLIMDASQTDPGQHFLERIRSLRPAPTYIALFARAAAMNGIFEKVQKADLFQRPLEWHFVFLDTRDRVFKYRRQAELCTRFTLNPRAICRSMPMPDLYCGSGFTMQRAMLLNVLRSLINAAQVSPGYPLAIYQDCNATASSSEVSDPLEKDDYNWLDMVHWSNFLAYAPPLPHIQDQFQSPVPGLTFAVNISAGYYSSEHEAKTDLAAWSSVGEMRLLNETISPARRFFRIGTAESIPWSYLRREEGTGELIRDRSGLPIWEGYCIDFIIRLSQKLNFEFEIVAPEVGHMGELNELGEWDGVVGDLVRGETDFAIAALKMYSEREEVIDFLPPYYEQTGISIAIRKPVRRTSLFKFMTVLRLEVWLSIVAALVGTAIMIWFMDKYSPYSSRNNRQAYPYACREFTLRESFWFALTSFTPQGGGEAPKAISGRMLVAAYWLFVVLMLATFTANLAAFLTVERMQTPVQSLEQLARQSRINYTVVKDSDTHQYFVNMKFAEDTLYRMWKELALNASKDFKKFRIWDYPIKEQYGHILLAINSSQPVADAKEGFANVDAHENADYAFIHDSAEIKYEITRNCNLTEVGEVFAEQPYAVAVQQGSHLGDELSYAILELQKDRFFEELKAKYWNQSNLPNCPLSEDQEGITLESLGGVFIATLFGLVLAMMTLGMEVLYYKKKQNALEITQVRPVNDSSGSGGNSSTAPPTATSTTKQAWHIPVLEAEEKPAKVSPPPSFETATFRGKKLPARITLGDGKFKPRHGLYARRNLGASDSHSGYME

>DmelIR20a

MLASLNRSTGLSAELLDLYGLVVHFLLSGEHTTLVYFNPAGLDCSWGVLWQRNLTAHPQIVWQRNYSYPDLYYQFNAKLLVLACLPMDSRAAIQLEILANSLSHLRTVVRLLIEVAGPDQVTLARQYLSFCLRRSMLHVELYFRDYHHSLILYSFRAFPSFELVMRWISVGQGVKLFLHKLDDLRGHRLRVIPDLSPPNTFFYRDARGDNQVTGYLWDFLATFAGRLNAGLEVVRPSWRAGSASDSSYMLEYSAKGLIDVGLTTTLITKWNLWAIHQYTYPLLVSSWCTMLPVEKPLATPDLFGRIVCPTLAMTLLLIILVTWLVFRQLRCLTRLKNSRPARIVPHLLTLLLLTTCSAQLLSLLIFPPYHVRIASFEDLLRGDQKILGMRNEFYNFDGAFRARYAGVFYLIDDPNELYDLRNHFNTTWAYTMPYIKWLVIKTQQRHFSKPLFRWSKDLCFFDFMPTSVIVAPDSIYWESIKDFTFRIHQAGLMKHWIRKSFYDMIKAGKMSIKDYSDLETLKPLNIGDLEIVWRVCGAAIAVASAIFIMELLYFYINVFFNSL

>DmelIR31a

MNLLISMFILILAAGEGEIIPSMEESVVTNFVKSLVKTKQAIVFSCLFKDFKEISLALMRINQFVSVVNLNQSYSLTSILTRENYARTSVMVNARCSGSSELLFEASENRYFNKTYQWFLWGVDLEVQSLFPLNLNYVGPNAQITYVNETADGYAYWDIHSKGRHLKSNLEINLIATLINDTLNIARDIFHLQSIDFRGQFNGLTLRGASVIDKEDIISNEQIESILSRPTKDAGVAAFIKYHYELLGLLRERFNFTVNFRNSRGWAGRLGNTTFRLGLLGIVMRNEADIAASGAFNRINRFAEFDTIHQSWKFETAFLYRYTSDLDTHGKSGNFLSPFSDRVWLFCLLTLGAFSIIWVLFEIIDYKILRIRVNSQKLEHLNQKSSVICIKTTCIERILQTFGACCQQGLDPNPVDRSVRFLVMTLFLFSLVMYNYYTSSVVGGLLSSSDQGPSTVDEITASPLKISFEDIGYYKVLFRESQNRSITRLIEKKLSSSRSLNELPIFSHIEDAVPYLKAGGFAFHCEVVDAYPVISEYFDANEICDLREVSGLMEVEILNWILHKNSQYTEIFKTAMCNAQEKGFVERILRRRQIKKPACQSLYTVYPVSLSGVLPGFVILICKSINKFS

>DmelIR41a

MFIDLSWSLVLSAIVGKYLNESTICIFWNDKFEFQLLHKSDYISFVGINIKSFDDNGGHYIIDTGLKKKELQNKHLFLDELVIKIIISIEVTHCETFVVFDKDIDRFVNAFNKASVYSIWRSLHNKFVFAHIANESPESRNHFFEDQPNILFVVRDHSSASSFDIKTNKFVGRKAENPSQMILVDRYLASEQRFQFGKSLFADKLNNLQGREVIIAGFDYPPYTVIKHNMSTNAQDMGVSGESDFKNVYIDGTETRIVLNFCEQFNCTIQIDSSAANDWGKVYPNMSGDGALGMLINRKADICIGAMYSWYEDYTYLDLSMYLVRSGITCLVPAPLRLTSWYLPLEPFKETLWAAILLCLCAEATGLVLAYKSEQALYVLPGYREGWWTCTSFGVCTTFKLFISQSGNSKAYSLTVRVLLFACFLNDLIITSIYGGGLASILTIPSMDEAADTVTRLRFHRLQWAANSEAWVSAIRASDEALVKDILYNFHIYSDDELLRLAQDQHMRIGFTVERLPFGHFAIGNYLGPQAIDQLVIMKDDIYFQYTVAFVPRLWPLLDKLNTLIYSWHSSGFDKYWEYRVVADNLNLKIQQQVQETMTGTKDIGPVPLGMSNFAGFIIVWILGSAIATLTFLLELSLTYILKQSNLK

>DmelIR47a

MRQIKLLVWLLVVGVVSSTEQLQFLKNFLEAVHKERSISTILLIQRKVHKNDFLHGLYPIFWPIICLDETKRVELVNNFNKDFLALVYMESEADTLLLSALAADLNHIRDARIMIWLQMSPSENFLDRIVFQASKQKFLNLVVIENTLKTRRFYPFPQPKVQVIDKPFEEKEIYPALWRNFMGKNAIAVPDLVPPRSFNSFDPKTGHRRESGSIYNVFKAFTQRYNITMLLKWPLIRNTTQEEIIGKSVRGEIDLPITGQLISFRHPNGSRSQPLLGMTALSIAVPCGPELPMFDRFFLFYGLATPITITGYYVLLNTIEIILGTLSDRIKRHPRRKKILNLVLNLRVFSCILSLPTPQGNRLRSVKGQLTMVMSITGLILSCIVAAQTSTILTMKPQYRHIKNFQELSDSNITVVCNHLNYLTIKQQMDPKFMAKFMQNIWIVNSIEQMKMIFDLNTSYAYQTFSYKKDPFTLLQMHTTRKAFCRTPGLDLVSGLAYTAVLEKNSIYALALQDYTLKAFSAGLVYYWAEESIRDLISTVGRTQFEKLPIVIGYQSLKLQDYNVCWKILLIGGALAFCVFIVEVVVGLINRRI

>DmelIR48c

MSLLRIILIIIFLRIVSSIPDTIISHLSAELQIKIQIYFGLGNDLYDFSRLDGNYQKIIISHNISEEFKTYHDEPVLIIIRLERDLNLNLATLDVLRSYLTDRQYNDILLIDNDEENLNSYVDIRKAYWNAGFSQVLIYNSQQRTWSIKPYPYLQIRPTSLKEYIENRNTRNLMGYPLRVLVTNDPPHCFVDKDELPGSPNRYKGSIVTMLKIFADQLNATFQANPFREFRRYSTADCVQMVSDDEIDACGSIFIRTYTYATSQPVRLNRVVIMAPFGNPIEKFYYFFRPFDLYVWIGTGIIVVYIAVMGSLLHRWHFKEWNVGQYLLLAVQTLLNRELSLPQSSSGSKFMLLLLLFAIGFILSNLYVALLSMMLTTKLYQRPIENLADLKAANVNILLQTHNIRPNSVYGSSEELRERFLLVEESQHLEKRNGLDPSYAYVDSEDRMDFYLYQQKFLRRRRMKKLSNPVGYTWAVQVIKQNWVLEKHYNDHVQRFFETGLQNKLVDDVHELAVKAGFLHFFPTQTQTIEPLRLEDIVMAAMVLGGGHALAVICFLVELFA

>DmelIR52a

MALGWSVIILGFIGQLSAQILNYTQSRDLELLEGSLFRVLSRLNLEEEYNTLLIYGKECVFHSLLRKLEISAVTVPSGSTDYDWSFSTAILILSCGYDAENEENSYTLMKLQRTRRLIYLEDNSEPESVCMRYSLKEQHNIAMVKSDFDQSDTFYSCRLFQTPNYVEGHFFKDQPIYIENFQNMRGATIRTVADSLVPRTILYRDEKSGETKMMGYLGHMINTYAQKLNAKLHFIDTSKLGAKKPSVLDIMNWVNEDIVDIGTALASSLQFKNMDSVWYPYLLTGYCLMVPVPAKMPYNLVYSMIVDPLVLSIIFVMLCLFSVLIIYTQHLSWKNLTLANILLNDKSLRGLLGQSFPFPPNPSKHLKLIIFVLCFASVMITTMYEAYLQSYFTQPPSEPYIRSFRDIGNSSLKMAISRLEVNVLTSLNNSHFREISEDHLLIFDDLSEYLVLRDSFNTSFIFPVSVDRWNGYEEQQKLFAEPAFYLATNLCFNQFMLFSPPLRRYLPHRHLFEDHMMRQHEFGLVTFWKSQSFIEMVRLGLASMEDLSRKRNEEVSLLLDDISWILKLYLGAMFISSFCFILEILRCGERCKRLWRCRW

>DmelIR52d

MVRIIIILLCLGYTKARILDATNTNHTDLEERLLSLLLRLQQEQFFNTLLIYGEDCAFSSLSRRLQVPTILVSSGSTSFEWNYSSLALILTCEFKAEREENYQTLKKLQMNRRLILLNGNIKPDSVCDFYSKKDQYNIAMVNNNFHQVGIIYACRLFQERNYEKVYLSEGNPIYVDQFRNMQGALLKSITFNLIPGSMAYRDPKTGQEKHIGYVANLLNNFVEKVNATLDMQVKLHKAGKKTSFYNITKWASEDLVDIGMSYAAYFEMTNFDTISYPYLMTSTCFMVPLPDMMPNSEIYMGIVDPPVLVVLIAIFCIFSVMLNYIKQRSWRSLSLVNVLLNDICLRGFLAQPFPFPRQSNRKLKLISMLVCFFSVITTTMYTSYLQSFMWGPPIDPKMCSFADLENSRYKLAIRRYDIEMLRPFNVSMDHVVVFDESSQLEYLRDSFDDNYMYPMSALSWSAFKEQQKLFAFPLFYYSEKLCLKPISFFSFPIRRHLPYRDLFEEHMLQQNEFGLSTYWIDRSFSDMVRLKLATMNDFSPPRLEDYIEVSDLSWVFGMYFTGLGISCCCFGLELLGLPSWTRRLRLTNWLRVRN

>DmelIR54a

MWTVITGIVLWAPVLVAGSAVDFIFRAAAEHSLSVIMIRIDYCPYNWAKDIFENQTIPVVVLSDSETFINIRMFSRPLHVACLPGHELQKDLALLENFTSSLMDFPSQKKIVYISNNFSDPTRMDYIFETCYHRRIWNIVGLLASDEHRYFYRYHLYPSFRTEYRSLESSTIFDKDFPNMHGHPLTVMPDQWLPRSVLYVDRRTGKQILAGSVGRFFHVLSWKLNATLQLSKKVTTGRFLNATALKELSESFSVDVPASLTIMERVEQLASTSYPMEVTHVCLMVPVARRIPIKDIYFILSSASNMFLAIVIVSSYGLALNLLRNMTHRDVRLVDFVLNDKALRGILGQSFNLPLSRSFSTRLIFLMLGIVGLNVSSIFGAGLDTLMAHPPRQFQARSFAGLRRTKIPLVTTEEDFPTWMKLRVPMLVVNVSEYNHLRNGRNTSNAYFASRLYWNLFSEQQKRFTRELFIYSTDDCLWSLALLSFQWPQNSLFTEPVSQLILEVNANGLYDFWVGMHYYDMTAAGLSGLEDPSLQLKEREHPTSLRIVDFQWMWQAYGTFMVIAILVFLLEVSWHRITSLFVSLVY

>DmelIR60d

MRLAIYVAFLSSIGNRSGFLSSLLMSLGKELHYKTILLVGGSSTCWSLEPFETGVPILNLRGENNAYPQDTFNSQMLALACLQTESEDAVKLLYRSLKDMRDTPTLLFASSEEHIHDTLFLGCFRENMLNVLALTASSKEFIYSYQAFPTFRVIKRKLVEIHRYFEPQLKDLGGHIVSALPGNIMPRTMCYRNAEGERQLAGYLNTFIRNYVESINGTLRISWGLVPEDDMRHLTISRLSKIQHVDFPLGIIPLYNKTDKQHVYMEISSWFLMLPMETSVPRAHLFVKLGLERLLPIIVVVGAVLGNAHRIEVGLGPSWRCYYLADKVLRGALAQPIVLPRRLSPKLMLIYSLLLLSGFFLSNYYMASLTTWLVHPPASDRILEWDQLRYLHLKVLTIPEEFKYMSLILGTDFMTAYGSIFQLTNSTDFQRRRISMDPSYAYPVTTSLWPFLELSQVRLRRPLFRRSYDMVLQPFQVMSLPLPRNSIFHKSLLRYAALTRETGLYYYWFRRSYYELVALGKISYKEEEGNPYCDLKWNDFRIVWLAFLGGTIISCLALLLEVAHYRWHLGNSSL

>DmelIR60e

MVIKMISFLLVSVLLCLVGASDSESMQVQVLQDLNLALQTELNVFIDFECCATSEILHKLDSPRILLSSNSREARDLRIRGNFTESTLIIVSVMDSDLNPLVASLLPRLLDELHELHIVFLSNEEPGFPKQDLYTYCFKEGFVNVILMSGKGLYSYLPYPSIQPISLSNVSEYFDRARIIRNFQGFPVRILRSTLAPRDFEYSNEQGGLVRAGYLFTAVKELTYRYNATIESVPIPDLPEYDVYLAVAEMLHTKKIDIVCYFKDFSLEVAYTAPLSIIREYFMAPHARPISSYLYYSKPFGWTLWAVVISTVLYGTVMLHLAARGARVEIGKCLLYSLSHILYNCHQKIRVAGWRDVAIHGILTIGGFILTNVYLATLSSILTSGLYDEEYNTLEDLARAPYPSLHDEYYRSQMKAKTFLPERLRRNSLSLNATLLKAYRDGLNQSYIYILYEDRLELILMQQYLLKTPRFNMIRQAVGFTLESYCVSNSLPYLAMTSEFMRRLQEHGISIKMKADTFRELIHQGIYTLMRDDEPPAKAFDLDYYFFAFVLWTVGLISSLLVFFAELVSGHL

>DmelIR67a

MLPILVPVLLLFNETSWINPILTSIYKDRHHETVLLLQHSQHGNASGLERFPWPVFSFNEQMDFYVRGKYNSEMLVLIWQTGNSDWDLDLWQALDRSLLNMRKVRVLLLRKWEKIPTADVAATAEHLLFLHVAVIGQGNRIYRLQPYAPQSWLQVDPIESPIFIKIRNYFGRYIVTLPDQFPPRSIVYRNPKTDEIQMTGYVYKFLLEFIRIYNFTFRWQRPIVQGERMNLILLRNMTLNGTINLAISLCGFETPSELGVFSDVYDMEEWYIMVPRAQEISIADVYVVMVSGNFLIVLIIFYFIFTILDTCFGPLLLKERVDWSNLMLNERMISGIMGQSFNMSARNTISSKVTNATLFLLGLVLSTLYAAHLKTLLTKRPTSQQISNFKQLRDSPVTVFFEEAERFYLKHAWDRPIRYIKDQLNFRETIEYNALRMGLNRSNAFSALTSEWMIVAKRQELFKQPIFTVQPELRVIQTSVLLSLVMQSNSIYEDHINDLIHRVQSAGIVEYWKHQTLREMITMGMISQKDPFPYVAFREFKVGDLFWIWLLWVSFLFMSFVIFLCELLVDCFISKTLIRNKRPH

>DmelIR75a

MQLVQLANFVLDNLVQSRIGFIVLFHCWQSDESLKFAQQFMKPIHPILVYHQFVQMRGVLNWSHLELSYMGHTQPTLAIYVDIKCDQTQDLLEEASREQIYNQHYHWLLVGNQSKLEFYDLFGLFNISIDADVSYVKEQIQDNNDSVAYAVHDVYNNGKIIGGQLNVTGSHEMSCDPFVCRRTRHLSSLQKRSKYGNREQLTDVVLRVATVVTQRPLTLSDDELIRFLSQENDTHIDSLARFGFHLTLILRDLLHCKMKFIFSDSWSKSDVVGGSVGAVVDQTADLTATPSLATEGRLKYLSAIIETGFFRSVCIFRTPHNAGLRGDVFLQPFSPLVWYLFGGVLSLIGVLLWITFYMECKRMQKRWRLDYLPSLLSTFLISFGAACIQSSSLIPRSAGGRLIYFALFLISFIMYNYYTSVVVSSLLSSPVKSKIKTMRQLAESSLTVGLEPLPFTKSYLNYSRLPEIHLFIKRKIESQTQNPELWLPAEQGVLRVRDNPGYVYVFETSSGYAYVERYFTAQEICDLNEVLFRPEQLFYTHLHRNSTYKELFRLRFLRILETGVYRKQRSYWVHMKLHCVAQNFVITVGMEYVAPLLLMLICADILVVVILLVELAWKRFFTRHLTFHP

>DmelIR75c

MTSWPLYRLIVFNLLEINLSNLMVFHCWSIKEAFPLVEMLNQNGIFSQYIDVQNPDNLANVHKEYLDSDLVRLGVFLDLGCDKAELVTNQSSRARLYNQNLHWLLYDEAGNFTKLTQLFEGANLSLNADVTYVSREDEERFILHDVYNKGSHLGGKLNITVDQTLQCNRSHCQVKEYLSELHLRPRLQHRMDLSSVTFRLAALVSVLPINSSEEELLEFLNSDRDSHMDSISRIGNRLIMHTQEILGFKLHYIWCGTWSVQDAFGGAIGMLTNESAELCTTPFVPSWNRLHYLHPMTEQAQFRAVCMFRTPHNAGIKAAVFLEPFMPSVWFAFAGLLIFAGVLLWMIFHLERHWMQRCLDFIPSLLSSCLISFGAACIQGSYLMPKSAGGRLAFIAVMLTSFLMYNYYTSIVVSTLLGSPVRSNIRTIQQLADSSLDVGFDTVPFTKTYLVSSPRPDIRSLYKQKVESKRDPNSVWLSPEEGVIRVRDQPGFVYTSEASFMYHFVEKHYLPREISDLNEIILRPESAVYGMVHLNSTYRQLLTQLQVRMLETGITSKQSRFFSKTKLHTFSNSFVIQVGMEYAAPLFISLLVAYFLALLILILEICWARYAKKKFSTIIPQNQ

>DmelIR75d

MKVQVAHWLPLIFFLLVSGTPRVAGSWRSEYSRQDPDPKTRWGNQLPDMLVAYYRHHGVHSLMLVVCHTDIADFRLWKLWQHFNLNNFYVQVSTESSLRDLQHVDALDEHKDAPPPKSFHANNSTHWETSFLLPALPYKMGILLLEFSSECALNLLRWSAASEHNYFTTNRFWLLLTEDPGDIDLLEDPEIFIPPDSELRVLHYENVGNFSCSLIDLYKVAAWKPLKRTLVGHNIRNSRHVIHALQHFGSAITYRQDLEGIVFNSAIVIAFPDLFTNIEDLSLRHIDTISKVNHRLMLELANRLNMSYNTYQTVNYGWRQPNGSFDGLMGRFQRYELDLAQLAIFMRLDRIALVDFVAETYRVRAGIMFRQPPLSAVANIFAMPFENDVWVSILMLLIITTVVLVLELFFSPHNHDMSYMDTLNFVWGAMCQQGFYVEVRNRSARIIVFTTFVAALFLFTSFSANIVALLQSPSDAIQSLSDLGQSPLEIGVQDTQYNKIYFTESTDPVTKNLYHKKIASKGENIYMRPLLGMEKMRTGLFAYQVELQAGYQIVSDTFSEPEKCGLMELEPFQLPMLAIPTRKNFPYKELIRRQLRWQREVSLVNREERKWIPQKPKCEGGVGGFVSIGITECRYALGIFGCGAAVSFVLFLFEFIFRHFKQVYRIIKGYREVQR

>DmelIR76a

MENLLVESYYFSTVLSFFAQQFFADSHATCIFWHPAFDFRLETVHPMPLIIMDWHRWANRSDQDVYDYKIKEDEFEGKGIPYNDWTLRLTVAIERSHCETFIAFQEQIPEFARYFYHASIYSIWRSLRNRFMFVYTKEFEDKKDSYLSGYIFQDQPNILVITSQYLNSSTFEIKTNRFVGPRNFNKNPEPVEFYILQRFDAKGTKATWETQSAMSSKMRNLKGREVVIGIFDYKPFMLLDYEKPPLYYDRFMNTTDVTIDGTDIQLMLIFCELYNCTIQVDTSEPYDWGDIYLNASGYGLVGMILDRRNDYGVGGMYLWYEAYEYMDMTHFLGRSGVTCLVPAPNRLISWTLLLRPFQFVLWMCVMLCLLLESLALGITRRWEHSSVAAGNSWISSLRFGCISTLKLFVNQSTNYVTSSYALRTVLVASYMIDIILTTVYSGGLAAILTLPTLEEAADSRQRLFDHKLIWTGTSQAWITTIDERSADPVLLGLMEHYRVYDANLISAFSHTEQMGFVVERLQFGHLGNTELIENDALKRLKLMVDDIYFAFTVAFVPRLWPHLNAYNDFILAWHSSGFDKFWEWKIAAEYMNAHRQNRIVASEKTNLDIGPVKLGIDNFIGLILLWCFGMICSLLTFLGELWRGQG

>DmelIR94e

MDCPKWILSGLCLISLVSGATVIELLGTLKLELDFEYVLLMKNRNFSLSDQVWNGTSLTKDVMDEVQVPVLQFNENVSYFLHNSISRRLVTLGFMSDANLDEHRGLLTALVANLRHMTTSRVIFLVQSKASTDFLYELFRNCWRKKLLNVIVIFQDFETTSTFYSYSNFPILQIEERIYETSLQTLPIFPDRLRNLHGYEMPVILGGTAPRMIAYRNKKGNVVYDGTVGHFMTAFQQKYNVKFVQPLQAKNPLDFAPSMQTVGAVRNETVEISISLTFPTIPPFGFSYPYEQMNWCVMLPVEADVPPFEYYTRVFELAAFLLTLGTLVLISCLLASALSLHGYATNISEFLLHDSCLRGVLGQSFVEVFRAPTLVRGIYLEICVLGILITAWYNSYFSSYVTSAPKQPPFRTYDDILASKLKVVAWKPEYAELVGRLLEFRKYETMFLVEPDFNRYLALRDTLDTRYGYMITTNRWVLINEQQKVFSRPLFQKRDDFCFFNNIPFGFPLHENSVFMEPVQKLIMELAETGLYYHWITTGFSELIDAGEMHFVDLSPHREFRAMQIQDLQYVWYGYAFMVVLSSLVWLLENLAYTVKSKTIFPTHFMQRNKK

>DmelIR62a

MYLQFLFALFLSRYQIVATENFDRAFELALFLDRIGRVHRLHAITIVNSLGSVDPSYLDDLHRGLMCNSSNHFYMLPQMTATDKDSSHVHFSSLQDEETIYLVFARDSKDAVIYLQAERARGRRYTRTMFLLRKQESQKDIKYFFELLWKLQFRSALVVVAARNFYQMDPYPTVRVIRMRRLSSYDPHHVFPPANRKNFRGYRMRLPVQQDVPNTFWYKNRRTKAWELAGLGGILINQLMMHLNVTMDLFRFEVNGSSLLNMAALTDLIVKGKVELSPHLYDTLQSNTSVDYSYPTQVAPRCFMIPLDNEISRSLYVFLPFSLTMWLCLLFVLLVVHFVYVRRLIPDGHFWAILGVPGAGQVRYGNRKPVRRFSTFLILFGIFILGQTYSTKLTSSLTVTLIRRPDNSLEELFLLPYRILVLPTDVYAIVDSLGHAEQFSTKFSCTDAENFSQKRISMHPEYIYPISTIRWRFFDMQQRFLRKKRFYFSKICHGSFPYQYQLRVDSHLKDALHRFLLHVQQAGLHDLWLDTCYRKAHRMGYLKDFSTLAELEEKLRLRPLALNLLVPAFSLFLCGMLGSGIAFLVEIRHSFGCRQKPPSINRNPGD

>DmelIR76b

MATGIELLVAAALCVACPPLNDSPPTNLIQMGENGTLSPVTELPMDVDASEAGFDADAPVETLETINRKKPKLREMLDWIGGKHLRIATLEDFPLSYTEVLENGTRVGHGVSFQIIDFLKKKFNFTYEVVVPQDNIIGSPSDFDRSLIEMVNSSTVDLAAAFIPSLSDQRSFVYYSTTTLDEGEWIMVMQRPRESASGSGLLAPFEFWVWILILVSLLAVGPIIYALIILRNRLTGDGQQTPYSLGHCAWFVYGALMKQGSTLSPIADSTRLLFATWWIFITILTSFYTANLTAFLTLSKFTLPYNTVNDILTKNKHFVSMRGGGVEYAIRTTNESLSMLNRMIQNNYAVFSDETNDTYNLQNYVEKNGYVFVRDRPAINIMLYRDYLYRKTVSFSDEKVHCPFAMAKEPFLKKKRTFAYPIGSNLSQLFDPELLHLVESGIVKHLSKRNLPSAEICPQDLGGTERQLRNGDLMMTYYIMLAGFATALAVFSTELMFRYVNSRQEANKWARHGIGRTPNGQSVAPSRWLRGWRRLNSGHGQLLGASTHGQNVTPPPPYQSIFNGGSHGDPLNRWRRPLANGNALGNGVLLGGDSEGGVRRLINGRDYMVFRNPNGQSQLVPVRSPSAALFQYSYTE

>DmelIR85a

MSIQWLKHILLLAILVNLAGTRENHIPLDLKKSSIVMVKMSQILCKARIKVLFVYFENQTSHEHTGQILKEVTKCDISNQNTPLEAVKDDGILMYMVMITTNISQPLELSLIRKKSAAKHRSHVFLLVRDADTVSDAWMRASFRQFWKIWLLNIVILYWRDGRLNAYRYNPFMDNYLIPVDNKPNEVPTLEQLFPKTIPNMQRKPLRMCIYKDDVRAIFWRQGTILGTDGLLAAYVAERLNATMMITRPHSYNNHNLSSDICFLEVAKEYVDVAMNIRFLVPDTFRKQAESTVSHTRDDLCVIVPKAKTAPTFWNIFRSFGSLVWALILVSVLVANVFCYILKSEVGRVPMQLFAGALTMPMTQIPPNHSIRLFLIFWLYFGLLICSAFKGNLTSMMVFQPYLPDINQLGALARSHYHIIIRPRHVKHIQHFLTLGHKHESRIREQMLEVSDTQMYEMMRNNDIRFAYLEKYHIARFQVNSRVHMHLGRPLFHLMNSCLVPFHAVYIVPYGSPYLGFLDSLIRSSHEFGFERYWDRIMNSAFIKSGVKVVNRRRGSGNDEPVVLKLQHFHAVFALWLVGIGMACIVLAWEHLTHNYNLAVTKRRD

>DmelIR56c

MQHLLNLLAPFGRMNVFQEIVWFVSPHQRLDQLDEFIMRIDEAFGKSATQTVVNNNTEMRMIYSSARRNHMSFVFTTGAEDPIMKVFSKVLLGRHFYVSMVIYVDKVGDMHPIYDLLTFAYNQQFFNSMVHFESMEGVNQLFGVSKFPVMSFENRTDFLKYMGKIWKQVQNARSDVGGFGFTTPLRQDLPHLFQSQGHYDGSTYRIIETFVRFINGSFKELIMPPDSLGGQVINMKDALQLIRERKMEFCAHAYALFMSDEELEKSYPLLVVQWCLMVPLYNSVSTYFYPLQPFDWNVWFFALGALLALVLLELMWLRMFGGWSGYRGAVLNSFCYIINVPIEGQLQQPCLLRFLLLATVFFHGFFLSAYYTSNLGSILTVNLFHAQINTMNDIVSAQLPVMIIDYEMEFLLNLNKELPQEFLELLRPVDSAVFSEHQTSFNSSFAYFVTEDHWEFLDEQQKHLKQRLFKLSSICFGSYHLAFPLQMDSSLWRDIEYFTFRIHSSGLLNFYARSSFGSALHAGLVQRMPDTQEYTSAGLQHLAIAFILLLVMSFLAGIVFVLETLSR

>DmelIR75b

MLQLHNLILHNLIHMAKLSHVLILHCSLSHLALLAQSKNIFTQFQPLHSDIQLNDDFLNHNILKLGVFLDINCDKSGTVLDMASAKRFFSHRYHWLIYDRSMNFSVLESHFKEAQIFVDADVTYVTHDPFSKNFLLYDVYNKGRQLGGELNITADREIFCNKTNCRVERYLSELYTRSALQHRKSFTGLTMRATAVVTALPLNVSIKEIFDFMNSKYRIQLDTYARLGYQARQPLRDMLDCKFKYIFRDRWSDGNATGGMIGDLILDKADLAIAPFIYSFDRALFLQPITKFSVFREICMFRNPRSVSAGLSATEFLQPFSGGVWLTFALLLLLAGCLLWVTFILERRKQWKPSLLTSCLLSFGAGCIQGAWLTPRSMGGRMAFFALMVTSYLMYNYYTSIVVSKLLGQPIKSNIRTLQQLADSNLDVGIEPTVYTRIYVETSEEPDVRDLYRKKVLGSKRSPDKIWIPTEAGVLSVRDQEGFVYITGVATGYEFVRKHFLAHQICELNEIPLRDASHTHTVLAKRSPYAELIKLSELRMLETGVHFKHERSWMETKLHCYQHNHTVAVGLEYAAPLFIILLGAIILCMGILGLEVIWHRHCTLH

>DmelIR84a

MIKLQVKVISWPLIILTAFLRVLQIESINTNFLELAAFEDFLRSEHLSHVLVVRGDDADGDWKIECHQKLLANYRVQFYRPEMSANFEDLMFYGSPRTAVLVLNSEHVLVRRQVFGVASEAGYFNNSLAWFILGSGRESLPVEQLIDQLLSGYRMGIDADITVALRGPDNASMLFYDVYRISRQANTPLIIEKKGLWTHSGGYQKFGNFKNTWVIRRRNFLNVTLIGSTVLTEKPPGFGDMEYLADDKQLQQLDPMQRKTYQLFQLVERMFNLSLAISLTDKWGELLDNGSWSGVMGQVTSREADFAVCPIRFVLDRQPYVQYSAVLHTQNIHFLFRHPRRSHIKNIFFEPLSNQVWWCVLALVTGSTILLLFHVRLERMLSNMENRFSFVWFTMLETYLQQGPANEIFRLFSTRLLISLSCIFSFMLMQFYGAFIVGSLLSESARSIVNLQALYDSNLAIGMENISYNFPIFTNTSNQLVRDVYVKKICKSGEHNIMSLQQGAERIIQGRFAFHTAIDRMYRLLLELQMDEAEFCDLQEVMFNLPYDSGSVMPKGSPWREHLAHALLHFRATGLLQYNDKKWMVRRPDCSLFKTSQAEVDLEHFAPALFALALAMVASALVFLLELFLHWLPDFRRRLGTMST

>DmelIR92a

MLLQPLVMHLSQLLRIIVGQYFAEFPSILIVYNNSASTTPLQLEYLSALELVLRELSKPIRLQWINVAFLKDLNDLEDQVMGALNSSVTEGFITILSQTHHFIHARYYATRNANVRLKDKRYLFLCEDESPAELLCMDILQFYPHHLMVRPGTETAPTGPTGPHPDPRRGGGASVSTKNKDDGEGGAGNKTTSPYRDINFELWTQKFVGAVGNLDALLLDAFLPNETFANRVELYPNKLLNLQRRSLLVGSITYVPYTITNYVPAGQGDVDPIHPQWPNRSLTFDGAEANVMKTFCQVHNCHLRVEAYGADNWGGIYDNESSDGMLGDIYEQRVEMAIGCIYNWYDGITETSHTIARSSVTILGPAPAPLPSWRTNIMPFNNRAWLVLISTLVICGTFLYFMKYVSYRLRYSGTQVKFHHSRKLEKSMLDIFALFIQQPSAPLSFDRFAPRFFLATILCATITLENIYSGQLKSMLTFPFYSAPVDTIEKWAQSGWKWSAPSIIWVHTVQSSDLETEQILARNFEVHDYSYLSNVSFMPNYGFGIERLSSGSLSVGDYVSTEALENRIVLHDDLYFDYTRAVSIRGWILMPELNKHIRTCQETGLYFHWELEFIDKYMDKKKQEVLMDLANGHKVKGAPQALDVRNIAGALFVLAFGVAFAGCALVAELLIHRMDLSK

>DmelIR94b

MSLIFNLLFILILSQAVSQETEFLQLKYLNNIVRSMIKLHKMETLVIVKHHLDNNCSLQNWNAHGMGIIRTNDQGKLIMKDTFNSRTLAIICIGQNSHITLLRNVFETFGKVQQKKIILWTQMELKEKFFQEISKKSRDLKLLNLLVLKAVTKDKLLIYRLNPFPSPHFKRIENIWTPNDTLFMDTKFNFHGMTAVVKHDYNWTIQMGNIRKFPISRIEDKEVIEFALKYNLTLQFFNDVERFDIELRKRIILKSNSTQPIDSGIPMVFSSLLIVVPCGNYLSIQDVIKVSGIEKWIFYIILVYVIFVLIEITFLGVTILISRQSRHQMIPNTLVNLCAFRAILGLPFPETRRTSLSLRQLFLAIALFGMIFSIFINCKLSSMLTNPCPRPQVNNFEELKTSGLTVVMDHDAENFIEKEIGVDFFNQYMPRKVTLTFTERAKLLFSLKGNHAFTLFSESFAIIESYQRSKGLRAHCTSEDLIVAERVPRIYILENNSILDRPLRRFIRQMQESGITNHWLKNIPSSLEKNLMQITIPYDRERVHPLSIEHLTWLWCILILGYSISMIVFFVEMSLKRRKKNLENRAPNICIC

>DmelIR94c

MSKVFKLLVLPLIYLSLTKGSKNPQLKFLRELINVIEEGREIRTIMVIKHSRDEYCHLDQWNPRGSPILRTNEMGSIRISGYFNDQAVILACMGENSDYGLLKSLANAMDNMRQERIILWSEREPTKMLMDYISQQADRYNFAQIIIVTMNEDVDAVPSLHQLNPYPTPRFRQITNISNIRRTSFFGCGLSFQGKTAILKESVVSNIRFKVWSPSGPIPLSELKDYEIVQFAVKYNLSLKLYDQNESKSDHFDIQLGPLFITKDFPTQMAFVSPNTACSLIVIVPCSPKWRFMDVLHKLGVLKLIGCLLIAYAVFVLIETLILWLTHRISGREVRLTSLNQLLNPRAFRGILGLPFPEFRRSSISLRQLFLVISVFGLVYSNFVSCTLSALLTKPAQNPQVRNFKELRDSGLITIMDKYTHSFIEKHIDPEFFDHVLPHYLILQKKEALRMIWNFNDSYSYVMYTTTWKSLNTVQKSFDERVFCESESLTIAWNLPRMYVLGNNSVLKWMLSRYITYMPQTGIPDSWTEQLPKVLKLLYNVTSPRRIKEGAVPLSIQHLSWIWHLLFIGESIATLVFIVEILLQKSNQHTSNMRERSSEDDDFV

>DmelIR94h

MLSNISFSSAPELVDLYGLVLKFLVSSETTLFYFNPTGQKCSWETLPRTILSNHPQIIWFREETYPGLYKRHSSNLFVMACLSSTSYDGQLQLLAESLTRYRSVRVLIEVQDKEGSFLASQILLLCQQHSMLNVVLYFSRWTRTLNVFSYLAFPYFKLLKQRLSGSLRPKIFINQLKDLQGYKIRVQPDLSPPNSFSYRDRHGECQVGGFLWRIVENFSKSLKGDTQVLYPTWAKAKVSAAEYMIQFTRNGSSDIGVTTTMITFKHEERYRDYSYPMYDISWCTMLPVEKPLSVEILFSHVLSPGSALLLILAFILFFLIVPQLIKCLGITFRGRLIGMASRIFALVMLCSSSAQLLSLLMSPPLHTRIKSFDDLLTSGLKIFGIRSELYFLDGGFRAKYASAFHLTENPNELYDNRNYFNTSWAYTITSVKWNVIEAQQRHFAHPVFRYSTDLCFSSETPWGLLIAPESFYREPLQHFTLKINQAGLITQWMTQSFHEMVRAGRMTIKDYSRTNLMKPLRIQDLRKCWVIFAVGLGTSTVVFTIELLLIYTNVFLNSL

>DmelIR94g

MSTAVNSVHSKLVSLISRGQELTSIFFYAPAKEKCHLEDTISSATWGLPLVIWRTDRTVILNGFIGEGLLVLACLPGFHWRALLGSLARSLKYLRQARILIELMQDRDEFLVSEVLQFCLSQDMINVNAIFDDFPETENLSSFEAYPSFEVVNQTFTPDTQVSDLYPNKMLNLRGGVIRTMPDYSEPNTILYQDKEGNKEILGYLWDLLEAYAHKHNAQLQVVNKYADDRPLNFIELLDAAQSGIIDVGASIQPMSMGSLSRMHEMSYPVNQASWCTMLPVERQLHVSELLTRVIPYPTLALLLLLWIFYEVLRGRWRRHSRLQSIGWLVLATLVSSNYVGKLLNLFTDPPSLPPVNSLAALMESPVRIISIRSEYSAIEFTQRTKYSAAFHLALHASILIGLRNAFNTSYGYTITSEKWKIYEEQQKRSSKPVFRYSKDLCFYEMIPFGLVIPENSPHRAPLHSYTLLLRQAGLHDFWVNRGFSYMVKAGKINFTAVGERYEAKTLTITDLRNVFIIYVSVLLISLILFTCELFVSWVNYWLGF

>DmelIR94f

MWQQVLLAETSNWFRSDVLQRFWTHLRVEIRFRTMLNYRLESCDCWFDNVLGSDNSTALLWNDQTYPHYLRRRQDTDILVVSCLRFHQYQEVLLALSLMLDQMRSMPVVLQLCGDEDSMQELNSARLLLKHSQDLKMPNVVLLSSTFFTSATLYSYEMFPEFNVQKLVYQAYLTLFPYKLGNLKGHPIRTVPDNSEPLTIVRKTLNGSIAIDGLVWQFMIEFAKHINATLQLPIEPHPEKSIKLVQILDLVRNQTVDIAASLRPYSLNVQRSSTHIYGSPMMVGNWCMMLPTERVIGSHEALTRLMKSPWTWLILLLFYSVHRFLAQKTRLRSSLIHLIKLLINLSLICFLQAQLSAYFIGPQKVNHISNMQQVEESGLKIRGMRGEFMEYPIDMRSRYASSFLLHDLFFDLAQYRNSLNTSYGYTVTSVKWELYKEAQRHFRRPLFRYSEEICVQKLSLFSLIQQSNCIYCYRSRIFILRMHEAGLIRLWYRRSYYVMVTAGRFPIGDLSTVHRAQPIRWTEWQNVVLLHGVGLLFSVVVFVIELTVHYANVCLNNL

>DmelIR100a

MATTLQLIMLALVGGTLGQANNTDHKQVLTSIVKQLEGGLELHLRTSEDGGNDLVQFLMQEKSSIIISAKQEEVPSRAKIMRHHFFIFDGVHQMQEIRTSLFNTDGFYILALENNTIEDDVLLMEFAADVWLQHGHSRIYYVQLSKKSVLLFNPFLQRLVVVQDSKTYSRIYKDLEGYHLRIYIFDSVYSSVIGDGENKVLSVTGADAKLAKTVARQLNFTADFVWPDDEFFGGRLANGEYSGGVGRAHRGEVDIIFAGFFIKDYLTTHIQFSAAVYMDELCLYVKKAQRIPQSILPLFAVHMDVWLCFLLVGLLGALVWLILRAVNLILGIEGVPDGSRATRISYFGAARRIFVDTWVIWVRVNVGRFPPFHSERIFVASLCLVSVIFGALLESSLATVYIRPLYYRDVNTLRELDESGQPIYIKHPAFKDDLFYGHNSEVYRRLDAKMMLVAEGEERLIEMVSKRGGFAGVTRSASLQLSDIRYVMTKKVHKIPECPKNYHIAYVLPRPSPYLEEVNRIVLRLVAGGIVGLWTGEAKERAKWSIQRFPEYLAELDVGRWKVLTLSDVQLAFYALTIGCLLSAIVCMAEILLGRQRRLHSPK

>DmelIR40a

MHKFLALGLLPYLLGLLNSTRLTFIGNDESDTAIALTQIVRGLQQSSLAILALPSLALSDGVCQKERNVYLDDFLQRLHRSNYKSVVFSQTELFFQHIEENLQGANECISLILDEPNQLLNSLHDRHLGHRLSLFIFYWGARWPPSSRVIRFREPLRVVVVTRPRKKAFRIYYNQARPCSDSQLQLVNWYDGDNLGLQRIPLLPTALSVYANFKGRTFRVPVFHSPPWFWVTYCNNSFEEDEEFNSLDSIEKRKVRVTGGRDHRLLMLLSKHMNFRFKYIEAPGRTQGSMRSEDGKDSNDSFTGGIGLLQSGQADFFLGDVGLSWERRKAIEFSFFTLADSGAFATHAPRRLNEALAIMRPFKQDIWPHLILTIIFSGPIFYGIIALPYIWRRRWANSDVEHLGELYIHMTYLKEITPRLLKLKPRTVLSAHQMPHQLFQKCIWFTLRLFLKQSCNELHNGYRAKFLTIVYWIAATYVLADVYSAQLTSQFARPAREPPINTLQRLQAAMIHDGYRLYVEKESSSLEMLENGTELFRQLYALMRQQVINDPQGFFIDSVEAGIKLIAEGGEDKAVLGGRETLFFNVQQYGSNNFQLSQKLYTRYSAVAVQIGCPFLGSLNNVLMQLFESGILDKMTAAEYAKQYQEVEATRIYKGSVQAKNSEAYSRTESYDSTVISPLNLRMLQGAFIALGVGSLAAGVILLLEIVFIKLDQARLWMLCSRLQWIRYDRKV

**The amino acid sequences of GRs used for building phylogenetic trees are listed below:**

>CpomGR1

MGVMPIMRVPRDAQTTKRTTYNWISKATFWAYLVWSLESIIVVKVGRERYENFQKSSNKRFDEVIYNIIFLSILIPHFLLPIASWRHGPQVAIFKNMWTHYQLKYLKITGTPIVFPNLYSLTWGLCFFSWGLSFAVILSQHYLQDDFELWHSLAYYHIIAMLDGFCSLWYINCNAFGTASKGLAQNLHKALEADHPALMLAQYRHLWVDLSHMMQQLGRAYSNMYGIYCMVIFFTTTISLYGALSEILEHGLSYKEMGLFVIVGYCMTLLFIICNEAYHASRKVGHEFQVRLLNVNLGAIDHSTQREVEMFLVAIAKNPPIMNLDGFTNINRELFTANISFMSTYLIVLMQFKLTLLRQGARKAVRAIVKAIFNTTTMLPDEEYEDEE*

>CpomGR2

MIPDHYFDEGLNGSLYPDDMKQLNTVKLVYEKTQADYEQEQRDMLSSQDGDTCETHDQFYRDHKLLLVLFRALAVMPITRSRPGTITFSWKSRATMYAISFYIVATVVVLMVGYERVMILRSIKKFDEYIYAVLFVAFLVPHFWIPFVGWGVAHQVAIYKTSWGKFQVRYYRVTGENLQFPNLQTQIVIISVGCLLLAVCFLLSLCALMDGFLLRHTTAYYHIIIMINMNCALWFINCKGIKIASQSLSECFRRDVNVEISAKLISRYRFLWLNLSELLQSLGNAYARTYSTYCLFMFFNITIAVYGALSEIVDHGFGFSFKEMGLFVDAAYCSTLLFIFADCSHKSTLKVAAGVQDTLLGIDVLAIDRPAQKEIDHFIQAIEMNPAVVSLKGYAHVNRELLTSAISMIAIYLIVLLQFKISLPKADS*

>CpomGR3

MAFYTNNSLFPNQPPIPNGIAAQMDEKSKNKIIFLDVTPNRTPRLPTPNNAIAPIQDNLINPDITRDIIYENIKPVFTLLKIMGVLPLSRPVPGVTQFQPTSPSMLYSVVVYCSLIGYLLYLSLNKVQIVRTGAQEGKFEEAVIEYLFTVYLFPMIAVPILWYETRKIAEVLNGWVEYEIAYKKLSNRVLPVGLYKKALAMSIVIPALSTASVIITHVTMVHFKLLQIIPYVFLEILTYMLGGYWYLLCETLSICAHILAEDFQQALRNIGPAGKVAEYRALWLRLSKLARDTGIANCYTFTFMSLYLFLIITLSIYGLLSKISEGFGVKDIGLALTAFCSIMLLFFICDEAHYASHNVRLNFQKKLLMIELSWMNADALTEVNMFLRATEMNPSQISLGGFFDVNRTLFKSLLATMVTYLVVLLQFQISIPDDSRVQEADDDDDFVNATASVTEAPTTLTTITTLLTTLAKKKKKH*

>CpomGR4

MNASMTIFFQIMAKIFRFSRWFGVAGSGNVLWKTFGLCILLLLGVIEGVAIWRVVKALAGLAIDIEGHRSVTARLAGATFYASSITTLILSWKLSSSWETIASYWASIDRSIAINVSSDKKIKTRMITVTSVMVTCVVVEHAMSMMSQVGFECPPSLILKRYTLMSHGFLLLRTDYSIWFAVPLLFMSKIATILWNYQDILIVLISMGLTSRYNTLNQYVAKFSTLSKDPWNPHGECSKGHTWRRIREAYVKQAQLVRQLDQSLGGLILLSNLVNFYFICLQLFLGITQGLSGDLIKRLYYVVSLVWLCVRVSCVVLAAADINVHSTKALRHLHASDRHYYNVEIVRLQNQLSKDYVALTGLGFFSLNRTVLLQMAGAIITYELVLIQFDDHGFTEHKSALNSTTF*

>CpomGR6

MRISYPWVFSFMPYSIPLGIITQFLHFQATFIWNFSDLFVICTSYYLTSRLDNVNRKLLIAQGKYLPASFWRSAREEYSRVTQLIRKVDQVISGIVFISFANNLFFVCLQLFNTLENGIKGTGACRASRLKGTTLFAGYEGPAYFIFSLVYLISRSVAVSLIASQVNSASLLPAPVLYDVPSPVYCIEVQRFIDQVNADNVALTGLQFFTVTRELLLTVAGTIVTYELVMLQLTPSAQVPTANGTMT

>CpomGR8

MKSTQSYNFLEIKKRNNYVEDEELHAEGALALLLRLCCWARVAPRRVHTRSGWRFETFASLASLQAFGMTLLNAAIITSIILDFYQEPEKRLRVGATFLKTVIWLTEIALVMGIASLAVYTGPAQVECLKRVLKQLQKINSDLNLNNPSAKTEKIKSIIMVILLTWVVFIMIMDVVFYYPHSLEEGTVCILCLQLPFYVAHLLWWQAVLRWALTVEAVHGAAATVNHRLQSFRLATMKPVSMTLDEFLSKPRSIGTLLNCIRDPALTAKTTELGIQNFTYPAQVKTLIRRLALSYERIGDIMRQMNETNGLLLMIILTTTFMKLVVTPYYMLIYALDDESNVVFDMMLSLNWSLAILAILVLTIEPCHRVHSQRERTEVLLRQLTTHLAPSRQLSKELEQFTKLIVLNKPRFTALGIYTLDRPLMAMMLSGITTYLVIIIQFQKFSHKLEYD*

>CpomGR9

EGPRICIDVPPVCVVSGALALLLRASSYAGVCPLRFTQTHDGWRPSPSAPLAAVQRLIMTIFNALMLAAFILDICQEPGQYIRIGETTLKMFVWCSDMLLMMMIASVAVYMAPKRMNHLVHMLDQLRQVSTELKMNPSARNEKIKSIAIVFIPLWAASILIADFYSFLGPLMNGKMWYIMCMYGPYYVGNFMGILVLLQWSCAVLAVHATVVAVNDELATLRRAKFDLGPTTTLEDLLRPPKPERNTLVGCFTKPAKSPGNSMTLPQAQATIRRLAFSHERISELMRQLNASNGVFLMFVLMSTFIRLVLTPYYLLQRFDHDERILYELLLQINWTLFHVITLLLTIEPCHWTQEQRERTQILLSHLIVHLAPKCERLSKELDQFAKQILLSGAKYMPLGVYTLARPLMATILGGVTTYLVIIIQFQKISDQL*

>CpomGR10

AALILDFQEDPSKRIHVGESAVTACVWISDLGLVLAIASLAVYRGSARMKKFIKLLRELHKINDDLHNTKCVKMEKIGVIAVTSFLMSAMIIQVAQIYLLTKLFINRGCNWSIMLMYSSYYVANCLGLLALLQWGFVVLAVYSAAATVNQHLLRLHHVKLKAKETAMSEVYLSPPKPRVDCFIEGHYDIAPPDFAAYPLQLQSMVRRLASSYGHIGELMRQMNETNGTIIIAILMAVFLHLLVTPYYSLRALNSNANVLEVVLVPLCWTFLQIAILLLTVEPCHWTHEQRETTKFLLSRVTVRLAPKSKLLARELDHFAKQICLSNIKFSPLGVLTLGRPLVASMFGGVATYLIILVQFYSHTDD*

>CpomGR29

MGYTSDLVLRFLRNYFPLKGILLVRAFFGHYFSFKCPKIYLKLHKVYCVVVTLVCVIIIFIMTNEWRKWVIFELVIMTLASILIEGDCCGKFLSFVECTDHSFGLGRRNLASPRLYAAFFIITSIRLYIDYINIHAFFVNPVLYSAFTFLYTGLDLNHLLRIIVFDILYERAKHLRNHFESVFSRVNGDDNMISEVKRGILLYKELIGSVTMLEKIQVTYLLALVARFAANVADIHLILCVEKRDEMLLGKMCRLGSESLYLAALVCAPAVIMELVHNEVDKITSILTIQHAIATDRELGA

>CpomGR30

RYFTYTIRLFNLKCTNFYKVFFPIRLLFNRDVIFFNNFAMEGILNFLKIYLPLNRILFIRAIFGHHFSFDCRMLYLNIHKLYCIFVSFIFPLIIYSFSDVLESTRYAIFMEFFVCIWITLIVEDNCFQEYLTSIKRTDQLITHGRFNLASYRLYMVYFFITLLRMIIHFMSVKFFSISLYKFLTYAFVYMTLDLSNIIRVLIFEALYQRMIFLRNHFESIFDRPTNDCRNIISEVRRGLLIYGQLLDSVKLVNKIQVTLFITLALRFVGFALKLNMMLFKVNSIWDHTRMFMYTFESAFILALDLTPAVFSELTHNEVEKIETMMSHKHSMCTHRSLRAALSKGILYFKLRPFEFKIWRVIPVDSTLIFSFLSVFLTVSLLIFQFHRMM*

>CpomGR55

VNYKNRDVEALLKQQMKRFRIQLALSISIVNSITLMYFIEQYKSGKFSEMISTLIVIVFDLTLEYRFYFENIVFFVLIDILVELLKYLNQSILSSIEKLNKDDIDEVGDTGRIANELEVWSEIIRLLAIACHRLQVCFGGQVLLSFFTTILYYIMYFYQGILYSIKQELLWDSSISSVIMALSMYIAMKFVIVWSGQRAQNEAETLEANLTKLQTLLVKKRNLSRVLK

>CpomGR58

MNKNYKNNNGHVGCLLENYIEKEILDANWPLFFCQSVLLMPPFCVTNGYVTPVDKKYYIKILLGVCLHIAARVYYYICLYESFLQISMNPFVIFMATANSATYCIAIIVIYFVNVIQSHNNLQVMLKLRQALFTINLEKQEILKDHKIWNFIYIIGLVSIEVVVSICYCRMEQRMSFIFSKITFLVCDINLVCLYRTVSFGASLLASWNRKMMIYTNQIVTKKEIESMFNAYMHIIDALGLCKK

>CpomGR60

MILDNVIDREFQMMLLPLNILEILYCQPKFRITETFITPNGIRENLLCTLGVLLMILANVGYVSINSYIPGNDEISDIIHSFICTDAAFYIVYCLLMYAMNIIYKNQIVQLIIKMQKAYRVLQNENGLRRFQKSNWIFVVAVFLFYFTYNLSYTIFNIYRVTHLLYDVVLFYFDVNIIVAIRIVKFLEYELILWKKELNKFLKTCSTTNHNQLVKYL

>CpomGR61

MENSRNIIDEKILDALSPLIFAQNFFLFPKFMITERCIAPIAPRSYTSSFVGAVLMLLIRIYRLVTVCFYNYFGENSDALLLANFVVGCFGTIFSYVINVVQSANAVYMVIELQEALWCLSSNIKQSLSDYKFWNIVNIACIFGGYILYTGLFGVANQETHGEASFLVSHLVSITYDLNIILATRTVILTASILEAWNSKMSEILSEETEVRENCSQDMFSAYEKIINAFNLCKKAYQFGIFYHTFQTFHSILYSMQLFLEYAKSASHEELKVFGLLRGVTYFAWNSKNFLLLVNVSVACERFYAALRDAE

>CpomGR63

MRVSGMRVISADRRSTELQNTFKPIKTLTSIVSLNCSGPNKSWQLFWIVLKALASASVLGCLTFYCLYIKIRYHYNDVILSIKLTDVIQMSYDYSQYLVDLFFVFKYGRDTYAEYDKQLINIDQILISTNYSAIKRRHINLIVYFIAIWIFSSVCDFTAWAVSYGSLLPTLYSTSYIYLLIKMISTLDLMSHVMHVEYRLKGIVNQLQECYCDTKPFPGDFSDPIGKKFWFYCESPSKPGNTNETPPDRTLVCNSPQAVRWLSRCYLLLCEQCVFINSMFGTRILLNSLSLLIDMIRFTNIAVRLVIGSQPTMYASGNYPAAANVLRMV

>CpomGR68.1

MIQFTVISFYYVLVLMVVGVLKNINEQMKSIYCSNRVNAQFIKVEKIITLNQIEVVYVHMLEMKREINRAFQASILATAIQCFHSIVSESHILYHGLVVEHTLTTHDVCNCSIWIVYQLIKIYIISCSGSMLKEQVSKIGRSLHNILPGKDDARLYLEVQHFSSMILYQNAEMTVYDFFPLDATFTFNVISAAVMYIVMLVQFDATKKS

>CpomGR68.2

MFTSLQKYFSPVVNEDEELCFLQIFKPLYIVLSALGLFPQAVRFPDGIQNTTLNIKNSVINSMCTLFMIVIVHAFLVFHLQELNISSKDNSMTEDNMTLMNYIIGLVLEILFCTVSYFCVIRDRNLYITMLNDMAVCWDKLAMGKRRLILGRLRVHINCVVLTTVLAMILVLAVATYTSYLGVWKMILITLTFVLPDLIQFTMIAFYLVLMLMVVALFKNIEEEFKVISLVKNNAPNDLVEAHLVVSIREIREIYVKTMEIKRRINEAFQAPILVAMMVCFLELVSMPHMIYHGLSFQANFTMHDAVECTIWVLNQLLKMYALAKSGALLNSQVNEIGRTIHNIPISGDKDLKLYLDVLHFSSLMTYQDTAITIYGYFPLDSTLVFNIVASAAMYLVILVQFDKPE

>CpomGR68.3

MSSALTKYFSPVVNHEEELCLLQIFKPLYVLLSALGLFPLSIKFPDGIYKTNVDLKNSTINSAFTIFMIIVIHGSFVFHLQELNISSKDNAMTESKMTLINFTVGLIIQVLFCTVSFFRVMYDRKIYITILNDMADCWERMAMGKRRLILGRLRVQVNCVVLPSVLLSFLLILISQYTEIDLNIWKLILISLTFDLPELIQIAMLTFYFVLVLIIVALLKNIEEELILILHVAKNNRNYPVEADMRMDMGEIMKVYVKTLGLKRQVNAAFQTSILVALMSTFHLLVSLPHLMYHGLTFQTNFSTHAIIECSAWAVNQLIKLYFLSRSGDLMTSQVNEIGRTIHNIPISGDLDWKVILEVQHFSSLMTYQDAKMTVYGFFPLDATLSFNMFASAAMYLVILVQFDKPE

>CpomGR68.4

MFSSLRLYFSPFVKKNEELRLLQIFKPLYLVLSFLGLIPCSLDLPQGNVDCIILHKSAFKHSCSAFLTLLIVYVFFGLHVYEVLTSHEENILADDKMAKANYIIELVTQFTFCNATYFCAFRYKEIYVSILKEITRSWDDLPYVNRGIILGHLRVKVNCGVIGTICLILLTLTAVTYAGSSSLWKRILITMSFNLPEMIQFILVAFYYVFVLMVVALLKNIEDHCRMFMKARRSIKNCSKVELGRIPITLSQMQCVYVKALRVKRQINVVFQAPIMFSLLQCFHTMVSESYDICQGLLYQDNFTTHNLVECSYWVLLQMLKIYALARSGSLLKLEALKIGRTIHNIRSDDEEIKLLVEIQHFSTLMAFQSTEITIFGYFPLEAPLMFNMVAAAAMYLIILVQFAKTH

>CpomGR68.5

LKIHKYLFLIIYFSISSSCTKHARNMFAALKKYFSPVVHQNEKLSLLQIFKPIYVLLSTLGLFPQAILFSDDGQDATFAWCRALFMIILIHSFYIFHLHELYIFNKDNSITQGNMTLTNYIIDLSLQVLSCTVSYFHVIRDRNLYSRMLKDMAGLWDRLAKGRRRQILGQLRVQMCALLCPGTVIIPLLLAITYRGSLRVWKKILFTVTFILPELIQFLMISFYLVMILMIVALLKNIEEEIKILALRNNICYNLLEADELGMSILKIKNVYVKTLKIKRQVNAAFEALILVALTVCFHELVGLPHMIYHGTVYVPNFSINNTIGLSLWVFTQLLKMSALAISGALLKSQVRSLRS

>EposGR1

MKSCSQPRSLASFLDTILTANLTLRPDSEPGCVVSGPTAVVLRASRVFGAAPLSVSRARAAWQIGPSSSQATLQRVLITACNIIMLAGLGWDYSQEPDRRIRAGETTLKGLIWVFDLFLVMLIANITVYTGPCRMANLIQILKQLQQVLRSDDLCTIIISIDKLSPQTNVFHIHSQILFFILFYINFNIHDQRANTLAKSINSDYIRRANNVHVLHILLRPCCGRDDGAAVALHGSRRFDAISIVSSYLWLRITLPDRAPMQASQAQATIRRLALAYDRISEVVTMMNAGNGLILLVLILSIFYRLVLTPYYLLLGLLGDDEIVLSWVVLQINWGVTLMINLMAIVEPCHWTQEERDDTEHLLGQLTVYLAPKFERISKEVDQFAKQVKLDNTKFTLLGIYTLGRPLMATILGGVTTYLVIIIQFEKISDLC

>EposGR2

MGVMPIMRVPKDAQTTQRTTFNWISKATLWAYLVWGFECIIVIKVGKERLDNFQNSSNKRFDEVIYNIIFLSILIPHFLLPIASWRHGAQVAIFKNMWTHYQLKYLKITGTPIVFPNLYSLTWGLCFFSWGLSIAVIMSQHYLQDDFELWHTFAYYHIIAMLDGFCSLWYINCNAFGTASKGLAQNLHKALEAEHPALMLAQYRHLWVDLSHMMQQLGRAYSNMYGIYCMVIFFTTTISLYGVLSEILEHGLSYKEMGLFVIVGYCMTLLFIICNEAYHASRKVGHEFQVRLLNVNLGAIDHSTQREVQMFLVAIAKNPPIMNLDGFTNINRELFTANISFMSTYLIVLMQFKLTLLRQGARKGVKTIMKAVFNTTTTLADEEYEDEE

>EposGR3

MNVNEGVHSTWTDRTSAEVQETLKPLHHVTKFFSLNCCPKSKTRWQKTSSVAKATACAAVLGFVNFYTLYIKSRDVFDSINMSIKLTDAIQMCYDYCQYLVDLYFVHKYGSGVSLEYFKRYADIDQVLGITVYATIRSRLVKLIVYFIVIWLVSSVIDFTAWTLSYGPYTPTLYAISYIFFLIKMLNSLDITSHVMHIEFRLTYIKDQLQDCYCSTRSLTGNLNDATCNQKWFYCENTTRISKSSIYPESLVAHRNNHQVIKWLSKCYLNLLEQCQFINKMFGIRILLNSLSLLIDMIRFTNIAVRLIIGSQVTRYDPGYFPAVANLLRMLTCALVIGSLVAHCERVYRQADATLSVIDHTLINKDPDGDVRAALTELRGLMQSRRVDFHMAYFFRLDYSLLVSIASVVVTYTIILLQNVPN

>EposGR4

MCTVLRKYFSPLLHRNEDLRLLEVFKPLYYILSIFGLFPYSIDFLYGKKYLNVAFKSASFTLACNFVVPTVICVFFALHMLELDVASRDNAFTEDEMTKTNFIIEMVCQFLVCMTAYICAFKNRSLYINVLNEMSGCWTNLPKNVGSKILGQLQIKAHCVLFGSILLVPLLESPMTYLSSATAWKKILVLVTFVLPELIQFVLIAFYFVLIMMVVALLENIEEHVKILYHSKSCWTSSNIVEDAKPLPASLRQLRDMYARALAVKRQVNAAFQAPLMLLLAQSFHTLISEAHFLFHGLTFQNDFNVLGVFDCCVWIVLQIIKIYILGHSGAILNQQATEIGRTLHNIPADIDEDISLYLEIQHFTTLMKFQAANITVFGYFSLESSLIFNVTASATMYLVILVQFDKST

>EposGR5

MTKIFRYSRWFGVAGSGNIVWKIFGVFILLLLGVIEGVAIWRVIKALAGWAVDIVGHRSVTARLAGTMFYASSIITLILSWKLSSSWKDLAVFWASVDRNMAINVPPDKSLKSRMISVTSVMMACVILEHTMSMMSQIGFDCPASLILERYTLMSHGFLLLRTDYSIWYAIPLLFMSKVATILWNYQDTLIVAVSMGLTSRYYRLNHFVAKFSAAVNKHVPWNSQSNRRNEYTWRKIREAYVKQAMLVRRVDAWLGSLILLSCLVNFYFICLQLFLGITQGLSGSFIKRLYYLVSLAWLCTRVSCVALAAADINVHSKRALRYLHACDAHCYNIEVERLQNQLSKEYIALTGMGFFSLNRTILLKMAGAVITYELVLIQFDDNGSTAHKPYQYNATF

>EposGR6

MRSGITATCVSVIILVMLLGLELTAIWKLIRAFGGWAVVKGSITARLSGAIFYGNALFSHLLSWKFISSWEDLSLHWTQIERAEVVRLPRDNKIKNRMTIVTSFVAACALVEHVLSMMAATGLNCPPTEYFQQYILRSHGFLVHAWEYSIWIAVPIFCLSKIATILWNFQDLIIILISMGLTSRYQRLNSCVEYILKSEKSKGKLERVGTDKYAEVQTWRRLREAYVRQAALVRRVDGSLGALILLSNFNNLYFICLQLFLGINNEQGPLINKIYYFLSLSWLILRACSVVLAAADIHKHSRSALPFLHACPCRVYNVEISRLKTQLTHDFVAIKGMGLFALDRKLLLEVAAVILKYELVLIQFDK

>EposGR7

MFLFVRKYFSPFVHQDEELSFLQIFKPMYYLVSAVGLFPSSIKFPTGKTLTVVYKSTTINLACTLLMATTVCVSFSLHMLELSINGRQELNNFEEDDITLTNYITNLVLMLIFSVIAFISALKNRSLYIKILNEMAECWAVLPNSVGSNGILGPLRVQVNCVVLGSLLLMLIVQLVVTCTADLPKSKIALIAMTFNLPEMLQFTVLAFYFVMIVMPVAILKNIEEHFGMIFDVRRVCDVENDFVGLPLGSSLASLRQLRGVYARALAVKRQVNAAFQAPLLLITALSFHTIVTVAHGIYHVLTYQNNFTTHDVVEECFWVVYQLIKFHTLGYSSALLELQANKIGSTLYKISTHINEQITQYKSSADVNKEIKFNLEVQHFASMMKYQDTKITIYGLFPLKSSLLFTAVASAASFIVILVQFDDGK

>EposGR8

MFSFLRKYFSPFVVQNENLSLLQVFKPLHFCLLVVGLFPSSIEFPSGKRDFTIVFKSSSISLACTLLMTVLISVSFGLHMLELSVSSKENAFTIDQFTLTNYISNLVLMLLFAGMAYICALKNRNIYIKILNEMAGCWADLPNSASDSILGQLRVQVNCLVLGSLLLMLIMQLVVTGTSDVPTSKIVLIAVTFNLPEMVQFTLLAFYFVMIVMIIAIMKNIEENFALILPMRRVNGVDNHYVRLESGSALASLRQLRGVYARALAVKRQVNAAFQAPLLFLLVQAFHSLICDAHGVYHGLTYYRDTFSTHDVVEEFFWVFYQLIKFHILGFSSAILKLQADKIGRALYNISAHVNKEITQYKSWADINDEITLHLEIQHFASLMKFQDILQYMAFFH

>EposGR9

MCIYSYILVTVLVICTIFGLASEINVGVELSVRMTSRMSQVVSTCDVLVVVATAGAGVYGAPMRMRNMLKFMDSVASVDNSIGAQYSIMTERKLIAVLLAILLFFTVLLADDFCFYALQAKKVDRQWDVVTNYIGFYLLWFVVMVLELQFAFTALSVRTRFRAVNDAIALTARHVAVPLEKLEHPTPVNMFAIRVAPAEARRSASNVSLLVDSLPGAQLEHPVIIRKTVNGEPRLIVPPCEAIRRLAALHGALCEVVQRIDCSYGLPLIVILLSTLLHLIVTPYFLIMEIIVSTNRIHFLVLQFLWCVTHMLRMFVVVEPCHYTVMEGKMTEELVCRLMTYAPSGGALPSRLELFSRQLMLRSVTYSPLGMCTLGRPLIASVIGAVTTYLVILIQFQRYDN

>HarmGR1

MNKEEHGFRVYNTNTVHKNETRKREMFQRIDEKDGIKEYDAKDLYGPEITDKDGALLDAHDSFYITTKSLLVLFQIMGVMPIMRVPKNAQTTKRTTFNWISKATLWAYLVWSLECIIVVKVGRERLANFQSSANKRFDEVIYNIIFLSILIPHFLLPIASWRHGPQVAIFKNMWTHYQLKYLKITGTPIVFPNLYSLTWGLCVFSWGLSFAVILSQHYLQDDFELWHSFAYYHIIAMLDGFCSLWYINCNAFGTASRGLAMNLHKALEAEHPALKVAQYRHLWVDLSHMMQQLGRAYSNMYGIYCMVIFFTTTISLYGALSEILEHGLSYKEMGLFVIVGYCMTLLFIICNEAYHASRKVGLEFQVRLLNVNLGAVDRSTQREVEMFLVAISKNPPIMNLDGFTNINRELFTANVSFMSTYLIVLMQFKLTLLRQSARKTLKTIVRAVFNTTTTILDDDFTDDVDEE

>HarmGR2

MTIPDHLFDEGINNTLLQHDMRHVQQNRIVYEKTQREYEQEQRDMLSSQDGDTCEIHDQFYRDHKLLLVLFRALAVMPITRSRPGTITFSWRSTATMYAVCFYIAATAVVMIVGYERIMILRSIRRFDEYIYAILFVIFLVPHFWIPFVGWGVAHQVAIYKTNWGKFQVRYYRVTGENLKFPNLKTTIVMISVGCLLLAVCFLLSLCILMDGFLLRHTTAYYHIITMINMNCALWYINCKGIKIASQSLSECFRRDVEAECSAKLISRYRYLWLNLSELLQSLGNAYARTYSTYCLFMFANITIAVYGALSEIVDHGFGFSFKEMGLFVDAAYCSTLLFIFVDCSHNSTLTVAAGVQETLLSIDVLSVDRPTQKEIDHFIQAIEMNPAVVSLKGYAHVNRELLTSAISMIAIYLIVLLQFKISLPRDPQIVAT

>HarmGR3

MTVPIPNGFPVQINSKPKNKIIFLDVTPVSTPIKPHSPNVVAPMRNNLVAPHISNDIIYENIKPVFTLLRIMGVLPITRPSACVNQFQIASSSMLYAILVFLSLVSYVLYLSLHKVQILRTAEGKFEEAVIEYLFTVYLFPMIAVPLLWYETRKIANVLNGWVDFEMVYKQLSGRTLPVKLYKKALAMAVIIPILSTTTVIVTHVTMVHFKPMQLVPYVFLEILTYMLGGYWYLLCETLSICANILAEDFQNALRHIGPAGKVAEYRALWLRLSKLSRDTGIANCYTFTFVNLYLFLIITLSIYGLLSQISDGFGIKDIGLALTAFCSISLLFFICDEAHYASHNVRTNFQKKLLMVELSWMNTDAQTEVNMFLRATEMNPSQISLGGFFNVNRTLFKSLLATMVTYLVVLLQFQISIPDESQNRDEEEEVPYNITSATTEAMTTSTTTIMTTVLTTLAKKKKKN

>HarmGR4

MEIKLCKLFVVLTEGIEYMKGNSVKNLNEKKRDDFLPTLNNVFLKARFFGISGYGLTISFFWSLILFSMLVVMESVAIWRVVTLLGEWLVSASNNGLIGRLSGAIFYGNALISLFLSSKFVHSWRSLSNYWLRMETSTALDFPADVRIRKRTIYITAFVVSVAVVEHILSMISATGVGFPPEEFLYRYVTLSHGFILKAQDYTIWKAIPIFVLSKLATALWNFQDLIIILISMGLSSRYNRLNLYVRHVVSVEKQFESKQRFGTELYLQIQVWRRLREAYVRQSTLVRMVDRNLGSLVLLSNINNLYFICLQIYLGIHKSSGSTISRCYFLFSLGWLIFRACSVVLAASDVHLHSQRALKSLHACPSAAYNVEIKRLQYQLAHDFVALTGMGFFSLRRELLLEVAAAILKYELVLIQYDK

>HarmGR5

MQNGWNNVISNISVGSVNTVNYLFRTWERLAPNRNMDLYSLEKFKKYKNDWNYPVHVRYQDQVMAEKEKPRMTFQTAMKVTLTIGQCFGLNPVQGIREKDASKLRFKLLSGRCLFTFFSLIGQFIMAFVLFLSLFKETSSTVDTASNFGFTTTILFFRIATNWPKLCMHIAKVESVDPNTDNKLGKKFNIACISILFLALMEHLFSELHGISIALDCFPDTPVYESFMKLSFQWLFGFIPYSDFAGGMAHFSNLQCTFNWNFADVFVICMSMYLTARLEQVNQRIIAAKDKNSPSSFWRTMREDYNRSVHLVRQVDKIIGGVVFMSFASNLFFVCSQLLHTLAGGIKASQRCKPEIGADRRFFYGYEHSIYFVFSFSFLVIRSLAVSLTASKVHAASLEPAYSLYDVSSANYCVEVERFLDQIHGDTVALSGLQFFHVKRGLVLTIAGTIVTYELVLMQFTGITPTTSPESVSGVIK

>HarmGR6

MGQTSFRRNMSFWIPVKKNKVDVAKPKVKNITSFQDALRVTVIIGQVFSLLPFVGVFTNVASNVKFVKTSWKCVYSLLSLVGQMFMAVLCINKLAKTTVSLNGTSPVIFYVTTCVTMMLFFQVARRWPALVQHISKAEDMDPNFDCSLTRKCNITCAVVLILALCEHILSLLSAFAGASACYSGMDTYEGFVTHFYPWVFSYLPYSIVLGVITQFLHFQSTFIWNFSDLFVICMSYYLTSRLEQVNRKLLAAQGKYLPEIFWRATREDYCRATQIVRKVDEVISGVVFISFANNLFFICLQLFNTLEDGLKGTGECTPKLKKIVVSKSGPLGGHEAAAYFLFSLVYLLSRSVAVSLIASQVNSASSVPAPVLYDVPSPVYCVEVQRFLDQVNGDKVALSGLQFFSVTRGLLLTVAGTIVTYELVMFQFNSSTPTLNITSPTVVTHTITTLAT

>HarmGR7

MSSRGFGQFLRDGNLILPEQPNHDDFLTVMEKVFKWSCLIGVLGSKRHINYAWSGFILLVLLFMESQAIWKVIKALAGWAIDTAGQRSVTARLAGTIFYTIAILSLVLSSRLYRSWGQLSALWARVERIMAVKAPPDKTLKRRMYFFLGFMTVCSLLEHIMSVVSAIGLDCPPALIIKRYVLISHGFMILRHEYSDWYALPLIFMSTLASLLWNFQDVLIVLISMGLTSRYSRLNQCLAKICALERKQMDSDKKNEATKVYAWRKLREAYVKQAMLVRKVDDAIGSIIILSCFCNFYFICLQLFLGITQSKASEPIKTAYYFLSLGWICFRVLCVVLAASDINVHSRLGLKYIYTHDSHSYNIEMGRLQDQLSKDYVALSGKGFFYLSKSILLQMAGAIITYELMLIQFDDQGTDDVQLNLTKNAIGV

>HarmGR8

MSSKEFKQFLRQNKLLLPQQPFHDDFLDVIEKVFHWSCFYGVFGSKRSISLIWSTLILGSLVIIEVLAIWKVIRALAGVARDMSGHRSVTARLAGTIFYSISILSLVLISKLYYNWRTNIAGLWGKVERSVGVKIPVDKTLKCRMSFVAGLMTFCSFFEHALSILASVGFDCPPSLILKRYVLVSHGFIFMGQDYSEWFAMPLVIISTIATLLWNFQDQLIVLISMGLTSRYRRLNECLAKFCELEKQHMDSDKKVEAVKVYTWRKIREAYVKQAMLVRKIDVALGGIIILSCSCNFYFICLQMFLGITQGMSTDFLTGVYYMVSLAWLCIRVLSVVLAASGVNTHSKLALNHLYTYETHCYNVEVERLQDQLTKDYIALSGMGFFYLNKTILLQMAGAIITYELVLIQFDDQGNDGIALNATNI

>HarmGR9

MGVESAKVEEVTAAPVPSESGARPSRPTHCVVGGAHAFILRISSFFGLAPLRFESRSNGFTVSISGAMCVYSYILVTVLVICTIFGLVAEINVGVELSVRMSSRMSQVVSTCDVLVVVATAGAGVYGAPRRMRNMLKFMENIASVDTSIGGQYSLVTERKLCGIILAILIFFSILIADDFTFYALQAKKLDREWDVVTNYLGFYLLWFVVLILELQFAFTALSVRARFSAVNDALALTARQVSIPVEKPKSSSPLNIYAIRVAPVDSQRSANVSLLVDTMTGREHVVIIKRTASGEPRLVVSPCDAVRRLAALHGTLCDVVNSIDDSYGLPLVVILISTLLHLIVTPYFLIMEIIVSTNRIHFLVLQFLWCVTHMLRMIVVVEPGHYTIAEGKRTEGLVCRLMTSAPSTGVLPSRLEIFSRQLMLQSVSYAPMGMCTLHRPLIASVIGAVTTYLVILIQFQRYDN

>HarmGR10

MEYGLDAKISKELESINFRVLQETQLDDEAKKRSTKPWIEEDNKLVGKRRIDVKDQFTAFQKAMKVLLVWGQTIGLNPVTGILQKDPSKMRFTVYTWKFLFSLTVAVAQTIGTTLCIYKLFREPTSISALGFVTFFTSTCFTTFLFILIASKWPTLMQDIVRSKLDEYVDKKIITKCRITCCIFIGMALMEHFLSILSRVARVIECSQNETDHGEVFVKVTSPWLYDLNVPYVVAVAVMVQYVNLITTANWNYSYIFIVCVSMYLSSILNQINKRIALEAQKTHVPAKIWINLREDYTRATHLVKRFDDVISGIVLVTYANDLFFICLQLYNVLSNMSKAAQLVNKLCPDQDGTFRAYSYPAYLIYSVLYLLVRFLTVSIVASGVNTASLLPAPILYGIPTTAYTKEVERFQNQVNGDVVALSGLHFFYITRDLVLTLQN

>HarmGR11

MPSKLFLKTFNMATRHRLDKREICGLHSTVRGTLFCSRVMGLLPVSGLTCPTSRRLRFTFRSPYTVLYVASLFGQLLMFVMTLCWLMMNGISLANITNAVFYTSSLISSLILLHIGRCWPALVGSVETLERELPPFHRNVASISNVTTIFILTAAIVEHLLSVFYGLKVACACDSNNVAENYFRFNMPWIFDYTPFTIWKGALSELFNIQSTFVWSLNDLLIMVISIYLTEHLLIHNELLKKAAEQEHFSCLEFRTQYLKIVRLVKLINGQFGIYILTSFGSNLYWICTQLFYSLSRTQTGHFITCTFKDSPTKVPPANEYENPLCPWMLGEKGALNGVEHSIYFTYSFSFLLLRTLLVLLLAARIHSNSVAPLYVLYGIPSSRFHIEVERFIAQINNLKVAMSGLDFFYVTRTMILTLLGTIVTYELVLLQFNR

>HarmGR12

MKVHQLTMRRSNKRCGLHVCLRHAMRLARWTGFFPLQGLGQAYADGTRYKILSLYFIYNFTTLLGQLVMSCFAILLFFQTEVTLNSISNVIFYVTSLISAVLFLKLAKQWPRLMARATETEQGLTELKLPNKVIVKCCVIAYVAMALALVEHILCTSFNLTFVMHCLKEAGITTNVMENYVVHRMPYVFNYVPYSLFWAFLFEYLCLQSTFVWSFNDVLITCFSIYITAYFRSLNQVVTANSKKDKDNMIPWSTLRVHYSKLVRLVKEIDNHISSFILLAFFTDLFYICLQLFNSLHRNYASFKFCNELQTKQALTSPSYLLYYLYSFIFLVLRATMLSLFASNVHCAALEPVHAVYDVPSTLYDNEVRRFQLQLHHTKVGLTGKFFYVTRNMVLKVIGTIITYEIVLLQYTITPNPYYNGTKVILNISSHSYS

>HarmGR13

MSAFRDTECVVTGTLSMMLRMSQIAGVAPLSFRRTHGGWYIRTSRAANCYGKALSFCLWFLSSFTIAIDILIQPERSFRTRTNSTRIVWLADVATVAIVVCAAAFTGISRMRCLTVYALKLEEINLRLSLFHEEPSNEANRRLIAVSSMIFVVSTILVDYSIFIYQVITEHGKIVTSCMYIFYNISTIVQQVILVTFSETVTSVLTSLQMLNNCLKNLLQEILDSSELTNCALNYDSYINMNPNRSAIPNKSINSVVDTMAVYKGYRKNSIKAVPSTIRRLALLYCSICDVIRLVNDSHGLILVALMLCLLLHLVITPYHVITNIFNKERSDRSSPLLQLNWAVLHFVNLLLIVEPCHRTHEEMEQTRHLISQMIRYTPSEHGVLLTELQMFYQHLILNEVSYAPLKMFSLNRSLIVTVI

>HarmGR107

MNADLLKCFAPIHNVLLFLGSSRLKIKNNMIAPSTRYQKMYALCCIFIVTLSFSYIQLYYYLTYYHEDTTIYVCYAIGIMVQNVSYLSHTIFARFLDVESSVKLCQNLQKVDNILRLKQFKRYNEQQYYWNVVVLVFIITSFECGFLVHIWYTVEYPILAFFAGIGLLNVYMELVLAASLIVYLAIRLKFLNKIAHHNFKIKGYYNNTRAACAVDEHLLINSDVKDANIDLGNFLICMKEILKMYQHITQVFSFPVSRCTTRAKIIKVRASWRALSSRLLSF

>HarmGR108

MPRNIEELSEDLFTDDFIRIFQPILFVLRALGLARVSIKYRYPTGTSKWYLLYSNVFWLLNALSAVYFFFHCGESFDSKYADSTLKFGVLNSGINGILVVFRNNLERNNKFGAMYVKLQKIERHLNMEDTKSINKQLRSQSTIVMVVAFFVTIFWIVLFKYLFMKSMCIPLIVNVVTSIGLQTEMAQIYFIIKFIITRVNYINDMLRQVSLLSIEPLTKPMDDGILFVVSNTLKHQDGEVPGELVSGMHCIFETLSDFTGLFQFSLFYFICQILAWNLVTIHYLVTSMKEQGAADTDLLLCVMPVLVALQFIILTLCLKAQDLSTKLEEARKLCIDIISSPLINGKSREHAKQLTLLVEGRRSVSIYNICTFGTRLPLHLLAITASYTVVLLQLALL

>HarmGR109

MKNLKENIKIEYLSKDILDEEFMKSFSLLYYTQRLIGSTRVQIKHRFVTTPSFLQKFHTLISVVLLLGLDYLVIQKYDKILFDRETIYYLSICVTGLQTITFLCNIINVRFINGDANVELLVNLQQIDRRMNINRNKSITTLLVKTNVISLVVVLIMFITLLGVASAKGTAAFWPYTGIAYSQFSFVIELISCSNMFMYFYVRARFINSIIKNYIDQKGTQEILYSKERFLSSYFASKVFMRRLAAGSHNFVSSDTDVYLKQLLEGFFKFQDIYKFQVFMFCCKLVASALLTFEFLLYAVQNDTVGLWDSLTPSFFTVIDLVMAILLGVRCEVFIREVKETKRLVITVMSRHYDGRLREKSKRMLKLVEETPPHFSVYDMWQLDANVLLQMFMLVTGLIVTQMQFAFL

>HarmGR64a

MKGNSVKNLNEKKRDDFLPTLNNVFLKARFFGISGYGLTISFFWSLILFSMLVVMESVAIWRVVTLLGEW

LVSASNNGLIGRLSGAIFYGNALISLFLSSKFVHSWRSLSNYWLRMETSTALDFPADVRIRKRTIYITAF

VVSVAVVEHILSMISATGVGFPPEEFLYRYVTLSHGFILKAQDYTIWKAIPIFVLSKLATALWNFQDLII

ILISMGLSSRYNRLNLYVRHVVSVEKQFESKQRFGTELYLQIQVWRRLREAYVRQSTLVRMVDRNLGSLV

LLSNINNLYFICLQIYLGIHKSSGSTISRCYFLFSLGWLIFRACSVVLAASDVHLHSQRALKSLHACPSA

AYNVEIKRLQYQLAHDFVALTGMGFFSLRRELLLEVAAAILKYELVLIQYDK

>BmorGR1

MNRHDHRFSIYNPKRNEAMWKRELFVNNEGKDIKDFQIKDIYGPEITDKDGALLDKHDSFYLNTKSLLVLFQIMGVMPIMRVPKSAQTTRRTTYNWISKATLWAYLVWGLECIIVVKVGQERLANFQIGSNKRFDEVIYNIIFLSILIPHFLLPIASWRHGPQVAIFKNMWTHYQLKYLKITGKPIVFPNLYILTWGLCIFSWVLSFAVVLSQHYLQDDFELWHSFAYYHIIAMLDGFCSLWYINCNAFGTASRGLAINLHKALEAEHPALKLAQYRHLWVDLSHMMQQLGRAYSNMYGIYCMVIFFTTTISLYGALSEILEHGLSYKEMGLFVIVAYCMTLLFIICNEAYHASRKVGHEFQDRLLNVNLGAIDRSTQREVEMFLVAIAKNPPIMNLDGFTNINRELFTANISFMSTYLIVLMQFKLTLLRQGARKTVTAIVRAIFNTTITDNGAGGSDEDQE

>BmorGR2

MIPDHLFEEGINNTFLDYDMRHVQRNRNIQEKTQKDYEQEQRDLLSSQDGDTCEIHDQFYRDHKLLLVLFRALAVMPITRSRPGTITFSWKSTATIYAVCFYIAATAVVLIVGYERIQILQSIKRFDDYIYAILFIVFLVPHFWIPFVGWGVAHQVAIYKTNWGKFQVRYYRVTGENLKFPNLKTLIVIISVGCLLLAVCFLLSLCALLDGFLLKHTSAYYHIITMINMNCALWYINCKAIKIASQSLSECFQRVAAGVQDTLLSIDVLAVDRPTQKEIDHFIQAIEMNPAFVSLKGYAHVNRELLTSVRFTTIIEADLLMIY

>BmorGR3

MSFEIKNNFFRTSVPIPNGFPVQTEAKSKNKPIFLDVSPAPTPKVNSPNAIIPMKNNLIDPFINKDIIYENIKPVFMVLRIMGVLPLTRTTSGVTYKQLSNRILPVKLYKKSLLIAIIIPILSTTSVIVTHVTMVHFKTSQIIPYVFLEILTYMLGGYWYLLCEILSLCANVLADDFQQALRHVGPAGKVAKYRALWLRLSKLARNTGVANCYTFTFVNLYLFLIITLSIYGLLSKISEGFGTKDIGLALTALCSVFLLFFICDEAHYASHNVRTNFQKKLLMVELSWMNTDAQTEVNMFLRATEMNPSQISLGGFFDVNRTLFKSLLATMVTYLVVLLQFQISIPDATQPEIPTNIDDHVQNITDTTTEASSPISTLMSAFAKRKND

>BmorGR4

MSRIFSMTRYFGVSTCKPSIAFGWTVILLLMLLAIEVGAIWKIVRLLGGWAVHSTDSRGFTARLSGCIFYGNALLSLILSIKFVSSWEQLSERWSRTETDPGLRLPSDSRIKRRTVLVSAFVMTCACVEHMLSMMSATGFDCPPEEYTERYILSSHGFLVQNDEYNLWLAIPIFIMSKLATALWNFQDLIIILISMGFTSRYNRLNTYVHRVVMLERNLKEGAQVSSENYMRFQIWRRIRQAYVRQAALVRLVDDQLGALVLLSNVNNLYFICLQLFLGINSKDRGSFINRLYYFISLGWLMFRACGVVLAAADVYIHSKKALISLYLCPELAYNLEIKRLKYQLKNDEVALTGMGLFSLNRELLLEVAGTIVTYELVLLQFSNED

>BmorGR5

MYACYKIIVALSLNRQYNHTVTRVGNRKHIKSTTRGNFRERILRKVQNRISPEPIQEDSKIPLTCQFQLFQTAMKHLLISGQFMGLNPVSRISDHSPTKIRFTVLSWKFVYGVTIGIAQACATVLCFCKLLKDSVNIVALDFVKIVSAYFAFYLSTGCNTFIFLRVASKWPTLIKHVYETQLDSYIDVKVKNKCFAAYIIFFSMSMTEHMLSLLSKFVITMDCLPKGSDLFESYIIRNFPWLFEFDVPYYLPIGVILQFLTLVSTINWSYSDLFIVCMSIYLTSILKQINKKIEMAGNSNHLPIPFWRTLREDYTRATRLVRSFDDTISSVIFLSFASNLFFICLQLYNILSNGVTSKYNLLKEMCPNYPSGPLGGYEQIMYLLFSLSFLLGRSLVVSLVAAKVHSASMVPASALYNIPRNMYCSEIQRFLDQVHGDKVALSGLRFFYVTRSLVLSVAGTIVTYELVLLQFSNED

>BmorGR6

MLLRNYKQNLSFWTSAKKSKIHKIQSQETVTFQGSLKLVLFIGQLFSLFPVCGLLSNDANKVKFVPISWKCGYSMLSMIGQLFIIVMCILYVAHFETTLNGTTPIIFYGVTFISMIAFIRASRRWPELIQHISKSEELDPSFDFRLKKKCNITLLLVLVLAILEHIFSIRSAYSASQICYPHTGFYEGFVRYLYPWVFDFLPYSAALGMVTQFLNIQSHFIWNFTDLFVICMSYYLTSRLDLVNKKLLPAQGKYLPEIFWRTTRETYCRATKLVRKVDEIINGILFISFANNLFFVCVQLFNTFDDSVDMVGLCYNYSERRTKPVGREPVIYLLFSLGFLISRSITVSLIASQVNLASTVPAPILYDVPSAVYCVEVQRFLEQVNGDNVALTGLQFFSVTRGLLLSVAGTIVTYELVMVQFNQAPASDSFTEKLVENNISTIETFYNYS

>BmorGR7

MVLEAHTQIQYCTAKANYCEFHAGLRHLMRLARWAGFFPVQGLSQTNPDDVRFEFRSLYALYHAITVIGQTVMTFLAFYSFVDSNVSLSVVSNFLFYFTNYVTLVLLWRLSKNWSALISKTLEFEQSVTEIRTTRNLVSRTNTLTYVVLIFAMIEHALSKVFNIRSVMCCLGETSLNHTVINNYFKFKWKFVFDYFSTSTTYSYFVGFIAEFLCMQATFLWSFTDVLIMCFSIYLSSFFEDFNSTVSSFMKKASKTVPWSTLRVQYSQIVLIVKQMDEQLDYFVLISYFTNLFFICFQLYNSLNRIYDANDVCNENMDIIATASVTYLTYYVFSFLFLVTRALLLSIMAANVHSCAQVPQLALYEVPTADYSLDVQRFQLQLRYTTVGLSGVCFNVTRGMILRVIGTIVTYELVLIQLTKKNLDNDTSIRDYYLPKHLI

>BmorGR8

MAPRSVRSMVGTSKKDMLKGGFYETVRIPLYIYRLIGILPISGLWHRSSKYNRFSLKSFYTIIYAPTIVMQTFLLLVHIYDLFAFFFGHQRLGRLIYHMNFYTITILIFMGSRKWKNVIKEIETIELTLPRLRNSKKALALTKSFVFAFFVFSLAEVVLILQFTLRLTKQRHVLPGDSGLYLRSYFVYIFPYLYDHFPFSYVMGFIVQIIKVQGIITLNMVNCSVVILSIYLTNRLKHYNRIVFAKGSKTNNTRLKWVELNLLYTRISNLVKIIDKNLNPFVFISFTANLSYICAQLFYILNKLTSSRTVKITSFLEDKRSDWETVLYISISFALVVLKVLLVSITAAEVHTTSREPLRLLYTLPTAEYTIETQRLMTQVYYSNLSLSGLNFFHITRGMLLGMVATLLTYEIVLLQI

>BmorGR9

MPPSPDLRADEPKTPCLVGGAHAFILKISSFCGLAPLRFEPRSQEYAVTISKGKCFYSYILVTFLVICTIYGLVAEIGVGVEKSVRMSSRMSQVVSACDILVVAVTAGVGVYGAPARMRTMLSYMENIVAVDRELGRHHSAATERKLCALLLLILLSFTILLVDDFCFYAMQAGKTGRQWEIVTNYAGFYFLWYIVMVLELQFAFTALSLRARLKLFNEALNVTASQVCKPVKKPKNSQLSVYATSVRPVSCKRENVIVETIRVRDKDDAFVMMKTADGVPCLQVPPCEAVGRLSRMRCTLCEVTRHIADGYGLPLVIILMSTLLHLIVTPYFLIMEIIVSTHRLHFLVLQFLWCTTHLIRMLVVVEPCHYTIREGKRTEDILCRLMTLAPHGGVLSSRLEVLSRLLMLQNISYSPLGMCTLDRPLMVTVLGAVTTYLVILIQFQRYDS

>BmorGR10

MTMSIKPRLQCMVPPSLALALRVSRLAGIAPLKFVAKQSNIMIRLSTSLCVYSYLLVTALNVCTLIAVMIDFSVPVKLSIRMQTETKRFVWIADVVIMGILSGVGVYTAPIQMRRLIAYLHRIHKINSDLGTYSSSLTDKMLHRLTIGMLLITSVIIVTDFTFVMYLADLNHRQLLIAIMYWCYYCSYFIAHLLEMQFVLIAALALSSLKLVNNGLRTLLHQSGIESLTEIPNSNEQHTANAILPQPPKKSVNNSIDTLAFVVTKRSVRFPTAGWMDQRTIRRLALSYGSICEVVRQIDNNNGIIVLLLLASFLLHLVVTPYYLIISFVTESPHTGFEKVLNPILQTVWCLYHTFGLVMIIEPCHRTHEEMETTRELVSRVMCSADPRDPISIELEMFFRQLVLNKASYAPLKVCTLTRSLVATILGSITTYLIVIVQLEIKNMQ

>BmorGR11

MKPFRFFLFVENVICVYRNYSFHKRYARAIILSRVMFEVSLIILTLHSCRNFGAVKYKTEIIFTYLATASSTILILLALYKTNRFTELFLNFKAFYRNRNLDVDHLEKWNRKQKMATVIIVLFCVIKFSTLIYTDLIGEYSTPCRGYFTEYLFYTNLFMCNARYLFEFSTACVVLHLVSEQLDYIAISMDCTMFLYIDISKKNIMSSAKKRKLKYFDIFKQFEKWTDAYMNVKRSANLCDTVFRAQLAIMITTITLYYIILLYGITSFNIERGKFSVVKSLSYLISLFGFLIALLLLSKAGQRIQKSAENLRRKLSKFLLHSLEDPEFHRAATNLLRLVCTHHIKMRCFGFIDIDMTLLPSCLMFVTSYTVIALQFNNVV

>BmorGR12

MKNLKLCRTTFYFKIIMCSRFISGLYFKATSKKWISYLYKVICVLYIICITRLFYAKEDTFKPLVFXXQFIGNSIESLRTGEGHVLKCYSTIFSLKLIRNYLPDSNNHIPISSITNFLVIIWKVFDQVYIVLMHYFYTTDIIIHLRILSILTTIGVNLSLMPIIVIFELMWRAVKALRKSLGEHLKGPVLIEGRERLKAQQILRCLNVYKDLNATLKFNSTPMKTMILISTLATFIRLTLFLYQAILGHNEGLHLPRKILAIIYYALPVCLLGVLMELVARECDKLKTLMTKELLVCKDDSYCTVIVDAVSYIELNPLKFSILRAFNVNSTLILGLTNLCTTYLIAVIQFTYSCEDINGLSHSHSH

>BmorGR13

MEDSFNRLLSIRNMIIFQNVCGFYHMCTEKLYISRIIKMYCVALAIVLSVFCFQNPDITYLSWDVVWVTFGYTLNVIICLRYNGNYFFQYWNGLHEIDIKMNLTSIDKEKVPISRAVFTVFLILRSTAFAMTIFVFGYLETGILSNTIISIYSINLTEFYRNMSNIPMILMFETFYVRIKILKEQLCSELSTVLGCNNDARQLKLILKYLRNYRSLVRHLMDTTLPFKILILVILVGSFLRSLLIGYAFVYNSDQIILLSLPVMFSTKILSEVVEIKLICTKELLKNKNEGLVLLDLDSKKPTFLTSKACGEQLQDALSFLNNRSYSYTLLQVIEFDCSLAFVFTSFCITHLIVVVQFTHVLD

>BmorGR14

MNLHKNIIPIRNNLFANKVTAIALPKTLSVLFKLIHIFFLLDLGVYEYKTFKIKCIVKFLTISGSLTISVVCFSFMVSNLSEHTFVGWYGFFISTYIFVVLFFNLSNRMTFVEFYKTLLRFDANYGIDSNEYKFNFKIIFVNILFIANRMVLSFVYCSYYPQNCIRPRYAQILFMLPWLTLDVLLTTNMFLFYATYCRIAKFPMLIKNSMNIVALRNSYKLIVDSLEKTQTSFDIVFIIALVFSVPEIMMSIYSTLLEVISKHFLEVASILSLNYVAIAQSLLLTLAPSLCAGVLPWKTNNIKIILHEKLFTEKDKASAREIELFIKYIESRPLKLRACNLVPLDFSLTIIVLNICVTYLIVIIQFTHLY

>BmorGR15

MISSSDINHKRNKVFAYNVPGIALSKTLTVLFKLLHYVLLLDVGIYEYKTFKNKCIVKFLTIATGVSVSIVYFCLIATVLRKNAFFYWFYVLFISQYMIIVFIFTLSNGMSFTDYYKMLLRFDAKYQINSNNYYFNIKIILVIIISILNRIGMAIIYCSYYTKNCYEMSFSQIIFVLPWLTRDVILIMNVFLFYVTYCRITKFPALLENTKNVGSLRNSYKLIVDSLEKTQKPFDFVFTISLVFNIPEIMLSIYFTLLQVIHSHFLEVAPTLSISYFSITHSVVLILAPSLCAGVLPWKTNTIKIVLHDKLFLEKDKNSARNIKLFIKYIEARPLKLRACNLVPLDFSLPVIVLNLCVTYLIVIVQFSHLS

>BmorGR16

MIMNLTTDRISKRNKVFAYNVPEVTLPTTLKVLFKLIQFTLSLDFGVYKYKTFKMKCVAKVLTLAGCLAASAACVSLIISNIFENQLFFGWYTLFVCQYTIVIFMFTFSNGMTFIDYKMMLLRFDAKYQIDSNVYHFNIKIVLVVVISVTSRLFLCAVYCIYSTENCIKPWYNQLLFFPWLSLDIVLIMNMFLFYATYCRLAKFPSLFENPKNVVPLRNSYKLIVDSLEKTKKSFDAVLIAALIFNIPEIMMSIYYTLFQVMNKHFQEVAPVLSLSYFTIILSVLLILAPSLCAGVLPWKTRHMRLILLEKLFAEKDKNSAREIELFIKYIEARPLQLRACNLVPLDFNLPVIVLNLCITYLIVIIQFTHLF

>BmorGR17

MGFSLGTTALSMFFFEKPVVFTIIQITMIIVKPAKYKLSDPFRPKDTSKLSESIIMYFKLFHIFLGIDLGGFRYQNRQVKYAVRLISLIQPLAIYGLCIYALLKIIANTEFLWYTISFTEYVAMSVAITLFSNEMTYCNFMINLKFIDTKLKIGDESFRIGVKLISSTILIGVTRCFTTTTYCLLGFCAKPTAAQILFQIPWLTIDLMLLQYMFIFYACYCRLVKILRILKKRNTDIEEMRRIYKTLVDVLDRARAPFDLAYLLGLLFSIPDVLYSIYESIIKVGEINTAKALSMSIIYITNIQSLALMFAPALTAGFLPSLTMKMRIILHDKLLEEQDKKTYRHIVLFIKYIETCPLKLKACQIIPLDFSFPIIILNIVVTYLIVAIQLTHFL

>BmorGR18

MRRSTKVISMVNQSDKGEIKTCSRFMKIYFFVIYILTGFNFGFYTGRGLNFLRVIQASVLLLRFIIASNCIYIAFHFRLLEAIWYSLTFSESLAIVVCFMLSRSALSCKNLFEYLYSVDQELKKSVGPSIEVKLALYTVVVSVLRLTVYVFCAIAYYETLHEGFCVELVYNTPCYCSDLYLVIHFTIFHSVYCRLKALRISMNEKFDVYKGTLIYKSLIDNLEEIKKSLDVPFFVILLNAVAIAMINILVTLEISYGQTMKFIRTAPRYLETVLLFSSAFAPVLAADMMASEAQKIKVTLNNILQRDDSLLEDDRRKVKQFAGYVSARPFRLRACRVLSLDCTLPVTVLSICVTYLIVVVQFTHLY

>BmorGR19

MRRSTKVISLVNQSDKGEIKTCSRFMKIYFFVIYILTGFNFGFYTGCGLNFLRVIQASVLLLRLSVASYSMYIARYSPLLEVIWCCLTASENLAVVVCFMLSRSALSCKNLFEYLYSVDQELKKSVGPSIEVKLALYTVVVSVLRLIIYVFCATAYYRKLFDGLRLELLYHTPCYSLDLYLVVHFTIFHSVYCRLKALRISLNEKFDVYKGTLIYKSLIDNLEEIKKSLDVPLFVILLNAVAIAMINILVTLHISYGKTGCRIDYKSVAEMACSQLVIRTSRSETANGSSEAKRKKLVVGPLMKLITAAPRYLETVLLFSAAFAPVLAADMMASEAQKIKVTLNNILQRDDSLLEDDRRKVKQFAGYVSARPFRLRACRVLSLDCTLPVTVLSICVTYLIVVVQFMHLY

>BmorGR20

MRRSTKVISLVKQSDKGEIKTCSRFMKIYFFVIYILTGFNFGFYTGCGLNFLRVIQASVLLLRSIIASYSIYVAIHFRVLEAIWYCLTFSESLMVVVCFMLSRSALSCKSLFEYLYSVDQELKKSVGPSIEVKLVLYTVVVSVLRLTVYVFCAIAYYESLHEGFSVELIYNTPCYCSDLYLVVHFTIFHSVYCRLKALRISMNEKFDVYKGTLIYKSLIDNLEEIKKSLDVPMKFIRTASRYLETVLLFSSAFAPVLAADMMASEAQKIKVTLNNILQTDDSLRKSYGATVFSFALLVTMIVEATMVGVEDNNIIGPYIDVEREARLTGLYVMAIILTLMFLAKFIFDLVFVYGVVMERAGIVKAYFIMWAVFFFLSVSVFFLNCLDFNTSTIVLEVFYIGLNIYAILLSHSFYKQLNTREDV

>BmorGR21

MAQRTNSINLFRSRPPDIRAGVGEPRIFSKFICGTMFTQKSLVNFDLGKTPRGGDQEHSKFFKIYFLAVHSVTVLDFGFDRNAKKLTKILISMFSISVRMGLAAVSFMSLWGRPNALALGWAPGTLLCENILVAVTYSASRSTFKCGDLFADLSTIDELFGSACDYRIESKMLLFTATMTVLRVVIYSTSRLVRADGFDFVDVLEVLNNLETMCMYLFLTVYFFVLFSIYCRFKKLRELMKNDFEIRRANLIYIALKDCTDKIKQSLDVPFLVVLVFTVLVVMVDVFITLEMIISNKYNMAVYVVRYLEITLDFLMLFAPVLLADMMAVQVDGLKITLHDRLCLNNGVGHDDLSDAMWMSFVDMKRYSSLAEFIGYVEARGCRLRACRVVPLDLTLPVTVFNVCVTYLIVMIQFADLY

>BmorGR22

MDFGFSLGVYKRMKVLENISLVLRVMVAIMCAAMVMKQDILDSAWADITLTESLLVIVSFKLSKPKLSYRELLENLSIVDETQGAPPAGYKVERKLITYIAGVTALRLTVLCLYCVAHTEQYSIDNFIEFLYNVPCYCLDLYLIVHFIIFHSIYCRLRTLRKALSNNFDVYRAHLIYKTLIDCTEEIKKCLDIPVSRSDRHRHSLHKPMGTFLKSYRTPRVMFQLVVILIATILVVMVNVLVTLRMLFKGEVTQRGSVHVQVVLPENLPLSYFNIVHCISLQSIISAFLLRYIEVILSLALLKLLDELKPDAFKSHWKKIWRVLIVGNDGQFARNQTSTDNIVQAANQWNMTRWIRADRTSDIREVAGSQDHLTETDLEEMLTTRSCVTAYWIFDAGSEHVWYVAAAEERREVRQFAHYVGTRPFRLRACHVLALDSSLPITVVSVCVTYLIVIVQFTHLY

>BmorGR23

MAQFQIPSMAGSGLNVAPFSRGRRHEGPKHSNFTKKYFLLVHLVTCLDFGFHRDNDTKTYKWFHAANIGVRLVLSAYVCSVSLSQDLSFASAAWTILNNSKHLLVVAIFTIFKPKSSCAEILKDLLMIDEALKIHRGCDVKGQITVCIVLVTAARLLIAAASSLSLHEAFSASVGAAEVLYSFQSYCLDFYILANFFIFYSVYCRLKNLRRVLQNNFNIYRGNMIYKVLVEHMDDIKKFLDIPFVTSLLVTVIMAMINVLKTLQLIHDGENDVLTIVLRYLEMFLSFSLIFAPVILSDLMSIEADNINVVLHNYIYETDAADIAVAVHPSARPSPVSATEERTAAAAAASVSLAIFSSRLTSFNLCWRARGAGAETFVAVCVVRLGAAVPTPRVSGALAGLHAPRDRAQHLRHLPHRGRAVHASLLISLSLL

>BmorGR24

MCINKKIQSIIKSLVSIRTIMLVQSILGFYHKMSNNFFVSFLFLTYTTILISVLSFYSVNDVMAHKFAYTLSMILEYDINTILSLITAGRQYFNFFEEMKKIDFSIGFGELNIEDLPLSRTLFVTIFVTNILLSIMTAALILFFSTPFLIISSGSTYAMAIVFFGLSLNVLPRIIIFELIYKRIKYINLSLKRKLKALALECDHTIARFEIINENLIIYNKLLQSLGNVNVSLKSSILLTTFTCFFRCSLICYYVITMNEDKVYIMQIIELTKQTLFLGVLIILAEYIKNEIENLKMTVSLQLFTCTDTQLYHQVSDXFEIHRITPIQLCGFKNMSVDTNLFLGLINVCSTYLIIITQFLNAYVN

>BmorGR25

MFVKCLKYVKKFKPMFSVMFIMNFRLICGLYYRIHSDAFVCFVFKVYCILCSMFLFFTSSDLAAPFSRSIPILATLFEYVANVLDCILTGQSYFFHLRMELMRIDPRLRGLDRPPASSIVFTAILSYKIFILAVYIHGKARTTYLQYEWFSSIGIHLLVLFSNLVHMNRMLIFEMVTFSLEAQKKTLGELLKSSLRRERVERKCEILNRFLKTYKRIIELFNNTMAATKLMTLISVVSCFIRILTYLYQVLTTQLSSSSAGSIFTTRHLTFIVTFVREHVLSVYFLKKVGICQRLQINFKIKCNVKTETLELISQDDEYTEKLEDALDFINSCSSKITILRAMTVDATLPLTFISLCTTYIIVVIQFSHIYD

>BmorGR64a

MGLPVGDEDRDESGVEDWEEEGEEDVASLSSPSDLEEGPSRGRQHDRKPQPTTVHHINDEYAMNIDRDSLVLPIGMPDFKLRCETVYLDGLKILMYKREFLPTMSRIFSMTRYFGVSTCKPSIAFGWTVILLLMLLAIEVGAIWKIVRLLGGWAVHSTDSRGFTARLSGCIFYGNALLSLILSIKFVSSWEQLSERWSRTETDPGLRLPSDSRIKRRTVLVSAFVMTCACVEHMLSMMSATGFDCPPEEYTERYILSSHGFLVQNDEYNLWLAIPIFIMSKLATALWNFQDLIIILISMGFTSRYNRLNTYVHRVVMLERNLKEGAQVSSENYMRFQIWRRIRQAYVRQAALVRLVDDQLGALVLLSNVNNLYFICLQLFLGINSKDRGSFINRLYYFISLGWLMFRACGVVLAAADVYIHSKKALISLYLCPELAYNLEIKRLKYQLKNDEVALTGMGLFSLNRELLLEVAGTIVTYELVLLQFSNED

>BmorGR67

MRERKKKFNKLLNTRNYNNIVEALLPSDSIRKISGVSVVYLAVNSENRIVTKFSFIGTIFFLFWYILYFYCTYKAHSEDQTILRTIYNTKLKRYGDDFERIASIIYVTYSMWKVPFRMSGNQVFIQRIVDIDSAIENMGEAVDYNKNAKTALVISIAQLGDFLVRMFCIWLSLENLSVIVPTEKLYQVVYTDALSFVITSHYCFSLIVLRGRYKYINKVLSEIKTRSAWEYKVFVRNKVAPDLEKVQRLQDRIVCEKIKACARIYSMLYKATEAINRMYGTALVLTMLLYLVFIILYMFYFMEATASGLLYDIKKYVDFLICVFWQMSHALSIIYANVYFSESITREVCKF

>HrhoGR2

MPITRSRPGTITFSWKSRATAYAIFFYIASTAVVLVVGYERIMILRSIKKFDDYIYAILFVVFLIPHFWIPFVGWGVAHEVAIYKTNWGKFQVRYYRVTGENLQFPNLKNLIVVISVGCLLLAVCFLLSLCALLDGFLLRHTAAYYHIITMINMNCALWYINCKAIKIASQSLSLCFRKVRKYNNSCVRRRLRDCGPRVWIQL*

>HrhoGR3

MITFANMSARIIIEAMGSETGFIFGVLEFTALSSLMRLGTCAAVMICVVSYCERVYRQRERILTIIDHLFISKMINAETRKSMNELRELVQSRTICFHMANLVVIQYSLLVSVASVVVTYTIILLQSIK*

>HrhoGR4

DVADEIRAMIVETDLKSKSAIIELRNMVQSRPITFTAANFFRIDYALLVSTATACITYTIILAKL*

>HrhoGR7

SNAQATFLWTFNDIMTMMFSIYLIAYFRDMNRLIYSPIPKNIHVWNRLRIFCSELVSLVQLVNPRLKYFILNSIGCSLY

>HrhoGR29

MSLICCFPKIMYILYGILKLYKARASALPIVVLGFGALQWGLMPCLPGAVMEFAYNEVEKIKKTLVHQCQYNKDELLREDIKEFIQYIDSRPYKYRILRMITVDMSLPIGLLKLCTTYFIVIIQFTHLFE*

>HrhoGR64a

MKVNGTYKRYDHQDEILERDEFLDTLNTIFKKSRWFGVAPRRRSLLFIWAIINAVLMMAVEAGAIWKLIRAITGTVFNTAGGHSLVARLSGSIFYANGLLSLALSWRLITSWSSVHSYWIKTELNRSLFLPPDVHIKKRVIFITSLVVTCALGEHILSMISAIGFRCPPSEYMEKYILVSHGFLIHKNEYHIWLAIPIFIVSKTATVLWNFQDLIIILLSTGLTSRYKRLNSYVKNLVEIEQSQEMKKHANEIYIEVQTWRRVREAYIHQANLVRRVSNKLGALILLSSLNNFYFICLQLFLGINKDKGEMINRLYHFLSLSWLMLRASGVALVGADVDEHSRKALKYMKMCSNHNVEIERLKNQMKNDRVVLRGLGFFALDRNMFLKVAAAIMKYELVLVQYDK*

>HrhoGR67

KRRIVEVDEAIKNMDEQIDYVRQVHSVWTIAVAPVVVSLMRVFSIYISIATSDIAVPIEKMGQLIYADIFALLITALHCGHNNLLRERFKIVNVTLRKIKDRKAWFRGALFSRISISDTKHVAQHREKYICDKIKACAKIYDKLMGCVISLNTIYGFAMVQTMSLSLVYIVLYLFYLMEATASGLYNDANRYVNFIFYVSWQILYGVGVIFFNIHYCEETVKEAKITSRIV

>HrhoGR68.1

MFRVISKIIPIYRQLNVTWYSAFKPIYYLTSLLGLFPYTLKHESTNVFKTYLNSYYLNFIYASIIFIILCVFCVLHIQDVIYAGKSYSMTNENLTEINYIFEFVFLLVYCFVAYYCAFTNGKLYINILNRVIATCRRTAVDRYEKNMKLVDKRLKKIVYSYTLLAVTTIIINFTRKGSVWKSVLVLFTFILPQTVQLATLAHYCSLVVMITGLLDNIILCLSYYDQNKRVAHGIWTTKPRAVSILSNLKTCFIKVYNIKEDINRAFQAPILFTAIQCFHCLVSEAGSLYHGVG

**The amino acid sequences of SNMPs used for building phylogenetic trees are listed below:**

>HrhoSNMP2

MMYEKWRKLPMPLNFKIYVFNVTNVEEVNAGANPKLVEIGPYVYKEYRERTDIEVTDNDTVRYMLKKSFVFDGEASGSKTEDDIITVIHYAYVAAIVQVHDTMASLLPILNPALQEFFGNVSSPFLTIKVKDLFFDGIFLNCNGSQQSLGLICSKIEVEKPPTMRQADGGNGFFWSMFGHLNRTITGPYEMARGLTNIQELGHIVSYQGKRVMTEWNDPYCGQINGSDSTIFPPIDENNVPSRLYSFEPDICRSLYISLSEKTTRFNMTAYLYEMDSSALASKSANPDNKCFCDKNWSANHDGCLVMGVLNLMPCQGSPAIVSLPHFYLASEEILSYIAEGIDAVKEKHKSYVYIDPSTGVPLDGLKRLQFNIELRKIPNIKQFENVKTGLFPLLWIEEGAVLPESLLSELRQGHTMIKYVEVFRWVLLAVALIVTAVSGYLVARAKSLVWPHHAPVSFVLQPHGMSEVNKVH*

>HrhoSNMP1

MKLRKHFKIAIGSAIVGVFGVLFGWLIFPTVLKSQLKKEMALSKKTDARKMWEKVPFALDFKVYLFNYTNVDEIHKGGVPIVKEVGPYYFEEWKEKVDIVDNDEDDTVTYKKLDTFYFKKDKSGPGLTGDEIITLPHAFILSLVTIISRDKPAMLNMAGKALNGIFDNPPNMFLKARALDVLFDGININCARTEFAPKAVCTALKKEAGNQLKILENNQFLFSFFGMKNHTVDSHVVKVSRGMKNVMDVAKVLEIDGKPQMDKFRDKCDLFDGTDGTKFPPFMTNQPVASFSTDTCRTFKPWYQKQSSYQGIKTLRYISNIGDYANDPELNCFCDTPDSCPKKGFMDATKCLSAPLYVTLPHLLDCDPEEQKNVKGLSPDVEAHGIAIDFEPITGTPMTARQRVQFNLRLIKTDKIEQCKELPDTIAPLFWIEEGYALDRDFVKLLKHQLFLPKRIVGVVRWLLVSIGILGTFGSLVFHFKDRIIQFAIPSNATSVTKIKPEEENKKQISVIGNTQDATELAKIDM*

>AipsSNMP1

MAMAKELKYAAIAGGVAVFWLIFGFILFPVILKGQLKKEMALSKKTDVRKMWEQIPFALDFKVYFFNFTNAEEVQKGAKPILKEIGPYHFDEWKEKVEIEDNEDEDTVNYKKRDVFYFNPEMSAPGLTGEEIIVLPHIFMMAMALTVHRDKPAMLNMIGKAMNGIFDDPPDVFMRVKALDILFRGIMINCARTEFAPKATCTALKKEKVTGLIVEANNQFRFSLFGARNGSIDEHVITVRRGIKNVMDVGKVIAIDGKPEQTIWRDHCNEFVGTDGTVFPPFLKETDRIESFSTDLCRPFKPWYQKKTSYRGIKTNRYIANIGNFAEDPELQCFCPAPDRCPPKGLMDLVPCMKAPMFASMPHFLDSDPALLDNVKGLNPDINEHGIEIDFEPITGTPMVAKQRIQFNIQLLKTDKMELFKDLSGDIVPLFWIDEGLALNKTFVNMLKHQLFIPKRVVGVLRWWMVSFGSLGAVIGIVYHYRDHIMRLAVSGDTKVSKVTPEDGPEQKDISVIGQEPAKINI

>AipsSNMP2

MFGKYAKLFLTVSLGFLVVAIIMVAWGFEKIVDKQIQKNVQLENNSMMFDKWLKLPMPLDFKVYIFNVTNVDEVNRGQKPMLQEIGPYVYKEYRERTILGYGENDTIKYMLRKRFEFDPEASGGLTEDDEVTVIHFSYLAALLTVHDMMPSLVGVINKALEQFFPSLEDAFLRVKVRDLFFDGIYLSCDGDNAALGLVCGKIKGEMPPTMRAAEGANGFYFSMFSHMNRSESGPYEMIRGRDNVYELGNIVTYKGQQVMSMWGDKYCGQINGSDSSIFPPIKESNVPKKLYTFEPDICRSLYIDLVGKTEMFNISSYYYEISESALAAKSANHDNKCFCKKNWSANHDGCLLMGLLNLMPCQGAPAIASLPHFYLASEELLDFFQSGIMPDKEKHKSYVYIDPTTGVVLSGYKRLQFNIELRKIDNVPQLSTVPTGLFPMLWLEEGATIPPSIQQELLDSHKLLGYVEVARWFLLTVAIIAVIVSAVAVARANALLSWPRNSNSVSFISGPGVTMVNKGN

>BmorSNMP1

MQLAKPLKYAAISGIVAFVGLMFGWVIFPAILKSQLKKEMALSKKTDVRKMWEKIPFALDFKIYLFNYTNAEDVQKGAVPIVKEVGPFYFEEWKEKVEVEENEGNDTINYKKIDVFLFKPELSGPGLTGEEVIVMPNIFMMAMALTVYREKPAMLNVAAKAINGIFDSPSDVFMRVKALDILFRGIIINCDRTEFAPKAACTTIKKEAPNGIVFEPNNQLRFSLFGVRNNSVDPHVVTVKRGVQNVMDVGRVVAIDGKTKMNVWRDSCNEYQGTDGTVFPPFLTHKDRLQSFSGDLCRSFKPWFQKKTSYNGIKTNRYVANIGDFANDPELQCYCDSPDKCPPKGLMDLYKCIKAPMFVSMPHYLEGDPELLKNVKGLNPNAKEHGIEIDFEPISGTPMVAKQRIQFNIQLLKSEKMDLLKDLPGTIVPLFWIEEGLSLNKTFVKMLKSQLFIPKRVVSVVCWCMISFGSLGVIAAVIFHFKGDIMHLAVAGDNSVSKIKPENDENKEVGVMGQNQEPAKVM

>BmorSNMP2

MLAKYTKTIFSVSVAFLVVSIVLATWGFPKIIRKQIQKNVQISNTSKMYDKWVKLPMPLDFKIYVFNVTNRDAINQGEKPNLKEIGPYVYKQYREKIILGYGDNDTIKYNLKKTFVFDPVASGDLREDDELTVINFSYMAAIISVQEMMPAAVGMINRALEQFFTNLTDPFQTVKVKDLFFDGLFLNCEGDNTALGLICGKIRAEKPPTMRISKSANGFYFSMFSHMNRTVSGPYEMVRGTENLSDLGHVISYQGKRIMSAWDDQYCGQLNGTDSTIFPPLEDGNIPEKLYTFEPDICRSLFASLVGKDTLFNISTYYYEISDMTLGSKSANPDNKCFCKRNWSVKHDGCLLMGVLNLAPCQGAPAIASLPHFYLGSDELADFFGDGIKPDKEKHNTYVHLDPITGVVIKGVKRLQFNIELRNVPSVPQLKEVPSGLFPLLWIEEGAEIPEWLRKEIMDSHTMLWYVDAARWLVLAVAVVAVLVSATLVARSAALIPWPRNSNSISFILGNSVNTSKVHS

>CmedSNMP1

MQLQKHMKIGLGTAGAGIFGILFGWVLFPVILKSQLKKEMALSKKTDVRAMWEKIPFPLNFKVYMFNYTNPEEVQKGGIPIVKEIGPYHFDEWKEKVGIEDHEEDDTITYKKRDVFIFRPDLSGPGLTGEEIVVIPHVLMMGIATIVNKEKPTMLGMLSKAFNGIFDEPKDVFLRAKVLDLLFRGIIINCQRTEFAPKAVCTALKKEAATGLSFEANNQYRFSLFGLRNGTIDPHVVTVKRGIKNVMDVGKVIAVDGKPEQDVWKDKCNEYQGTDGTVFPPFLTEKDNLESFSGDLCRSFKPWYQKKTSYRGIKTNRYVANIGDFANDPDMQCFCDTPDTCPPKGVMDLMKCMKAPMYASLPHFLDSDPNLLKHVKGLSPDANEHGIEIDFEPISGTPMVAKQRVQFNMLLLKADKLELIQDLPNTLSPVFWIEEGLALNKTFVKMLKTQLFIPKRIVGVVKWLLVTVGVIGMIGTAVYHYKGNIAAFALKPSSATVTKVNPETQNQPKDISVIGEPQNPPKVDM

>CmedSNMP2

MLGKHTKLFFAVSLFAVVISVILATWGFPKIVKKQIQKNVQLDESSQMFEKWRKLPMPLTFNVYVFNVTNVDDVNEGARPKLQELGPYVYKEYRERTILGYGDNNTVKYMLKKTFLFDQEASGALSQDDEVVVINFSYLAAILTVQDMMPSIVGVVNGALEQFFTNLTDAFLRVKVRDLFFDGVHVNCNGNHSALGLVCGKLKTDAPPTMRPAEDGSGYYFSMFSHMNRTETGPYEMVRGRDNIKELGHIVSYKGKASMGGRWGRDPYCGMLNGSDASIFPPIDEADVPDKLYTFEPEVCRSLYASLVGKSSIFNMSAYYYEISRLALASKSANPDNKCFCKKDWSSNHDGCLLMGVLNLMPCQDAPAIASLPHFYLASEELLEYFDGGISPDKEKHNTYMYLEPVTGVVLKGIRRLQFNIELRNIPMVPQLAKVSTGLFPLLWIEEGAELPESVLDELRHSHKLLGYVEAVRWVLLCVSLAALLASGVAVLRAGLLPVWPRNNSVSFILSPHPAENKVH

>CsupSNMP2

MLAKHMKVFFLASLAALVLAVILAAWGFPRIVSKQIQKNVQLENSSVMFEKWRKLPMPLTFKIYVFNVTNAEDINSGAKPMLTEIGPYVYKEYRERTILGYGENDTVRYTLKKTFIFDAEESGPLTENDEVVVINFSYMAAILAVQEMMPSLTTVVNQALEEFFTDLKDPFMRIKVRDLFFDGIHVNCVGNHSALGLVCGQLKSDTPPTMRPTEDGTGYYFSMFSHMNRTESGPYDMVRGTEDIRELGHVVAYKGERSMSQWGDPYCGQLNGSDSSIFPPIDGGNVPQRLYIFEPEICRSMFATLVGKTTVFNMSAFHYSISSDVLAARSANPNNKCYCRKNWSANHDGCLLMGVMNLAPCQGAPAIASLPHFYLASEELLQYFASGINPDKEKHDTYLYLEPVTGVVLKGLRRFQFNIELRNIPEVPQLAKVPTGLFPLLWIEEGATLPDSVVKELQSSHKLLSYVEAARWILLVVAVIATVVSAVTLARSGVLPVCPRNSNSVSFILNPHPTVIDVNKVH

>DpunSNMP1

MQLARELKCAAVGGGVAIFGILFGWIIFPTVLKSQLKKEMALSKKTDVRQMWEKIPFPLDFKVYFFNYTNPEEVHNGAVPIVKEIGPYYFEEWKEKMEVQDHEEDDTITYKKKDVFYFKPELSNGLTGEELIIVPHVFIMAMILSVARDKPGMLNVAGKALNGIFDNPPDVFFRGKPMDLLFRGIIINCDRSEFAPKAACTTIKKEKPAGVIFEPNNQFRFSLFGTKNGTIDTHVVTVKRGIKNVMDVGQVIAIDGKPELDIWRDSCNQYQGTDGTVFPPFLTRKDRLQSYSGDLCRSFKPWYQKKSSYKGIKTNRYVANIGDFASDPELQCFCDSIDTCPPKGLMDLMKCMKAPMYASMPHFLDADPEIQKNVIGLNPDVNEHGIAIDFEPISGTPLVAKQRIQFNMQLYKTEKIAICKNLPNTIVPLFWIDEGLALNKTFIKMMKRQLFTPKKVVSVLKWMMVFFGFLSAIGGVAYHHKGRIMRGAGTGESKVTKVNPEDVEQKEISVIGQGQEPAKVM

>DpunSNMP2

MLFKNSKLMFMLSVAFLIVAIILATWGFPKIINRQIQKNIQIENSSVMYEKWVNVPVPLDFKVYLFNVSNAEEVNEGSKPKLMEIGPYVYKQTREKEVLGYGENDTIIYNLKKTFVFDPEASNGLSEDDDITVINFSYMGAILTINDMMPAGVAYINKALEEFFTNLTDPFSRVKVRDLLFDGIFLNCVGNNSALGLVCAKIRAEAPSTMRKAEEEGVNGFYFSMFSHMNRTASGPWAMKRGVDNMYEIGEIVSFKNQKAMRVWGDPYCGQINGSDSTVFPPIDEAKVPERLYTFEPEICRSLYVSLVGRRTLFNISAYYYELPESALAAKTANPDNKCFCKKNWSSNHDGCLLMGLLNLMPCQGAPAIASLPHFYLGSEELLDYAEGLKPDREKHSSFVYLDPTTGVALKGVKRLQFNIELRNMPKVPQLKSVPTGLFPLLWIEEGAELPTTLQEELREAHSLLSYVEAARWLILAIAMVCCVVAAAAVARAASLLSWPRNRNSVSFILGPTVPHVNKVQGNQ

>HarmSNMP1

MQLPRELKYAAIAGGVALFGLIFGWVLFPTILKSQLKKEMALSKKTDVRKMWEKIPFALDFKVYIFNFTNAEEVQKGATPILKEIGPYHFDEWKEKVEVEDHEEDDTITYKKRDVFYFNPEMSAPGLTGEEIVVIPHIFMLGMALTVARDKPAMLNMVGKAMNGIFDDPPDIFLRVKALDILFRGMIINCARTEFAPKATCTALKKEGVSGLVLEPNNQFRFSIFGTRNNTIDPHVITVKRGITSVMDVGQVVAVDGKTEQTIWRDTCNEFQGTDGTVFPPFVPETERIESFSTDLCRTFKPWYQKKTSYRGIKTNRYIANIGDFANDPELNCYCAKPDTCPPKGLMDLAPCMKAPMYASMPHFLDSDPALLSKVKGLNPDVTQHGIEIDYEPITGTPMVAKQRIQFNIQLLKTDKLDLFKDLSGDIVPLFWIDEGLALNKTFVNMLKHQLFIPKRVVGVLRWWMVSFGSLGAVIGIVFHFRDHIMRLAVSGDTKVSKVTPEEEEQKDISVIGQAQEPAKVNI

>HarmSNMP2

MCGIIGGVSTLVVGSVLVVASSVISFVFVPNIIRNIIAGEVTLLDDTIQMERFKEVPFPMNFTVRVFNMTNPAQVLTGGVPVMQEIGPYVYRLYQTREILEKDGDVIVYKMHEHFEFDADLSYPNQEDDLVTIINVPFHAVIQVAESLFPYLMSLLQMAMDEVFKEFNTPITTIRVRDLLFDGITMCKHPTGLGIIACSIIRDIADNAQNIEEKPDGSLVFSILNYKQQLPSQEYRVQRGLDDPADLGRILSYAGSPFFPQWLNLTTREPNVCMEVNGTDAGIFAPFVETERSIYAINTDICRSVELRYERDSEYEGIPTVRFAANEWLLDNDDGCFCLNVTRGINRDDGCLLRGAMELYTCVGAFLIMSYPHFLFADVRYRDSVLGMHPNEENHKIFIELEPNTGTPIRGAKRAQFNIFSRPVRNIPVTQNLRTAIVPILWIEEAIDLPHEFVDELTERLLSSLQLVDIFIPVLIAACVMVLVVGVALTARARVYRK

>HvirSNMP1

MQLPKELKYAAIAGGVALFGLIFGWVLFPTILKSQLKKEMALSKKTDVRKMWEKIPFALDFKVYIFNFTNAEEVQKGATPILKEIGPYHFDEWKEKVEVEDHEEDDTITYKKRDVFYFNPEMSGPGLTGEEIVVIPHIFMLGMALTVARDKPAMLNMVGKAMNGIFDDPPDIFLRVKALDILFRGMIINCARTEFAPKATCTALKKEAVSGLVLEPNNQFRFSIFGTRNNTIDPHVITVKRGIKNVMDVGQVVAVDGKLEQTIWRDTCNEYQGTDGTVFPPFVPETERIQSFSTDLCRTFKPWYQKKTSYRGIKTNRYVANIGDFANDPELNCFCPKPDSCPPKGLMDLAPCMKAPMYASMPHFLDSDPELLTKVKGLNPDVTQHGIEIDYEPITGTPMVAKQRIQFNIQLLKTDKLDLFKDLSGDIVPLFWIDEGLALNKTFVNMLKHQLFIPKRVVGVLRWWVVSFGSLGAVIGIVFHFRDHIMRLAVSGDTKVSKVTPEEPEQKDISVIGQAQEPAKVNI

>MsepSNMP1

MLLPKELKYSAIAGGVAVFGLIFGWVLFPVILKGQLKKEMALSKKTDVRKMWETIPFALEFKVYLFNYTNAEEVQKGAKPILKEIGPYHFDEWKEKVEIEDHEEDDTITYKKRDTFYFNPELSAPGLTGEEIVVMPHIFMLGMALTVNRDKPAMLNMVGKAMNGIFDNPPDIFMRVKALDILFRGIIINCARTEFAPKATCTALKKEGVSGLIIEPNNQFRFSIFGTRNNTIDPHIITVKRGIQNVMDVGQVVAVDGKPEQTIWKGACNEYQGTDGTVFPPFLTENDRIQSFSTDLCRSFKPWYQKKTSYRGIKTNRYIANIGNFAEDPELQCFCPDPDKCPPKGLMDLAPCIKAPMYASMPHYLESDPALLNNVKGLNPDINQHGIEIDFEPISGTPMVAKQRIQFNLQLLKTDKIDLFKDLSGDIVPLFWIEEGLALNKTFVNMLKHQLFIPKRVVGVLRWWMVSFGSLGAVIGIVYHFRDHIMRLAVSGDTKVSKVTPEEGQEQKDISVIGAQAQEPAKINI

>MsepSNMP2

MLGKHSKLIFAVSIGFLVVAIIMASWGFQKVVDKQIQKSVQLENDSLMFDKWLKLPMPLDFKVYVFNVTNVEEVNKGEKPILEEIGPYVYKQYRERTVLGYGPNDTIKYMLRKRFEFDAEASGGLTEDDEVTVIHFSYLAALLTVHDMMPSLVGVINKALEQFFPSLEDAFLRVKVRDLFFDGIYLSCDGDNAALGLVCGKIKGDLPPTMRLAEGSNGFYFSMFSHMNRSETGPYEMNRGRDNIYELGNIVTYKGQEIMPMWGDKYCGQINGSDSSIFAPINEANVPQKLYTFEPDICRSLYVDLVEKRELFNISAYYYEISESALAAKSANHDNKCFCRKNWSANHDGCLLMGLLNLMPCQGAPAIASLPHFYLGSEELLEFFQSGIAPQREKHNSHVYIDPTTGVVLSGVKRLQFNIELRKIDTIPQLSSVPTGLFPMLWLEEGATIPDSIQQELRDSHKLLGYVEVARWLLLTVAVIAVIASAVAVARANALLSWPRNSNSVSFILGPSVTQVNKG

>MsexSNMP1

MRLARGIKYAVIGAGVALFGVLFGWVMFPAILKSQLKKEMALSKKTDVRKMWEKIPFALDFKIYLFNYTNPEEVQKGAAPIVKEVGPYYFEEWKEKVEIEDHEEDDTITYRKMDTFYFRPELSGPGLTGEETIIMPHVFMMSMAITVYRDKPSMMNMLGKAINGIFDNPSDVFMRVNAMDILFRGVIINCDRTEFAPKAACTAIKKEGAKSLIIEPNNQLRFSLFGLKNHTVDSRVVTVKRGIKNVMDVGQVVAMDGAPQLEIWNDHCNEYQGTDGTIFPPFLTQKDRLQSYSADLCRSFKPWFQKTTYYRGIKTNHYIANMGDFANDPELNCFCETPEKCPPKGLMDLTKCVKAPMYASMPHFLDADPQMLENVKGLNPDMNEHGIQIDFEPISGTPMMAKQRVQFNMELLRVEKIEIMKELPGYIVPLLWIEGGLALNKTFVKMLKNQLFIPKRIVSVIRWWLLSFGMLAALGGVIFHFKDDIMRIAIKGDSSVTKVNPEDGEQKDVSVIGQSHEPPKINM

>MsexSNMP2

MLAKHSKLFFTGSVVFLIVAIVLASWGFPKIISTRIQKSIQLENSSMMYDKWVKLPIPLIFKVYFFNVTNAEGINEGERPILQEIGPYVYKQYRERTVLGYGPNDTIKYMLKKNFVFDPEASNGLTEDDDVTVINFPYMAALLTIQQMMPSAVAMVNRALEQFFSNLTDPFMRVKVKDLLFDGVFLNCDGDSPALSLVCAKLKADSPPTMRPAEDGVNGYYFSMFSHLNRTETGPYEMVRGTEDVFALGNIVSYKEKKSVSAWGDEYCNRINGSDASIFPPIDENNVPERLYTFEPEICRSLYASLAGKATLFNISTYYYEISSSALASKSANPDNKCYCKKDWSASHDGCLLMGVFNLMPCQGAPAIASLPHFYLASEELLEYFEDGVKPDKEKHNTYVYIDPVTGVVLKGVKRLQFNIELRNMPRVPQLQAVPTGLFPMLWIEEGAVMTPDLQQELRDAHALLSYAQLARWIILAAAIILAIIATITVARSTSLISWPRNSNSVNFIIGPMVNDKMR

>OfurSNMP1

MQLQKPLKIGLGMMGAGLFGIIFGWVLFPVILKSQLKKEMALSKKTDVRAMWEKIPFALDFKVYMFNYTNVEEIMKGAAPIVKEIGPFHFDEWKEKVDIEDHDEDDTITYKKRDYFYFRPDKSGPGLTGEEVVVMPHLLMLSMATIVNNEKPAMLNMLGKAFNGIFDEPKDIFIRVKVLDLLFRGIIINCARTEFAPKAVCTALKKEGATGMTFEPNNQFRFSLFGMRNGTIDPHVVTVRRGIKNVMDVGKVIAIDGKTEQDVWRDKCNEFEGTDGTVFPPFLTEKDNLESFSDDLCRSFKPWYQKKTSYRGIKTNRYVANIGDFANDPELQCYCDSPDKCPPKGLMDLMKCMKAPMYASLPHYLDSDPQLLKDVKGLSPDANEHGIEIDFEPISGTPMVAKQRVQFNIILLKTDKMDLI

KDLPGTMTPLFWIEEGLALNKTFVKMLKNQLFIPKRIVSVVKWLLAGVGFVGLVGSLVYQFKGKMINFALSPSSAQVTKVNPEINQQNQPKDISIIGESQNPPKVDM

>OfurSNMP2

MLGKHTKLFFGVSLVALIVSVILAAWGFPKIVSKQIQKNIQIDNSSVMFEKWRKIPMPLTFNVYVFNVTNVEDVNNGAKPRLQQIGPYAYKEYRERTVLGYGDNDTVSYTLKKTFIFDQEASGLLSEDDEVTVIHFSYMAAILTVNDMMPSITGVVNGALEQFFTNLTDPFLRVKVKDLFFDGVYVNCAGNHSALGLVCGKLKADAPQTMRPAGDGNGFYFSMFSHMNRTESGPYEMIRGRENIKELGHIISYKGKSFMKNWGNDMYCGQLNGSDASIFPPIDENNVPEKLYTFEPEVCRSLYASLVGKSSIFNMSAYYYEISSDALASKSANPGNKCYCKKNWSANHDGCLIMGILNLMPCQDAPAIASLPHFYLASEELLEYFDGGISPDKEKHNTYIYLEPVTGVVLKGLRRLQFNIELRNIPMVPQLAKVPTGLFPLLWIEEGAELPDSIIQELRQSHTLLGYVEAVRWALLAIAIVATAISAIAVARSGLIPVWPRNANSVSFILSPHPNSDVNKVH

>PxylSNMP1

MKLPKHLKFAAGAGGAFLFGILFGWVMFPAILKGQLKKEMALSKKTDVRKMWETIPFALNFKVYLYNYTNPEEVQKGGVPIIKEVGPYHFDEWKEKVEIEDHEEDDTITYKKRDTFYFNQEKSGPGLTGEEVITMPHVFMLAMATVVSREKPAMMNMIGKAINGIFDNPADVFIRVKALDIMFRGTMINCARTEFAPKAVCTALKKEAVNGLVMEPNNQFRFSLFGSRNGTIDPHVVTVKRGIKNVMDVGQVVAIDGKPQQDVWRDHCNEYQGTDGTVFPPFLTEHDRLQSFSGDLCRSFKPWYQKKSFYRGITTHRYIANIGDFANDPELNCFCDGPCPPKGLMDLMKCMKAPMYASMPHFLDSDPELLKNVKGLNPDVNEHGIEIDFEPISGTPMVANQRVQFNMQLLKHDKVELLNNLPDTIVPLFWIDEGLALNKTFVNMLKFQLFYPKKAVGVIKWLLVTFGGFGLIGCTIYHYKDRIMSFASSPGSAAVTKVKPEEVEQKDVSVIGQPQEPAKINM

>SexiSNMP1

MLLPKELKYAAIAGGVALFGLIFGWVLFPTILKSQLKKEMALSKKTDVRQMWEKIPFPLDFKVYIFNYTNAEEVAKGAVPILKEIGPYHFDEWKEKVEVEDHEEDDTITYKKRDVFYFNPEMSGPGLTGEETVVIPHVFMLGMALTVHREKPAMLNMVGKAMNGIFDDPPDIFLRVKAMDILFRGMMINCARTEFAPKATCTALKKEGVSGLVLEPNNQFRFSIFGTRNNSIDPHVITVKRGIKNVMDVGQVTAVDGQTVQTIWKDHCNEYQGTDGTIFPPFLTENDRLQSFSTDLCRSFKPWFQKKSSYKGIKTNRYVANIGNLAEDPELQCFCPQPDKCPPKGLMDLAPCIKAPMYASMPHFLDCDPALLSKVKGLNPDVNAHGIEIDFEPISGTPLVARQRLQFNIQLLKTDKLDLCKDLSGDIVPLFWIEEGLALNKSFVNMLKHQLFIPKRVVGVLRWWMVSFGSLGALIGVVFHFRDHIMRLAVSGDSKVSKVTPEEGEEQKDISVIGPAQEPAKINI

>SexiSNMP2

MLGKHSKLIFAVSMGFLVVAVIMAAWGFQKIVDKQIQSNVQLENNSMMFDKWLKLPMPLDFKVYVFNVTNVEDVNRGEKPILNEIGPYVYKQYRERTILGYGPNDTIKYMLRKRFEFDPVASGDLTEDDEVTVINFSYLAALLTVHDMMPSFVGMVNKALEQFFPSLEDAFLRVKVRDLFFDGIYLSCDGDNAALGLVCGKIKSDTPPTMRPAEGANGFYFSMFSHMNRSESGPYEMVRGRENVYELGNIVSYKGQKVMPMWGDKYCGQINGSDSSIFPPIKEGNVPKKLYTFEPDICRSVYVDLVGKKEIFNISAYYYEISESAFAAKSANPNNRCFCKKNWSANHDGCLLMGLLNLMPCQGAPAIASLPHFFLGSEELLEYFGSGIKPDKEKHNTYVYIDPTSGVVLSGLKRLQFNIELRQIDTVTQLKRVPTGLFPMLWLEEGATIPASIQQELRDSHKLLGYVEIARWFLLTVAIIAVVTSAVAVARANALLSWPRNSNSVSFILGPSVTHVNKGN

>SlitSNMP1

MLLPKELKYAAIAGGVAIFGLIFGWVLFPTILKSQLKKEMALSKKTDVRQMWEKIPFPLDFKVYIFNYTNAEEVAKGAVPILKEIGPYHFDEWKEKVDVEDHEEDDTITYKRRDVFYLNPELTAPGLTGEEIVVIPHVFMLGMALTVQREKPAMLNMVGKAMNGIFDDPPDIFLRVKAMDILFRGMIINCARTEFASKATCTALKKEAVSGLVLEPNNQFRFSIFGTRNNTIDPHVITVKRGIKNVMDVGQVVAVDGQTEQTIWKDTCNEYQGTDGTVFPPFLTENDRLQSFSTDLCRSFKPWYQKKSSYRGIKTNRYVANIGNLAEDPELQCFCPQPDKCPPKGLMDLAPCIKAPMYASMPHFLDCDPALLSKVKGLNPDVNAHGIEIDFEPISGTPLVARQRIQFNIQLLKTDKLDLCKDLSGDIVPLFWIEEGLALNKTFVNMLKHQLFIPKRVVGVLRWWMVSFGSLGAVIGIVFHFRDHIMRLAVSGDSKVSKVTPEEVEEQKDISVIGPAQEPAKINI

>SlitSNMP2

MLGKHSKLIFAVSMGFLVVAVIMAAWGFQKIVDKQIQKNVQLENNSMMFDKWLKLPMPLEFKVYIFNVTNVEDVNQGEKPILNEIGPYVYKQYRERTILGYGPNDTIKYMLRKRFEFDPEASGVLTEDDEVTVINFSYLAAVLTVHDMMPSFVGMVNKALEQFFPSLEDAFLRVKVRDLFFDGIYLNCDGDNAALGLVCGKIKSDTPPTMRPAEGANGFYFSMFSHMNRTETGPYHMIRGRENVYELGNIVSYKEQKVMPMWGDKYCGQINGSDSSIFPPIKEGNVPKKLYTFEPDICRSVYVDLVGKKEIFNISAYYYEISESAFAAKSANPNNKCFCRKNWSANHDGCLLMGLLNLMPCQGAPAIASLPHFFLGSEELLEYFGSGIMPDKEKHNTYVYIDPTSGVVLSGLKRLQFNIELRQIDTVPQLKRVPTGLFPMLWLEEGATIPASIQQELRDSHKLIGYVEVARWFLLTAAIIAVVTSAVAVARANALLSWPRNSNSVSFILGPSVTQVNKGN
