# Supplementary material for: Identification and expression profiles of candidate chemosensory receptors in Histia rhodope (Lepidoptera: Zygaenidae)
Source: PeerJ. 2020 Sep 24;8:e10035. doi: 10.7717/peerj.10035 (PMC7520089; doi:10.7717/peerj.10035)
Supplement: Table S1 [file peerj-08-10035-s002.docx]

**Table S1 Primers designed for fluorescence quantitative realtime PCR**

| **Primer name** | **Forward primer （5' to 3'）** | **Reverse primer （3' to 5'）** |
| --- | --- | --- |
| Reference gene |  |  |
| GAPDH | CCTTCATCGGTCTGGACTACAT | TGCCCCAAGGAATAGATGC |
| ORs |  |  |
| HrhoORco | GCTTTGAAGAAGATGAGGA | GTAAGCACCATAAATAAGTT |
| HrhoOR4 | ACCGAACTGCTCGCTTAT | CGGTAAACAAGTTGGTAG |
| HrhoOR9 | GTATCACTTACAGCATTTTCTA | ACCTTTACTTGTTGTTTCA |
| HrhoOR11 | ATAGTAAACCGTCATCAAAG | AGAACCTAAAAGACTGCA |
| HrhoOR13 | GATACGGGCGTTGGATTA | AGTAGCAGGTCTCGGTTC |
| HrhoOR24 | GAACGAATAGGTGGTAGTG | CAAGTCCGTAGATGTAGC |
| HrhoOR26 | GATTTTCGTAGGACTGGC | GGTTTTACTCAGTTCACTA |
| HrhoOR33 | ATGGTGGTGGCATTGTTC | GATGCTGCTTGGGGCTGC |
| HrhoOR37 | TTGGATAGTGAATGGAGC | GAAAGCAAATAGACGGAG |
| IRs |  |  |
| HrhoIR8a | GGTGGTTGGGAATAAAGG | TAGAGCGAAGCAAGATAGC |
| HrhoIR21a | GGATTGGAACTTTAGACCG | CTCCCACGAAGTTTCACC |
| HrhoIR40a | CCTCGTGAACTGGTGGTC | CCTCGTGAACTGGTGGTC |
| HrhoIR41a | ACATAGGGTGCTCCGATTA | CTGAACATACCCTTTTGTGA |
| HrhoIR60a | TACAGTCAGTGGCGATTC | CGGTGGTGTTTATGATGC |
| HrhoIR68a | GAACGGGTCATTTTCAGG | ACAACGAGCAACAGAGCG |
| HrhoIR75p | GACCCAGCAGAAAGGAAC | ATAAGATGCGGCGATAGA |
| HrhoIR75q.2 | GTATTGTGCCACCGCTCTT | CAAGAAACCCAGCGTAAC |
| HrhoIR76b | AGGCTGGGGCAGTTTTAG | TACTTTGCTTGGTCTCGC |
| GRs |  |  |
| HrhoGR2 | CAAGAGCCACGGCATACG | CCTACCTAAAGACGACGCA |
| HrhoGR64a | TTGCCTCCCGATGTTCAC | CATCTCACTCCCTCCGCA |
| HrhoGR67 | CACAACAATCTTTTGAGGGAA | CCAAGCGATACCAGGTCT |
| SNMPs |  |  |
| HrhoSNMP1 | ATCGGTTCCGCAATCGTC | TATTGCTACTTCTACTATGGC |
| HrhoSNMP2 | ATGACAGCGTATTTGTATGA | TTGATGCGGTTCCAGTGT |
