# Supplementary material for: Identification and expression profiles of candidate chemosensory receptors in Histia rhodope (Lepidoptera: Zygaenidae)
Source: PeerJ. 2020 Sep 24;8:e10035. doi: 10.7717/peerj.10035 (PMC7520089; doi:10.7717/peerj.10035)
Supplement: Table S2 [file peerj-08-10035-s003.docx]

**Table S2. Amino acid sequences of chemosensory receptors of *H. rhodope* in the study.**

**ORs**

>HrhoOrco

MMTKVKTQGLVTDLMPNIRLMQIAGHFMFNYHDENAGMSFLLRKIYAGVHAFLFVIQYVCMGVNMAMYSEEVNELSANSITMLFFAHSLIKLLLFAINSKSFYRTLAIWNQSNSHPLFTESDSRYHQIALKKMRRLLYIICGVTLFSVTSWVTITFFGESVRFIVDKETNETLTEPAPRLPLKAFYPFNAMGGPMYVFAFIYQIYWLLYAMSIANLMDVLFCSWLIFACEQLMHLKAIMKPLMELSATLDTYRPNTAELFRASAEKSEKLPDPVDLDIRGIYSTQQDFGMTLRGGGGRLQTFGEPTPNNPNGLTQKQEMLVRSAIKYWVERHKHVVRLVTAIGDAYGTTLLFHMLVSTITLTLLAYQATKINGINVYMFSTVGYLLYTLGQVFHFCIFGNRVIEESSSVMEAAYSCQWYDGSEEAKTFVQIVCQQCQKAMSISGAKFFTVSLDLFASVLGAVVTYFMVLVQLK*

>HrhoOR1

MKRPTKHIISLINSIIFLFPLNGIKLEEINSMNTSMYRFFFVLNFLCMNFDAAGEIIWFLDGLWSGKEFVMLAHIVPCLIMTFLGNIKMIFHFLNEKEVVKLIINFKELEDDKNIELANSNEQKSTENIYDREIKHLNTIIKLVKVLNSSTIIAFGLAPFILMGAKYLKTKEFVPILPLYVKYHFFDPYNMIYYGLTYIHQFWSMCICMSSVTGVDMLFCTMCVFIKIH

>HrhoOR2

MHSCRIKPIDAFETSFQWLKLTGYFIAVPNIENPTKALLHNIYRAFTVFILIVYELQHLIFIIQVFGNVEYMIDGLVVLITSLYETIKLFLVNINSRRFEYLNDILNDDMFCAYLPIDEEIMRENKEQLERLSKIIYRTIAVTGAFWLIAPFLNKMSDGHPLLAAYFPFDTNDWFGFTCANIWLTTIIVWVGYGHMSLNILLIGYYSQVKVQLRIIRHHLEHIADDHVNSTEANKYTNYLDTVCCDVEKEFIVLLQRYEKVVRFYQEVELLLDRAMLVQFCGSTGIICAVVYKMTDIPINTTFLYLALYLAGLLLELFIYCYYGTL

>HrhoOR3

MVSKKVSSLVKELEDPNHPLLGPNIKAFYFFGFLQSYHTIQNICYKAWYTIGFFYVVTQWIELWLLKGDLNKALENLSISTLAIMSTTKGVTTVIWQKYWKELLENISTEEKKQINKKDNTTMKLMKNYKNYSRIITYFYWFLVSATVSMVITSPFLRYMYLKSRINDQNNSTISYPEIASSWYPFDKTNILIHSMKCFLDAIMLGQGATVIATYDSTVIVIMIFLKGQMRILQENCKKMFQEKQISNHVVINRIKNCYEHHQFLVRQHNLLNSLLSPIMFMYVLLCSIMICCSVYQLTLEEATTYQRLWFVEYTIAIVFQLFLYCWHSNEVAVESDLLDRGLYESDWWKSDINIRPIFILLAGKLNRIFVLEAGPFTTLSVATFIKIMKGAYSFYTLFTQMQK*

>HrhoOR4

MGLTKIQQENKYHSIIMFVYICGLPHFWYKDVDWSPRKKNLLKFASKFINHIGNLFFITELLAYFTQKELDEQQFSFWFGCAFTHTMCISAIFSLVYHKKNIESLITRMIVTIPNIHHDEEVSEKMIKKCFLYVLTSISTLNLTVLFHGVKASKEYMNGGIFLPVITFWPKTSDLSTAATIGRFVAYIMWWIWVARVSGILTTAIILTICSSHLFNHLQTYFKKMSTIFEENLTIDQKQQKYEASMKVAFKMHHDILNHIEVLIDVCNVTYGGQILMNVSILTIMMFQLASLEHFSIVEILPHIMIMITVLTVTACYMWSLGDVTIESAELSNAIYMSGWENCQNDYSIKMRRLVMIAMTQTQKPLETKTLGLIPVSHESYVSIVKASYSIFSLIFYNNT*

>HrhoOR5a

MCTKASKGVRAAVVRLRVCGFYRLGAGAVAAEAHMAYRALMLILTAIYLLQEVVYAVCERYDMDKLARVMFLLLCHFTSIVKQIVFFVDADRIDNLITLLDEPIFSRGPATLEAAALGAERLGRAYSGTAAVTCMLWTVFPVLNFLQGHHVEFPIWTGSINYNSNSLALLCFTFLYQLRYSLLTFSLLLIEIGGANTNINRMITKSKITYLPISRSYFCYVI*

>HrhoOR5b

MDAFLGTLLHQAKTQLSILRGNYENITERAKIVAQLTKEDYDKVLKRLFVDCLTHFKKVSELLELLQSIFSSAIVVQFTIGGWILCMAAYKIIELNVLSIEFTSMVLFILCILTELFIYCYYGNEVTLESERVAGSVYAAQWVPAPAWFRRALLAALVRARRPLRPLAGRVLPLSLNTFLKILKSSYSFYAVLRQTKNQV*

>HrhoOR6

MRNKIVSYLENEEHPLLGPTLWGLRAWGLYPPINSNWYTIISCSVHLAAIAFVVTQYIELWLIRFNLNLAMRNLSITMLSSICVIKAGTFVLWREDWMKIINFVSSLEKTQLSETYAIKNNIISRYTKYSRCVTYIYWGLVTATVFTVVMAPLAIFLSSPSTNELMRNGTIPYPEIMSSWTPFDRTRGYGYWACVVEHMLICFYGGGIVANYDSNAVVLMSFFAGQLKLLSANCTMLFGDENEVVSYNEALRRIRDCHYHHVQLVKYAKILNSLLSPVLFLYVIICSLMICASAIQLTTEGTTSMQRIWIAEYLMALIAQLFLYCWHGNEVLFMSEKVDDGIYTSTWYLQNNVLRRNVLLLRGQLRKRIIFTAGPFTTLTIAAFVGILKGSYSYYTILNKRDD*

>HrhoOR7

KMLTKTSVKKEFLCDMAFLTTIGSKIFLYPFEGRTGYKIIGYTTVCCLIYITIAQLFLTLFVTGFHDLIDISNIAPNIGVCILTVIKYTKIYTHRKLYHAIITHYQKYMWSIIQMNDIKNLKTIVKYKSVSYFINKILYYYSLPLIIIIVSLPLIVMFYNKKFNDNEFEYLYPFDGWYPFDKVIWFYFIYAWESFMTALVIYTFAFSDMINMSCVAYMCMELSVLSNLLTNLITNEDIEDLKKFKNIFKIHQRIRRSLKIIIKRHQFLNKLAQGLDLALEDVSLINYIFGSVFICLTAFTFTFIDDLYKKIRYFFFFISLNFVILNQSIIGQILSDHSVKLTNAIYSSNWIYADRTTKTTLLYLMMKTQIPFTLTAKGYISMNLNTMTQVTSTSYQYYNLLRCIYHS*

>HrhoOR8

YWPSSNDNSISGDAFRVTTTIVLCILMVTMLSIDCSTMIYLIMYKYKFITLRNFFENLREEFDKNVKENEIVATNLLTDGVIKGIVMHEELLSLSQDIDKAFGTVMACQVCQSSGSAVSLLLQIALADHLTLAGGMKIIFFVAALFFLLALFLCNAGEITYQASLMSDAIFYCGWHASLPQPPQHRDLRRLVSFACARAQRPLVMKAFKMLELTYATFILVLRSTYSVFALFYAQNK*

>HrhoOR9

MVVFAGFTVILFVSITYSIFYTCSIFTRMHFTILKSDFEGIINIKKPFDVQKFNKNFVSLFKRHRLLIQIVDLLELVYNKAILANFISGSILICLCGFNAMEMNNKVAVVPYAFFLMMCLMQMFFLCLCGDMISRAVT*

>HrhoOR10

MLHDRRPLNYFGLHFWLLRFLGIGWWHDPFSEDKRNFPSWYLYYSILMQIVWVAGFVGLETIDPFVGDRDLTQFMFSLAFVVTHDLTIFKLFIFYIKNKDIKDVVRTLEVDLYDYYQQDEKIFATIKKTKILTGAFLFFGWIIIGNTNVHGAIVDIQWKAEVAMLNNSSTKPPRTLPLPIFIPWSYQNDTSYIFTFIFETIGLFWTGHIVMTIDSFIGTLILHMSNQFIMLQDAYRSAYDRTVNRMLQKHNFNDNIDAEIEINKHFLEADKKELIVRKIYTAEHFNSELEKTLKSCYKQHQILIECVQKFATTYSYGFMIQLLSSVTAICALMVQISHDASSLTSSRLVTSLAFFVVMIIQLAIQCFTGNELTYQAGLVSEAVMECKWEHMPVRLGRMLVLCCARAQRPLRLTAAGFTHINIDCFLSIMKAAYSYYAVLSQKQYSN*

>HrhoOR11

MLACAMDVLEKFTNDYKNTFIRYIKMINFIGLDFYLETGESLFKSRKRYYLFLICFIIFFLCQITFIFRSHKTDAKFLDIANAVPCLVLVIQDFVKLLAITTKRKEIKNVIFKINDEWPKEKNYGERSNIIENWTKRNKSFQNVYYGISLFCLCIYELIPLVATLYNRVMGLDTEYFFPFELYYPYKVDSFFVYLVTYFCQAFASSSLHACIYIASDLLITSLLSDVTALFALLQYDLENVTSQLKQTSINQDEEQYNCAVKNIVNRHQRLLGIIKELNGIYGVVIFIFITSSSIICCFFCFLTVVQNGLQSVKNLLAGGAMLGAILVVAFPGQQHYDMSFGVAYAAYCSLWYERNEKFKNLILILIVRSQRASCLSALGFSDVTLATFSKVSF*

>HrhoOR12

MFFLTSAITTCTLWAMIPLFDDAGSRSFPFKIWMPVDPQKSPHYEVGYVYQMITIYISACLFIGVDSTTLSMIMFGCAQIEIIMEKVRQLQPLSNIKLKPYKRKQQIEEKNALFIECIKHHQEVVKYIEKVEDTYHANIFFQLSGTVAIVCIVGLRISIVDKSSVQFYSMVNYMVTMLSQLFLYCWCGNELTIRSQDLREVIYQTPWYDFDRKFCRLLWVAMERMKRPIIFKAGHYIALSRPTFVSILRSSYSYFAVLNQANK*

>HrhoOR13

MASVKSHWLFAPSKDFFEFNLKYLTYLGLWPKEDWSKPQRFLFEIYSMTLSVFEGVFLILTSIGTYNCKNDITALLTNLDKILVVYNFVMKALIFFIKRKQIKILIDEIKHSKDEITMSRNRMMSVHVVVISVLVISIVSAFSLLATYKQEMTIEAWMAFDPLIDKKHLILASLILAVLFVPCACRAMAIQGIVCSILMYLCDQLIELQIRIRALDYRPETHKQMRVDFNEVIKKHVRLIGRFFRYSNTLRAIFKEYFLFQNLAVTVELCFNAMMVTMVGFKEKTLLLTFFAYLSVALLNSYIYCFLADELIVQSQGIALAAYESQWTTWPVELQKDILIIILVAQRPLTLSAGGMATMSIQTFGQTLYNGYSIFAVLSDVVD*

>HrhoOR14

HKVLLKRQHLTMPKNDLTFANNFKLTTFALKVTRCHPDIIRNKFWIFQMLLIIIMNTTVAWILLNSVVFHDIKSGDFAEVSKNVAMFIICVTISFKLYIIVRECKFLIETMNNMNQDYRDSPENERNIIIKYTKRGAAVSKFWLAAASATSCIYPAKAAVAMVHAYLNGEFHLVPMFDVAFPENLNEKKHNLEISLPFFTLCLTFALYAMTVYVCFDPLAPIFMLHVCGQIEIINTKIAQVFCNKDLTRENFKTIVIKLQELYKFIRCIEKKFTIVYEFVMKTTTILLPLSAFQIVQSVQRREVNLEFISFFSAAMLHFWLPCYYSNQLMEKGEELRSAIYSCKWECSNDLLARKMVLLMLVRATNPLVISSVFYTIKLETFTQMCRDAYAIFSIMNAAWS*

>HrhoOR15

MDKPMLVQFCGSTGIVCAVVYKMTGIPFNTTFIYLALYLGCLLLELYIYCYYGTLLMNESLLVNDSIYLSNWTSLSPRFRRIILIAMTRWSRPMTPTAAGLVSISLKTFVSVLRLSYSIYTIIKS*

>HrhoOR16

MNDPIKSKYILRKITTFAYISGIPYLWYEELGWPKQLTNFHDKITNILTVLLCIFMSLEILALFTQKNMNNQQSSDALKYAISQPIFFMNFFSFVYYKEEVRILFYNLTNSMSTYHRDEEVERKLVKKIKFYVSAFISVAIAASMSAGLDGLWRVLEKDDTFTTVITAWPDVDDRSLAAGIGRIIAFIMWCIHTVRFVGGVTIVVALTVCVCHQYKYLQSYFYSLDKIFEINCSQSVKEEEYEKGLLIGVKLHNNIIRYTQDLCNVCNIAYGGQIVVNVTVLVILMVQMQNGNRQFMLVLTCAIMVSALLVLNGFYMWNLGDITVEASEVCTAMYMSGWENCTRRSSVRVRKILMVAMTQAQKEVTIKTLMILQVSYASYVSIVKFSYSVFSLIY*

>HrhoOR17

SLFDHKGSTRFTYTTLSVFVPRTNVRMESLGRIDCFKFNINFWKFLGVWPETKSCYYAFYSKIFTFAFTFLYVILPTVNLAYIPPVMDIFVEEMMFYFTEVIGLFKVLTMLLTHEKIVNILKVLQSDLFHPESSESVEIINKAKIFIVKYWKFIATVSVTSNLTHVLSPLIVHLLLSVELQLPLCNYFFLPKNIKDTFIYPLYLYQCFGIHMQMWYNVNFDSFILGLMILVIAQLEILDLKLRTITDRCAKLYIKRKQNDTEVLTKLNQALQHYNELGRFCNLIQDVFSIALFMQFGIASCIICVCLFRFTLPAPWQYYIFLGTYILVMIFQILIPCWFGTRIIEKSQLLKFSVYSCDWTCQSRIFRSSLKIFSERANRPITLIAGKMFALSLGTFTSIMNSAYSFFTLLRHMQTRDDMD*

>HrhoOR18

MAHVNNEDMYLNRAKFVMKILGVWMPLENETRFKKIYRTAMMFLQYIFLIFQTIFIFQVWGDLEAVSEASYLLFTQACLCFKITVFQIKIPMLRDLLKQMNADIFRPQTLEHINILILQATRIKRFLLAFMVSSQITCGMWALKPLFDNAGKREFPFDMWMPVSSEYSPQYELGYGFQLITICMSAYMYFGVDSVALSMVIFGCAQIDIVKAKILSIKSISNSVGTTKEHTRRIRKENYNKIIECITQHQAVLKFIQMTENTYHAYLFFQLTGSVGLICMSALRLVVVKFPSIHFFSIVFYVSVMISQLFICCWCGHELTATSEELFATLYQCLWYEQDFKFKRDLRFMMMCVRRPVVLKVGNYITLSRQTFVSILRMSYSYFAVLNSTEK*

>HrhoOR19

LDRRHDAMPLLKKIKTYFDKEGFDYSKNYINPYEFHSTFYFFMKYFKVIDDEPAPKWANVVQVLIGAVGFTNLCLSILMCAINSVKPFTLPKFIEGGTYIIVVFYGISIHLCSIFNKSGYHCLLRMLRQDFDFICTRGQTYRKKFFENHLIIWKLSIVSIIFTQSIAIGMIAFSIILLSYYMATHEPGDGTSRPLLIPFWIFNLDLNKSPIYEILFNYSHLAQLCYGFTYVFLVQTQIVWIKHIETKADIVIWLLNDLFDNLTYPNTEEEKKVCDTEIKNRMCFIIRQHQSVYTLLESYAAVYRKLLMFEQKLCGPVVCFASYCIVLQLEAGEFNGVLLLLCIGALTLTYIPCYLCTTLSEKIMSVSDECMNIPFWNANPKIIRPYLVLMIRRSLRPLPLKVPGFQPHTLQTFSKSMVSAYSLFNMLRQANVQ*

>HrhoOR20

LLDNFNKDFIFICSLGTSYRTSFLNSQLIVWKLFIIWITFCITISCAFVLNTVVYLMYQTLFMTITEDTVRPLIFPLWLPTGDDPYRSPNYEMFFVFEILGTALIPTAFAVYTYNLFHLLLHVYNLMDVMIKAISELFLGLNPDVADLPARNPERQITQAILKTKIKQIVKWHKSVYKYMSTISSIYGPVLVYQVMFSAIAICVMAIQIANALDKGKIDFLFCMLESAALLQLWITCYIGTMIRNKAFAVGDACYNSGWENSKFGCWLRSDIVLIIMRSQIPVSIKFPLLPQIELETFSSITSTSYSYFNMLRRAT*

>HrhoOR21

MKRLKHKHIITSINSIRFLFELNGLKLEDTNSSDNLKYRIIYAFNFTWLNFDGIGELIWFCDGIWSSEHFVKLAAMFPCVIMCFLSNIKMILHNLNEKKVADLINSFKELEDDEFINDTDADDRSKEKIYEEEMKYLEKIVKIVKVLNVVTVIAFGIAPFLLMGAHYLETKEFMPVLPFYVKYYLFDPYNMKYYGLLYLHQFWSMCICLIGILGVDMLFCTMCVFIKIHFKLLEYDFERFIPIHTTPHGCLRENETITRRFKWLVKKHQKVISCSNLLNRIHSNEFMMNFFTSSFLICLSAFIITVVEEMRFRISFLSFLVTSLQQLFLLCFFGDMVMTCSINLSGSIYSSLWHSVKCNIGKQLSYALQRSQKPCKITAGGFIDVNLIVFTQIIGKTWTLFALLRTIFNP*

>HrhoOR22

MLNDEGSFDVNEKFKPFHETYKIFTYIMTMGLIYPNPKTEKIRLKLILFAILFVSPLLFLIGYDVYKCCLRHDIVNIIRHSTVAGPIVFILLKIMSFYYNRDLVKELIDEINRDHVRYNKLPTKYQDIVEKSLRYHKTTEKRWVACVSISSFLFVIMATVFTIYSQIFDAEPMKYMIHEIDAPTIESIIGWPYYEIMFVYESYVSIYFVLNFSGFDGFFGVVINHACLKIKIFCNAFSDALKESNEDEIMRLIHEIIRDQCKMFSFVNTILAVFSSWFVCILIVALALICNCMYLVIQGHGFDIRYIVFTIATIIHIFMPCWYASKLKSMSQESSTMAYFSGWEDVPIPRVRRTLMFFMARGQVPLQIEALNIIKFDMELFVSIMRTSYTMLTLLQSSS*

>HrhoOR23

MKNSECLSASIAILKVTGVWWSDSMVYKIIGSLIQLFLYVFTVLAEIAYVLMVLGDTERTVDAAVLLLSHLVQGVKVATVWFRQRRIKGLIKLIDGPNFEKTDLMKAKMIESFGALLKLSGHLFLSTAAVTALFWVIVPMLKSEITLPLKTAYPFDINDPGSFTLMYGYTTISVVLVGVGDAAENYLLAALLILPTIHLEILCQELQELDHGDDIYERTVSCIKYHQHIIEYANEVASVFGIIMFCQFVTSSVIICMTLFKITITTEPIEMITMVFYLVCVCLELFLYCYAGDLLMNKSLLVSEASFPGKWLKDTRSCRALLMTTVRAQRPLIVKAGGVFTVSLPTAAAIMQTAYSYYAVLQQKTKEHN*

>HrhoOR24

MSSSRRQDDPQNTSSIIISIIMQSIQFIGVWSSVGYKRSIANFATICFILTIGAQVINLVLERNDSEKMMEAFSVFSVCLMGLLKYISLRRNSTAWQYLLSRVSQIENEKMNENNDNLLDYETDDDNKETVPSVKHVYTYNDKAKFISTVLTRFYTLTVFMFVSTPIFEYFWKTYKNHEPMKLPHILPGWTPQDDFHFCAYFITVACEAIAAVYCVRIHVTFDVTFVTLMIFSCGQFNYLWVKSERIGGSGNNCQLSIKRDKRANFRIMQCHKSHIMLVDLVTRLNKLLKIILGVYFTVITLTLCTVAVRLRASDKLGLVKLILLLQYMATNLMQLYLYCRYGDALLNQSSINMGEGPFGAAWWALSPPTRRHLSLLAAGMSRQQYMSSGIYIDNLPAFLQVT*

>HrhoOR25

LTTLNKMTIFIRNVNLSISVSLTVLKLVGFWAPNDIKNNFNIIYYLYAIVTFMILLGIYLIIQVVDMFLIWGDLPLMTGTAFLLFTNLAQAIKILNLVWKRQRVEEIIFNANQLLRDQRTEEGKNIVKKCDRETTLQQLLYFCLTTITVAGWAGSAEKNKLPLRAWYPYDTSKSPAYEITYVHQVGALFVAAYLNVGKDTLVTALIAQCRCRLKLVGLALRNLNEELKADDKHIFNAEQQFVVRRRLNRCVVQHQSALEATVQLQDCFSVPTFAQFTVSMVIICVTAFQLASQTGNLVRVFSMGTYLLNMTFQVFLYCYQGNQLSEESMEIAGAAYESPWYTFSSSTRRSILVLMSRSRRPARLKAGGFTTLSLASYMAIIKASYSFFTVLQQVDENK*

>HrhoOR26

MDDINSDKIPNYTHYIILPLKLVGCWDWYKNPEKEYQIIINNGYYALVLFVLMNLQWSLTVNLYTEWTTIMDNLEKLADSLPLLVSLAIIIHLASNKKKMYELVDFMNNNFKYHSARGLTNMTMRESYTTAKKFGYVYTACTLFSITVYVCMPLLSYAWTKQPLQYWVYVDVSSVSDFVIVFIRQCVGQIFVGLAVGQLGVFFASNAILICGQLDLLCCSLRNARYTALLQNDVHHRDIVADHGDIVNDEKHSYVYNKAVLQDSDYHYDEKMKPTLSRTDFDIYDAEFDVATITAYRECAKMSQVILQYKRDFELLVSPLLVLRVVQVTLYLCTLLYAASLKFDMVTVEYLAAVALDIYIYCYYGNQIIIQADRVTCAAYQSAWPAAGARARGLLLRVLAAHCPRRPVAVRAGGFLTMDLHTFVVIIKTSFSYYTLLVNVNEK*

>HrhoOR27

MYKWLALIPLFNGAICICIVLVVISKEIDWHFVTHILPLFGEIFVYSYFGEQIKTKAKNIELALLSFDWCNMNKEDKINYIIVFTYMQKQFGIEVASNKDLCMVTLTAVLKLTYQAYTVVQSIDF*

>HrhoOR28

CVFIKIHFKLLQNEFERFIPTQTKSRCYLTENETIKKRFKRLVKKHKKVISCAKLLDKIHSNELMLNFVTSSFLLCFSAFTMSIAEDVRFRISFLTFLLAGLEQLYLLCYFGDMVMT

>HrhoOR29

ETLQTFHSILSFAGIPIYAKTNWDSKIRLTHQIFNVFIGFLTFIFTTVFVIINYSDLLLCIQGACIWTTGIIMFISLGVCLIFRRKFRMFLTEMGFKDTMLEMPLVAHVMSLELEDGQKLKELKLKVTESQERLLKLTRDLLKLYVASVWLCATLYICSPIYFMITGEEKSPRLLAFDMWFPWSFDNLNVYVASFVFHAYAGYLCCIAYPGLQLTISLLVGQIVRQLKITSFIMFHLEEIATELSKGRLGNKQMYCTNILTQCVDHYIKMKRFSNNLNVICQPFYLALILVATMLVCVCSVKIAISDKLSLDTMKYYVHESCFILVVYMFCLLGQQVDNECENLERAVTEKWYKFNKTHKINVKIFKMAVNQRMPIYIFGSMKLSLPTFTWFIRNGMSFFTLVMSVLED*

>HrhoOR30

MELDFERIYKLSVNLLKFNRFYPFYKIDMKWIVQVLFLYSISLLIFLALMQSSVYYIKINELSDVCDNGVFSLAFLGLTFMYGTVIWHKNDLIYLIESVQKDYDESKDLSQTEINFILDYIEKGKRVVHLWAFVSIFNVFFIPSRILVVMVSEGKYNLVDVLDSFHPNILEKSTSDIWIFFLELLIRLYYSIYANIMYVGFSPLGPIFMAHACGQLEIVMTRIKSIFTERNYDEREAKIKLIDVVQRMQRIYRFVDSINNTCELYYQITLNSTSLMLPLIVYMVIKDFQMDKVFQYITFIFGSFLMTFIPCSYSTLLLAKGDEMRESIYMSGWERHLDRDARATIIIVLTRASRPISIHTLFKTINLDAFTDVNIDFFINQSATIFIPDHRPLATPS*

>HrhoOR31

MKIMNNNMNGKLAFLLPFLPLSDSETWDKLDPKLYHGVHIYWLKIYGLWYYSFSPKTFKFWLQLAYTAIVLWLVCFLPGIGEIVYLLKRRDNIGDVAGGLYLFLSEMYTYFKLAVFWLNKQKITRLLQYLYCDEFKPKELEHKDIILKSIKRARFVMSAYSTMCVCAVSVGIVMPLTENFDVLPTNVEYDQFDVYKSPAYGILYSHHIYYKPATCIIDGVMDTILAAFVASAIGQIEILSFNLRNFDKLAYRLRTRAIIAKENLYPDQYYIQVTMKECIKHHNSIIRYVSMIEEAFSLASALQFMLSVMVLCLVGIQFLSIEDPRSHLMQIVWMAIYLWCMLVEVFILCWFGDELIWKSQSVRQAAFEGPWLNVDVKTAKYIIIFLERSKRPLRVTAGKIFTLSLDTYTILINWAYKAFAVMSNMKK*

>HrhoOR32

MENKIKPLDAFKMLFRTLTFTAYFMPVPDIENPVKKKWHRYYRIATVIILLIYDLQHITFVILVFGDVDRMIEGLSVLLTILNVTYKLITVNLNEKRFNKLHNVLEDDIFSAKCPKHEELMTKNKEELDGISKTINRTVTVIAVCWLLTPFLKKLSDEEVILPAYFPFPTDDWISFSCASIWITFVIIWVGYGHMTLNILIVGYYSQVKVQLSIVRYSLEHLADDDEGIPHEVFTCRNHGYKDNQSKLYQEKLVALIKRYDKAVWFSKEMESIMNKALLVQFSGSTGIVCTVVYKMTGVSNQYIIKNL*

>HrhoOR33

MVVALFCFMPITLMAVDYYKNGKYKVNFPFLVKYFFDPFTEIWPYVYFHQVVSTFIVWVNVYGPDTFFYAFCVYVQMHFRILSQRLRKLFYKPNLLVEDKEKLIKLLKRHQELIQLVKDFETLYTSSNLWNMVISSILICLSAFNATTNPDAKAVLTFICFLFMSLSQISILCFFGDMIVNSSALVAEAAYSCGWYNVDADVKKSLLIVIMRAHTPCKLTAANFAVLNLRAFAMIISKSWSYFALLKTLYK*

>HrhoOR34

KEVAEMISRFLETFEDPKRPLLAPNYWILNKVGLLLPDSKLGKFFFIIIHEIATLFVLTQYIELYVIRSDFDLVLTNLRISMLSTVCIVKSNTLLLNQSKWKQIIDYVTTADIFERENRVPDKKNMLDSCTKYCRSVTYFYWVLVFSTVMTTTSTPLVRVMSSLSYRDELRNGTELFPHIFSSWMPFDKYHSPGLWITVGWHIIICVYGATILGAYDTTVIVMMEFFGIKLELLRARCQMMFGKDESGISDDKATKIIQQLHTIHVKLLEHSRLLNSILSPVMFVYVIICAFMLCTSAYQLTTATSTTQKVFMAEYLIFGIAQLFMFCWHSNKFLVKSQEGMFGPYESNWWAAGIKQKKLILMLTEQLKLVHIFSAGPFTNLTVSTFLGILKGAYSYYTLLRK*

>HrhoOR35

MMCNVSTDSFMAGIVTILIAQTKVLNYKLQNLKVRNENFVEETHVQHKIFTVILKKYLKHYGLIMECYIKIQEILSLAMFVQFTMASAIICVTLCGLYLGPTMETLIFLVTYLIIIILQIFIPSWLGTQFSHECNKLAVAAYNCDWISQSEDFKKSLNLFILKANATVILKGLKIFPLSLETFVSIMKTAYSFFALVQNVQAR*

>HrhoOR36

MTLQYDMAKIKKKETETKFKSFHETYSMCAFALAIGLMYPNSENRRKRLIYMIIFTTANVPQLYWLTINTLQTLKDSDFYNFSRHITISVVVLLFLFKTVYAIIMCDMYKKLLNQITDDMNKGNELDDSYKAIYKQYIKEAKFGQICWVFIPIAMSFQFPAYAAICTIYESIISDVGPKCMIHNLDLSFMGDQYNISPYFEIMFVYNAIQTIALVPNFTGFDGSFCIVTSHLRLNLKLLSHKLKRIFEDSKSNLELRKNIKTCVIEHQEILRFYDAIQVFYAPWLMTVFLLTSVLISFNLYRMHLDQKIDLKYSFFALSGVIHMLAPCYFSSKLIEAGEEVAIEMYSVKWQRWNDNKVTKVLIFMIARAQKEFVLVGAGIILFNMNLFLSLMRTSYSVFTLLCTR*

>HrhoOR37

MFDSIRNYIAYFKNRIKDNNFDSLLWIVNGAPSIVGFNLRKDKIWAPFFVIHMSLLTYVYGVGNVMYQVKYAKNTGDFIESYVNITIMILAAVSGYWFIVYRPPLRVILNLAEENDRLSKTSPIVKKKREKLLATIKIIVFIFYGCNLTNATFVYLPHRVDILSHYAMQPCVGLEPLTSSPNREICLTILCAQELSIMTVVLNYQALWLVLVAHTAVMYQVLSEEMLIVNTDEEILEDKLLSFIHRHNVILDITHRLKEIYSMPIGMNLGVNAICMCLFFFMPLSDWFKFMPILVYCFVVFFLNCFLCQRLINASEEFERAVYGCGWENFDVIKKKWVYIMLMNSQKPAQLLAADIIPVNIATFATTMQSMYKFITVFKL*

>HrhoOR38

MENTNSPERSVIQYVRSSRAFRQFKNPPQPHMCIQDTLKDTTEKMFINVLGWQKIANPKQYSDPIPLYGGMQVPQGCGPNSNKPPLLVFAVMVNPDILKANGKNATNPTDRDALVNLLCDFVEAMNPGLALARKPVILRDRDLAGELKDVWLAVQKKRDKEKEGNQEVMYKVYDIDGVGNDEVNDDERQANLRYNQGDGGSPTKVQNNRKNAVKSSKQILMNAGQKSEFDLGMNNCQLNQNHSNRDNKCATDTTYCTPVYEQIVSYSENHNQINDIQEKFTKHDANASTSFTAEWDALHGKPSDGWEDFSKRNINSIKSNEADTRQRCSKTENGKLVNKTQYNFFPVFNKTEADSSIDNVSNEQNLRIEEKAKIILDPMQKLVLHSTDNKICDNNSSALSSLSS*

>HrhoOR39

MSSLSSHRAIKLFRRMCCYAYVTGLPNFWYEKPNWSIHVGKFHDFISHVTDVINCVFYLGQFFSFFTQKNLNERQETDQIIFTTINPCIYWAPIAMNYYKEQVRDLIRNLVLVLPSVYNDREVERKMVKKSCLYVSLLLSTANATLLLYGIDSFIKVLKGAVFTTVITAWPSVEDRSRIAGIGRAAVFIIWTQFMFRSCGAISLIISLTINTSHQYIQLQSYFRNLSNIFQENLIQEEMETKYEEHLKIGIQQHIKILSYTKKLKQACILVYGGQIFTNMVILVMTMMVMMGDDLSLTKLMTFMTLAISSTVTNGFYMCTIGDITVEVQRYGSQ*

>HrhoOR40

MTVWPDLKDESFKAGAFRIATYIAWWILMFRLSGAIVLVMSLMTYTSYQFKQLQSYFITLANIFQQDLSQLEKERKYEEALKIGIKLHVDVISVTRKLVTTCNVSYGGEIIVNVIVIATIMIRLANEDRNLTNILASVQIALTVLGITGFYMWTLGDITLEAD

>HrhoOR41

MTGAKNVKNRVEAWNVNKRQNRINCANANPVHSEATFQEALRATLIIGQIFSLIPVDRVSSKNTANVKFSWTSWKCFYLVLSLGGQVFMTAMCLNKLFDRDTSLKATTSVIFYTMTTVTMLMFFQIARNWPCLVQQIANTEQMDPNFDRNLTFKCNITCAIVLTLALTEHILSLLSAFAGALTCYPNMSLYEGFVKYFYPWVFNFLPYTVPLGILTQFFHFQSTFIWNFSDLFVINMSYYLTSRLQQINNKLLSVQGKYLPESFWRVTREDYSRATQLVRRVDDVISGIVFISFANNLFFICLQLFNTLEDGIQRTEACRRRLGRTGPLGGHEAETYFLFSLGYLIARSVAVSLIASQINMAASVPAPVLYDVPSPVYCIEVQRFVDQVNGGSVALSGLQFFNVTRGLLLSVAGTIVTYELVMFQFNSSGNSNSTEVAANATIS*

>HrhoOR42

MFIDGELKDGSEQLEQLNKKKKTKSNAKGTNQQEAPKSMLVSLFVPFENSMPESVEYMECDGSIHFSGVVSSSVFMYPKATVNEAIASVKQDIVRSLASRFTMHCDALIDDNLLPEEKVCFNEPPRRVLVPVGALHLCDYLFPGEAPAEALLSVRELLDLHITEADVVCDIETPADTSEFDALDRDTSSEELLASPQEASQFMYITGICFAMLVLFISIIIHYYDGITKFISGMFSKAT*

>HrhoOR43

MPRSQSIRNTEWFNVTFILMAAVGIWEPPCSENKIIVKYLYLIYRLIFLSLFAFAIISMQLFLFFLVLGDMDALIEASVLFFCNIIHGIKMITIIIQRKRIKSLLTIVDDDVDNHKVYENLGKRAGFMSNMFYLNVAATGILWSIYPMTKSELKLPYSCPLISKDSYWFTYFYVY

**IRs**

>HrhoIR8a

MDFFVLFFILMIVNVIFVTSEISLRFVFILEHHDYELAEQIGNALKTVEESTPGVHLSDAVIFLNREEDGESYRKLCSSVSTGVSMIINLSWAPWPAAEDMASSSGVPIIHTALGSQQLIKALDDYLESRNASDAAYILESEKDVDKTLYELLGRSNVRVWVHAGLTRDSANVLKSMRPEPSFHVVVGNKGFIMDTYRRAVKEKLVRRKYRWNLVFTDYSGADMDWSQVTLPAMVLYINPDECCKLMKQEKCTCPPDFQRTQSMLSYLIEYIVTSYGKLEDQQFTTKLDCNAIEMGDMNVTKEKLLDFFNQDSTNNDSLFYWNIERSGLFLRSRFVLSYSDGSERLELVAKWSADEEYKLLPGVTLEPLRMFFRIGTSAAIPWTLHKMDPNGQPMVTDEGDPVYEGYCIDLIQKLSEVMEFDYEIVTPKSGSFGRKLPNGTWDGLIGDLARGETEIAVAALTMTAEREEVIDFVAPYFDQTGIIIVIRKPIRKTSLFKFMTVLRTEVWLSIIAALILTGFMLWLLDKYSPYSARNNPDAYPYPCREFTLKESFWFALTSFTPQGGGEAPKALSGRTLVAAYWLFVVLMLATFTANLAAFLTVERMQTPVSSLEQLARQSRINYTVVEGSSIHQYFINMKFAEDTLYRVWKEITLNATSDQAQYRVWDYPIREQYGHILLAINASGPVPDARTGFRQVEEHLDADFAFIHDSAEIKYEVTKNCNLTEVGEVFAEQPYAIAVQQGSRLQEDLTRALLDLQKERFFEQLNSKYWNESLRQSCPDADESEGITLESLGGVFIATLFGLGLSMITLAWEVFYYKRKQKTNVHDTTTSEKPRPAFVKKGKLRRRKKTVTIGDSFKPAVDVSHITVYPKGYVP*

>HrhoIR21a

MFWYVFGTFTNCFTFVGKNSWGKTTKNTTRLLIGWYWVFTIIITSCYTGSIIAFVTLPIFPATVDSIKQLLSGFYRIGTLDRGGWERWFINSSDPDTNKLFKKIELVSTIQAGIKNTTKAFFFPYAFLGSQAELEYIVQANYTKTRSKRAVLHISNECFVPFGVALSFPNNSIYTSRFSNDIRRVLQSGILKKIVSDVKWEMQRSSSGKLLSVGSAILKSTSIEEKGLTLEDTQGMFLLLGAGFLIAAGALLSEWMGGISRRCCVIKKKATSANSSRNLMTPFNTENEVKDDTDGFDLERKSDLDSNNSSADSRKTLDGHIIKLTENSITVHENFNSNDWNCRRSSSVDIDQEVKEIFEKDISRRRKAFDDRSSLQDGRLTTASKGKFGEYIP*

>HrhoIR76b

MVANGRAVFRSFTSDRDFLPTVKAGAVLVKEQTAVDHLMYFDYLTKVREGVVEEERCTYVVAPNAFMKRTRAFAFPMNTNLTTLFDPILTYLLQSGIVDFLEHRDLPTTKICPLDLQSKDRRLRNSDLMMTYMIMGVGLASAIAVFIIEMILKRYAVKHKLKPLKKFNSKTFTFKDDSMPPPYDSLFGKNSKYRGSKRTVVNGREYWETKMKDGTTRLIPLRTPSALLYQ*

>HrhoIR41a

MLTSKMLSIPIEILLKIILQKYFINSYCITVVSEDSIQLKTSIPFIYAIPNDNFVDLLLNSSDIGCSDYIVNMKNPQEFMKAFEKVTHLGLLRKSDRKILILTHSKSYNAQDKDAILKVLSMNETRFVANILLVIQADVNEKCYIYDLITHQYVGKDDVRKPIYLNQWNSCTGFTNNVSLFPHYNMSDLYGKTLKLACFNYEPYSLLDLDTSVDPLGRDGMEVRVMDEFCRWVNCTIELVRDDNQWGEIYSYENLTGVGVIGNVVKDEADVGISALYSWYEEYIALDFSTPLVRTAVTCIAPAARVLASWELPLLPFSLHMWLGLGFTFFYASMALMIAKGFNTDKMFLTTFGMMITQVRFLNSCKKSGGYHSPH*

>HrhoIR60a

MTMVVFLFISLLINEVSLINPNGPTAVEDFTNCITSIVKVSFKNPGLLVFVDTFFIAEAVGRIKGNVLKQIHLNKKFSVRVVRPKNEYPVCVNLNEFNTGVVHQNQVDVIPLADYFVIIVDSYSEFTHAASRLIRLRNWNPHGKFLILLYSFDNIYYLKQIEYIFTCLFRYNVLNVVVLVPHIRNIRATIIYTWEPFEPPKYCGYYNETAENRIKVADFCEKGHLKNNTTLFENVVPIDMMSCVVNILAIEKQPFIGKDDNVQEANIERFLINEVLSTINMKTNYIITNKSRGERFYNEWNGALKKIVSKKFNVLLGGIFPDFDVHEDFQCSNTYLEDSYTWVVPRAHPRPPWVALTIIFHKTVWLSVLIGFTISALSWKFLSTVSGDSTYYTSIDHCLLSTWLCILGLTTHIRPKKESLRIFFVFFNIYCIIFITAYQTKLFDVLTNPSFEYQIANVEELIDSGLKFGGFEELHDLFYNSSDPFDNLIGSQWVIVENMSNAMVDVVVHRNFSVLCSRLELTYLSATMPQLSDSIGHHKYYAFKTNVFTVPIELIAMRGYALVEKFSEILEAFKQSGIVSGIRRHYVTFAERKRASIILKLQSQQNDVRALTIQHLQGGFLALVLGYVGGIIVFIVELIIKCNLVQKVLYQK*

>HrhoIR75q.2

MKAVNLLALFIITVISCLAEADLVSIIGDLIRVMNKPSSVIATLCWPQYKQLKLYYFLHRENISHLTTIQFLKLGHEPKNYWPSQNILFLLDLNCTNVTNHLKLSNDKNLFRSPYRWFLIGIDETLNNSNSNINIKRQFKPFDIFPDSEVMIILFHYNSRNDSRYEVIDIYKTCKNSEDMKTKLYGNWDATNRFQKSLNFYKPTALQRLDLGGCEIAISYVLTNNNSIHHLYDQMDDHVDTITKVNFPTTNHLLEFLNATRKYSFTDTWGYRLNGTWNGMSGYLFRGEVEIGGSPMFVTSERISFVEYISNPTPTSSKFVFQQPKLSYGNNIFLLSFRETVWYCATALVILIFLTLFAVTFWEWKKINDGNMLDNRDPGILRPNVTDILILIVGALCQQGSPVQLKGSLGRIVLLVLFLALMFLYTSYSANIVALLQSSSSQIKTLEDLLHSRMKFGVDDTVYSRYYFSIATEPIRKAIYETKVAPRGEKPRFMSMEEGIKNMQKGLFAFHMEVGVGYKFVGKYFLEGEKCGLKEIPYLQVQDPWLAVRKNTPYKEMFKIG*

>HrhoIR68a

MNFKSKLYKPVNADINKWGQKQSNGSFSGLIGEMVRGKADVGLGNLQYTPYHLDLMDLSIPYTSQCWTFLTPEALSDNSWKTLILPFKLYMWITVLLVLLVTGSIFYGLAKFYLNLLEFKDHSSIYDKHLDKQIVYDGAKPVGLYLFGEIINSILYTYGMLLVVSLPKLPTGWSIRLLTGWYWLYCILLVVSYRASMTAILANPAPRVTLDTLKELVESKIACGGWGTQTKIFFEESLDEIGEKIGEKFQIVNDPDEAAAKVAQGVFAYYENKHFLKYLSVKRKNSLIDTTPQDNSTANSTAVTAKKKDERNLHIMSDCVINIPISLGFQKNSPLKPLADLYLSRIVEVGLVEKWLNDAMHPIKSLDSQEEEIKALMNLKKLYGAFIALAIGYFLSTISLIGEFIHWYLIVKKDPNFDKYALDVYYANKNKRQ*

>HrhoIR75p

MKLYNLLYILLCIPTDVACSRNVDDMLLSYITMENQPTSLLAPELCWPLHHKTSFTRLLNGVGVNVAYTMRPSRKEQYLHHITILADFSCSSASDLVLQSDEHGFFMSPYRWIFINLHQQSPNATILDKLNILIDSNVVVVQKVDDTKYVFHEVYKIAKDYQVIKNLRAVWRAVNDTEKRNEQTTSSIHVNLTPGNNSVSVNSKTNGEIEDLFYSTPLSCRRRNLRGHSLTMVNVITDSNETKNHMHDRLFLHHDSISKMSYMVVRICFEMMNASENLLFTNTWGYRDKHGNWQGLIDHLLKKKADLGTLTIFTKERTEHIDYIAMVGSTAVRFVFREPPLAYVSNIFTLPFSGAVWFAILICVLGCALFLYITSKWEATMGSHPLQLDGSWADVLILIIGAVLQQGCTLEPRFTAGRCVTLLLFISLTILYAAYSANIVVLLRAPSPSVRSLQDLLSSPLKLGASDFEYNRYFFRQLNDPTRKSIYDKKIAPKGKKPNFYNMTEGVEKIRQGLFAFHMELNPGYRLIQETYQEDEKCDLVEIDYINEIDPWLPGQKRSPYKDLFKVK*

>HrhoIR40a

MRVAVVTNPRESVFRIYYNQGTPNLLHHLTLVNWWSGRLYRSPVLPPAEKVYKDFRGREFEIPVLHAPPWHFVKYNNDSTVNVTGGRDDKLLSLLAKKLNFRYKYYDPPERSQGSSISGNGTFKGTLGLIWKRKAPFFIGDMTMTWERLQAVEFSFLTLADSGAFLTHAPAKLSETLAIIRPFRWEVWPLVLATLLVTGPALWMVIAAPSLWRRQRRDQLQLFNNCCWFTTSLFLRQSSSKEPSSTHKARLVSVVISLGATYVIGDMYSANLTSLLARPAREQPIGTLQALEEAMRDNGYELVVERHSSSLTILQNGTGVYGRLAKLMKRQQVQRVRNVEVGVRLVLTRKRVAILGGRETLYYDTEKFGSHNFHLSEKLYTRYSAIAMQIGCPYLETFNNVVMTLFEAGILTKMTTDEYRDLPKLSRRSDPVTESDTEGSDAIGESTAASQTQVESTKGLEPVTLRMLRGAFCLLGIGYLLAGVSFCIEIQIHRRRTRTKAPVPETKIKEKQKKFQRILTNIKIRFRRIAIKIYSKIDTALGP*

**GRs**

>HrhoGR2

MPITRSRPGTITFSWKSRATAYAIFFYIASTAVVLVVGYERIMILRSIKKFDDYIYAILFVVFLIPHFWIPFVGWGVAHEVAIYKTNWGKFQVRYYRVTGENLQFPNLKNLIVVISVGCLLLAVCFLLSLCALLDGFLLRHTAAYYHIITMINMNCALWYINCKAIKIASQSLSLCFRKVRKYNNSCVRRRLRDCGPRVWIQL*

>HrhoGR3

MITFANMSARIIIEAMGSETGFIFGVLEFTALSSLMRLGTCAAVMICVVSYCERVYRQRERILTIIDHLFISKMINAETRKSMNELRELVQSRTICFHMANLVVIQYSLLVSVASVVVTYTIILLQSIK*

>HrhoGR4

DVADEIRAMIVETDLKSKSAIIELRNMVQSRPITFTAANFFRIDYALLVSTATACITYTIILAKL*

>HrhoGR7

SNAQATFLWTFNDIMTMMFSIYLIAYFRDMNRLIYSPIPKNIHVWNRLRIFCSELVSLVQLVNPRLKYFILNSIGCSLY

>HrhoGR29

MSLICCFPKIMYILYGILKLYKARASALPIVVLGFGALQWGLMPCLPGAVMEFAYNEVEKIKKTLVHQCQYNKDELLREDIKEFIQYIDSRPYKYRILRMITVDMSLPIGLLKLCTTYFIVIIQFTHLFE*

>HrhoGR64a

MKVNGTYKRYDHQDEILERDEFLDTLNTIFKKSRWFGVAPRRRSLLFIWAIINAVLMMAVEAGAIWKLIRAITGTVFNTAGGHSLVARLSGSIFYANGLLSLALSWRLITSWSSVHSYWIKTELNRSLFLPPDVHIKKRVIFITSLVVTCALGEHILSMISAIGFRCPPSEYMEKYILVSHGFLIHKNEYHIWLAIPIFIVSKTATVLWNFQDLIIILLSTGLTSRYKRLNSYVKNLVEIEQSQEMKKHANEIYIEVQTWRRVREAYIHQANLVRRVSNKLGALILLSSLNNFYFICLQLFLGINKDKGEMINRLYHFLSLSWLMLRASGVALVGADVDEHSRKALKYMKMCSNHNVEIERLKNQMKNDRVVLRGLGFFALDRNMFLKVAAAIMKYELVLVQYDK*

>HrhoGR67

KRRIVEVDEAIKNMDEQIDYVRQVHSVWTIAVAPVVVSLMRVFSIYISIATSDIAVPIEKMGQLIYADIFALLITALHCGHNNLLRERFKIVNVTLRKIKDRKAWFRGALFSRISISDTKHVAQHREKYICDKIKACAKIYDKLMGCVISLNTIYGFAMVQTMSLSLVYIVLYLFYLMEATASGLYNDANRYVNFIFYVSWQILYGVGVIFFNIHYCEETVKEAKITSRIV

>HrhoGR68.1

MFRVISKIIPIYRQLNVTWYSAFKPIYYLTSLLGLFPYTLKHESTNVFKTYLNSYYLNFIYASIIFIILCVFCVLHIQDVIYAGKSYSMTNENLTEINYIFEFVFLLVYCFVAYYCAFTNGKLYINILNRVIATCRRTAVDRYEKNMKLVDKRLKKIVYSYTLLAVTTIIINFTRKGSVWKSVLVLFTFILPQTVQLATLAHYCSLVVMITGLLDNIILCLSYYDQNKRVAHGIWTTKPRAVSILSNLKTCFIKVYNIKEDINRAFQAPILFTAIQCFHCLVSEAGSLYHGVG

**SNMPs**

>HrhoSNMP1

MKLRKHFKIAIGSAIVGVFGVLFGWLIFPTVLKSQLKKEMALSKKTDARKMWEKVPFALDFKVYLFNYTNVDEIHKGGVPIVKEVGPYYFEEWKEKVDIVDNDEDDTVTYKKLDTFYFKKDKSGPGLTGDEIITLPHAFILSLVTIISRDKPAMLNMAGKALNGIFDNPPNMFLKARALDVLFDGININCARTEFAPKAVCTALKKEAGNQLKILENNQFLFSFFGMKNHTVDSHVVKVSRGMKNVMDVAKVLEIDGKPQMDKFRDKCDLFDGTDGTKFPPFMTNQPVASFSTDTCRTFKPWYQKQSSYQGIKTLRYISNIGDYANDPELNCFCDTPDSCPKKGFMDATKCLSAPLYVTLPHLLDCDPEEQKNVKGLSPDVEAHGIAIDFEPITGTPMTARQRVQFNLRLIKTDKIEQCKELPDTIAPLFWIEEGYALDRDFVKLLKHQLFLPKRIVGVVRWLLVSIGILGTFGSLVFHFKDRIIQFAIPSNATSVTKIKPEEENKKQISVIGNTQDATELAKIDM*

>HrhoSNMP2

MMYEKWRKLPMPLNFKIYVFNVTNVEEVNAGANPKLVEIGPYVYKEYRERTDIEVTDNDTVRYMLKKSFVFDGEASGSKTEDDIITVIHYAYVAAIVQVHDTMASLLPILNPALQEFFGNVSSPFLTIKVKDLFFDGIFLNCNGSQQSLGLICSKIEVEKPPTMRQADGGNGFFWSMFGHLNRTITGPYEMARGLTNIQELGHIVSYQGKRVMTEWNDPYCGQINGSDSTIFPPIDENNVPSRLYSFEPDICRSLYISLSEKTTRFNMTAYLYEMDSSALASKSANPDNKCFCDKNWSANHDGCLVMGVLNLMPCQGSPAIVSLPHFYLASEEILSYIAEGIDAVKEKHKSYVYIDPSTGVPLDGLKRLQFNIELRKIPNIKQFENVKTGLFPLLWIEEGAVLPESLLSELRQGHTMIKYVEVFRWVLLAVALIVTAVSGYLVARAKSLVWPHHAPVSFVLQPHGMSEVNKVH*
