# Supplementary material for: Identification and expression profiles of candidate chemosensory receptors in Histia rhodope (Lepidoptera: Zygaenidae)
Source: PeerJ. 2020 Sep 24;8:e10035. doi: 10.7717/peerj.10035 (PMC7520089; doi:10.7717/peerj.10035)
Supplement: Table S3 [file peerj-08-10035-s004.docx]

**Table S3. Nucleotide sequence of chemosensory receptors of *H. rhodope* in the study.**

**ORs**

>HrhoOrco

ATGATGACCAAAGTGAAAACTCAAGGTCTGGTGACGGATCTGATGCCAAATATCAGATTAATGCAAATAGCTGGGCATTTTATGTTTAACTATCATGACGAAAATGCTGGCATGTCATTTCTCCTGCGTAAGATCTACGCTGGCGTGCATGCGTTCCTCTTTGTCATCCAGTACGTATGTATGGGTGTGAACATGGCTATGTACTCCGAGGAAGTGAACGAACTCTCAGCCAACTCAATCACGATGTTGTTCTTCGCTCACAGCCTGATCAAACTGTTGCTCTTTGCGATTAACTCCAAAAGCTTCTACAGGACCCTGGCGATATGGAATCAGAGCAACAGTCACCCGCTGTTCACGGAGTCCGACTCTAGGTATCATCAGATCGCTTTGAAGAAGATGAGGAGACTTTTGTACATTATTTGTGGCGTCACACTTTTCTCAGTGACAAGCTGGGTCACGATCACTTTCTTCGGTGAGTCGGTCCGTTTTATCGTCGATAAAGAAACAAATGAAACTTTAACAGAACCCGCACCAAGGCTTCCCCTGAAGGCATTTTATCCTTTTAACGCTATGGGCGGTCCGATGTACGTCTTCGCTTTCATCTATCAGATATACTGGTTGCTGTATGCTATGTCGATTGCTAACTTAATGGACGTCTTGTTTTGCTCGTGGCTGATCTTCGCCTGCGAACAACTGATGCATTTGAAAGCTATAATGAAGCCTCTGATGGAGTTAAGCGCGACCCTGGATACTTACAGGCCTAACACAGCTGAACTGTTCAGGGCATCGGCGGAAAAATCTGAAAAACTCCCTGATCCTGTCGATCTGGACATTCGTGGTATTTATTCAACGCAGCAAGACTTTGGCATGACGTTGAGAGGGGGCGGAGGAAGACTGCAGACGTTCGGAGAACCGACTCCCAACAACCCCAACGGGTTGACTCAGAAACAGGAGATGTTGGTTCGTTCAGCGATCAAGTACTGGGTGGAACGGCACAAGCATGTCGTTAGATTAGTTACTGCCATAGGTGACGCCTATGGTACAACTTTACTATTCCATATGCTTGTGTCGACTATAACACTGACTCTATTGGCTTATCAGGCGACTAAGATCAACGGTATCAACGTGTATATGTTCAGTACAGTTGGATACCTCCTCTACACTCTCGGACAAGTGTTCCACTTCTGTATATTCGGCAACAGGGTCATTGAAGAGAGTTCGTCAGTGATGGAAGCAGCTTACTCCTGCCAATGGTACGACGGGTCAGAGGAAGCCAAGACGTTCGTTCAGATCGTGTGCCAGCAGTGCCAGAAAGCGATGAGCATCTCCGGCGCTAAGTTTTTCACGGTCTCATTGGATCTTTTTGCTTCGGTGTTGGGCGCCGTGGTCACTTATTTCATGGTGTTAGTACAACTGAAGTAA

>HrhoOR1

ATGAAGCGACCAACAAAACATATTATATCATTAATAAATTCTATAATTTTTTTATTCCCTTTGAACGGTATCAAATTGGAAGAAATAAATTCAATGAATACTTCGATGTATAGATTTTTCTTTGTTTTAAATTTTCTATGCATGAACTTCGATGCTGCTGGTGAAATTATTTGGTTTCTCGATGGATTATGGTCCGGCAAGGAGTTCGTTATGCTAGCACACATCGTTCCCTGTCTGATCATGACTTTCTTGGGCAACATAAAAATGATATTTCATTTCTTAAACGAAAAAGAGGTGGTTAAACTAATTATAAATTTCAAAGAACTAGAAGATGACAAAAATATTGAGTTGGCGAATTCAAATGAACAAAAATCGACGGAGAACATATATGATCGAGAAATAAAACACTTAAACACAATAATCAAATTAGTGAAAGTATTGAATTCTTCAACTATAATAGCGTTTGGTTTAGCTCCGTTTATTTTGATGGGAGCAAAATATTTGAAGACAAAGGAATTTGTGCCGATACTGCCTTTATACGTCAAATATCATTTCTTTGATCCGTACAATATGATATATTACGGTTTGACATATATACATCAGTTTTGGTCAATGTGCATATGCATGTCAAGTGTTACCGGGGTGGACATGCTTTTCTGTACCATGTGTGTATTTATCAAAATACAT

>HrhoOR2

ATGCACAGCTGTAGAATTAAACCTATAGATGCTTTTGAAACTTCATTTCAGTGGCTAAAACTTACTGGATATTTTATCGCTGTGCCCAATATAGAGAATCCAACAAAAGCTTTATTGCATAATATTTACAGAGCTTTTACCGTTTTCATATTAATAGTGTATGAGCTACAACATCTAATATTTATTATACAAGTTTTCGGAAATGTTGAATACATGATCGATGGTCTTGTTGTGTTAATAACTTCATTATACGAAACTATCAAACTGTTCCTGGTGAACATAAACAGCCGTCGTTTTGAATATTTGAACGATATTCTTAATGATGATATGTTTTGTGCGTATTTACCAATAGATGAAGAAATTATGAGAGAAAATAAGGAACAATTGGAAAGGCTTTCTAAAATAATATACAGGACAATTGCAGTGACAGGAGCATTTTGGTTGATTGCGCCATTTCTAAATAAAATGTCCGACGGCCATCCTCTTTTAGCGGCGTATTTTCCATTCGACACAAACGACTGGTTTGGATTCACCTGCGCGAACATTTGGCTAACGACAATAATAGTGTGGGTCGGTTACGGCCACATGTCACTAAATATTCTTTTAATCGGATATTACAGTCAAGTTAAAGTGCAATTAAGGATAATAAGACACCATTTAGAGCATATAGCTGATGATCACGTTAACAGCACCGAAGCTAATAAGTACACAAATTATTTGGACACTGTTTGTTGTGACGTTGAAAAAGAATTTATAGTATTACTTCAAAGATACGAAAAAGTTGTGAGGTTTTATCAAGAAGTCGAGTTGCTGCTAGATAGAGCGATGTTGGTTCAATTTTGTGGATCGACAGGAATCATATGTGCCGTTGTATATAAAATGACGGATATTCCTATTAACACTACATTTCTATATCTCGCATTGTATTTGGCAGGCTTGTTATTGGAGCTATTTATTTATTGCTACTATGGTACATTG

>HrhoOR3

ATGGTTTCCAAGAAAGTATCGTCTTTAGTTAAAGAGCTTGAGGATCCTAATCATCCTTTACTTGGTCCAAATATAAAAGCGTTTTACTTCTTTGGTTTTTTGCAAAGTTACCATACCATTCAGAATATATGTTACAAAGCCTGGTATACAATAGGTTTCTTTTATGTTGTTACTCAGTGGATTGAGCTATGGCTCCTTAAGGGTGATCTTAACAAGGCATTGGAAAATTTGTCAATTTCTACATTGGCCATAATGAGTACTACCAAAGGAGTAACTACAGTCATATGGCAAAAGTATTGGAAAGAACTACTTGAAAATATATCAACAGAAGAAAAAAAACAAATAAATAAAAAAGACAACACTACAATGAAACTAATGAAGAATTACAAGAATTATTCTAGAATTATCACATATTTTTATTGGTTTTTGGTATCAGCAACAGTATCAATGGTGATAACATCGCCGTTTCTAAGATACATGTATTTAAAGTCACGTATTAATGATCAAAATAATAGCACAATATCATATCCAGAAATCGCCAGCTCATGGTATCCGTTCGACAAGACAAACATATTAATCCATAGTATGAAATGTTTTCTAGACGCTATAATGTTGGGCCAAGGTGCGACAGTTATAGCCACTTACGATTCTACCGTCATAGTAATCATGATTTTCTTAAAGGGTCAAATGCGAATTCTCCAAGAGAATTGTAAGAAGATGTTCCAAGAAAAACAAATATCAAATCATGTTGTAATAAACAGAATTAAAAATTGTTACGAACACCACCAATTTCTTGTAAGACAACACAATTTACTTAACTCATTGTTGTCACCGATTATGTTCATGTATGTGCTTCTATGCTCGATAATGATTTGCTGTAGTGTCTATCAACTGACTTTGGAAGAAGCAACAACTTACCAGAGACTTTGGTTCGTGGAGTATACTATTGCAATAGTATTCCAACTGTTCTTGTATTGTTGGCACAGCAATGAAGTTGCAGTTGAGAGTGACCTATTGGATCGCGGACTTTACGAGAGTGACTGGTGGAAGTCGGATATAAATATTCGTCCTATCTTTATATTATTAGCTGGAAAATTAAACAGAATCTTTGTGTTGGAGGCGGGTCCGTTCACAACACTTTCTGTAGCCACCTTCATTAAGATCATGAAGGGCGCCTACAGCTTTTATACGTTATTCACTCAGATGCAGAAATAA

>HrhoOR4

ATGGGGCTGACGAAAATCCAACAAGAAAATAAATACCATAGCATCATAATGTTCGTGTACATCTGCGGCTTGCCTCATTTCTGGTACAAAGATGTCGATTGGTCGCCAAGAAAGAAAAACTTATTGAAATTTGCATCGAAATTTATAAATCACATAGGAAACTTGTTCTTCATTACCGAACTGCTCGCTTATTTCACACAAAAAGAATTGGACGAACAGCAATTTTCATTTTGGTTCGGGTGCGCGTTCACTCACACTATGTGCATCTCGGCTATATTTAGCTTGGTCTACCACAAGAAGAACATCGAGAGTTTAATCACCAGAATGATTGTGACGATACCCAACATCCACCACGACGAAGAAGTCAGCGAGAAAATGATCAAGAAATGTTTTTTGTACGTTTTGACTTCGATCAGTACTTTGAACTTGACCGTACTGTTTCATGGTGTCAAAGCGAGTAAGGAGTATATGAACGGGGGTATCTTTTTACCGGTGATTACATTTTGGCCAAAAACAAGCGACTTGAGTACAGCAGCTACAATAGGTCGTTTTGTAGCCTACATCATGTGGTGGATATGGGTGGCCAGGGTCTCAGGGATCTTGACCACTGCTATTATCCTCACGATCTGCAGCAGCCATTTGTTCAACCATCTCCAGACTTACTTTAAGAAAATGTCTACAATATTCGAGGAAAACTTGACCATTGATCAAAAACAGCAGAAGTATGAGGCGTCAATGAAAGTAGCTTTTAAAATGCATCACGACATTTTAAATCACATAGAAGTGCTCATCGACGTTTGTAACGTAACATACGGAGGTCAAATACTCATGAACGTGTCCATTTTGACCATCATGATGTTCCAGTTAGCGAGTTTGGAACACTTTAGTATCGTGGAAATATTGCCACATATCATGATCATGATTACGGTATTGACGGTGACAGCCTGCTACATGTGGTCTTTAGGAGACGTCACTATTGAGTCGGCAGAACTAAGCAATGCGATTTACATGTCAGGGTGGGAGAACTGCCAGAATGACTACTCCATCAAAATGAGGAGGTTGGTTATGATAGCCATGACTCAAACACAGAAACCACTGGAGACTAAAACACTAGGGCTCATACCTGTGTCCCACGAGTCGTATGTGTCGATTGTGAAGGCCTCATATTCGATATTCTCACTTATTTTTTATAATAATACATAA

>HrhoOR5a

ATGTGTACAAAAGCGTCAAAGGGCGTGCGAGCGGCGGTGGTGCGGTTGCGCGTTTGCGGCTTCTATCGGCTGGGCGCCGGCGCCGTAGCGGCGGAAGCTCACATGGCGTACCGCGCACTAATGCTCATCCTGACAGCAATCTACTTGCTGCAGGAGGTAGTGTATGCGGTTTGCGAGCGCTATGATATGGACAAGTTGGCGCGCGTTATGTTTCTGCTGTTGTGTCACTTCACATCCATCGTAAAACAGATTGTGTTCTTCGTGGACGCGGATCGAATCGATAACCTGATCACACTTCTAGATGAGCCCATTTTCTCGCGAGGGCCGGCTACACTGGAGGCGGCGGCACTCGGCGCTGAGCGGCTGGGGCGCGCATACAGCGGCACTGCAGCGGTCACCTGCATGCTGTGGACCGTGTTTCCGGTGCTAAACTTCCTGCAGGGCCACCACGTCGAGTTTCCTATTTGGACCGGTTCCATCAATTATAACAGCAATTCCCTTGCGTTACTATGTTTTACATTTTTATATCAATTACGCTATTCGCTTCTTACCTTTTCATTATTGCTTATTGAAATAGGAGGCGCAAACACTAACATAAATAGAATGATCACTAAATCTAAAATAACATACCTACCTATATCCAGAAGTTATTTTTGTTATGTAATTTAA

>HrhoOR5b

ATGGATGCGTTCCTCGGGACTCTGTTGCATCAAGCCAAGACTCAGTTAAGTATTTTACGGGGCAACTACGAAAATATCACCGAGAGGGCAAAAATTGTTGCTCAACTAACCAAAGAAGATTATGACAAAGTACTCAAAAGGTTATTTGTTGATTGTTTGACGCATTTCAAAAAAGTTTCCGAGTTGCTTGAGCTACTCCAGAGTATTTTTAGCAGTGCTATTGTCGTGCAGTTTACAATAGGGGGTTGGATTCTTTGTATGGCCGCCTACAAGATAATTGAGCTGAACGTGCTCAGTATTGAATTTACATCAATGGTGCTATTTATCTTGTGTATTCTCACGGAACTCTTCATCTACTGCTACTATGGAAACGAGGTGACACTTGAGAGTGAGCGAGTGGCAGGCTCGGTGTACGCGGCGCAGTGGGTGCCAGCGCCGGCGTGGTTCCGACGCGCTCTGCTCGCGGCGCTAGTGCGCGCGCGCCGTCCGCTGCGCCCTCTCGCTGGTCGGGTGCTGCCTCTCTCATTAAACACTTTCTTAAAGATTCTGAAATCCTCTTATAGTTTTTATGCCGTATTACGACAAACAAAAAACCAGGTTTAG

>HrhoOR6

ATGCGAAATAAAATTGTGTCTTATCTAGAAAATGAAGAGCATCCATTACTCGGCCCGACTTTGTGGGGCCTTAGAGCTTGGGGATTGTATCCGCCTATAAACAGTAATTGGTATACTATAATATCTTGTTCCGTACATCTTGCTGCTATAGCTTTTGTGGTTACCCAATATATTGAACTGTGGCTAATCAGGTTCAACCTGAACTTAGCTATGCGCAATCTCTCTATAACCATGTTGAGCAGTATTTGCGTGATAAAAGCTGGAACATTCGTCCTGTGGCGAGAGGATTGGATGAAAATAATTAATTTTGTATCAAGTCTAGAAAAAACACAGCTATCCGAGACGTATGCTATAAAAAATAATATTATCAGCAGATACACGAAATATTCCAGATGTGTAACATATATCTACTGGGGACTGGTTACAGCAACAGTTTTTACTGTTGTTATGGCGCCGTTAGCAATATTTTTGTCGTCACCCTCGACCAATGAACTCATGAGGAATGGCACCATTCCATATCCAGAAATAATGAGTTCATGGACACCTTTCGACAGAACCAGAGGTTACGGTTATTGGGCGTGCGTTGTTGAACATATGTTAATTTGTTTTTACGGCGGCGGCATAGTTGCTAACTACGACTCTAACGCCGTTGTTTTAATGTCGTTTTTCGCTGGTCAACTCAAGTTGCTGTCGGCGAACTGTACAATGTTATTTGGGGATGAAAACGAGGTTGTGAGTTACAACGAAGCGTTAAGGAGGATTCGAGATTGTCATTACCATCATGTACAGTTAGTCAAATATGCAAAGATATTGAACAGTTTGTTGTCTCCTGTGTTGTTTCTTTACGTGATTATCTGTTCTTTAATGATATGTGCGAGCGCTATACAACTTACTACGGAAGGAACAACGAGCATGCAACGGATTTGGATAGCTGAGTACCTCATGGCACTTATTGCTCAATTGTTCCTTTACTGTTGGCATGGCAACGAAGTTTTATTTATGAGCGAGAAAGTCGATGACGGAATATACACGAGCACGTGGTACTTGCAGAACAATGTATTACGCCGCAACGTGTTACTGCTACGCGGCCAGTTGAGGAAGCGTATTATATTCACTGCAGGCCCGTTCACTACTCTTACCATCGCCGCTTTTGTCGGGATACTAAAAGGATCTTACAGCTATTACACAATTTTAAACAAAAGAGATGATTAA

>HrhoOR7

AAGATGCTCACCAAAACGAGTGTCAAAAAAGAATTTTTGTGTGACATGGCGTTTTTAACTACAATAGGATCCAAAATATTCTTATACCCATTTGAAGGTAGAACAGGTTACAAAATTATCGGTTACACAACCGTGTGTTGTTTAATTTACATTACTATTGCGCAACTATTTCTTACTTTATTTGTAACTGGTTTCCATGATTTAATAGACATCAGTAATATTGCTCCTAACATCGGTGTATGTATCTTGACTGTTATTAAGTACACTAAAATCTATACCCACAGGAAACTTTATCATGCTATCATCACTCACTACCAAAAATATATGTGGAGCATAATCCAAATGAATGACATAAAAAATTTAAAAACGATCGTCAAATATAAATCAGTTTCATATTTTATCAACAAGATTTTGTACTACTATTCTTTGCCGCTGATCATCATTATTGTTTCATTGCCTCTAATTGTTATGTTTTATAACAAAAAATTCAATGATAATGAATTCGAGTATTTATATCCATTTGACGGCTGGTATCCTTTTGATAAAGTAATTTGGTTTTATTTTATCTACGCATGGGAGAGTTTTATGACGGCTCTCGTAATTTACACCTTCGCATTTAGTGATATGATCAATATGTCGTGTGTGGCCTACATGTGTATGGAATTGAGTGTGTTGAGCAATTTGCTTACTAATCTCATAACGAATGAAGACATTGAAGATTTAAAAAAATTCAAAAATATCTTTAAAATCCATCAACGAATACGGCGGTCATTAAAAATTATTATCAAGAGACACCAATTTTTAAATAAGTTAGCACAAGGTTTAGATTTAGCTTTAGAAGACGTGTCGTTGATCAATTACATATTTGGATCTGTGTTCATTTGTTTAACCGCTTTTACATTTACGTTTATCGATGACCTTTACAAGAAAATAAGATATTTCTTCTTTTTTATATCACTTAATTTCGTTATACTTAACCAGTCAATAATTGGACAAATACTTAGTGATCACAGTGTTAAACTAACGAATGCAATATACTCATCCAACTGGATTTACGCTGATCGCACGACGAAAACCACTTTACTTTATCTTATGATGAAAACTCAGATACCTTTCACGTTAACTGCCAAGGGATATATCAGCATGAATTTGAACACTATGACTCAGGTTACAAGTACCTCATATCAATATTACAATCTGCTGCGGTGTATTTATCATTCATAA

>HrhoOR8

TACTGGCCGTCGTCTAATGACAATTCTATATCCGGAGATGCGTTTAGAGTTACTACCACGATAGTTTTATGTATTTTGATGGTCACTATGTTATCTATCGATTGTTCGACGATGATCTACTTAATTATGTATAAATACAAATTCATAACGTTAAGAAATTTCTTTGAAAATCTTAGAGAGGAATTTGATAAAAATGTCAAAGAAAATGAGATAGTCGCAACAAATTTACTTACCGACGGGGTTATAAAAGGAATCGTTATGCACGAAGAACTATTAAGTTTATCACAAGACATCGACAAAGCGTTCGGCACGGTGATGGCTTGTCAAGTATGCCAAAGTTCTGGCTCGGCTGTGTCGCTCCTTCTACAAATTGCCCTTGCTGACCACCTTACGTTAGCTGGGGGTATGAAAATTATATTCTTCGTGGCGGCACTTTTTTTTCTCTTGGCATTATTCCTGTGTAATGCTGGAGAAATTACATATCAGGCGTCCCTTATGTCAGACGCCATTTTCTATTGCGGCTGGCACGCGAGTTTGCCGCAGCCGCCGCAGCACCGCGATCTCAGGCGGCTAGTGAGCTTCGCGTGCGCCCGCGCACAGCGCCCGCTTGTCATGAAAGCATTCAAAATGCTTGAACTGACCTATGCTACATTTATCTTGGTGCTGAGGAGTACTTATTCTGTGTTTGCTCTATTCTACGCACAAAACAAATAA

>HrhoOR9

ATGGTTGTGTTTGCAGGTTTCACCGTAATTCTATTCGTCAGTATCACTTACAGCATTTTCTATACTTGCTCTATATTTACCAGAATGCATTTTACAATTCTCAAAAGTGATTTTGAGGGAATCATCAATATCAAGAAACCTTTTGATGTGCAAAAGTTTAACAAAAATTTTGTGTCTTTATTCAAGAGACATAGATTGTTGATACAAATTGTGGATTTGCTCGAATTGGTTTATAATAAAGCGATTCTTGCGAACTTTATTTCTGGCTCAATACTGATTTGCCTTTGTGGGTTCAACGCTATGGAAATGAACAACAAAGTTGCGGTAGTGCCGTACGCTTTTTTCTTAATGATGTGTTTGATGCAAATGTTTTTTTTGTGCTTATGTGGTGATATGATATCTAGAGCGGTAACTTAA

>HrhoOR10

ATGTTACACGACAGGCGCCCGTTAAATTACTTTGGACTACATTTTTGGTTACTTAGATTTTTAGGCATCGGTTGGTGGCATGACCCGTTCTCAGAGGACAAAAGAAATTTTCCCAGCTGGTATCTCTACTACTCTATTCTGATGCAGATAGTTTGGGTTGCAGGCTTCGTAGGCTTAGAAACTATCGATCCGTTTGTGGGTGACAGAGATTTGACGCAATTTATGTTCAGCCTCGCGTTCGTCGTTACTCATGATCTTACAATCTTCAAACTCTTCATATTTTATATAAAAAACAAAGACATTAAGGACGTTGTCCGAACTTTAGAAGTCGACTTGTACGACTACTATCAACAAGATGAAAAGATCTTTGCTACTATAAAGAAGACGAAAATACTAACTGGAGCGTTTCTCTTTTTTGGCTGGATAATCATTGGCAATACTAATGTCCATGGAGCTATAGTCGATATTCAATGGAAAGCCGAAGTAGCTATGTTAAACAATTCATCAACAAAGCCTCCGCGGACACTCCCTCTGCCTATCTTCATACCATGGAGTTATCAGAACGATACATCTTATATATTTACATTTATTTTTGAAACAATAGGGTTGTTTTGGACAGGTCACATTGTAATGACAATTGATTCTTTTATTGGCACTCTAATTTTACATATGAGCAATCAATTTATTATGTTACAAGATGCTTACAGAAGCGCTTATGATCGAACAGTTAACAGAATGTTACAGAAACATAATTTTAATGACAATATTGATGCAGAAATAGAAATAAATAAACATTTTTTGGAAGCCGATAAAAAGGAGTTGATTGTTCGGAAGATTTACACCGCGGAACATTTCAATTCAGAATTAGAAAAAACTTTGAAGAGTTGTTATAAACAGCACCAAATCCTAATTGAGTGTGTACAAAAATTCGCTACCACATACTCGTACGGGTTTATGATTCAACTATTGTCGAGCGTGACAGCAATTTGCGCATTAATGGTCCAAATTTCGCACGATGCGTCTTCGTTGACGTCATCACGCCTCGTTACGTCACTGGCATTTTTTGTTGTAATGATCATTCAACTAGCAATACAGTGCTTCACTGGAAATGAACTTACTTATCAGGCGGGCCTAGTTTCGGAGGCGGTGATGGAGTGCAAATGGGAACACATGCCCGTGCGGCTCGGCCGCATGCTAGTTCTGTGTTGCGCGCGCGCTCAGCGCCCGCTGCGCCTCACGGCCGCAGGATTCACGCACATCAACATTGACTGCTTTCTTAGTATAATGAAAGCCGCTTATTCATATTATGCAGTTTTAAGCCAGAAGCAATATTCAAATTAG

>HrhoOR11

ATGCTTGCCTGTGCTATGGACGTCTTAGAAAAGTTTACAAATGACTACAAAAATACTTTCATCCGCTATATCAAAATGATAAATTTTATAGGACTTGATTTCTATTTAGAAACAGGAGAGAGTTTATTTAAAAGTCGTAAAAGATATTACTTATTCCTTATATGTTTTATAATATTTTTCCTTTGTCAAATCACATTCATATTTAGAAGTCATAAGACAGATGCGAAATTTCTGGATATAGCTAACGCAGTACCTTGCTTGGTGCTGGTTATTCAAGATTTTGTTAAACTCCTGGCTATAACAACAAAGAGAAAGGAAATAAAAAATGTCATTTTTAAAATTAATGATGAATGGCCTAAAGAAAAAAATTACGGAGAAAGATCTAACATAATTGAAAATTGGACTAAACGAAACAAATCCTTTCAGAATGTATATTATGGCATTTCCTTGTTCTGCTTATGCATATACGAGCTGATACCCTTAGTAGCGACATTGTACAACAGAGTGATGGGACTTGACACTGAATATTTTTTCCCCTTCGAGTTGTATTACCCATATAAAGTCGACTCTTTCTTTGTGTACCTTGTGACTTACTTTTGTCAAGCTTTCGCATCTTCAAGTTTACATGCGTGTATATATATTGCAAGTGATCTACTTATAACATCGCTTTTATCAGACGTAACAGCGTTGTTTGCTTTGCTCCAGTATGACTTGGAAAATGTTACTTCACAACTGAAACAAACATCGATAAATCAAGATGAAGAACAATATAATTGCGCTGTGAAAAATATAGTAAACCGTCATCAAAGATTACTTGGGATAATAAAGGAACTGAATGGAATTTACGGCGTTGTTATCTTTATTTTCATCACAAGCTCTTCTATTATTTGTTGTTTTTTCTGTTTTCTAACAGTGGTTCAGAATGGATTGCAGTCTGTAAAAAATCTTCTAGCCGGCGGTGCGATGCTTGGGGCGATTCTTGTTGTGGCATTTCCTGGACAACAACATTATGACATGAGTTTTGGTGTAGCATATGCAGCATATTGTAGTCTTTGGTATGAAAGAAATGAAAAATTCAAAAACTTGATATTGATTCTTATAGTAAGATCCCAAAGAGCAAGTTGTTTGTCAGCTCTTGGATTTTCTGACGTGACGTTGGCAACTTTTTCAAAAGTCAGTTTTTAA

>HrhoOR12

ATGTTTTTTCTGACAAGTGCAATAACGACTTGCACTCTTTGGGCGATGATACCACTGTTCGACGATGCTGGGAGCAGATCATTTCCGTTCAAAATATGGATGCCGGTAGACCCACAAAAGTCTCCCCACTACGAAGTAGGCTACGTCTATCAAATGATCACAATCTACATCAGCGCCTGTCTATTTATTGGAGTGGACAGTACTACCCTCTCCATGATTATGTTTGGGTGCGCCCAAATTGAGATTATCATGGAAAAAGTGCGCCAGCTGCAGCCATTGTCGAATATAAAACTAAAACCTTATAAGAGAAAACAGCAAATAGAAGAAAAAAATGCTCTTTTTATTGAATGCATAAAACATCACCAGGAGGTTGTTAAGTATATCGAGAAAGTTGAAGACACGTATCACGCGAACATATTCTTTCAACTCAGTGGAACCGTTGCGATTGTTTGCATTGTTGGACTTCGAATATCTATTGTAGACAAAAGCAGCGTGCAGTTTTATTCAATGGTGAATTACATGGTCACTATGCTATCGCAGTTATTCCTATATTGTTGGTGTGGCAATGAACTTACTATTCGAAGTCAAGACCTGCGAGAGGTTATCTACCAAACGCCATGGTACGACTTTGACAGGAAATTCTGCCGTCTTTTATGGGTTGCTATGGAGCGGATGAAAAGGCCTATTATTTTCAAAGCCGGTCACTACATAGCGTTGTCCAGGCCTACTTTTGTATCGATTCTACGTTCATCGTACTCCTACTTCGCTGTACTTAACCAGGCGAACAAATAA

>HrhoOR13

ATGGCATCTGTAAAGTCACATTGGCTTTTTGCTCCAAGTAAAGATTTTTTTGAATTCAATCTGAAGTACCTTACCTATTTGGGTTTGTGGCCTAAAGAAGATTGGTCAAAACCACAAAGGTTTTTGTTCGAAATATATAGTATGACGTTAAGCGTTTTTGAGGGTGTATTTTTAATATTGACAAGCATTGGAACATACAACTGTAAAAATGATATAACCGCATTATTAACTAATCTGGATAAAATCTTAGTTGTTTACAACTTTGTAATGAAAGCCTTAATATTTTTTATAAAAAGAAAGCAAATAAAAATTTTAATTGACGAAATTAAACATTCAAAAGATGAAATAACAATGAGTCGTAATAGAATGATGTCAGTACATGTTGTTGTAATATCTGTGCTGGTAATATCGATTGTTAGTGCATTTTCTTTGTTGGCGACATACAAACAAGAAATGACGATCGAAGCATGGATGGCGTTTGACCCGTTGATTGACAAAAAGCATCTGATTCTGGCATCGCTAATATTAGCGGTATTGTTCGTGCCCTGTGCGTGTAGAGCGATGGCTATACAAGGGATAGTTTGCAGCATTCTCATGTATTTGTGCGATCAACTGATTGAATTGCAAATTCGGATACGGGCGTTGGATTATCGCCCAGAGACGCATAAGCAAATGAGGGTAGATTTCAATGAAGTTATAAAGAAACACGTACGTTTAATAGGCCGCTTTTTCAGATATTCTAATACGTTGAGAGCAATATTTAAAGAATATTTTTTATTCCAAAACTTGGCTGTTACTGTGGAGCTTTGTTTTAATGCTATGATGGTGACAATGGTGGGGTTCAAAGAAAAGACATTATTGTTAACATTCTTTGCATATTTGTCCGTGGCCCTGCTTAATTCATATATTTATTGTTTTTTGGCTGATGAACTCATCGTCCAGAGCCAAGGGATTGCTCTAGCAGCTTACGAAAGTCAATGGACTACATGGCCAGTGGAGTTGCAAAAGGATATTTTGATCATCATACTTGTTGCTCAGCGACCGTTAACCTTGTCTGCTGGAGGCATGGCAACTATGTCAATTCAGACGTTTGGTCAGACACTGTACAATGGCTACTCTATATTTGCTGTATTGAGTGATGTGGTTGATTAA

>HrhoOR14

CACAAAGTGTTACTAAAACGGCAACACTTAACAATGCCCAAAAACGATTTAACCTTTGCAAACAATTTTAAACTCACGACATTCGCGTTAAAAGTTACAAGATGTCATCCGGACATCATCAGAAACAAATTCTGGATATTTCAAATGTTACTTATCATTATAATGAATACAACAGTCGCTTGGATACTCCTCAACTCTGTCGTATTTCACGACATCAAGTCCGGCGACTTTGCAGAGGTGTCCAAGAACGTTGCTATGTTCATAATCTGTGTAACTATATCATTCAAACTGTATATCATTGTTAGAGAATGTAAATTTCTTATTGAGACAATGAACAACATGAACCAAGATTATCGTGATTCGCCCGAAAATGAACGGAACATTATTATTAAGTATACTAAAAGAGGTGCAGCCGTGAGTAAGTTTTGGTTGGCAGCGGCTAGTGCCACCTCTTGCATTTATCCAGCGAAAGCTGCTGTGGCAATGGTACACGCGTATTTGAACGGTGAATTCCATTTGGTCCCGATGTTCGATGTGGCTTTCCCCGAAAACTTGAACGAGAAGAAACATAACCTCGAGATATCTCTACCGTTTTTTACACTATGTCTTACATTCGCTTTGTACGCTATGACAGTGTACGTGTGTTTCGATCCTTTAGCTCCAATTTTTATGCTCCACGTCTGCGGTCAAATAGAAATAATCAACACTAAAATTGCCCAAGTATTTTGCAATAAAGATTTGACTAGGGAAAACTTTAAGACCATAGTAATCAAGCTACAAGAACTGTACAAATTCATACGCTGTATCGAGAAAAAGTTCACAATTGTGTACGAATTTGTAATGAAAACAACTACTATATTACTACCGCTAAGCGCTTTTCAAATTGTTCAGTCGGTACAACGTCGAGAAGTTAATTTGGAGTTCATTTCGTTCTTCAGCGCTGCAATGTTACACTTCTGGCTACCTTGTTACTACAGCAATCAGTTAATGGAGAAGGGTGAAGAACTGCGAAGCGCTATTTACTCTTGCAAATGGGAGTGCAGTAACGATCTTCTAGCGCGGAAGATGGTGCTGTTGATGCTAGTGCGAGCTACCAATCCTCTGGTCATCAGTTCCGTCTTTTACACCATCAAATTGGAGACGTTTACTCAGATGTGTCGCGACGCGTACGCAATATTTAGCATAATGAACGCTGCTTGGTCATAA

>HrhoOR15

ATGGATAAACCAATGTTGGTTCAATTTTGTGGTTCCACTGGAATTGTATGCGCTGTCGTTTACAAAATGACTGGAATTCCTTTTAACACTACATTTATATATCTTGCTTTGTATCTGGGATGCTTATTATTGGAACTCTACATTTATTGCTACTATGGTACATTGCTGATGAATGAGAGTTTACTTGTGAATGATTCAATATACCTGAGCAACTGGACTTCATTGTCCCCTCGTTTCCGACGAATAATTCTCATTGCAATGACTCGTTGGTCCAGACCCATGACTCCAACAGCTGCTGGTCTAGTGTCTATAAGTCTCAAAACTTTTGTCTCGGTATTACGGCTCTCGTACTCGATATACACTATAATAAAATCTTAA

>HrhoOR16

ATGAATGATCCCATAAAATCCAAGTATATCCTGCGTAAAATTACTACTTTCGCATATATAAGCGGAATACCGTACTTGTGGTACGAAGAATTGGGATGGCCTAAACAATTAACAAATTTCCATGATAAAATTACTAATATCTTGACTGTACTTCTATGCATCTTCATGAGCTTGGAGATCTTGGCTCTCTTCACTCAGAAAAACATGAACAATCAACAAAGCTCTGATGCTCTTAAGTACGCAATATCGCAACCAATATTTTTCATGAATTTTTTTAGTTTTGTATATTACAAAGAAGAAGTTCGAATATTATTTTACAATTTAACAAATAGTATGTCGACTTATCACAGAGATGAAGAAGTCGAAAGGAAATTAGTAAAAAAAATAAAATTTTATGTGTCAGCTTTTATTAGTGTTGCTATTGCTGCTTCAATGTCGGCTGGACTGGATGGATTGTGGAGAGTTTTGGAGAAAGATGATACATTTACGACAGTCATTACAGCGTGGCCCGACGTTGACGACAGAAGTTTAGCAGCTGGTATTGGCCGAATCATCGCTTTTATAATGTGGTGCATACATACGGTGCGCTTTGTTGGTGGTGTAACAATTGTAGTAGCCCTAACGGTGTGTGTCTGCCATCAGTACAAATATTTACAGAGCTACTTTTATAGTCTTGATAAGATTTTTGAGATAAACTGTAGTCAATCTGTAAAAGAAGAAGAGTATGAAAAAGGCCTGCTGATTGGTGTAAAGCTTCATAATAATATTATAAGATACACACAGGATCTCTGCAACGTATGTAACATAGCTTACGGAGGACAAATTGTTGTAAATGTTACTGTGTTGGTCATTTTGATGGTACAAATGCAGAATGGAAATAGACAATTCATGTTGGTATTAACTTGCGCAATAATGGTGTCAGCTTTACTAGTGCTCAACGGATTTTACATGTGGAACCTCGGAGATATCACCGTCGAAGCGTCTGAGGTGTGCACAGCGATGTACATGTCCGGTTGGGAGAACTGCACGCGACGCTCTTCAGTGCGAGTAAGGAAAATTCTGATGGTAGCTATGACACAAGCTCAGAAAGAAGTTACAATAAAAACTCTAATGATACTGCAAGTTTCATACGCATCGTATGTGTCGATCGTCAAATTTTCATACTCCGTGTTTTCACTTATATATTAA

>HrhoOR17

TCATTGTTTGATCATAAAGGTTCTACTCGTTTTACTTACACCACGTTGTCAGTTTTTGTTCCTCGAACAAACGTTAGAATGGAATCGTTGGGAAGAATTGATTGTTTTAAATTTAATATTAACTTCTGGAAGTTTTTAGGAGTGTGGCCTGAAACTAAGTCATGTTACTACGCATTTTATTCTAAAATCTTCACTTTTGCCTTTACGTTTCTTTATGTTATTTTGCCGACGGTGAATTTAGCATACATTCCACCAGTAATGGATATTTTCGTGGAGGAGATGATGTTTTATTTTACTGAAGTAATTGGATTGTTTAAAGTTCTAACAATGTTATTAACTCATGAAAAGATTGTAAACATATTAAAAGTACTGCAAAGTGACTTGTTTCATCCTGAATCATCTGAAAGCGTAGAGATTATAAATAAAGCAAAGATATTCATTGTTAAATATTGGAAATTCATAGCTACTGTCTCAGTGACCTCCAATCTCACTCATGTTTTATCCCCATTGATTGTCCATCTTTTATTATCTGTAGAGTTACAATTGCCGTTGTGCAACTATTTTTTTTTACCTAAAAATATTAAAGATACCTTTATTTATCCCTTATACTTATATCAATGCTTTGGGATTCATATGCAAATGTGGTACAACGTTAATTTTGATTCATTCATTTTGGGGCTTATGATTTTGGTTATAGCACAGCTAGAAATATTAGATCTGAAATTACGCACAATAACGGATCGTTGTGCGAAATTATATATAAAAAGAAAACAAAATGACACTGAAGTTTTAACAAAACTCAATCAAGCCCTACAACACTATAATGAACTAGGCAGGTTTTGTAATTTGATACAAGATGTATTCAGTATTGCTTTATTCATGCAATTTGGTATCGCTTCCTGTATAATTTGCGTTTGTCTCTTTCGCTTTACATTGCCAGCACCATGGCAGTACTACATATTTCTAGGTACATACATTTTGGTCATGATCTTTCAAATTTTAATCCCTTGCTGGTTTGGAACCCGTATCATTGAGAAGAGTCAGCTGTTAAAATTTTCTGTATATAGCTGTGATTGGACATGCCAGTCACGGATATTCAGAAGCAGTTTAAAAATCTTCTCCGAGAGAGCAAACCGTCCCATCACTTTGATAGCTGGAAAGATGTTTGCGCTGTCTCTTGGCACGTTTACTTCGATTATGAATTCTGCGTATTCGTTTTTCACTCTTTTACGTCACATGCAAACTAGAGATGATATGGATTAA

>HrhoOR18

ATGGCACATGTAAATAACGAAGATATGTATTTAAATAGAGCGAAATTTGTTATGAAAATCTTAGGCGTATGGATGCCATTAGAAAACGAGACGAGATTTAAAAAGATATATAGAACTGCCATGATGTTTTTGCAGTACATATTTTTAATTTTTCAAACGATATTTATATTTCAAGTGTGGGGCGATTTAGAAGCCGTGTCTGAAGCCTCATATCTGTTATTCACTCAGGCGTGTCTTTGTTTTAAAATAACAGTCTTTCAAATAAAAATCCCGATGCTCCGAGATTTATTGAAACAAATGAACGCAGACATATTTAGACCACAGACTTTAGAACATATAAATATTCTAATATTGCAGGCAACTAGGATCAAAAGATTTTTACTAGCGTTTATGGTGAGTTCTCAAATAACATGTGGGATGTGGGCGCTGAAACCTTTATTTGACAATGCTGGCAAGAGAGAATTCCCTTTTGATATGTGGATGCCAGTTAGTTCCGAATATTCTCCGCAATATGAGTTAGGATACGGTTTTCAATTGATCACTATATGCATGAGTGCTTACATGTACTTCGGTGTAGACAGCGTCGCATTGTCTATGGTTATCTTTGGCTGCGCACAAATTGATATAGTTAAAGCGAAGATTTTGAGTATAAAATCGATTTCAAACTCAGTTGGAACCACGAAAGAACATACGAGGCGTATACGCAAAGAAAATTATAACAAAATAATTGAATGCATAACTCAACATCAGGCCGTACTGAAATTCATCCAAATGACAGAAAACACGTACCACGCGTACCTTTTCTTTCAACTTACTGGCAGCGTTGGGCTTATTTGCATGTCAGCGCTACGATTAGTGGTCGTAAAATTTCCGAGCATCCACTTCTTCTCCATAGTCTTTTATGTATCAGTTATGATAAGTCAATTGTTCATTTGTTGTTGGTGTGGGCACGAGTTAACAGCAACGAGCGAAGAGTTGTTTGCCACTCTTTACCAATGTTTGTGGTACGAGCAAGACTTCAAGTTCAAGCGCGACCTTCGTTTCATGATGATGTGCGTGCGTCGTCCCGTAGTGCTCAAAGTCGGAAATTATATTACTCTCTCAAGACAGACTTTTGTTTCGATTTTACGCATGTCATATTCTTATTTCGCTGTTCTTAACTCTACTGAGAAATAA

>HrhoOR19

TTGGACAGGCGACACGACGCCATGCCACTGTTGAAGAAAATTAAAACGTACTTTGATAAGGAAGGTTTTGACTATTCCAAAAACTATATAAATCCATATGAATTTCATTCAACGTTTTATTTTTTTATGAAATATTTCAAAGTTATTGACGATGAACCAGCACCGAAATGGGCTAATGTTGTTCAAGTATTAATTGGCGCGGTGGGATTTACCAATTTATGTTTGTCGATATTAATGTGTGCTATCAACAGTGTAAAGCCTTTTACATTGCCTAAATTCATAGAAGGTGGAACCTACATAATTGTTGTATTTTATGGTATTTCCATACATTTATGCTCAATATTTAACAAGTCAGGGTATCATTGTTTATTGAGAATGTTGAGGCAAGATTTTGACTTCATCTGCACAAGAGGACAGACATACCGGAAGAAGTTTTTCGAAAACCATTTAATAATTTGGAAACTTTCAATCGTTTCCATAATATTTACTCAAAGTATTGCAATTGGAATGATAGCTTTTAGTATTATTTTACTTTCTTACTATATGGCCACTCACGAACCAGGAGACGGCACCAGCAGGCCTCTGTTGATACCATTTTGGATTTTTAATTTAGATTTAAATAAATCACCAATTTACGAAATTCTCTTTAACTACTCACATTTAGCACAACTTTGCTATGGATTCACTTATGTCTTCTTGGTTCAAACTCAAATAGTATGGATTAAGCATATAGAGACCAAAGCGGATATAGTTATTTGGTTATTAAATGATCTTTTTGATAATCTTACATATCCCAATACCGAAGAAGAAAAAAAAGTTTGTGACACTGAAATAAAAAACAGAATGTGTTTTATAATCCGGCAACATCAGTCTGTTTACACGTTGTTAGAAAGTTATGCGGCAGTATATAGAAAATTGTTGATGTTTGAACAAAAATTGTGTGGTCCAGTGGTATGTTTTGCCTCGTACTGTATTGTGCTGCAACTAGAAGCGGGAGAGTTTAACGGTGTGTTGTTATTACTTTGTATCGGTGCACTAACACTCACTTATATTCCGTGTTATTTATGTACAACGTTATCAGAGAAGATAATGTCAGTGAGTGATGAATGCATGAACATACCATTTTGGAATGCAAATCCAAAAATAATCCGGCCTTATTTAGTTTTAATGATAAGACGGTCGCTTCGGCCGCTACCACTAAAAGTACCGGGATTTCAACCGCATACTCTACAGACATTTTCTAAGTCGATGGTATCGGCCTATTCGTTATTCAACATGTTGAGACAAGCAAACGTACAATAA

>HrhoOR20

TTACTGGATAATTTTAATAAGGACTTCATATTTATTTGCAGTTTAGGTACTTCTTACAGAACGAGTTTTTTAAATAGCCAACTAATAGTATGGAAATTGTTCATAATTTGGATAACATTTTGCATCACCATATCTTGTGCGTTCGTGTTAAACACTGTTGTGTACCTAATGTACCAAACTCTATTTATGACAATCACCGAAGACACAGTCCGTCCGCTGATATTCCCACTGTGGCTTCCTACCGGGGACGATCCTTACAGGTCGCCCAACTATGAAATGTTCTTCGTGTTCGAAATACTTGGCACTGCTCTTATACCAACCGCTTTTGCAGTTTATACATATAACCTGTTTCATCTACTTCTTCACGTGTACAATTTGATGGACGTGATGATTAAAGCAATCAGCGAGCTATTCCTTGGGCTGAATCCAGACGTTGCTGACCTACCTGCTCGTAACCCTGAACGACAAATCACTCAAGCTATTCTAAAGACGAAAATAAAACAGATCGTCAAATGGCACAAATCTGTATATAAATACATGAGTACCATTTCTTCAATATACGGTCCTGTTTTAGTATATCAAGTGATGTTCAGCGCAATTGCTATATGTGTTATGGCAATCCAGATTGCCAACGCGCTGGATAAAGGTAAAATTGATTTCCTCTTCTGTATGTTGGAAAGTGCAGCATTACTGCAGCTCTGGATAACTTGTTATATTGGTACTATGATTAGAAACAAGGCTTTTGCAGTGGGAGACGCTTGTTATAACAGCGGTTGGGAAAATTCCAAATTTGGGTGCTGGCTTCGCAGCGATATTGTTTTAATAATCATGCGCTCCCAGATACCGGTGTCCATAAAATTCCCACTGCTACCGCAGATCGAGCTTGAAACCTTCTCCTCGATAACAAGCACTTCTTATTCGTACTTCAATATGTTACGCCGAGCTACTTAA

>HrhoOR21

ATGAAGCGGCTAAAACATAAACATATTATAACATCAATTAATTCAATAAGATTTTTATTTGAACTTAACGGTCTCAAATTGGAAGACACAAATTCGTCTGATAACTTGAAGTACAGAATTATTTACGCTTTTAATTTTACATGGCTGAACTTCGATGGCATTGGTGAACTAATTTGGTTTTGCGACGGGATATGGTCGAGCGAGCACTTCGTTAAGCTAGCGGCTATGTTTCCTTGCGTGATAATGTGTTTCTTGAGTAACATTAAAATGATATTGCATAATTTAAACGAAAAAAAAGTGGCTGATTTAATCAACAGTTTCAAGGAATTAGAAGACGACGAATTCATTAACGATACTGATGCAGATGACCGTTCGAAAGAGAAAATATACGAAGAAGAAATGAAATATTTAGAGAAAATAGTAAAAATTGTTAAGGTATTGAATGTTGTAACAGTAATAGCTTTCGGTATAGCTCCATTTCTTCTTATGGGCGCACATTACTTAGAAACCAAGGAATTCATGCCTGTACTGCCATTTTATGTCAAGTATTATTTGTTTGACCCGTACAATATGAAATATTACGGTTTGTTATATCTACATCAGTTTTGGTCAATGTGTATTTGTTTGATTGGAATCCTCGGAGTGGACATGCTTTTCTGTACTATGTGTGTATTTATCAAAATACATTTCAAGCTGTTGGAATATGATTTTGAAAGATTCATACCAATTCATACAACACCGCATGGGTGTTTGAGAGAGAATGAAACAATTACAAGAAGATTTAAGTGGTTGGTTAAAAAGCATCAGAAAGTTATAAGTTGCAGTAATTTGCTGAACAGAATTCACTCAAACGAGTTTATGATGAATTTTTTTACAAGTTCTTTTCTGATTTGTTTATCGGCGTTTATTATAACGGTCGTTGAAGAAATGAGGTTCAGAATTTCCTTCTTGAGTTTCCTAGTAACAAGTCTACAGCAATTGTTCTTGTTGTGTTTCTTCGGTGACATGGTCATGACGTGCAGTATCAATTTAAGTGGTTCCATTTACAGCAGTTTATGGCATTCGGTGAAATGTAATATTGGTAAACAGCTATCTTACGCATTACAGAGATCACAAAAACCTTGTAAAATAACAGCGGGTGGCTTCATAGACGTCAACCTTATTGTTTTTACACAGATAATCGGCAAGACGTGGACTTTATTCGCACTTTTAAGAACAATATTCAACCCTTGA

>HrhoOR22

ATGTTAAACGATGAAGGTAGCTTTGATGTTAATGAGAAATTCAAACCTTTTCACGAAACATATAAAATATTTACATATATAATGACAATGGGACTGATATATCCTAACCCTAAAACAGAGAAAATCCGATTGAAACTAATATTATTTGCGATTTTGTTTGTGTCGCCTCTTCTGTTTCTGATAGGCTACGATGTGTACAAATGTTGCTTAAGGCACGACATCGTCAACATCATCAGACACAGTACGGTGGCAGGGCCTATAGTATTTATATTATTAAAGATAATGAGTTTTTATTACAACAGAGATCTGGTGAAAGAACTAATAGATGAGATCAACAGAGATCACGTTAGATACAATAAACTTCCTACAAAATACCAGGATATCGTTGAAAAATCTTTAAGGTATCACAAGACAACTGAAAAACGTTGGGTGGCTTGTGTCAGCATAAGCTCTTTCCTGTTCGTTATTATGGCCACAGTATTCACCATATACAGTCAAATATTTGATGCGGAACCCATGAAATACATGATTCACGAGATAGATGCGCCCACAATTGAGAGCATTATTGGCTGGCCGTACTACGAAATTATGTTCGTCTATGAAAGTTACGTGTCGATTTATTTTGTATTGAATTTCTCAGGGTTCGACGGGTTCTTTGGTGTTGTCATAAACCACGCATGTCTCAAAATAAAGATATTTTGCAACGCGTTCTCGGATGCACTAAAAGAATCTAATGAGGATGAAATAATGCGTTTGATCCATGAGATCATTCGTGATCAATGCAAGATGTTTAGTTTTGTAAATACCATTCTTGCGGTATTTTCTTCATGGTTCGTGTGTATTTTGATAGTTGCCTTGGCACTGATATGCAACTGCATGTATCTCGTCATTCAGGGGCACGGATTCGATATTCGTTATATAGTATTCACTATTGCAACCATAATACATATTTTTATGCCGTGTTGGTATGCTTCGAAGCTAAAATCCATGTCGCAGGAGTCGTCGACTATGGCATATTTTAGCGGGTGGGAGGACGTCCCCATTCCGAGAGTACGCAGGACGTTGATGTTCTTCATGGCACGGGGTCAGGTGCCTTTACAAATCGAGGCTCTCAATATCATCAAATTTGACATGGAGTTGTTTGTCTCGATTATGCGGACTTCGTACACGATGCTTACATTACTTCAATCATCATCTTAG

>HrhoOR23

ATGAAAAATTCTGAATGTTTATCAGCTAGTATTGCTATACTAAAAGTTACAGGCGTATGGTGGAGCGATAGCATGGTTTATAAAATTATTGGCTCACTGATTCAACTTTTTCTTTACGTCTTCACAGTTTTAGCAGAGATTGCTTACGTTTTAATGGTTTTAGGTGATACTGAAAGGACCGTAGATGCAGCGGTGCTGTTGCTTTCGCACTTGGTGCAAGGTGTCAAAGTAGCCACCGTTTGGTTTAGACAACGCCGGATTAAAGGACTAATCAAACTAATTGACGGTCCAAATTTTGAAAAAACAGATCTAATGAAAGCGAAAATGATAGAAAGCTTTGGAGCACTGCTGAAGTTATCTGGTCACTTGTTTCTGTCTACTGCAGCGGTGACAGCGTTGTTTTGGGTTATCGTGCCAATGTTGAAATCAGAAATCACGCTGCCTTTGAAAACGGCTTACCCATTTGATATAAATGACCCAGGCAGTTTCACCTTGATGTACGGGTACACAACAATATCTGTGGTGCTGGTGGGAGTGGGCGACGCAGCCGAGAACTATCTTCTTGCAGCTTTACTAATACTACCTACGATACATCTTGAAATTCTTTGCCAGGAATTACAAGAACTAGACCATGGTGACGATATTTACGAAAGAACCGTGTCCTGCATAAAATACCACCAGCACATCATCGAGTATGCTAATGAAGTTGCATCTGTATTTGGGATCATAATGTTTTGTCAGTTCGTCACCAGCAGCGTTATTATATGCATGACATTGTTCAAGATAACGATTACCACGGAACCGATAGAGATGATAACTATGGTGTTTTATTTGGTTTGCGTGTGCCTGGAGTTGTTCTTATATTGTTACGCTGGAGACTTGCTAATGAATAAGAGTTTACTGGTGTCTGAAGCGTCGTTTCCTGGAAAATGGTTAAAAGATACTCGCAGCTGCCGCGCTTTACTTATGACTACGGTCCGCGCGCAACGCCCTCTCATAGTCAAAGCCGGCGGAGTATTCACCGTATCTTTACCTACTGCTGCTGCTATAATGCAAACAGCTTACTCATATTACGCAGTCTTACAACAAAAAACTAAAGAGCACAATTAG

>HrhoOR24

ATGAGTTCATCAAGAAGACAAGACGATCCACAAAATACGAGCAGTATCATAATATCGATAATAATGCAATCCATCCAATTTATAGGGGTTTGGTCTAGCGTTGGTTACAAAAGATCTATAGCTAATTTCGCCACTATTTGTTTTATTTTAACCATTGGAGCCCAAGTCATTAATCTCGTTTTAGAAAGAAATGATTCCGAAAAAATGATGGAAGCTTTCAGCGTCTTTTCAGTCTGTCTTATGGGACTCTTGAAATATATATCATTGCGCCGCAATTCAACTGCTTGGCAGTATCTTTTATCAAGAGTTTCTCAAATAGAGAATGAAAAAATGAATGAGAATAATGATAATTTATTAGATTACGAAACGGACGATGACAATAAAGAAACTGTACCCTCTGTCAAACATGTCTACACCTATAATGATAAAGCTAAATTTATATCGACTGTTCTAACAAGATTTTATACATTAACTGTATTTATGTTTGTTTCAACACCAATCTTTGAGTACTTTTGGAAGACTTACAAAAACCATGAACCGATGAAATTGCCGCATATTTTGCCGGGGTGGACGCCTCAGGATGACTTTCATTTTTGTGCATACTTTATTACAGTAGCTTGCGAAGCAATAGCAGCAGTTTATTGTGTAAGAATTCACGTTACTTTCGACGTTACTTTCGTCACTCTCATGATATTTTCCTGTGGTCAATTTAACTACTTGTGGGTGAAGAGCGAACGAATAGGTGGTAGTGGCAACAACTGTCAGTTATCAATAAAAAGAGACAAGAGGGCAAATTTTAGAATAATGCAATGTCACAAATCGCATATCATGCTAGTTGACCTGGTGACGAGGTTGAATAAGTTGCTGAAAATTATATTGGGAGTATACTTCACGGTGATTACACTAACACTGTGCACGGTAGCCGTACGACTACGTGCGTCCGACAAGTTGGGTCTCGTGAAATTAATTTTACTGTTGCAATATATGGCAACAAATCTGATGCAGCTGTATCTCTACTGTCGATATGGAGATGCACTTTTAAATCAGAGTTCTATCAACATGGGTGAGGGTCCATTCGGTGCGGCATGGTGGGCGCTCTCGCCGCCGACGCGCCGCCACCTGAGCCTGCTCGCAGCCGGCATGTCGCGTCAACAATACATGAGTTCAGGCATCTACATCGATAATTTACCTGCATTTCTGCAGGTAACTTAA

>HrhoOR25

CTGACAACACTCAACAAAATGACGATATTCATAAGAAACGTGAATTTGTCTATCAGTGTGTCTTTAACAGTATTAAAATTGGTCGGCTTTTGGGCGCCAAATGATATAAAGAATAATTTTAATATAATATATTATTTATATGCTATTGTAACATTCATGATATTATTGGGTATATACTTAATCATTCAAGTGGTGGATATGTTCCTTATATGGGGTGACCTGCCCCTCATGACGGGGACCGCTTTCCTTCTCTTTACGAACCTAGCCCAGGCCATCAAGATCCTAAATCTAGTATGGAAGAGACAGCGGGTCGAAGAGATTATATTTAATGCCAATCAATTACTGCGGGATCAGAGAACTGAAGAGGGAAAGAATATCGTTAAAAAGTGCGATAGGGAGACAACGCTGCAGCAATTGTTGTACTTCTGTTTGACAACCATTACCGTGGCAGGATGGGCTGGAAGTGCTGAAAAGAATAAACTGCCTTTGAGAGCTTGGTACCCGTATGACACTTCTAAATCACCAGCATACGAGATCACGTATGTTCACCAAGTGGGTGCGTTGTTTGTGGCCGCGTACCTCAACGTGGGCAAGGATACGTTGGTCACAGCGCTGATAGCTCAATGCCGGTGCCGGTTGAAGCTGGTCGGGCTCGCCCTCAGGAATTTGAACGAAGAGCTGAAAGCAGATGATAAGCATATATTCAACGCTGAGCAGCAGTTTGTGGTCCGTCGCCGCCTCAACAGATGCGTGGTGCAGCATCAGTCAGCGCTGGAAGCGACTGTTCAACTCCAGGATTGCTTCTCTGTACCGACGTTCGCGCAGTTCACAGTGTCGATGGTCATCATATGCGTCACAGCATTCCAACTTGCTTCCCAAACGGGCAACTTGGTCCGCGTCTTTTCTATGGGCACGTACCTGCTTAATATGACGTTTCAAGTGTTCCTCTATTGTTACCAGGGAAACCAGCTCTCCGAGGAGAGCATGGAGATCGCGGGCGCAGCGTACGAGTCCCCGTGGTACACCTTCTCGTCTTCAACGCGACGGTCCATCCTGGTGCTGATGTCGCGCTCGCGCCGTCCAGCTCGGCTCAAAGCTGGAGGGTTTACGACTCTCTCCCTAGCATCCTATATGGCCATAATCAAAGCGTCGTATTCATTCTTCACTGTGCTCCAGCAGGTGGACGAAAACAAATAA

>HrhoOR26

ATGGACGACATCAATTCCGACAAAATACCAAATTACACCCACTACATAATTTTGCCACTAAAATTGGTCGGTTGTTGGGACTGGTACAAAAATCCTGAGAAGGAATACCAGATAATAATCAACAATGGATACTATGCTTTAGTTTTATTTGTGTTAATGAATCTACAGTGGAGTTTGACTGTAAATTTGTATACAGAATGGACTACTATCATGGATAATTTGGAGAAATTGGCAGACAGCTTGCCTTTATTGGTGTCTCTTGCAATAATCATACATTTGGCATCGAACAAGAAGAAAATGTACGAACTAGTTGACTTTATGAACAATAATTTCAAGTATCATTCAGCGAGAGGTCTGACTAATATGACCATGCGCGAGAGTTATACCACAGCAAAGAAATTTGGTTACGTGTACACCGCGTGCACTCTTTTCAGCATCACTGTGTATGTATGCATGCCTTTGTTAAGTTACGCATGGACGAAGCAACCTTTACAATACTGGGTCTACGTTGACGTTTCCAGTGTTTCAGACTTTGTTATTGTGTTCATAAGACAATGCGTGGGCCAGATTTTCGTAGGACTGGCTGTTGGGCAATTGGGAGTATTCTTTGCATCAAATGCTATCCTGATATGCGGTCAACTGGACCTGTTGTGCTGCAGTCTCCGGAACGCTCGGTACACGGCTCTCCTGCAGAATGACGTCCATCATCGTGATATCGTAGCCGATCATGGTGACATTGTCAACGACGAGAAGCACAGTTATGTTTACAATAAAGCTGTACTGCAAGATTCCGATTACCACTACGACGAGAAGATGAAACCCACTTTAAGTAGGACAGACTTTGATATATACGATGCAGAATTCGACGTGGCCACCATAACCGCGTACAGGGAGTGCGCCAAAATGAGTCAAGTGATTCTACAATACAAGCGTGATTTTGAACTTCTGGTGTCGCCTCTACTCGTGTTGAGGGTTGTACAAGTGACCTTGTACTTATGCACATTGCTGTATGCGGCCAGCTTAAAATTCGACATGGTTACAGTCGAATATTTAGCAGCAGTTGCTTTGGATATATACATTTACTGCTATTACGGAAACCAAATTATAATTCAAGCCGATCGCGTCACTTGTGCGGCGTACCAGAGCGCGTGGCCCGCGGCGGGCGCGCGCGCGCGCGGCCTGCTGCTGCGCGTGCTGGCGGCGCACTGCCCGCGCCGGCCCGTCGCCGTCCGGGCCGGGGGCTTCTTGACCATGGACCTGCATACCTTTGTCGTTATTATCAAAACATCGTTCTCCTACTACACACTACTAGTGAACGTCAATGAAAAGTGA

>HrhoOR27

ATGTACAAATGGCTAGCTCTGATCCCACTTTTTAATGGGGCTATATGTATTTGTATTGTTCTTGTTGTAATTAGTAAGGAAATAGATTGGCATTTTGTTACACACATTTTACCGCTCTTCGGCGAAATATTTGTTTACTCTTACTTCGGAGAACAAATCAAAACTAAGGCTAAAAATATTGAACTAGCTCTGTTAAGCTTCGATTGGTGCAACATGAACAAGGAAGATAAAATAAATTATATTATTGTTTTCACTTATATGCAAAAACAATTTGGTATCGAAGTGGCCAGCAACAAAGATTTGTGTATGGTGACCTTGACTGCTGTTCTCAAGCTTACCTACCAGGCCTATACAGTTGTACAAAGTATTGACTTTTAG

>HrhoOR28

TGTGTATTTATCAAAATACATTTTAAGCTGTTGCAAAATGAATTCGAAAGATTTATACCTACTCAGACAAAATCAAGGTGTTATTTGACAGAGAATGAAACAATAAAAAAACGATTTAAACGTTTAGTGAAAAAGCACAAGAAAGTAATAAGTTGTGCTAAATTACTGGACAAAATTCACTCAAACGAGCTTATGCTGAATTTTGTAACTAGTTCATTTCTTTTATGTTTTTCTGCGTTTACCATGTCGATTGCTGAGGATGTGAGGTTTAGAATTTCCTTTTTAACATTCTTACTAGCGGGTCTGGAGCAATTATACCTGTTGTGTTACTTCGGTGACATGGTCATGACGT

>HrhoOR29

GAAACTCTGCAAACGTTTCACAGTATTCTGTCTTTTGCTGGGATCCCAATCTACGCTAAAACCAATTGGGACTCCAAAATTAGACTCACGCATCAAATCTTCAACGTCTTTATTGGTTTCTTGACATTCATTTTCACCACTGTTTTCGTCATTATTAATTACTCTGATCTTCTTCTTTGTATACAAGGAGCGTGTATTTGGACTACCGGAATCATAATGTTCATATCTCTTGGAGTATGCTTAATCTTTAGAAGGAAGTTCCGTATGTTCTTAACAGAAATGGGTTTTAAAGACACGATGCTAGAGATGCCATTGGTTGCTCATGTTATGAGTTTAGAACTTGAAGATGGACAGAAATTGAAAGAATTAAAATTGAAGGTGACAGAATCTCAGGAGAGGCTACTGAAGCTGACGAGGGACCTGCTGAAATTGTATGTGGCAAGCGTTTGGTTATGTGCTACGTTGTACATCTGCAGTCCAATTTACTTTATGATAACTGGAGAAGAGAAGTCCCCTCGTCTTTTGGCTTTCGACATGTGGTTCCCTTGGAGTTTCGATAATTTGAACGTGTATGTAGCTTCGTTCGTGTTCCACGCTTACGCTGGGTATCTTTGTTGTATTGCATATCCAGGCTTGCAATTAACCATAAGCCTTCTAGTGGGACAGATAGTGCGGCAGTTGAAGATAACCAGCTTCATCATGTTCCATCTGGAAGAGATAGCAACGGAATTAAGTAAAGGAAGATTGGGCAATAAGCAAATGTACTGTACGAACATATTGACCCAATGTGTCGACCATTATATCAAAATGAAAAGGTTCAGCAACAACTTGAACGTGATATGCCAGCCTTTCTACTTAGCGTTGATCTTAGTCGCCACGATGTTGGTGTGCGTTTGTTCTGTCAAAATCGCTATATCGGACAAGTTATCGCTGGACACGATGAAGTATTACGTGCACGAGTCGTGTTTTATATTGGTCGTTTACATGTTTTGTTTGTTGGGACAACAGGTTGACAACGAGTGTGAAAATCTTGAGAGAGCCGTTACTGAGAAATGGTATAAATTTAACAAGACGCATAAAATAAATGTAAAAATATTTAAGATGGCTGTCAACCAGAGGATGCCTATTTACATTTTTGGATCTATGAAACTGTCGCTGCCTACTTTTACATGGTTTATTAGAAATGGAATGTCGTTCTTCACACTTGTAATGTCAGTGTTGGAAGACTAA

>HrhoOR30

ATGGAACTTGATTTTGAACGCATCTACAAACTATCAGTGAATTTATTAAAATTTAATCGCTTCTATCCATTTTATAAAATAGATATGAAATGGATAGTTCAAGTATTATTTCTTTATAGCATTTCGCTTTTGATATTCCTCGCTCTAATGCAAAGTTCGGTTTACTACATTAAAATCAATGAATTATCAGACGTATGTGACAATGGGGTCTTCTCATTGGCCTTTCTTGGTTTGACGTTCATGTACGGCACAGTAATATGGCACAAAAATGACCTCATTTATCTAATTGAATCTGTGCAAAAAGATTACGACGAGTCAAAAGATTTATCACAAACAGAAATTAATTTTATACTTGACTACATAGAGAAAGGGAAACGAGTGGTCCACCTTTGGGCTTTCGTGTCTATTTTTAATGTCTTTTTTATACCGTCCAGGATCCTTGTTGTGATGGTATCGGAGGGCAAATATAATTTGGTGGACGTATTGGACTCATTTCATCCAAATATTTTAGAAAAAAGCACATCAGATATCTGGATATTTTTTTTAGAGTTATTGATACGTTTGTATTATTCCATTTACGCAAATATTATGTACGTTGGATTCTCGCCTTTGGGTCCTATTTTCATGGCTCACGCTTGTGGTCAACTCGAAATTGTTATGACGCGGATCAAATCTATTTTCACCGAACGTAACTACGATGAGCGAGAGGCAAAAATTAAGCTTATAGACGTTGTTCAACGAATGCAAAGAATTTACAGATTCGTTGATTCCATTAATAATACATGTGAATTATATTATCAAATAACACTTAATTCTACCTCGCTGATGTTGCCGTTAATTGTGTACATGGTTATTAAGGATTTTCAAATGGACAAAGTTTTCCAGTACATAACATTTATATTTGGAAGTTTTTTGATGACATTCATACCTTGTTCCTACAGCACTTTACTGCTTGCTAAAGGTGATGAAATGAGGGAATCGATTTACATGAGCGGCTGGGAGCGGCACTTGGACAGAGACGCGCGCGCAACTATCATTATTGTGTTAACGCGCGCCTCGCGTCCCATATCTATACACACTTTGTTCAAAACCATCAATCTTGACGCATTCACTGATGTAAATATCGACTTTTTCATAAATCAATCTGCAACGATATTTATTCCCGATCACCGTCCATTGGCAACCCCATCATGA

>HrhoOR31

ATGAAAATTATGAATAATAATATGAATGGGAAATTGGCATTCCTATTACCGTTCCTTCCGTTAAGTGATTCCGAGACGTGGGATAAACTCGATCCGAAATTATATCACGGCGTTCATATATATTGGTTGAAAATTTACGGATTGTGGTATTACAGTTTTTCGCCCAAGACATTCAAGTTTTGGCTTCAACTGGCTTACACGGCGATTGTACTATGGCTGGTGTGTTTTTTGCCGGGCATCGGCGAGATCGTTTATTTGCTGAAGCGACGGGACAACATCGGGGACGTCGCTGGAGGATTGTATTTATTTTTAAGTGAAATGTACACGTATTTCAAGCTAGCTGTGTTCTGGTTAAATAAACAGAAGATAACGAGACTCCTTCAATACTTGTATTGCGACGAGTTCAAACCTAAAGAACTGGAACACAAGGATATTATACTGAAGAGTATTAAAAGAGCTAGATTTGTTATGTCTGCTTATTCTACTATGTGTGTGTGCGCTGTGTCAGTCGGTATTGTGATGCCTTTGACAGAGAATTTTGACGTTTTACCGACAAATGTGGAATACGATCAATTTGACGTTTACAAATCACCGGCATACGGGATACTCTACAGTCATCATATTTATTACAAGCCAGCAACTTGTATTATAGATGGAGTGATGGATACAATATTAGCAGCTTTTGTAGCGTCAGCAATTGGTCAAATTGAGATATTATCATTCAACTTGCGTAACTTCGACAAGCTAGCCTACAGGCTCAGAACCAGGGCTATTATTGCCAAGGAAAATTTATACCCCGACCAGTACTACATACAAGTCACTATGAAGGAGTGCATTAAACATCACAACAGCATAATCAGATACGTTTCAATGATAGAGGAGGCGTTCAGTCTAGCGTCTGCCCTTCAGTTCATGTTGAGCGTCATGGTCCTCTGTTTGGTTGGCATACAATTCTTATCGATCGAGGATCCTAGGAGCCATTTAATGCAAATAGTTTGGATGGCGATATATTTGTGGTGCATGCTCGTCGAAGTGTTTATACTGTGTTGGTTCGGTGACGAACTTATCTGGAAGAGTCAAAGTGTACGTCAAGCAGCTTTTGAAGGGCCGTGGTTGAATGTCGACGTCAAAACTGCTAAATATATCATTATATTCCTGGAGAGAAGCAAACGCCCTCTCCGAGTCACCGCTGGGAAAATTTTCACTCTGTCTCTAGATACTTATACTATTCTTATCAACTGGGCGTACAAAGCGTTCGCGGTTATGAGTAACATGAAGAAGTAA

>HrhoOR32

ATGGAAAACAAAATTAAACCTCTGGATGCATTCAAGATGCTATTCCGGACGTTAACATTCACTGCTTATTTTATGCCTGTACCTGACATAGAGAATCCTGTAAAGAAAAAATGGCACAGATATTACAGGATTGCAACTGTAATAATATTATTAATTTACGATTTACAACATATAACGTTTGTTATTTTGGTTTTCGGCGACGTCGATCGAATGATAGAAGGTCTCAGTGTTCTTCTTACGATTCTCAATGTTACGTACAAACTGATTACAGTCAACCTGAACGAAAAAAGGTTTAACAAATTACACAACGTACTGGAAGATGACATATTTAGTGCTAAATGCCCAAAACACGAGGAACTGATGACCAAGAATAAAGAGGAATTGGACGGCATATCAAAAACAATTAACCGGACTGTCACAGTGATCGCTGTGTGTTGGCTCCTCACACCGTTCCTCAAAAAATTATCTGATGAAGAAGTTATACTACCAGCTTATTTCCCCTTTCCAACTGATGATTGGATCAGTTTTAGTTGTGCATCAATATGGATAACATTTGTAATAATTTGGGTGGGTTACGGCCATATGACCCTCAACATCCTTATTGTCGGATATTACAGTCAAGTCAAAGTGCAACTAAGCATAGTAAGGTACAGTTTGGAACATTTAGCTGATGACGACGAAGGAATACCACACGAGGTTTTCACTTGCAGAAACCATGGCTACAAGGATAATCAAAGCAAGCTGTATCAAGAGAAACTTGTCGCGTTAATTAAAAGATACGACAAAGCTGTATGGTTTTCCAAAGAGATGGAATCAATTATGAATAAGGCTTTGTTGGTTCAGTTCTCGGGTTCCACAGGCATTGTGTGTACTGTTGTCTACAAAATGACCGGTGTAAGTAATCAATATATTATTAAAAACCTATAA

>HrhoOR33

ATGGTGGTGGCATTGTTCTGCTTTATGCCAATAACTTTGATGGCAGTCGATTATTATAAAAATGGCAAGTATAAGGTGAATTTTCCGTTCCTAGTGAAGTACTTTTTCGACCCTTTCACTGAGATATGGCCTTACGTGTACTTTCATCAAGTTGTTTCAACGTTCATAGTATGGGTCAATGTTTATGGTCCAGATACATTTTTCTATGCGTTCTGCGTTTACGTGCAAATGCATTTCCGTATACTGAGTCAAAGATTACGAAAATTGTTTTACAAACCAAACCTTTTAGTTGAAGATAAAGAAAAATTGATCAAACTGTTGAAACGACACCAAGAATTGATTCAATTGGTGAAAGACTTCGAAACCTTATACACCAGCTCCAATTTGTGGAATATGGTCATCAGCTCAATATTGATTTGTCTCAGCGCATTCAATGCTACGACGAACCCCGACGCTAAAGCTGTGCTGACGTTCATCTGTTTCCTCTTCATGAGCCTGTCGCAGATATCGATTCTGTGCTTCTTCGGAGACATGATCGTGAATTCAAGTGCGCTAGTAGCCGAAGCGGCCTACAGTTGTGGATGGTACAACGTTGATGCTGACGTCAAGAAAAGTTTACTAATCGTCATTATGAGAGCTCATACACCATGCAAGTTGACAGCAGCCAACTTCGCCGTACTTAACCTGAGAGCATTCGCTATGATTATTAGCAAATCGTGGTCGTATTTTGCTTTATTAAAAACACTTTACAAATAA

>HrhoOR34

AAAGAAGTCGCTGAAATGATATCAAGATTTTTAGAAACTTTTGAAGATCCAAAAAGGCCGCTATTGGCACCGAATTATTGGATTCTAAATAAAGTAGGTCTGTTACTGCCTGATTCGAAGTTAGGTAAATTTTTCTTCATTATTATTCATGAGATTGCGACATTGTTCGTTCTCACTCAATATATAGAGCTATACGTGATCAGATCAGATTTTGACTTGGTATTAACGAATCTAAGAATATCCATGCTGAGCACCGTCTGTATTGTGAAATCAAACACATTATTGTTAAACCAAAGTAAATGGAAACAAATTATTGATTATGTAACCACCGCTGATATATTTGAAAGAGAGAACAGAGTCCCCGACAAGAAAAACATGTTGGATTCCTGTACCAAATATTGCCGGAGTGTGACCTATTTTTACTGGGTTCTAGTATTTTCCACAGTTATGACAACAACCTCAACGCCGTTGGTGAGGGTCATGTCGTCTTTGTCTTATCGTGACGAATTGAGAAACGGGACTGAGCTGTTTCCCCATATATTCAGTTCCTGGATGCCTTTCGATAAATATCACTCTCCTGGTTTATGGATCACTGTCGGATGGCATATTATCATATGTGTTTATGGTGCGACAATTTTGGGAGCTTACGATACAACAGTTATTGTGATGATGGAATTTTTTGGTATAAAGTTGGAACTTCTAAGAGCGAGGTGCCAGATGATGTTCGGAAAGGATGAATCAGGAATCAGTGACGATAAAGCGACTAAAATAATTCAGCAGCTTCACACAATTCATGTAAAACTTCTTGAGCATTCAAGATTGTTAAACTCGATATTGTCTCCGGTAATGTTTGTTTACGTTATAATATGTGCTTTCATGCTGTGTACCAGTGCTTATCAACTTACGACTGCAACGAGCACCACACAAAAAGTGTTCATGGCTGAATACCTAATTTTCGGTATCGCACAACTCTTCATGTTCTGCTGGCACAGCAATAAATTTCTTGTCAAAAGCCAAGAAGGTATGTTTGGCCCTTACGAAAGTAATTGGTGGGCAGCGGGAATTAAGCAAAAAAAGTTAATATTAATGTTAACCGAGCAATTGAAGTTGGTCCATATTTTTTCAGCGGGACCATTTACTAATTTGACTGTGTCTACGTTTCTTGGGATTTTAAAAGGAGCCTACAGTTACTACACTCTTTTAAGAAAATAA

>HrhoOR35

ATGATGTGCAATGTGTCCACGGATTCGTTTATGGCAGGAATTGTTACTATACTTATCGCTCAAACTAAAGTCCTGAACTACAAATTACAAAATTTAAAAGTTCGCAACGAAAACTTTGTAGAAGAGACTCATGTTCAACATAAAATATTTACTGTAATTTTAAAAAAGTATTTAAAGCATTATGGTCTTATTATGGAATGTTACATAAAAATTCAAGAGATATTAAGTCTAGCAATGTTCGTGCAGTTCACAATGGCTTCAGCAATCATATGCGTCACGTTATGTGGATTGTATTTGGGTCCGACAATGGAAACATTGATATTCCTGGTAACATATTTGATAATTATTATTTTACAAATATTCATACCTTCTTGGCTGGGAACACAATTCAGCCATGAGTGCAATAAACTGGCGGTCGCTGCGTACAATTGCGATTGGATTTCGCAGTCGGAAGACTTTAAGAAAAGCTTAAACCTCTTTATATTGAAGGCCAACGCTACCGTTATTCTCAAAGGATTAAAAATATTTCCTTTGTCGCTTGAAACATTTGTTTCGATTATGAAAACGGCTTACTCGTTTTTTGCTCTTGTACAAAATGTTCAAGCACGTTAA

>HrhoOR36

ATGACATTGCAGTACGACATGGCAAAAATTAAAAAAAAAGAAACTGAAACAAAATTTAAAAGTTTTCACGAAACTTATTCTATGTGTGCGTTTGCGTTAGCAATAGGACTGATGTATCCAAACAGCGAAAATCGACGTAAAAGATTGATATATATGATAATATTCACTACCGCCAACGTTCCTCAACTTTACTGGCTAACGATTAACACGTTGCAAACGTTGAAAGATTCTGATTTCTACAATTTCTCAAGACATATAACAATATCTGTTGTGGTGCTGCTATTTTTATTTAAAACAGTATATGCAATAATCATGTGCGACATGTACAAAAAGTTACTGAATCAAATCACCGATGACATGAACAAAGGAAACGAACTAGATGATAGCTACAAAGCTATATATAAACAATACATAAAAGAAGCAAAATTTGGACAGATCTGTTGGGTTTTTATACCAATTGCAATGAGTTTTCAATTCCCTGCGTATGCAGCAATCTGTACTATTTACGAGAGTATTATATCAGATGTTGGGCCAAAATGCATGATACATAATTTAGATTTAAGTTTCATGGGAGACCAATATAATATCTCACCTTACTTTGAAATTATGTTCGTGTACAACGCAATACAGACTATAGCCCTGGTGCCTAATTTTACAGGGTTCGATGGTTCTTTTTGCATCGTTACCTCCCATTTGAGACTAAACTTAAAACTTCTATCGCATAAACTTAAAAGAATATTCGAAGATTCAAAGAGTAATTTGGAATTGCGCAAAAACATTAAGACTTGTGTAATAGAACACCAAGAAATTTTGAGATTCTATGATGCGATCCAAGTGTTTTATGCACCTTGGCTTATGACAGTATTTCTTTTGACGTCAGTTCTCATTTCTTTCAACTTGTATAGAATGCATCTGGATCAAAAAATCGATTTGAAATATTCATTTTTTGCTTTGTCTGGCGTCATACATATGTTAGCGCCGTGTTATTTTTCAAGCAAATTGATTGAGGCTGGGGAGGAAGTGGCTATAGAGATGTACAGTGTAAAATGGCAACGCTGGAATGATAATAAAGTTACTAAAGTACTGATTTTTATGATAGCCAGAGCCCAAAAGGAGTTTGTGCTTGTAGGGGCAGGAATAATATTATTCAACATGAATTTATTTCTGTCTTTAATGCGTACTTCCTATTCAGTATTCACTTTACTTTGTACGAGGTAA

>HrhoOR37

ATGTTTGATTCAATCAGAAATTATATTGCGTACTTTAAAAACCGTATAAAAGATAACAACTTTGACAGCTTGCTTTGGATAGTGAATGGAGCGCCATCTATAGTTGGATTTAATTTGAGAAAAGACAAAATATGGGCGCCATTTTTCGTCATTCACATGAGCCTTTTAACATATGTGTATGGAGTAGGAAATGTGATGTACCAAGTGAAGTATGCAAAGAACACGGGTGACTTCATCGAGAGCTACGTCAACATCACAATCATGATTCTGGCAGCTGTCAGTGGTTATTGGTTCATCGTGTATAGACCACCGTTACGAGTTATATTAAATTTAGCAGAGGAGAACGATCGCTTATCTAAAACTTCACCAATTGTGAAGAAGAAACGTGAAAAGTTACTTGCAACTATCAAAATTATAGTCTTCATATTTTATGGATGCAACTTAACTAACGCTACTTTCGTTTATCTGCCTCATAGAGTGGACATACTAAGTCATTATGCAATGCAACCTTGTGTAGGTTTGGAACCTCTGACATCATCGCCGAATCGCGAAATATGTCTTACTATATTATGTGCGCAGGAATTATCTATTATGACGGTGGTACTAAATTACCAAGCACTCTGGTTAGTGCTTGTTGCCCATACAGCGGTGATGTATCAGGTTTTGTCAGAAGAAATGTTGATCGTTAACACAGATGAAGAGATTTTAGAAGACAAGCTTCTCTCATTTATCCATCGTCACAATGTAATATTGGACATCACTCACAGATTGAAAGAAATTTACAGTATGCCCATTGGAATGAACTTGGGCGTGAACGCTATATGCATGTGTTTATTCTTCTTCATGCCTCTCTCCGACTGGTTCAAGTTCATGCCCATCTTAGTGTATTGTTTTGTTGTCTTTTTTCTTAATTGTTTCTTATGTCAGAGATTGATTAATGCTTCTGAGGAGTTCGAGAGAGCTGTGTACGGATGCGGTTGGGAGAATTTTGATGTTATCAAAAAGAAATGGGTGTATATAATGCTGATGAACTCCCAGAAACCTGCTCAGTTGCTGGCAGCTGATATAATACCAGTGAACATAGCTACTTTTGCTACTACCATGCAATCGATGTATAAATTTATTACTGTATTTAAACTGTAG

>HrhoOR38

ATGGAAAATACAAATTCACCGGAAAGAAGCGTGATTCAATATGTGCGAAGTTCTCGGGCGTTTCGACAGTTCAAAAATCCACCCCAACCTCACATGTGTATTCAAGACACATTAAAAGATACCACAGAAAAAATGTTTATCAACGTGCTGGGTTGGCAGAAAATAGCAAATCCAAAACAATATTCAGATCCTATACCTCTTTATGGAGGAATGCAGGTACCCCAAGGATGTGGACCAAATAGCAACAAACCACCTTTATTAGTGTTTGCTGTAATGGTTAATCCTGATATATTGAAAGCCAATGGGAAGAATGCAACCAATCCAACGGATCGAGATGCCCTTGTTAACTTGCTTTGCGATTTTGTGGAAGCCATGAACCCTGGATTAGCATTAGCCAGAAAACCAGTTATATTGAGGGATCGAGACTTGGCGGGTGAGCTTAAAGATGTATGGCTTGCTGTTCAGAAAAAGAGAGATAAGGAAAAAGAGGGAAATCAAGAAGTCATGTACAAGGTTTATGATATTGATGGAGTCGGTAATGATGAAGTAAATGATGATGAAAGGCAAGCCAATTTAAGATATAATCAAGGTGATGGAGGCTCACCAACCAAAGTTCAAAACAATAGGAAAAATGCTGTGAAATCATCCAAACAAATTCTCATGAACGCTGGACAAAAGTCAGAATTTGACTTAGGTATGAACAACTGCCAATTGAACCAAAATCATTCTAATAGAGACAATAAATGTGCCACAGACACCACATACTGTACTCCAGTTTACGAACAGATAGTTTCCTACAGTGAAAATCATAATCAAATCAATGATATTCAAGAGAAATTTACTAAGCACGATGCGAACGCGTCCACGTCATTTACAGCCGAATGGGACGCGTTACACGGAAAACCGTCGGATGGATGGGAAGACTTCTCGAAACGTAACATAAACTCGATCAAATCTAACGAAGCCGATACGAGACAGAGGTGTTCAAAGACTGAAAATGGTAAGCTAGTCAACAAGACTCAGTACAATTTTTTTCCAGTATTCAATAAAACCGAAGCAGATAGCAGTATTGATAATGTGAGTAACGAACAAAACTTAAGAATTGAAGAGAAAGCTAAAATAATATTAGACCCAATGCAGAAGTTAGTTTTGCATTCGACTGATAACAAAATATGTGATAATAATTCAAGTGCTCTCAGTTCACTAAGCTCATAG

>HrhoOR39

ATGTCGTCTCTATCATCACATAGAGCCATCAAGTTGTTTAGACGTATGTGTTGTTATGCTTACGTAACGGGATTACCCAATTTTTGGTATGAGAAACCCAATTGGTCTATACATGTTGGTAAATTTCATGATTTCATATCCCACGTCACAGATGTAATAAACTGCGTGTTCTATTTAGGCCAATTCTTTTCGTTCTTCACTCAAAAAAATTTGAACGAAAGACAAGAGACTGATCAAATTATTTTCACGACGATCAATCCGTGCATATATTGGGCTCCTATTGCAATGAATTATTACAAAGAACAAGTGAGAGATTTGATTCGTAATCTAGTACTTGTTCTACCGTCTGTTTACAATGACAGAGAAGTTGAGAGGAAGATGGTGAAGAAATCGTGTTTGTACGTCAGCCTCTTACTGAGCACGGCGAATGCTACCCTGCTGTTATATGGAATTGATAGTTTCATTAAAGTATTAAAAGGAGCAGTTTTTACCACAGTTATCACTGCCTGGCCGAGTGTAGAGGACAGGAGTAGGATAGCTGGCATTGGACGTGCGGCAGTCTTTATTATTTGGACGCAATTCATGTTTCGTTCGTGCGGAGCTATTTCTTTGATAATTAGTCTCACCATCAACACCAGCCACCAGTACATACAACTGCAGAGTTACTTTCGTAATTTAAGTAACATTTTTCAAGAAAATTTGATTCAAGAAGAGATGGAAACAAAATATGAGGAACATCTTAAAATTGGAATACAACAGCATATTAAGATTTTAAGTTACACTAAGAAGTTGAAACAAGCATGCATCTTGGTGTACGGTGGTCAAATATTCACCAATATGGTTATTCTTGTAATGACCATGATGGTTATGATGGGCGATGATCTGTCGTTGACGAAACTGATGACTTTCATGACTTTGGCAATCAGTAGTACTGTTACCAATGGCTTCTATATGTGTACCATCGGGGACATCACGGTCGAGGTACAACGGTATGGGTCTCAGTGA

>HrhoOR40

ATGACTGTCTGGCCTGACTTAAAAGATGAAAGCTTTAAAGCAGGTGCATTTAGAATAGCCACGTATATAGCGTGGTGGATACTGATGTTCCGCCTCTCCGGAGCCATCGTCCTGGTGATGAGTCTTATGACTTACACCAGCTACCAATTTAAACAGCTGCAAAGCTACTTCATCACCCTTGCTAACATTTTCCAGCAAGATTTGAGTCAATTGGAGAAGGAGAGGAAATATGAGGAAGCTTTAAAGATTGGAATAAAGCTTCACGTAGATGTTATTAGTGTCACTCGAAAATTAGTCACGACGTGTAATGTCTCCTATGGAGGAGAAATTATAGTAAATGTGATAGTCATAGCGACTATAATGATTCGTCTAGCGAATGAAGATCGCAATCTCACAAATATATTAGCTAGTGTTCAAATCGCTTTAACAGTACTGGGTATAACCGGTTTCTATATGTGGACTCTTGGAGATATCACTTTAGAGGCTGAT

>HrhoOR41

ATGACAGGTGCTAAGAATGTAAAAAACAGAGTGGAAGCCTGGAATGTGAACAAAAGACAAAACAGAATAAATTGTGCAAACGCCAATCCAGTCCACAGCGAAGCTACCTTTCAAGAGGCTCTACGTGCTACATTAATTATTGGGCAAATATTTTCGTTAATACCCGTTGACAGAGTATCTAGTAAAAACACTGCAAACGTAAAATTCTCTTGGACGTCATGGAAATGTTTCTATTTGGTTCTATCTTTAGGAGGCCAAGTGTTTATGACAGCTATGTGCCTCAACAAATTGTTCGATCGAGACACTTCCCTTAAAGCGACTACATCAGTGATCTTCTACACAATGACAACTGTCACTATGTTGATGTTCTTTCAAATAGCAAGAAACTGGCCTTGTCTCGTTCAGCAAATAGCTAACACTGAACAAATGGATCCTAATTTCGATAGAAACTTGACTTTCAAATGTAACATCACGTGTGCTATCGTTCTCACTTTGGCATTGACGGAACACATTCTCTCGCTGCTGTCTGCGTTCGCCGGCGCTTTGACATGTTACCCAAACATGAGTTTATACGAGGGTTTTGTGAAATATTTTTATCCGTGGGTATTTAATTTTCTGCCATACACAGTTCCATTAGGAATATTAACCCAGTTTTTCCACTTTCAATCGACATTCATATGGAATTTCTCGGATTTGTTCGTGATTAACATGAGTTATTATTTGACGTCACGACTTCAGCAAATTAATAACAAGCTCCTATCGGTGCAAGGGAAGTATCTACCGGAGAGTTTTTGGAGGGTGACTCGTGAAGATTACAGTCGAGCCACCCAATTGGTGAGGAGGGTTGATGACGTCATCAGCGGAATCGTCTTTATTTCCTTTGCCAACAATCTGTTTTTCATTTGTCTACAGCTTTTTAACACTTTGGAAGATGGTATTCAGAGAACTGAAGCATGCCGACGTCGTCTAGGACGTACTGGACCCTTGGGAGGTCATGAAGCCGAGACGTACTTCCTGTTCTCCCTCGGCTACCTGATCGCACGGTCGGTCGCCGTGTCTCTCATTGCATCCCAAATCAACATGGCTGCGTCTGTGCCAGCACCAGTGTTGTATGACGTACCGTCGCCTGTGTACTGCATTGAGGTGCAAAGATTCGTGGATCAAGTGAACGGTGGCAGCGTCGCTTTGAGTGGACTCCAGTTTTTCAACGTTACTAGAGGGTTACTGTTATCTGTGGCGGGAACAATAGTAACGTATGAATTGGTGATGTTTCAATTCAACTCGTCGGGTAATTCTAACTCAACTGAAGTCGCAGCTAACGCAACAATCTCATGA

>HrhoOR42

ATGTTCATTGATGGTGAACTGAAAGATGGTTCTGAACAACTTGAGCAGTTGAATAAGAAGAAAAAAACTAAGAGTAATGCAAAAGGCACTAACCAGCAAGAGGCACCTAAGTCAATGTTAGTGTCATTATTTGTGCCTTTTGAAAATTCGATGCCTGAAAGCGTCGAATACATGGAATGCGATGGCAGTATACACTTCAGTGGAGTAGTTTCGTCGAGCGTGTTCATGTATCCGAAGGCGACTGTTAACGAAGCGATTGCGTCTGTCAAACAAGATATCGTCAGGTCTCTGGCCTCTAGATTCACTATGCACTGTGATGCTTTAATAGACGATAATTTATTGCCTGAAGAGAAGGTGTGCTTCAACGAGCCGCCGCGGCGCGTGCTGGTGCCGGTGGGCGCGCTGCACCTGTGCGACTACCTGTTCCCGGGCGAGGCGCCGGCCGAGGCGCTGCTCTCCGTGCGCGAGCTGCTGGACCTGCACATCACCGAGGCGGACGTCGTCTGCGACATCGAGACACCCGCTGACACATCCGAGTTCGACGCGCTGGACCGCGACACTAGCAGCGAGGAACTCCTCGCGTCGCCGCAGGAAGCGAGCCAGTTCATGTACATCACTGGGATATGCTTCGCGATGCTTGTCTTATTTATATCTATTATAATACATTATTACGACGGTATAACTAAGTTTATAAGTGGAATGTTTAGTAAGGCAACTTAA

>HrhoOR43

ATGCCGCGTTCGCAATCAATAAGAAACACAGAATGGTTCAATGTAACGTTCATATTGATGGCCGCTGTCGGCATATGGGAACCTCCATGTTCAGAAAATAAAATAATAGTTAAATATCTGTATTTAATATATAGATTGATATTTTTATCTTTGTTCGCATTTGCTATAATATCTATGCAACTATTTTTGTTCTTTTTGGTTTTAGGTGATATGGACGCGCTTATCGAAGCTAGCGTTTTGTTTTTTTGCAATATCATTCACGGAATAAAAATGATAACGATCATTATACAAAGGAAAAGAATAAAAAGTTTATTGACTATCGTCGATGATGATGTGGACAATCACAAAGTTTACGAGAATTTAGGTAAACGCGCTGGATTTATGTCGAATATGTTCTACTTAAACGTCGCAGCTACTGGAATCTTGTGGAGTATTTATCCTATGACGAAATCGGAACTGAAACTTCCTTACTCCTGTCCTCTCATCTCCAAAGATTCCTATTGGTTCACATATTTTTACGTCTACG

**IRs**

>HrhoIR8a

ATGGATTTCTTTGTATTATTCTTCATACTTATGATTGTAAATGTAATATTTGTGACTTCAGAAATCAGTTTGCGGTTCGTATTTATCCTGGAGCATCATGATTACGAGCTAGCGGAGCAGATTGGCAACGCCCTAAAGACTGTCGAGGAGTCCACGCCCGGAGTTCATCTATCAGACGCTGTGATATTCCTTAACAGAGAAGAAGACGGCGAGAGTTACAGAAAACTTTGCTCCTCTGTATCTACTGGCGTCTCTATGATCATAAACCTCTCTTGGGCCCCTTGGCCGGCCGCTGAAGACATGGCCTCATCATCAGGAGTCCCGATAATCCACACAGCACTAGGATCGCAGCAGTTAATAAAAGCCTTAGATGATTATCTGGAAAGCAGGAACGCAAGTGACGCGGCCTACATATTGGAGAGCGAGAAGGATGTGGACAAAACGCTGTACGAACTATTGGGTAGATCCAACGTCAGGGTTTGGGTGCACGCTGGTCTAACAAGAGACTCGGCAAATGTCTTGAAGAGCATGAGGCCAGAGCCTAGTTTTCACGTGGTGGTTGGGAATAAAGGGTTCATTATGGATACATATAGAAGAGCTGTGAAAGAGAAGCTGGTGCGTCGGAAATACCGCTGGAATCTAGTCTTTACCGACTATTCCGGGGCTGATATGGACTGGTCCCAGGTGACTCTACCAGCTATGGTCTTGTACATCAATCCAGATGAATGCTGCAAGTTGATGAAGCAAGAAAAATGTACCTGTCCTCCGGATTTTCAGAGAACGCAATCTATGTTATCATATCTAATTGAATATATAGTAACGAGTTATGGGAAACTTGAAGATCAGCAATTCACGACCAAACTCGACTGCAACGCTATAGAAATGGGAGACATGAATGTGACTAAAGAGAAGTTGCTGGACTTTTTCAATCAGGATTCAACAAATAACGACTCATTGTTCTATTGGAATATTGAAAGGTCTGGTCTCTTTCTTCGATCTCGCTTCGTTCTATCGTACTCTGATGGATCAGAACGCTTGGAGCTGGTGGCTAAGTGGTCAGCGGACGAGGAATATAAATTGTTACCGGGTGTGACCTTGGAACCTCTGAGAATGTTCTTTAGAATTGGAACATCAGCTGCTATACCATGGACGTTGCACAAAATGGACCCAAATGGGCAGCCAATGGTCACGGATGAAGGTGATCCTGTGTACGAGGGTTACTGCATCGACTTGATACAAAAGCTATCGGAGGTGATGGAGTTCGATTATGAAATCGTTACCCCCAAATCTGGATCTTTTGGTAGAAAACTGCCAAACGGCACGTGGGATGGCCTCATCGGAGACTTGGCTCGAGGGGAAACGGAAATAGCTGTAGCAGCTTTAACCATGACAGCTGAGAGAGAAGAGGTCATAGATTTCGTCGCCCCATACTTTGATCAAACAGGAATTATAATTGTGATCCGTAAACCAATTCGAAAGACCTCACTCTTTAAATTCATGACCGTGCTCCGCACTGAAGTGTGGTTGAGCATCATAGCGGCGTTGATATTGACAGGCTTCATGCTGTGGCTGCTCGACAAATACTCACCGTACTCTGCCAGGAACAACCCTGACGCGTATCCTTACCCTTGTAGAGAGTTCACCCTTAAGGAGAGTTTTTGGTTTGCACTAACGTCGTTCACCCCTCAAGGAGGGGGCGAAGCACCCAAAGCTTTGTCAGGGCGAACGCTCGTGGCTGCCTACTGGCTCTTCGTGGTGCTTATGTTGGCTACGTTCACAGCTAATTTGGCCGCGTTCTTGACAGTCGAGAGGATGCAGACTCCAGTGTCTTCGCTGGAGCAGTTGGCGAGACAGTCTCGTATCAACTACACGGTGGTGGAAGGCTCTTCCATACATCAATACTTCATTAATATGAAGTTCGCTGAGGACACCCTGTACAGGGTATGGAAGGAGATTACGCTGAACGCTACCTCAGACCAAGCCCAGTACAGAGTATGGGACTATCCGATTAGAGAACAGTATGGACACATATTACTTGCTATTAACGCTTCAGGACCTGTGCCAGACGCAAGAACAGGGTTTCGTCAAGTGGAAGAGCATTTGGACGCGGACTTCGCCTTCATTCACGACTCAGCTGAGATCAAATATGAAGTAACTAAGAATTGCAACTTGACGGAGGTGGGCGAGGTGTTCGCCGAACAGCCCTACGCAATAGCCGTGCAGCAGGGCTCCAGGTTGCAGGAGGACCTGACCAGGGCGTTGCTCGACTTGCAGAAGGAGAGGTTCTTTGAGCAACTTAACTCTAAATATTGGAACGAATCACTCCGCCAGTCGTGTCCTGATGCCGACGAATCTGAAGGAATCACACTGGAGAGTCTGGGTGGAGTTTTCATAGCGACGCTCTTCGGGCTGGGCCTGTCGATGATCACCCTCGCCTGGGAGGTGTTCTACTACAAGAGGAAACAGAAGACCAATGTGCACGATACAACGACCAGCGAGAAACCGAGGCCAGCTTTCGTGAAGAAGGGCAAACTGCGTCGCAGGAAGAAGACGGTGACAATCGGCGACAGTTTCAAGCCAGCCGTCGATGTGTCACATATTACCGTTTATCCCAAAGGCTACGTCCCTTGA

>HrhoIR21a

ATGTTTTGGTATGTCTTTGGAACATTTACCAACTGTTTCACTTTTGTTGGTAAAAATTCGTGGGGCAAAACCACGAAGAATACAACTAGATTGTTAATAGGGTGGTACTGGGTGTTTACAATAATCATCACAAGCTGTTACACTGGTTCTATAATTGCGTTTGTGACTCTACCCATTTTTCCGGCGACCGTTGATTCAATCAAACAATTGTTGTCTGGATTTTACCGGATTGGAACTTTAGACCGTGGTGGCTGGGAGCGATGGTTCATTAACTCCTCCGATCCAGACACTAATAAACTATTTAAGAAAATCGAGTTGGTATCTACAATACAAGCTGGTATAAAAAATACTACTAAAGCCTTTTTCTTCCCGTACGCCTTTTTGGGGTCTCAGGCTGAGTTGGAATATATAGTTCAAGCTAATTATACAAAAACTCGATCCAAACGAGCTGTTCTTCACATATCAAACGAATGTTTCGTGCCTTTTGGAGTTGCGTTGAGTTTCCCAAACAATTCTATTTACACATCAAGATTCAGCAACGATATTAGGAGGGTGCTTCAAAGTGGCATTTTAAAGAAAATCGTTAGTGATGTTAAATGGGAAATGCAGAGGAGTTCTTCAGGAAAGTTGTTATCGGTTGGAAGCGCTATATTAAAATCTACCTCAATTGAAGAAAAAGGTTTGACACTTGAAGACACTCAAGGAATGTTTCTTCTATTGGGTGCAGGGTTCCTAATTGCTGCGGGAGCTTTGTTATCAGAGTGGATGGGTGGCATATCCAGAAGATGTTGCGTGATAAAGAAAAAAGCAACAAGTGCTAATTCAAGTAGAAATCTAATGACCCCATTTAACACAGAGAATGAAGTAAAAGATGATACAGATGGTTTTGATTTAGAGAGGAAATCTGATTTAGATTCTAATAATTCTAGTGCCGATTCGCGGAAGACATTGGATGGTCACATTATAAAGTTGACAGAAAACAGCATAACTGTTCATGAAAATTTTAATTCGAACGACTGGAATTGTAGGAGATCAAGTTCAGTGGACATAGATCAAGAAGTTAAAGAGATATTTGAGAAGGATATCAGTAGACGACGTAAAGCTTTTGATGATAGAAGTAGTTTACAAGATGGGAGATTAACAACCGCTTCTAAAGGAAAATTTGGTGAATACATACCATGA

>HrhoIR76b

ATGGTAGCGAACGGCCGGGCGGTGTTCCGCTCTTTTACATCTGATCGAGACTTCTTACCGACGGTCAAGGCTGGGGCAGTTTTAGTTAAAGAACAAACAGCGGTTGATCACCTTATGTACTTCGACTACCTGACCAAGGTCAGGGAAGGGGTAGTGGAGGAAGAGAGATGTACCTACGTGGTCGCTCCGAACGCTTTTATGAAACGAACCAGAGCGTTCGCTTTTCCCATGAATACCAATCTCACTACTTTATTTGATCCCATCTTGACTTACCTGTTACAATCAGGAATAGTTGATTTCTTAGAGCACCGAGATCTCCCTACTACAAAGATTTGCCCGCTAGACCTTCAGTCGAAGGACCGTAGACTGAGGAATAGTGATTTAATGATGACGTACATGATCATGGGCGTGGGGCTCGCATCAGCTATAGCAGTGTTTATTATCGAGATGATTCTCAAGCGCTACGCAGTCAAGCATAAGCTGAAACCGTTAAAGAAGTTTAACAGCAAGACATTCACTTTCAAAGACGACAGTATGCCGCCCCCTTACGACTCATTGTTCGGGAAGAACTCAAAGTACCGCGGTTCCAAGAGAACTGTTGTCAACGGTAGAGAGTACTGGGAGACGAAAATGAAGGACGGTACCACTAGGTTGATCCCTCTAAGGACACCCTCGGCCTTACTGTATCAATAG

>HrhoIR41a

ATGCTGACGTCCAAAATGTTATCTATTCCAATCGAAATACTTTTGAAAATCATACTCCAAAAGTATTTTATCAATTCATATTGTATAACTGTTGTTTCTGAAGATTCGATACAACTAAAAACATCGATTCCTTTTATTTATGCGATACCAAATGATAACTTTGTAGATTTGTTATTAAATTCATCAGACATAGGGTGCTCCGATTATATTGTTAATATGAAGAATCCTCAAGAATTTATGAAAGCATTTGAAAAAGTTACCCATTTAGGACTACTGAGGAAAAGCGACCGTAAAATATTAATTCTGACACATTCTAAAAGTTATAATGCCCAAGACAAAGATGCTATTTTGAAAGTTCTGTCAATGAATGAAACTAGATTTGTTGCAAATATTTTGCTGGTAATACAAGCTGATGTGAATGAAAAATGTTATATTTATGATTTGATCACCCACCAGTACGTTGGTAAAGATGACGTTAGAAAACCTATATATTTGAATCAATGGAATTCTTGTACCGGCTTCACGAATAATGTTAGTCTCTTTCCCCATTATAACATGTCCGACTTGTATGGGAAAACACTAAAACTAGCCTGTTTCAATTATGAACCGTATTCACTGCTGGATCTCGACACAAGTGTAGATCCTTTGGGACGTGATGGCATGGAAGTAAGAGTTATGGATGAGTTTTGCAGATGGGTAAATTGTACTATAGAATTAGTGAGAGATGACAACCAATGGGGAGAGATATACTCCTATGAAAATTTGACTGGAGTTGGAGTTATTGGCAACGTTGTGAAGGATGAAGCTGATGTAGGGATAAGTGCACTCTACTCTTGGTATGAGGAGTATATAGCTTTAGATTTTTCAACTCCACTTGTAAGAACTGCTGTTACATGTATTGCTCCGGCTGCAAGAGTACTAGCGAGTTGGGAACTGCCATTACTCCCATTTAGTTTGCATATGTGGCTGGGTTTAGGATTTACTTTTTTTTATGCATCTATGGCGCTTATGATAGCAAAGGGTTTTAATACGGATAAAATGTTTTTGACTACTTTTGGCATGATGATAACGCAGGTAAGGTTTTTAAATTCATGTAAAAAGTCAGGTGGATATCATTCACCTCATTGA

>HrhoIR60a

ATGACTATGGTTGTCTTTCTTTTTATTTCACTATTAATAAACGAAGTCAGTTTAATCAATCCAAATGGACCTACGGCAGTGGAAGATTTTACTAATTGTATAACTAGTATAGTGAAAGTAAGCTTCAAGAACCCTGGATTACTCGTTTTTGTGGATACATTTTTTATAGCCGAAGCTGTAGGAAGAATCAAAGGAAATGTACTGAAGCAAATTCATTTAAACAAAAAATTTTCAGTAAGAGTAGTGCGTCCAAAAAATGAATATCCTGTTTGTGTTAATTTAAATGAATTTAATACCGGTGTTGTACACCAGAATCAGGTCGATGTTATCCCTTTGGCTGATTACTTTGTGATCATAGTTGACAGTTACAGTGAGTTTACTCACGCAGCAAGTCGACTGATTAGATTACGAAATTGGAATCCACATGGCAAATTTCTCATCTTGTTGTATAGTTTCGATAACATTTATTATTTAAAACAGATAGAATATATATTCACTTGTTTGTTTCGCTATAATGTATTAAATGTTGTGGTTCTCGTGCCACATATTCGGAATATTCGTGCTACAATAATTTATACCTGGGAGCCATTCGAACCCCCGAAATATTGCGGGTATTATAATGAAACCGCAGAAAATAGGATAAAAGTCGCGGATTTTTGTGAAAAGGGCCATTTGAAGAATAACACGACACTGTTTGAAAACGTGGTCCCAATTGACATGATGTCATGTGTTGTAAATATTCTTGCTATAGAAAAACAGCCGTTTATTGGTAAAGACGACAATGTGCAGGAAGCTAATATTGAACGATTTCTTATCAATGAAGTTCTTAGTACAATTAATATGAAAACTAATTATATCATCACAAATAAATCAAGAGGTGAAAGGTTTTATAATGAATGGAATGGTGCACTTAAGAAAATAGTTTCAAAGAAATTCAATGTATTACTTGGAGGTATATTCCCAGATTTTGATGTGCATGAAGATTTTCAATGTAGTAATACATATTTAGAAGATTCTTACACTTGGGTTGTGCCACGCGCTCATCCACGCCCTCCATGGGTAGCGCTGACGATCATATTTCACAAAACCGTCTGGCTGTCTGTTCTCATCGGTTTCACTATTAGTGCATTATCATGGAAATTTTTAAGTACAGTCAGTGGCGATTCCACTTATTACACAAGTATCGACCACTGCTTATTGAGTACTTGGCTATGTATATTAGGATTAACTACACATATTCGTCCAAAGAAAGAAAGTTTACGAATCTTTTTTGTATTTTTTAACATCTACTGCATAATATTTATAACAGCCTATCAAACAAAGTTGTTTGACGTTTTAACTAATCCGTCTTTTGAATATCAAATAGCTAATGTCGAAGAATTAATTGATAGCGGCCTAAAGTTTGGCGGGTTCGAGGAATTACATGACTTGTTTTACAATTCAAGTGACCCATTTGATAATTTAATAGGATCCCAATGGGTTATAGTGGAGAACATGAGCAATGCAATGGTGGATGTTGTTGTGCATAGAAATTTCTCTGTACTGTGCAGTCGTTTGGAACTAACCTATTTGTCTGCAACAATGCCTCAACTCAGTGATAGTATCGGCCACCACAAATACTACGCATTTAAAACAAACGTTTTCACGGTGCCTATAGAACTTATTGCTATGAGAGGATACGCGTTAGTAGAAAAATTCTCAGAAATTTTGGAAGCATTCAAGCAGAGTGGTATAGTTTCTGGAATAAGAAGGCATTACGTAACATTCGCCGAGAGAAAACGGGCTTCAATTATACTTAAATTACAATCACAACAAAATGATGTGAGAGCATTAACTATTCAACATTTACAAGGAGGTTTTCTTGCTCTCGTTTTGGGTTACGTAGGGGGAATAATTGTGTTCATTGTGGAACTTATAATTAAATGTAATCTTGTTCAGAAAGTACTGTATCAGAAGTGA

>HrhoIR75q.2

ATGAAGGCTGTAAATTTGTTGGCATTGTTTATAATTACAGTTATTAGTTGCCTAGCGGAAGCTGATTTGGTATCTATTATCGGTGATTTGATTCGTGTTATGAATAAGCCATCATCCGTAATAGCCACGCTGTGTTGGCCTCAGTACAAGCAACTTAAGTTGTATTACTTCCTTCACCGTGAAAATATATCTCATTTAACAACGATTCAGTTTTTAAAATTAGGACATGAACCAAAAAATTATTGGCCCTCTCAAAATATACTCTTTTTGTTGGACCTTAACTGTACCAATGTTACAAATCATCTAAAATTGAGTAATGATAAAAATCTTTTTCGAAGTCCTTACCGCTGGTTTTTAATAGGTATTGATGAAACTTTAAACAATTCAAACAGTAATATTAATATAAAAAGACAATTCAAACCTTTTGATATCTTTCCGGATTCTGAAGTCATGATAATATTGTTTCACTACAACAGCAGAAACGATTCACGTTATGAAGTTATTGACATTTATAAAACATGTAAAAATAGTGAAGATATGAAAACAAAACTTTACGGAAATTGGGATGCGACAAATAGATTCCAAAAAAGCTTAAATTTTTATAAGCCAACAGCTTTACAGAGACTTGATTTAGGTGGATGCGAAATTGCTATTTCCTACGTTCTCACAAACAATAACAGCATACATCATTTATATGATCAAATGGATGATCACGTCGATACCATCACCAAAGTTAATTTTCCAACAACGAATCATTTGTTAGAATTTCTCAATGCCACTAGAAAATATAGTTTTACTGACACATGGGGATATAGGTTGAATGGAACATGGAATGGCATGTCTGGCTACCTTTTTCGCGGAGAAGTTGAAATTGGAGGCTCTCCAATGTTTGTTACTTCAGAAAGAATATCATTTGTAGAATATATTTCTAACCCTACACCAACTAGTTCAAAGTTTGTGTTTCAACAACCTAAGCTCTCATATGGAAACAATATATTCCTCCTTTCATTTCGTGAAACTGTTTGGTATTGTGCCACCGCTCTTGTAATTTTGATATTTCTAACACTGTTCGCTGTTACATTTTGGGAGTGGAAGAAAATTAATGATGGAAATATGTTGGATAACAGAGATCCAGGCATTCTACGTCCAAATGTTACTGATATTTTAATACTAATTGTTGGAGCTCTATGTCAACAAGGATCGCCTGTGCAATTGAAAGGTTCTTTGGGTCGCATTGTCTTACTAGTTTTGTTCCTAGCTCTCATGTTCCTTTATACTTCTTATTCGGCCAATATAGTAGCATTACTTCAATCGAGCTCATCACAAATTAAGACGCTTGAAGATTTGCTTCATTCAAGAATGAAATTTGGTGTAGATGATACAGTGTATAGCAGATATTATTTTTCGATAGCGACTGAACCAATAAGAAAGGCAATTTACGAAACAAAAGTAGCACCACGCGGTGAGAAACCTCGTTTCATGTCTATGGAGGAAGGGATCAAGAATATGCAAAAGGGACTCTTTGCATTTCACATGGAAGTCGGCGTTGGTTATAAGTTCGTTGGGAAATATTTTCTCGAAGGTGAAAAGTGTGGACTGAAGGAAATTCCTTATTTACAAGTTCAAGATCCGTGGTTAGCCGTCAGAAAAAACACTCCTTACAAGGAAATGTTTAAAATCGGGTAA

>HrhoIR68a

ATGAACTTCAAATCTAAATTATATAAACCCGTTAATGCGGATATTAACAAATGGGGGCAGAAGCAGTCGAACGGGTCATTTTCAGGACTTATAGGTGAAATGGTACGGGGTAAAGCTGATGTTGGTTTAGGAAATCTGCAATACACGCCGTATCATTTAGACCTAATGGACTTGAGCATTCCATACACGTCGCAATGTTGGACTTTTCTTACACCAGAAGCATTGAGTGATAATTCTTGGAAGACTCTAATTCTGCCTTTCAAATTGTACATGTGGATCACCGTTCTGTTGGTGTTGTTGGTCACTGGATCTATATTTTATGGTTTAGCAAAGTTTTATTTGAATCTACTGGAATTTAAAGATCATTCCTCAATTTATGATAAACATTTAGACAAACAAATTGTTTACGATGGTGCGAAACCGGTCGGCTTGTACTTGTTTGGCGAAATAATTAACAGTATCCTGTACACGTATGGAATGTTGCTCGTTGTCTCGCTACCGAAGCTGCCAACCGGATGGTCAATCAGACTTCTAACGGGCTGGTACTGGCTGTACTGCATCCTTTTAGTGGTCTCATACCGAGCAAGCATGACCGCCATCCTGGCGAACCCCGCACCGAGAGTGACCCTTGACACTCTCAAAGAACTCGTTGAAAGCAAGATCGCATGCGGTGGCTGGGGGACCCAAACGAAAATCTTCTTCGAAGAGTCTCTAGACGAGATAGGAGAAAAGATCGGTGAAAAATTCCAAATAGTTAACGATCCGGATGAAGCAGCTGCAAAAGTAGCTCAAGGAGTATTCGCATATTACGAAAATAAACATTTTCTTAAGTATCTAAGTGTGAAACGAAAAAACTCTCTTATAGACACGACTCCACAAGATAATTCTACGGCAAATAGTACAGCAGTGACAGCGAAAAAAAAAGATGAACGAAATCTGCACATTATGAGTGATTGCGTCATAAACATACCTATTTCACTTGGTTTTCAAAAGAACTCTCCTTTAAAACCTCTCGCCGATTTATACTTGAGTAGAATCGTGGAAGTAGGATTAGTAGAGAAGTGGCTCAATGACGCCATGCATCCGATAAAGTCATTAGACTCCCAAGAAGAAGAAATTAAGGCTCTTATGAATCTCAAAAAACTATATGGTGCATTTATAGCATTGGCTATTGGATATTTTTTAAGCACAATTAGCCTGATTGGAGAATTCATTCATTGGTACTTAATAGTAAAAAAAGACCCGAATTTCGACAAATATGCTTTAGATGTTTATTATGCAAACAAAAATAAAAGGCAATGA

>HrhoIR75p

ATGAAATTGTATAATTTGTTATACATTTTGTTGTGTATTCCAACAGATGTAGCGTGTTCTAGGAATGTTGACGATATGCTCCTATCGTATATAACTATGGAGAACCAACCAACGTCACTGCTTGCCCCCGAACTGTGTTGGCCGTTGCATCACAAAACCTCATTCACGAGACTATTGAACGGCGTTGGAGTCAACGTAGCATACACTATGCGACCCAGCAGAAAGGAACAGTACCTACATCACATCACTATACTGGCAGACTTTTCCTGCTCTAGCGCATCGGATTTAGTTTTACAGAGTGACGAACATGGCTTCTTCATGTCACCATACAGATGGATTTTCATAAACTTGCATCAACAATCACCGAATGCTACTATATTGGATAAATTAAATATATTGATCGATAGCAATGTCGTTGTTGTACAAAAAGTTGATGATACTAAATATGTGTTTCATGAAGTGTATAAAATTGCGAAGGATTATCAAGTTATAAAAAATCTACGAGCCGTGTGGCGAGCAGTCAACGATACTGAAAAACGAAACGAACAAACTACGAGCAGCATTCACGTCAATTTGACTCCAGGAAACAATAGTGTTTCAGTTAACAGCAAGACGAATGGTGAAATTGAGGATTTGTTTTATTCTACGCCGCTATCTTGCAGAAGAAGAAATCTCAGAGGTCACAGTTTAACAATGGTCAATGTTATAACAGACAGTAATGAAACAAAAAATCATATGCATGATCGACTGTTTCTCCATCACGATTCTATTTCAAAAATGTCTTACATGGTTGTAAGGATTTGTTTTGAAATGATGAACGCTTCAGAGAATCTTTTATTCACAAACACTTGGGGTTACCGAGATAAACACGGTAACTGGCAAGGCCTGATTGATCATTTGTTAAAGAAAAAAGCTGATTTAGGCACATTGACAATATTCACCAAAGAACGTACAGAGCACATTGATTACATTGCAATGGTTGGATCCACTGCAGTCAGGTTCGTGTTCCGAGAGCCCCCGCTGGCCTACGTCTCAAACATTTTCACCCTGCCTTTCTCCGGAGCGGTGTGGTTTGCAATACTAATATGTGTGCTGGGATGTGCACTGTTTCTGTACATCACATCCAAATGGGAAGCTACAATGGGATCGCATCCTCTTCAACTAGATGGCTCTTGGGCTGATGTACTAATTTTGATTATCGGTGCCGTTCTACAACAAGGCTGCACTCTCGAACCGAGGTTTACAGCAGGTCGTTGTGTCACGCTATTGCTGTTCATCAGCCTCACCATTCTGTATGCAGCTTATTCAGCCAATATAGTAGTACTTTTGAGGGCGCCAAGCCCCTCAGTGAGAAGTCTCCAAGATTTATTGAGCTCACCACTAAAGTTGGGTGCCAGTGATTTTGAATACAACAGATATTTTTTCAGGCAATTAAACGATCCCACCAGGAAATCAATTTACGACAAAAAAATTGCGCCTAAAGGAAAGAAGCCGAACTTCTATAACATGACAGAAGGTGTCGAAAAAATTAGACAAGGATTATTTGCGTTTCACATGGAGCTAAACCCTGGCTACCGTCTTATCCAGGAGACGTATCAAGAAGACGAGAAATGTGATCTAGTTGAAATTGATTACATAAATGAAATCGATCCTTGGCTTCCGGGACAAAAGAGATCACCGTACAAAGATTTGTTTAAAGTCAAGTGA

>HrhoIR40a

ATGCGAGTCGCTGTTGTTACAAACCCGAGGGAGAGTGTGTTTCGTATCTACTACAACCAAGGAACCCCGAATCTGTTGCACCATTTGACCCTCGTGAACTGGTGGTCAGGTCGCCTGTACAGGTCTCCTGTGTTGCCCCCAGCTGAAAAAGTATACAAAGATTTCAGAGGGAGAGAATTTGAGATTCCTGTTTTACACGCTCCACCTTGGCACTTCGTGAAATACAACAATGATTCCACAGTCAACGTTACGGGGGGGCGGGACGACAAATTGCTGTCATTACTCGCAAAGAAATTGAACTTCAGGTACAAATACTACGATCCCCCGGAAAGAAGTCAAGGTTCGAGCATCTCTGGTAATGGAACATTCAAAGGCACCTTAGGGCTGATTTGGAAACGTAAAGCGCCATTTTTTATCGGAGACATGACAATGACGTGGGAACGGCTTCAAGCGGTGGAGTTTTCTTTTCTGACTCTTGCCGACTCTGGCGCCTTCCTGACGCACGCGCCAGCGAAGTTGAGTGAAACACTAGCTATAATAAGACCATTCCGATGGGAAGTATGGCCTCTAGTGCTCGCCACTTTGCTAGTAACTGGACCAGCGTTATGGATGGTAATAGCAGCTCCATCTTTATGGAGACGTCAACGTCGGGATCAACTCCAACTTTTTAATAATTGCTGCTGGTTTACAACTTCTCTTTTTTTACGACAATCTTCTAGCAAAGAACCGTCTAGCACTCACAAAGCTCGCTTGGTGTCTGTAGTGATATCTCTTGGTGCCACTTATGTGATTGGAGACATGTATTCCGCAAATTTAACCAGCTTGTTGGCAAGACCAGCAAGAGAGCAACCCATTGGAACACTACAAGCTTTGGAAGAAGCAATGAGGGACAACGGATATGAATTGGTGGTAGAAAGACATAGTTCTTCTCTGACTATACTCCAGAATGGGACAGGTGTATACGGCCGTCTTGCGAAACTGATGAAACGACAGCAGGTGCAACGAGTGAGAAACGTGGAGGTCGGCGTGCGTCTTGTTTTAACACGGAAACGTGTCGCCATACTCGGTGGCCGGGAAACATTGTATTACGATACAGAGAAATTTGGTTCTCACAATTTTCATTTAAGTGAAAAATTGTACACTAGATACTCCGCTATAGCTATGCAAATTGGATGTCCTTATTTAGAAACCTTTAATAATGTAGTAATGACACTATTCGAGGCTGGTATCCTGACTAAGATGACAACAGACGAGTACAGGGATCTACCAAAACTCTCTAGGAGATCTGATCCCGTGACTGAAAGTGACACAGAAGGCAGCGATGCTATAGGTGAAAGTACTGCAGCTTCGCAGACCCAAGTAGAGTCTACTAAAGGATTGGAGCCCGTTACATTGAGGATGCTGCGTGGAGCGTTTTGTTTGCTAGGAATAGGATATTTGTTGGCCGGAGTCAGCTTTTGCATCGAAATCCAAATACATCGACGAAGAACACGTACAAAGGCACCGGTACCCGAAACTAAAATCAAAGAGAAGCAGAAAAAGTTTCAAAGAATTCTAACGAACATCAAAATAAGATTTCGAAGAATCGCCATAAAAATATACAGTAAAATTGATACAGCTTTAGGTCCTTAA

**GRs**

>HrhoGR2

ATGCCCATTACTAGGTCACGTCCAGGTACAATTACATTTAGTTGGAAATCAAGAGCCACGGCATACGCAATTTTCTTTTATATTGCTAGTACTGCTGTAGTACTGGTAGTGGGTTATGAACGAATAATGATCCTCCGTTCAATCAAGAAATTCGATGATTACATCTACGCTATCCTGTTCGTTGTGTTTCTTATCCCTCACTTCTGGATCCCGTTCGTAGGTTGGGGGGTGGCACATGAAGTTGCCATTTATAAAACAAACTGGGGGAAGTTTCAGGTAAGGTACTACCGGGTCACCGGGGAGAACCTTCAGTTTCCAAACTTGAAGAATCTAATAGTCGTCATTAGCGTGGGTTGTTTACTTCTCGCAGTGTGTTTTTTGCTCAGCCTCTGTGCACTGTTGGATGGATTTCTGCTGCGTCACACAGCCGCTTACTATCATATAATAACAATGATCAATATGAATTGTGCCCTCTGGTATATAAACTGCAAAGCTATAAAAATCGCATCACAAAGTCTATCGTTATGTTTTCGTAAGGTTCGCAAATATAACAATAGCTGTGTACGGCGCCGTCTCAGAGATTGTGGACCACGGGTTTGGATTCAGCTTTAA

>HrhoGR3

ATGATAACATTCGCAAATATGTCAGCACGAATTATTATTGAAGCTATGGGATCCGAAACTGGTTTCATATTCGGCGTGTTAGAGTTTACTGCGTTATCCAGTTTAATGCGGCTGGGCACCTGCGCTGCGGTGATGATCTGTGTAGTTAGTTACTGTGAACGAGTGTATCGGCAGAGGGAGCGGATTCTTACCATTATTGACCATTTATTTATCAGCAAAATGATAAATGCTGAAACAAGAAAATCAATGAACGAACTTCGCGAATTAGTTCAATCGAGAACAATCTGCTTTCATATGGCGAATTTAGTTGTAATTCAATATTCTTTGCTTGTATCTGTCGCTTCTGTTGTGGTCACTTACACTATTATTCTACTGCAAAGCATAAAGTAA

>HrhoGR4

GATGTAGCAGATGAAATACGAGCTATGATTGTCGAGACAGATCTTAAATCGAAGAGTGCTATTATTGAACTACGGAATATGGTGCAGTCAAGACCGATCACCTTCACCGCTGCTAATTTCTTTAGAATCGACTACGCGCTGCTTGTGTCAACTGCTACAGCTTGCATTACTTATACAATAATCTTAGCAAAACTTTAA

>HrhoGR7

TCAAACGCACAAGCTACTTTCCTATGGACTTTCAATGACATTATGACCATGATGTTCAGCATATATCTCATAGCTTACTTTAGAGATATGAACAGACTGATTTATTCTCCCATACCAAAAAATATCCACGTTTGGAACAGACTAAGAATATTCTGCTCTGAATTGGTATCTCTAGTCCAATTAGTGAACCCCCGCCTCAAATATTTCATACTCAATTCAATTGGATGCAGTTTGTAT

>HrhoGR29

ATGAGCTTAATATGTTGTTTTCCGAAAATTATGTATATTCTTTATGGAATACTAAAACTATATAAAGCAAGGGCATCAGCTTTACCCATAGTGGTGTTAGGTTTCGGAGCTCTCCAATGGGGACTCATGCCGTGCCTCCCTGGTGCGGTTATGGAGTTTGCTTACAATGAAGTGGAAAAGATTAAGAAGACTCTAGTTCACCAGTGTCAATATAATAAAGATGAACTTCTTCGAGAAGATATTAAAGAGTTCATCCAGTACATCGACAGCCGCCCCTACAAATACCGCATCTTACGCATGATCACAGTGGACATGTCGCTGCCTATTGGTCTCCTCAAGCTGTGCACGACTTACTTCATTGTTATTATACAATTCACTCACTTGTTTGAATAA

>HrhoGR64a

ATGAAAGTTAATGGAACGTACAAGAGATATGACCATCAAGATGAAATTCTTGAGAGAGATGAATTTCTTGATACTTTAAACACGATTTTCAAAAAGTCTAGGTGGTTCGGCGTCGCCCCAAGGAGACGTTCGTTGCTCTTTATTTGGGCAATCATTAATGCAGTACTAATGATGGCTGTAGAAGCAGGTGCTATATGGAAACTCATCAGAGCTATTACCGGCACAGTCTTTAACACTGCAGGTGGACACAGCCTGGTCGCCCGGCTATCAGGTTCGATATTCTACGCCAACGGTCTTCTTTCATTGGCGTTATCGTGGCGTTTGATAACATCTTGGAGTTCTGTACATTCGTATTGGATTAAGACAGAATTGAACCGAAGCTTGTTTTTGCCTCCCGATGTTCACATAAAGAAAAGGGTTATTTTTATTACCAGTTTAGTGGTTACGTGCGCTTTAGGAGAACATATTTTAAGTATGATCTCAGCAATAGGCTTCCGATGCCCACCATCAGAGTATATGGAAAAGTATATTCTGGTATCTCATGGATTCCTAATACACAAGAACGAATATCACATCTGGCTGGCGATTCCTATATTTATTGTGAGCAAAACCGCCACCGTATTGTGGAATTTTCAGGATCTGATCATCATTTTGTTGAGTACCGGCCTGACTTCCCGTTATAAAAGATTGAATTCGTACGTAAAGAATTTGGTAGAAATTGAACAAAGCCAAGAAATGAAAAAGCACGCAAATGAAATATATATTGAGGTCCAGACGTGGCGTAGAGTGAGGGAGGCGTACATCCACCAAGCAAACCTGGTGCGGCGTGTGAGCAACAAGCTTGGTGCCTTAATACTGCTGTCCAGCCTAAACAACTTCTATTTCATATGCCTTCAATTATTTCTTGGGATCAATAAAGACAAGGGTGAGATGATAAATCGCCTATACCACTTCCTATCTCTCTCTTGGCTGATGCTCAGAGCGAGCGGCGTGGCGCTGGTAGGTGCTGACGTCGATGAACATTCTAGAAAGGCATTGAAATATATGAAAATGTGCTCAAATCACAACGTTGAGATAGAAAGATTGAAGAATCAAATGAAGAACGATCGAGTTGTCCTTCGAGGTTTAGGTTTCTTTGCGTTGGATAGAAATATGTTCTTGAAGGTAGCAGCTGCTATCATGAAATATGAATTAGTATTGGTACAGTATGACAAATAA

>HrhoGR67

AAAAGAAGAATTGTTGAAGTAGATGAAGCTATTAAAAATATGGATGAGCAAATCGACTACGTCAGACAAGTACATTCAGTGTGGACGATAGCTGTGGCTCCGGTAGTGGTTTCTTTGATGCGTGTGTTCAGTATTTACATTAGCATCGCCACCTCCGATATAGCTGTCCCTATTGAGAAAATGGGTCAACTTATTTATGCTGATATATTTGCGCTACTCATCACCGCCTTACATTGTGGGCACAACAATCTTTTGAGGGAAAGGTTTAAAATTGTTAACGTTACGCTCAGGAAAATTAAAGACAGGAAGGCTTGGTTTCGCGGAGCATTGTTTTCAAGAATATCTATAAGTGACACTAAACATGTTGCGCAACATAGAGAGAAATATATATGCGATAAGATCAAGGCTTGTGCAAAAATTTACGACAAGTTGATGGGCTGTGTGATTTCTTTGAACACAATTTATGGGTTCGCTATGGTCCAGACAATGTCTTTAAGTCTCGTCTACATAGTGCTGTACTTGTTCTACCTGATGGAGGCCACCGCCTCTGGCTTGTACAATGATGCGAATAGATATGTCAACTTCATTTTCTATGTCTCCTGGCAGATATTATATGGAGTCGGTGTCATATTCTTCAATATTCATTATTGTGAGGAAACTGTTAAAGAAGCAAAAATAACATCACGTATTGTG

>HrhoGR68.1

ATGTTCCGTGTAATAAGCAAAATAATTCCTATTTATAGACAATTAAATGTAACGTGGTACAGTGCGTTCAAGCCTATATATTATTTGACATCGTTATTGGGATTATTTCCTTACACTTTGAAACATGAGTCTACAAATGTTTTTAAAACTTATCTAAATTCTTACTACTTAAATTTCATATATGCTTCTATAATTTTTATTATTTTATGTGTGTTCTGCGTGTTACATATTCAAGATGTTATATATGCAGGCAAATCGTACAGCATGACAAATGAAAACCTGACAGAAATAAATTACATATTCGAGTTTGTTTTCTTGTTAGTTTACTGTTTTGTAGCATACTATTGTGCTTTTACAAATGGCAAATTGTATATAAATATTTTAAATCGAGTGATAGCTACATGCCGTCGGACAGCCGTTGATCGATACGAAAAAAATATGAAACTAGTCGATAAAAGGCTAAAAAAAATAGTATATAGTTATACACTATTAGCCGTGACAACGATAATAATCAATTTTACTAGAAAAGGTTCTGTATGGAAATCGGTTCTCGTTTTATTCACATTTATTTTACCCCAAACTGTGCAGTTAGCGACTCTTGCGCACTATTGTTCACTGGTCGTGATGATAACAGGCTTATTGGACAACATAATTTTATGTTTATCTTATTATGATCAAAACAAAAGAGTGGCTCACGGTATTTGGACTACCAAACCTCGAGCTGTATCAATATTGAGCAATTTAAAAACATGCTTTATAAAAGTGTACAACATAAAAGAAGACATCAACAGAGCTTTTCAAGCACCTATTCTGTTTACGGCTATCCAATGTTTCCATTGTCTTGTGAGTGAAGCCGGCAGTTTGTATCATGGAGTTGGG

**SNMPs**

>HrhoSNMP1

ATGAAACTTCGAAAACATTTCAAAATTGCGATCGGTTCCGCAATCGTCGGGGTATTCGGCGTTCTATTTGGATGGCTGATTTTCCCGACCGTTCTCAAAAGCCAGCTGAAAAAGGAAATGGCTTTATCGAAGAAAACAGATGCACGCAAGATGTGGGAAAAGGTACCGTTTGCTCTAGACTTCAAGGTGTATCTGTTCAACTACACAAACGTCGATGAAATACACAAGGGCGGTGTGCCGATAGTCAAAGAGGTTGGACCTTATTACTTCGAGGAATGGAAAGAGAAAGTAGATATCGTTGATAACGATGAAGATGATACCGTAACGTACAAGAAACTCGATACGTTCTACTTCAAAAAGGATAAGTCGGGGCCTGGGCTCACTGGAGATGAAATTATAACTTTGCCACATGCTTTTATTCTTTCATTGGTGACAATCATATCACGGGACAAACCGGCAATGCTAAATATGGCAGGTAAAGCGTTAAATGGAATATTCGACAATCCGCCAAATATGTTCTTGAAAGCCAGAGCATTGGATGTTCTCTTCGATGGCATCAATATTAATTGCGCAAGAACCGAGTTTGCGCCTAAAGCTGTGTGCACAGCTTTGAAAAAAGAAGCCGGCAACCAACTGAAAATTCTTGAAAACAATCAATTTTTATTTTCTTTCTTCGGAATGAAAAATCATACTGTTGACTCTCACGTTGTGAAAGTAAGTCGAGGCATGAAAAACGTAATGGATGTGGCCAAAGTTCTGGAAATCGATGGTAAACCTCAAATGGACAAGTTCCGAGACAAATGCGACTTGTTCGACGGTACAGACGGCACTAAGTTTCCGCCTTTCATGACCAACCAGCCCGTAGCCAGTTTCAGCACTGACACATGCAGAACTTTCAAGCCATGGTACCAAAAGCAATCTTCCTATCAAGGTATTAAGACATTACGCTACATCTCCAACATTGGGGATTACGCCAATGACCCTGAACTGAACTGCTTCTGTGATACACCGGACTCTTGTCCCAAGAAAGGTTTTATGGATGCAACCAAATGCTTGTCGGCGCCTCTGTATGTGACGCTACCACATCTACTGGACTGCGACCCTGAGGAACAGAAAAATGTCAAAGGGCTGAGCCCTGATGTTGAAGCTCATGGGATTGCAATTGATTTTGAGCCGATAACAGGAACACCGATGACTGCTCGACAACGGGTGCAGTTCAATCTACGTTTAATAAAGACAGATAAAATAGAACAATGCAAGGAGTTGCCTGATACCATTGCCCCATTATTCTGGATCGAAGAGGGTTACGCATTAGACCGTGATTTTGTGAAGTTGTTGAAACACCAGCTGTTCTTGCCGAAGCGAATCGTGGGCGTCGTAAGATGGTTGCTAGTGTCGATTGGTATCCTTGGAACATTTGGGAGCCTGGTGTTCCACTTCAAAGACCGCATAATACAATTCGCAATTCCTTCCAACGCAACCTCAGTCACCAAAATCAAACCGGAAGAAGAAAACAAAAAACAAATCAGTGTTATCGGCAATACTCAAGACGCAACTGAACTAGCGAAAATTGATATGTAA

>HrhoSNMP2

ATGATGTACGAGAAATGGCGGAAACTGCCGATGCCGTTAAATTTCAAAATATACGTGTTTAACGTGACAAACGTGGAGGAAGTCAACGCAGGCGCAAATCCAAAGTTGGTTGAAATTGGACCATATGTGTACAAAGAATATAGAGAAAGAACAGACATTGAGGTAACTGATAACGATACAGTGAGATACATGCTGAAGAAGAGCTTCGTCTTCGACGGAGAAGCCTCCGGGTCGAAGACTGAGGACGACATCATTACTGTTATACACTACGCTTACGTGGCTGCTATAGTCCAAGTGCACGACACAATGGCCAGTTTACTGCCAATACTGAACCCAGCGTTGCAGGAGTTCTTTGGAAATGTTTCCAGTCCATTCTTAACGATAAAGGTCAAGGATCTATTTTTCGACGGTATATTCCTCAACTGTAACGGAAGTCAACAATCTTTAGGTTTGATTTGTAGTAAAATTGAAGTTGAAAAACCTCCCACAATGCGACAGGCAGACGGCGGTAACGGTTTTTTCTGGTCGATGTTCGGACATTTGAACCGGACTATAACAGGCCCTTATGAGATGGCCCGTGGCTTAACTAATATCCAGGAACTTGGTCATATAGTGTCTTACCAGGGTAAGAGGGTTATGACTGAGTGGAATGATCCATACTGCGGTCAAATCAACGGCTCCGACTCTACGATATTCCCACCGATCGACGAGAATAACGTTCCCTCAAGGCTATACTCATTTGAACCAGATATCTGCAGGTCTCTCTATATAAGCCTGTCAGAAAAAACCACGCGGTTTAACATGACAGCGTATTTGTATGAGATGGATTCATCCGCTTTGGCATCGAAAAGCGCGAACCCCGACAACAAATGCTTCTGCGACAAGAATTGGAGCGCCAACCACGACGGCTGTTTAGTAATGGGTGTGCTGAACCTCATGCCATGCCAGGGCTCGCCGGCGATCGTCTCTCTGCCTCACTTCTATCTGGCGTCAGAAGAGATACTTAGTTATATAGCTGAGGGCATCGACGCGGTCAAGGAGAAACACAAATCTTACGTTTATATTGATCCGTCGACGGGAGTACCGTTGGATGGATTGAAACGTTTACAATTCAATATCGAGTTGAGGAAAATACCAAATATCAAGCAGTTTGAAAACGTTAAAACTGGATTATTCCCGTTGTTATGGATTGAAGAGGGAGCAGTGTTACCAGAGTCTTTGTTAAGCGAACTACGCCAAGGTCACACTATGATCAAGTATGTAGAAGTGTTCCGTTGGGTCTTGCTAGCGGTGGCACTTATAGTAACAGCAGTCAGCGGGTACTTGGTGGCGAGAGCGAAATCGCTGGTATGGCCGCACCACGCACCCGTCAGTTTCGTTCTGCAGCCTCACGGGATGAGTGAAGTTAATAAAGTGCATTGA
